# Eliciting improved quantitative judgements using the IDEA protocol: A case study in natural resource management

**DOI:** 10.1371/journal.pone.0198468

**Published:** 2018-06-22

**Authors:** Victoria Hemming, Terry V. Walshe, Anca M. Hanea, Fiona Fidler, Mark A. Burgman

**Affiliations:** 1 The Centre of Excellence for Biosecurity Risk Analysis, The School of BioSciences, The University of Melbourne, Melbourne, Victoria, Australia; 2 The Centre for Environmental and Economic Research, The School of BioSciences, The University of Melbourne, Melbourne, Victoria, Australia; 3 The School of BioSciences, The University of Melbourne, Melbourne, Victoria, Australia; 4 The School of Historical and Philosophical Studies, The University of Melbourne, Melbourne, Victoria, Australia; 5 The Centre for Environmental Policy, Imperial College London, London, United Kingdom; University of Waikato, NEW ZEALAND

## Abstract

**Introduction:**

Natural resource management uses expert judgement to estimate facts that inform important decisions. Unfortunately, expert judgement is often derived by informal and largely untested protocols, despite evidence that the quality of judgements can be improved with structured approaches. We attribute the lack of uptake of structured protocols to the dearth of illustrative examples that demonstrate how they can be applied within pressing time and resource constraints, while also improving judgements.

**Aims and methods:**

In this paper, we demonstrate how the IDEA protocol for structured expert elicitation may be deployed to overcome operational challenges while improving the quality of judgements. The protocol was applied to the estimation of 14 future abiotic and biotic events on the Great Barrier Reef, Australia. Seventy-six participants with varying levels of expertise related to the Great Barrier Reef were recruited and allocated randomly to eight groups. Each participant provided their judgements using the four-step question format of the IDEA protocol (‘Investigate’, ‘Discuss’, ‘Estimate’, ‘Aggregate’) through remote elicitation. When the events were realised, the participant judgements were scored in terms of accuracy, calibration and informativeness.

**Results and conclusions:**

The results demonstrate that the IDEA protocol provides a practical, cost-effective, and repeatable approach to the elicitation of quantitative estimates and uncertainty via remote elicitation. We emphasise that i) the aggregation of diverse individual judgements into pooled group judgments almost always outperformed individuals, and ii) use of a modified Delphi approach helped to remove linguistic ambiguity, and further improved individual and group judgements. Importantly, the protocol encourages review, critical appraisal and replication, each of which is required if judgements are to be used in place of data in a scientific context. The results add to the growing body of literature that demonstrates the merit of using structured elicitation protocols. We urge decision-makers and analysts to use insights and examples to improve the evidence base of expert judgement in natural resource management.

## Introduction

Protecting and managing ecosystems requires that we are able to clearly identify, assess and communicate threats, and make effective decisions in a timely manner [1, 2]. Advances in our ability to collect, store and utilise data continue to provide conservation scientists a large array of sophisticated tools for decision-making. For example, citizen science [3], drone technology [4], remote sensing [5], and environmental DNA [6, 7], provide new and practical ways to collect data. This information can be better shared via cloud databases, and used in predictive models, maps, and decision support tools [8].

Despite these advances, the data to inform decisions often is absent, incomplete and uninformative [1, 9–11]. The collection of new data can come at considerable delay and costs, and provides no guarantee of improving decision quality [12, 13]. As discussed by [13], delaying decisions to collect more data is not always risk free, and can lead to adverse conservation outcomes.

To aid decisions when data are insufficient, expert judgement is used routinely in conservation and ecology [14–19]. Examples include threatened species assessments [20–23], environmental risk and impact assessment [24–29], priority threat management [30, 31], protected area management [32, 33], and monitoring targets [11, 34]. In these applications, expert judgements help to parametrise predictive models [14], supplement missing data [30, 35], and assess the likelihood and consequences of current or future hazards such as proposed developments [27, 29, 36].

In conservation, and across many domains, expert judgement is listed as a last resort in terms of scientific evidence [37]. However, this does not mean it should be considered a ‘quick fix’ (i.e. whereby any judgement will suffice), until such time as empirical data can be obtained. On the contrary, such judgements may be used no differently to empirical data in these critical decisions, and in many cases they may never be replaced by empirical data. It is therefore imperative that if we must utilise expert judgements, that they provide the best possible data. This includes appropriately quantifying uncertainty and applying methods which meet the basic standards expected for empirical data, such as transparency, repeatability, and empirical control [38].

Unfortunately, in many environmental applications, expert judgements continue to be elicited using qualitative, or semi-qualitative categorical estimates, constructed scales, or single point estimates without uncertainty [23, 32, 39–44]. These approaches have been criticised as being vague and ambiguous [9, 35, 45, 46], and leading to inconsistent or value laden judgements [9, 17, 47]. Their opaque nature leads to ‘mistrust’ by stakeholders and decision-makers, while a failure to appropriately quantify uncertainty (i.e. providing point estimates only) can mislead or frustrate decision makers [35].

**Fig 1:**
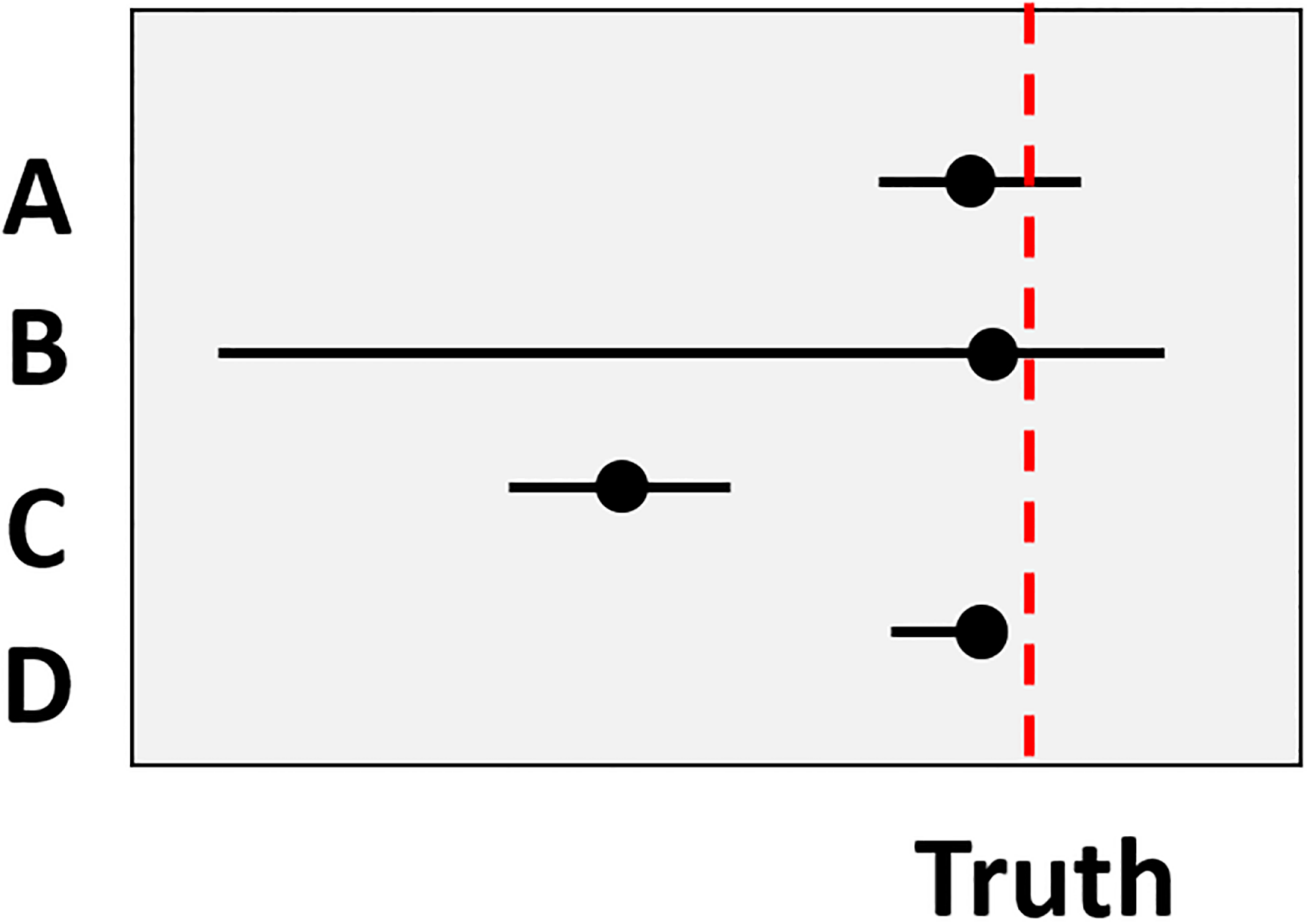
Accuracy, calibration and informativeness for the IDEA protocol explained. The graph shows four hypothetical experts, their best estimates (black dots), and their credible intervals (horizontal lines). The red dashed vertical line represents the realised truth. Expert A has a best estimate close to the realised truth, and their interval captures the realised truth (which over many questions contributes towards their calibration), they are also informative (narrower intervals) relative to Expert B, although Expert B is more accurate (provides a best estimate closer to the realised truth). Expert C is informative but is not accurate and does not capture the realised truth (calibration). Expert D is accurate and informative. However, their bounds do not encapsulate the realised truth (calibration).

Quantitative judgments can include a best (point) estimate of an unknown fact together with an interval defined by credible lower and upper bounds. Whilst the elicitation of quantitative estimates and uncertainty is important, it is equally important that the judgements are as accurate, informative and well-calibrated as possible (we note these terms can vary across the literature depending on the types of judgements being assessed (S1 File)). In this paper, we define *accuracy* as the distance of the expert’s best estimate from the realised truth [48–50]. *Informativeness* refers to the relative width of the intervals provided by experts [51, 52]. An expert providing narrow intervals will be considered more informative than an expert providing wide intervals. *Calibration* typically relates to notions of overconfidence [53] and under-confidence. We assess the calibration of interval judgements, therefore, define calibration as the proportion of questions answered by an expert for which their intervals capture the realised truth [53, 54]. If experts are asked to provide 80% credible intervals, over similar questions, then an expert would be perfectly calibrated if they capture the truth for 8 out of 10 questions. Someone with good judgement would be considered accurate, well-calibrated and informative, although there are often trade-offs between calibration and informativeness (Fig 1) [55, 56].

**Fig 2:**
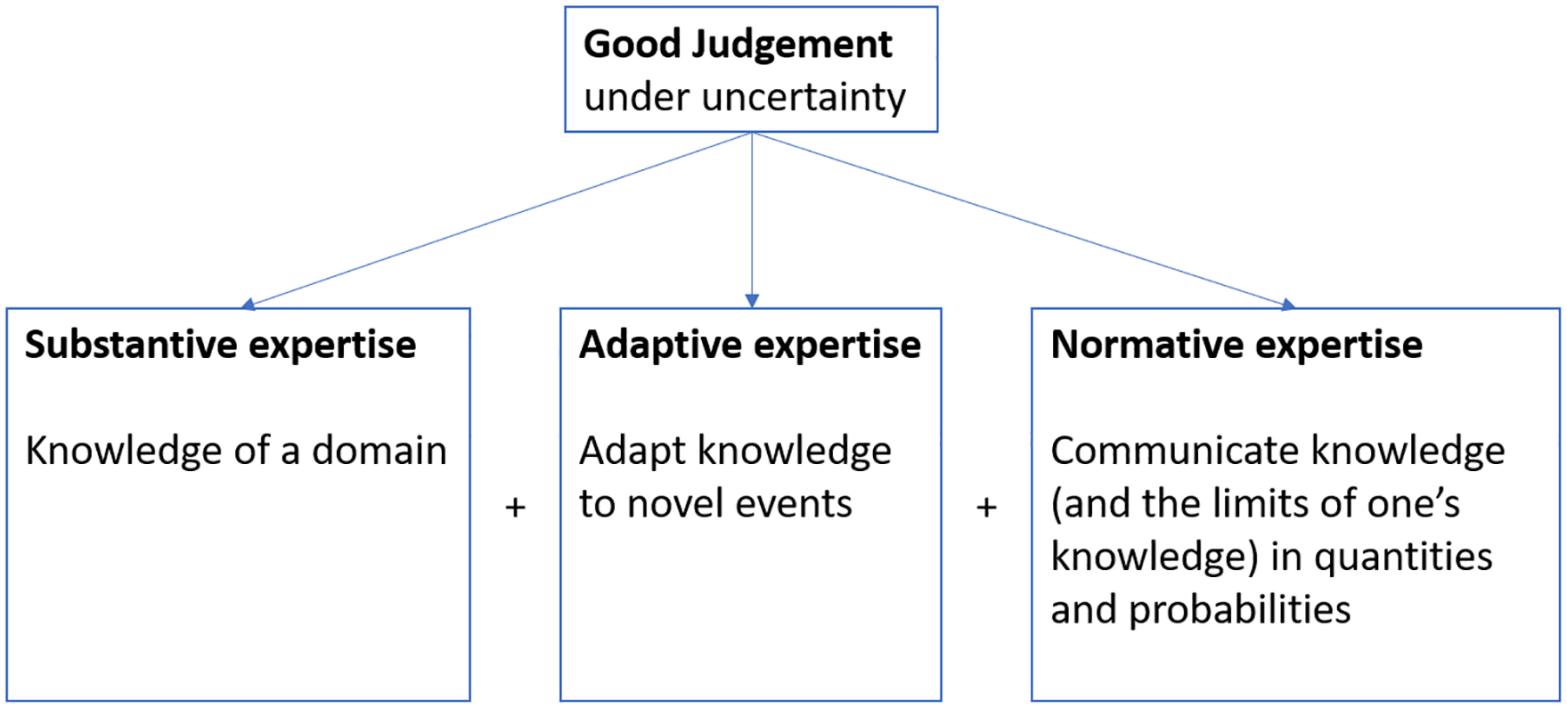
Three essential elements of good expert judgement under uncertainty.

Providing good judgements under uncertainty is notoriously difficult. While many of the people perceived to be experts may have excellent in-depth knowledge and skills in a domain (referred to as substantive expertise), they may be unable to adapt this knowledge (adaptive expertise) to novel circumstances or accurately communicate their knowledge and the limits of their knowledge in numbers and probabilities (normative expertise). All three traits (Fig 2) are essential for judgements under uncertainty [15, 57–59].

A number of factors lead to poor judgement under uncertainty. For example, when asked to make judgements about novel events, people tend to rely on a range of unreliable heuristics [60]. They may anchor on irrelevant information [61], and /or form judgements based on easily recalled events [62]. They may be misled by their feelings about the consequences [63], sourcing information which supports an initial belief [64]. They may be overconfident in their own ability to form judgements under uncertainty [65].

Those relying on expert judgements select experts whom they believe will be capable of providing good judgements. Thus, experts are often selected based on attributes such as years’ experience or education in a subject domain, self-rating, and peer-recommendation [66–68]. This has sometimes led to a reliance on only one expert [18, 69], or if a group is convened, only one expert per subject domain. However, in many domains these attributes have been found to be uncorrelated with a person’s ability to accurately adapt and communicate their knowledge under uncertainty [67, 70–72]. Furthermore, a reliance on these attributes can lead to homogenous and systematically biased group selection (especially in gender, age and credentials), the exclusion of knowledgeable individuals [66], and poor and unsubstantiated judgements (i.e. [18]).

Good judgements under uncertainty are possible, if care is taken to select experts and elicit their judgments. For example, work by [73] and [74] demonstrate that many biases and heuristics can be made to ‘disappear’ if attention is paid to the framing of questions and presentation of information. There is some evidence to suggest that if skills and resources are available, that a person’s estimates will improve if they build explicit models of the system [75, 76]. [77] demonstrated that overconfidence can be reduced in interval estimation by using a four-step question format (often referred to as the four-step elicitation). Many studies have found that structured group judgements usually perform as well, or better than the best credentialed expert [67, 71, 78]. While one individual can sometimes out-perform a group, rarely can that individual be identified *a-priori* [67, 71, 79]. [67] and [80] demonstrated that feedback and discussion followed by the opportunity to revise estimates can be used to effectively resolve ambiguous language (i.e. questions which were interpreted in different ways by two different assessors) and introduce new evidence, helping to further improve individual judgements. [81] undertook a review of 73 studies in the TU Delft database to demonstrate that performance-based weights can be used to further improve group judgements.

Structured elicitation protocols incorporate this research, and are widely advocated as the best means of eliciting expert judgements to help reduce the bias and error associated with heuristics [78, 82–87]. These protocols have been developed because expert judgements can be, and often are, treated no differently to empirical data. Structured protocols therefore restrict elicitations to facts, in the form of numbers and probabilities. Importantly, these protocols aim to apply the same level of rigor to the methods for elicitation and documentation of expert judgements as is expected of the collection of empirical data [38, 83, 86].

While structured protocols are used increasingly in conservation and natural resource management [16, 21, 88, 89], they are not routinely applied. Structured protocols can be expensive and time-consuming [90], particularly if experts are convened in the one location [27, 91]. The limited resources available in most applications in environmental management make elaborate protocols cost-prohibitive.

In some cases, good practice in the elicitation of judgments is actively discouraged. For example, guidelines by the International Union for Conservation of Nature (IUCN) for assessing extinction risk assert that uncertainty can be determined by the ‘opinion of a single expert’ [44]. Furthermore, policies under the IUCN and the Intergovernmental Panel on Climate Change (IPCC) advocate that when group judgments are elicited, that consensus is to be achieved [44, 92]. However, little or no advice has been provided on how to achieve consensus, and the lack of warning about behavioural consensus procedures may have led to the (incorrect) assumption that behavioural consensus should be the goal of the elicitation [93].

**Fig 3:**
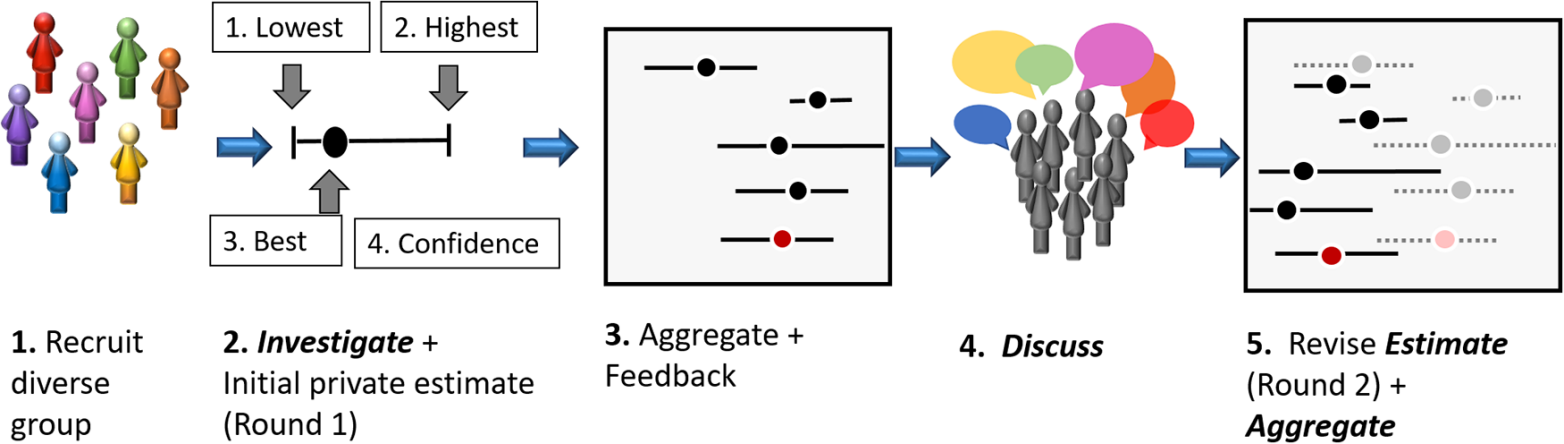
Key steps of the IDEA protocol used in this study and outlined above. In this study we used the four-step elicitation (step 2), which is outlined in Fig 4 below. The question format produces a best estimate (black dots in step 3) with associated credible upper and lower estimates from individuals (horizontal lines in step 3), these are aggregated to form group judgements (estimates marked with red dots in step 3). The results are then discussed by the group, and individuals are enabled to update their estimates (black dots and horizontal lines step 5). These Round 2 judgements are then aggregated (red dots and horizontal lines step 5) and taken as the final estimate. A practical guide to the protocol is provided in [87].

In this paper, we demonstrate how the IDEA protocol for structured expert elicitation may be used in a practical setting typical of many natural resource problems. The acronym IDEA stands for the key steps ‘Investigate’, ‘Discuss’, ‘Estimate’, and ‘Aggregate’. The protocol has been outlined in [80, 94], and an overview and practical guidelines for its implementation are provided in [87]. In brief, the protocol involves (Fig 3):

- Recruit a diverse group of experts to answer questions with probabilistic or quantitative responses.
- Ask experts to first ‘Investigate’ the questions and to clarify their meanings, and then to provide their private, individual best-guess point estimates and associated credible intervals (termed ‘Round 1’) [77, 95].
- Provide feedback on estimates in relation to other experts.
- Facilitate expert ‘Discussion’ of the results, resolve different interpretations of the questions, cross-examine reasoning and evidence, and then provide a second and final private ‘Estimate’ (termed ‘Round 2’).
- ‘Aggregate’ individual estimates mathematically.

Key advantages of the protocol have been highlighted previously [87], including its application within the practical and financial constraints of most decision contexts typical of conservation. Cost-efficient applications use remote elicitation and the four-step or three-step elicitation procedures to obtain probabilities, quantities and uncertainty estimates from experts who may otherwise eschew quantification [21, 22, 96, 97]. The accompanying dialogues can also provide support for the final decision or reveal alternative causal models which require further investigation.

We demonstrate through a case-study, the time and effort required to apply the entire protocol (the recruitment of a diverse group of individuals, the modified Delphi, and group aggregation), and document the extent to which this effort leads to improved judgements. In doing so, we address the following questions:

1. Can the best experts be selected *a-priori*?
2. Do randomly assigned groups outperform the average individual?
3. Do individual and group judgments improve in the second round of elicitation?
4. When aggregating individuals into group judgements, are there advantages to including the first round estimates of those who did not participate in the second round?
5. Do larger groups lead to improved judgements?

A case study was developed for the Great Barrier Reef, Australia, where expert judgement is routinely used to assess trends, conditions and risk to the reef [32, 33, 42]. Currently expert judgement is mostly elicited using qualitative categorical statements (Very Good, Good, Poor, Very Poor), which have been considered to be more practical than quantitative approaches [32, 42]. However, feedback by experts suggests many would prefer a more detailed, transparent and repeatable approach, including the elicitation of uncertainty [32]. The Great Barrier Reef has a large number of monitoring programs for which data are collected relatively frequently, thus enabling expert judgement on questions to be compared to data collected subsequently over a relatively short time-frame, and used to assess accuracy, calibration and informativeness.

## Methods

### Human subjects research

The study was undertaken under the Human Research Ethics Committee of the University of Melbourne (HREC 1546009.1). Participants provided their written consent to take part in the study. The study was undertaken as part of a larger study of 21 estimates which aimed at assessing two questions: 1) whether the IDEA protocol could be used to derive relatively accurate and transparent judgements of continuous variables within a domain of conservation, and 2) how to further improve final judgements through performance-based weights. While participants answered 21 questions, in this paper, we focus on the findings from the first study question relating to the performance of the IDEA protocol on 14 abiotic and biotic questions, without performance-based weighting.

### Question design

Fourteen questions (Table 1), relating to seven abiotic and seven biotic future events on the Great Barrier Reef were developed based on the Great Barrier Reef Outlook Report 2014 [98] (pages 288–292). These questions were reviewed for clarity by agencies collecting the data (the Australian Institute of Marine Sciences, and the Department of Environment and Heritage Protection, Queensland, Australia).

**Table 1:** A summary of the 14 biotic and abiotic questions asked of participants during the elicitation.

Q	Management context	Topic	Abiotic / Biotic
1	Outbreak of crown-of-thorns starfish	Density of crown-of-thorns at Rib Reef	Biotic
2	Bleaching	Reef with ≥1% bleaching (max = 24)	Biotic
3	Outbreak or bloom of species other than crown-of-thorns starfish	Asian Green Mussel detections	Biotic
4	Outbreak of disease	Prevalence White Syndrome (Reef number: 21060)	Biotic
5	Retained take (extraction) of top order predators	Commercial catch coral trout	Biotic
6	Species of conservation concern	Turtle strandings	Biotic
7	Retained take (extraction) of top order predators	Shark control	Biotic
8	Increased sea temperature	Days water temp above 28C	Abiotic
9	Terrestrial point source discharge	Discharge volume Burdekin River	Abiotic
10	Nutrients from catchment run-off	Average chlorophyll Pine Island	Abiotic
11	Climate change effects on weather patterns	Wind Speed Davies Reef	Abiotic
12	Climate change effects on weather patterns	Air temperature Hamilton Island	Abiotic
13	Sediments from catchment run-off	Turbidity High West	Abiotic
14	Increased sea temperature	Sea-surface temp Nino 3.4 region	Abiotic

Questions were compiled into a questionnaire using an interactive PDF form (S2 File). The inclusion of background information can lead to priming and anchoring effects [22, 60], so background information such as past data or trends were only included where it was publicly available, and deemed necessary to clarify the questions.

**Fig 4:**
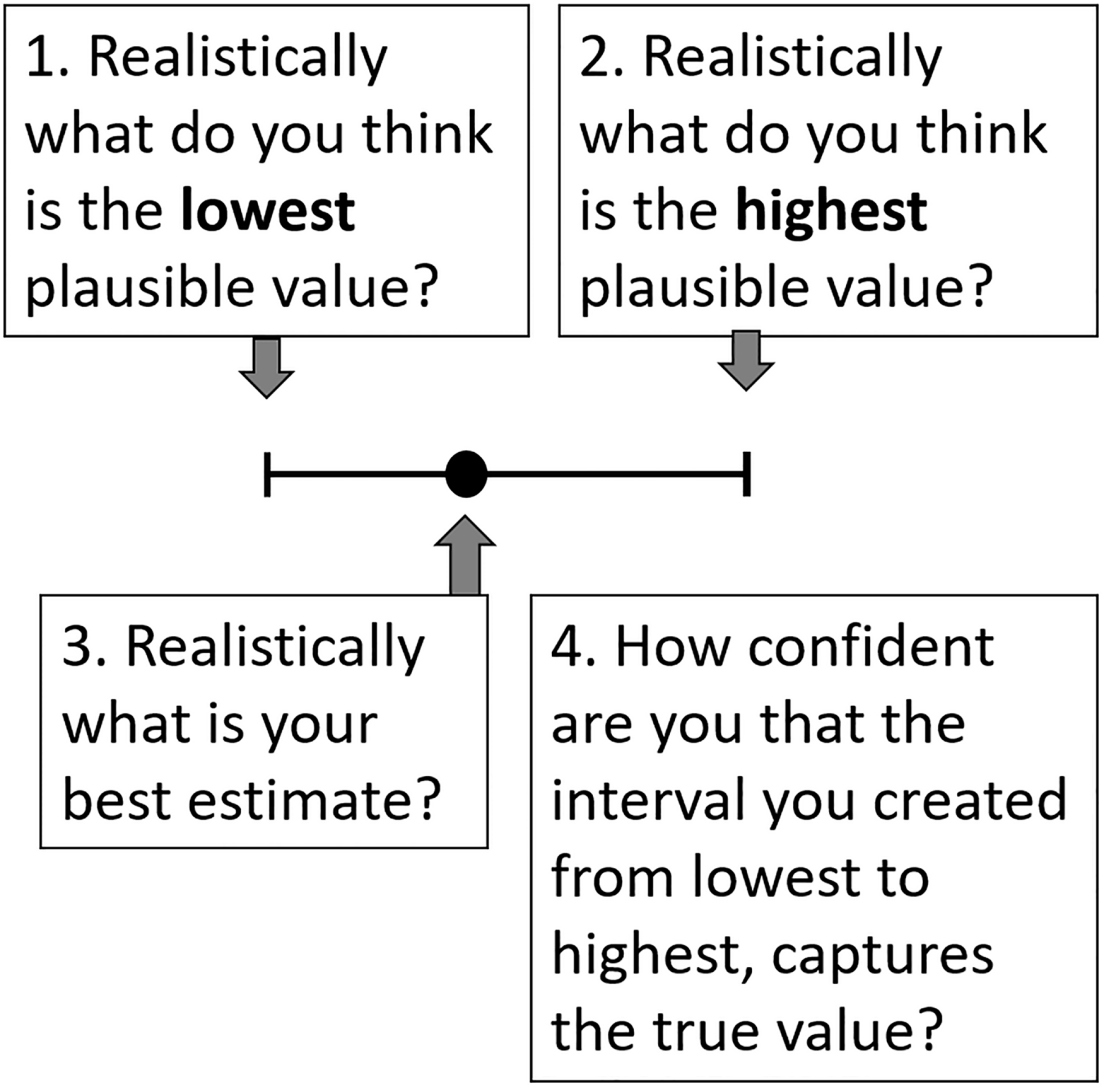
In this elicitation we used the four-step question format [77] outlined in this figure to derive a best estimate (black dot) and upper and lower credible intervals (horizontal lines).

All questions were framed using the four-step question format [77] which derives a best estimate and credible upper and lower intervals for each question (Fig 4. The format was chosen as it has been shown to reduce overconfidence in interval judgements, and helps experts to construct their estimates quantitatively.

### Participants

The study was advertised initially at an international conference in marine science in Sydney, Australia, in January 2016, during which attendees were asked to register their interest in the study. Additional participants were sourced via professional networks of the researchers, peer-recommendation, people who had provided advice in relation to monitoring programs, and through word of mouth (i.e. participants forwarding the study to their professional networks).

In February 2016, a personalised email was sent to 305 potential participants inviting them to be involved in the study, which involved contributing responses to two rounds of questions. A brief project information sheet was attached to the email to outline the aims of the project. Those who agreed to take part were asked to complete a consent form and were invited to a teleconference on 1 March 2016 (S2 File). As part of the consent, those who agreed to participate in the study were requested to create a code name, and to provide basic demographic information and experience related to the Great Barrier Reef (S2 File).

As part of the demographic information, participants were asked to rate themselves (self-rating) and indicate their years of experience across five domain topics that the questions related to: crown-of-thorns starfish (CoTS), coral reef ecology, marine pests and disease (other than CoTS), water quality, and weather patterns (temperature, rainfall, and wind speed). We devised a scale of self-rating between 0–10 which aimed to avoid correlations with years of experience (which has been found to be highly correlated with self-rating [99]), instead taking into account prior knowledge and provision of knowledge to others:

- 0- No prior knowledge or understanding (e.g. you have never heard of this topic before).
- 1‐ Basic understanding, (e.g. have read a report, or news article, but have no direct or relevant experience).
- 5‐ Intermediate understanding (e.g. relevant experience gained through work, study, hobbies, or lay knowledge).
- 10‐ Specialist understanding (e.g. regularly collect data, prepare or sign off on reports, or provide advice on this topic).

In addition, participants were requested to nominate three people who they would perceive as ‘experts’ in relation to abiotic and biotic events on the Great Barrier Reef. In order to encourage inclusion of potentially knowledgeable individuals who may be overlooked as they are not considered experts, participants were also requested to nominate three people who would not necessarily be considered experts but who may have some insight in relation to the domain of questions (denoted as ‘novices’). Note that we reserve the words ‘expert’ and ‘novice’ in describing the results of the study to refer to those recommended by their peers as ‘experts’ or ‘novices’ and use the term ‘participants’ to collectively describe all of those who took part in the study.

From those invited, 101 returned consent forms, 76 of whom completed Round 1, and 58 took part in Round 2. Participants who participated in Round 1 were sourced through professional networks of the researchers (30), peer-recommendation (18 participants plus one additional participant already sourced via the professional networks of the researchers), the international conference (16), word-of-mouth (7) and through contacts made in the development of questions (5). The group consisted of a near equal split between males (40) and females (36).

Peer recommendation led to twice as many men (85) being recommended as ‘experts’ as women (37). For recommendations of ‘novices’ there were similar numbers of recommendations for men (37) and women (31). Only 19 of these recommendations actually took part in the study (experts (13) or novices (6)). Despite the gender bias in expert recommendations, the number of men and women recommended as experts by their peers who actually took part in the study was similar—Round 1 (5 women, 8 men), and Round 2 (4 women and 5 men).

Participants included researchers working on the Great Barrier Reef as well as researchers from Japan, Canada, Italy, and Mexico. The background knowledge of participants was mostly ecological, and mostly marine, but captured a broad range of professions from marine conservationists, to epidemiologists, biosecurity experts and fisheries managers, as well as supporting disciplines such as physics, management and decision science. A summary of the demographics of participants is provided in the S3 File.

### Elicitation

#### Teleconference

A teleconference was held 1 March 2016. During the teleconference, the reasons for the elicitation and the instructions for answering the questions were outlined. A total of 31 participants indicated that they attended the teleconference.

#### Round 1

Immediately following the teleconference all participants (101 people) who had submitted a consent form were sent the set of questions and written instructions (compiled in an interactive PDF form) via email. Participants were given two weeks to complete the survey and return their initial private estimates.

#### Feedback

Following the closure of Round 1, responses were examined for data inconsistencies, for example, numbers in the wrong boxes (the full code is available at [100]). The 76 participants who responded were randomly assigned to one of eight groups of nine or ten people.

The intervals of each participant were standardised to 80% credible intervals using linear extrapolation (Eqs 1.1 and 1.2):

Lower standardised bound:

len,r=be′n,r-((be′n,r-le′n,r)*(jm′en,r)) (1.1)

Upper standardised bound:

uen,r=be′n,r+((ue′n,r-be′n,r)*(jme′n,r)) (1.2)

where, *b*′ = best estimate, *ℓ*′ = lower bound estimate, *u*′ = upper bound estimate, *m*′ = level of confidence given by the participant *e*, in Round *r*, and *j* = the level of confidence each of the intervals was to be standardised to (i.e. 80%). In cases where the adjusted intervals fell outside reasonable bounds (such as below zero for question 2, 3, 4), we truncated intervals at their extremes.

As discussed in [87] it’s often asked why participants are asked to assign their own level of confidence when they are subsequently standardised. [77] found that overconfidence was reduced if participants were obliged to specify their own level of confidence and the credible intervals were subsequently standardised. It’s important to emphasise that the main purpose of the adjusted intervals at this stage is to allow for comparison during the discussion phase. Our experience is that alternative approaches (e.g. using the elicited responses to fit a distribution such as the beta, betaPERT or log-normal) make little difference to the visual representations that result, or to the discussions that follow. Thus, we use linear extrapolations for simplicity. Participants are encouraged to change their estimates in Round 2 if the extrapolation / truncation does not represent their true belief.

**Fig 5:**
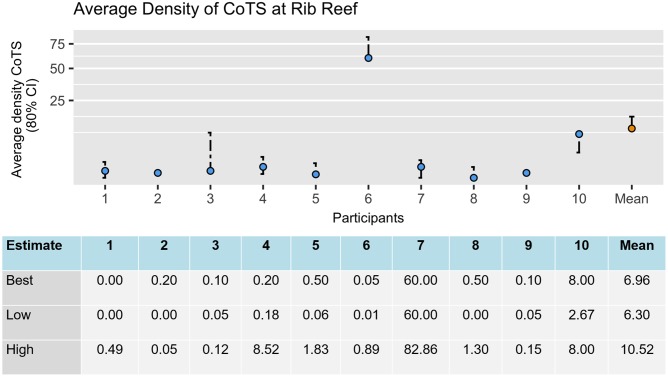
Graphical feedback provided to one group of participants with their Round 1 estimates standardised to 80% credible intervals. The circles represent their best estimates. Note the estimates are plotted on a non-linear square root scale (as this provided the clearest representation of the spread of the estimates). The table below the graph was included to clearly show participants (in numbers) the effect of the standardisation on their upper and lower bounds. CoTS = Crown-of-thorns starfish.

For each question and each group, quantile aggregation [101] using the arithmetic mean was used to calculate an aggregated group judgement for the participants’ best estimate, and their standardised upper and lower bounds [87]. For each group, a graph containing the average and the individual anonymised estimates for each question was compiled into a single PDF feedback document containing only the responses made by members of that group (Fig 5). Additional comments and questions provided by the participants were also compiled (Table 2). Despite considerable effort to develop clear questions, Questions 2 and 8 contained linguistic ambiguities which appeared to have led to large variation in participant responses. These questions were, therefore, further refined and clarified by the facilitator in the feedback documents (S2 File).

**Table 2:** An example, of comments provided by participants when making their Round 1 estimates, and subsequent comments received during the remote discussion phase. CoTS = crown-of-thorns starfish.

Name	Comments	Round / Date
Participant 2	CoTS still seem to be only sporadically present in the Innisfail sector north and upstream of the Rib Reef.	Round 1
Participant 4	Based on data from link given	Round 1
Participant 8	It appears that this is a very broad technique that could be biased by the trained eye of the diver and how conspicuous is the organism.	Round 1
Facilitator	Some good comments here. I’d like to hear from people at the lower and higher ends of this spectrum. Can you elaborate on your reasoning?	Round 1
Participant 3	CoTS are moving south but the numbers (as estimated by the LTMP technique) were still very low in 2015. I expect an increase over the 2015 counts (which were 0.05 per tow according to the web page), but not by >10-times	Discussion 21/03/2016
Participant 7	Excuse me, but fortunately I was wrong to write 60. Whereas the percentage of coral cover is around 40, and analyzing the data, I correct my answer: better value 0.6 and lowest 0.06.	Discussion 22/03/2016
Participant 10	The CoTS are traveling down the GBR. I thought Rib Reef was closer to Innisfail rather than Townsville on reviewing I would lower my best guess to 4	Discussion 29/03/2016

On 18 March 2016, participants were sent feedback documents containing the estimates and comments provided by their groups (see Fig 5, Table 2, and S2 File).

#### Discussion phase

Participants were sent an interactive PDF form to provide additional comments and questions for their group (S2 File). Over 10 days, participants sent their comments to the facilitator (VH), who compiled the comments and questions for each group and circulated the collated discussion via email each day (Table 2).

#### Round 2

On 30 March 2016, the discussion phase ended participants were sent a blank form in which to enter their revised estimates. Participants had until 4 April 2016 at which point the elicitation closed. Of the 58 who remained in Round 2, 45 updated one or more of their estimates whilst the remaining participants confirmed they had reviewed their estimates and did not want to update them.

#### Post elicitation

**Fig 6:**
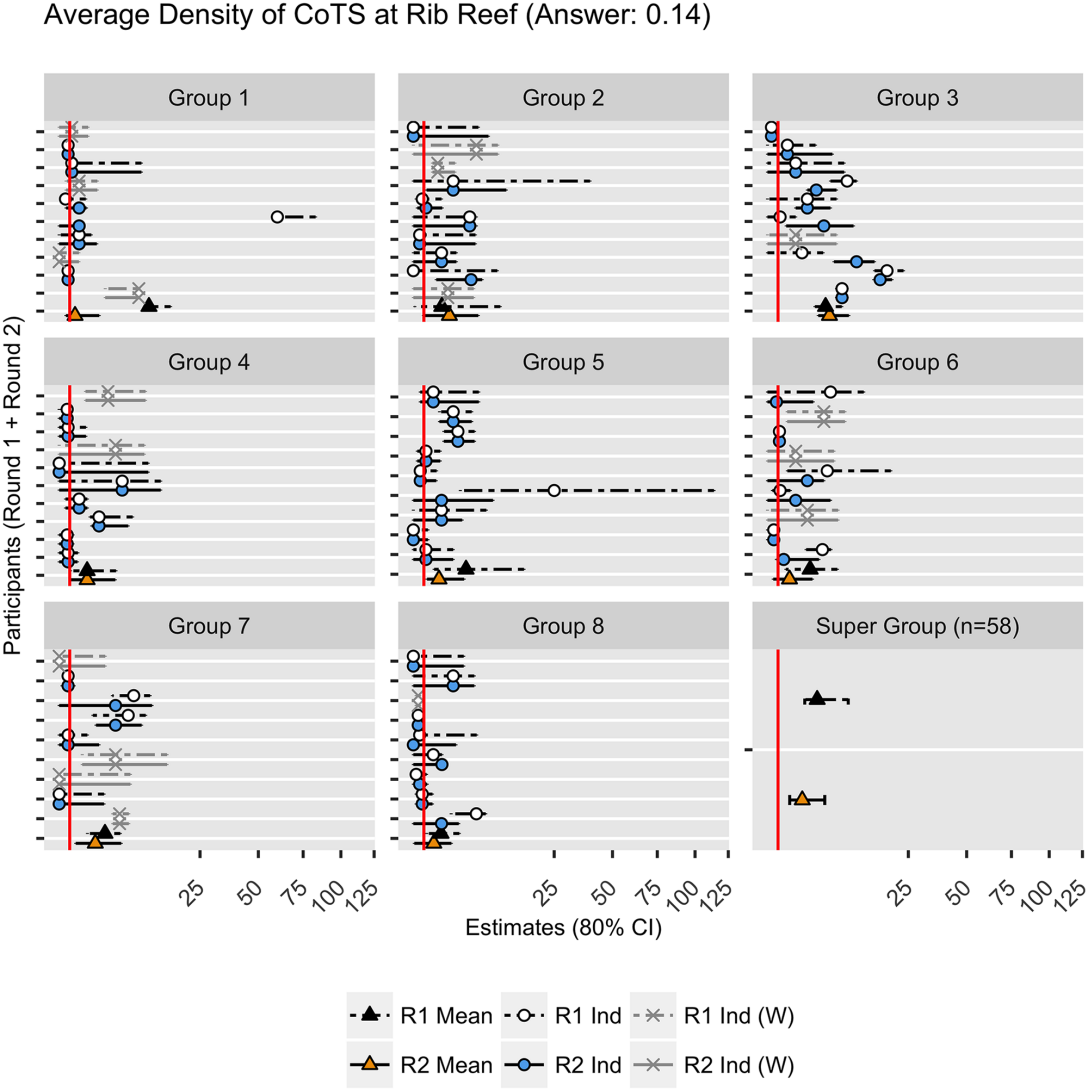
An example of judgments provided by each group for Question 1 of the elicitation. The question asked for the average density of crown-of-thorns starfish (CoTS) that would be detected per 2-minute manta-tow on Rib Reef, Queensland, Australia, by the Australian Institute of Marine Science in 2016. The graph shows the estimates (best estimate, with 80% upper and lower credible intervals) provided by participants in Round 1 (R1 Ind), and then estimates provided in Round 2 (R2 Ind). Participants who withdrew following Round 1 (R1Ind (W) and R2 Ind (W)) were not included in the group aggregation (R1 Mean and R2 Mean). A ninth group (‘Super Group’) was created from the aggregation of all 58 participants who took part in Round 1 (R1 Ind) and Round 2 (R2 Ind). The realised truth (0.14 CoTS), is displayed as a red vertical line. Note that the scale of the x-axis is a non-linear (square root) scale.

**Fig 7:**
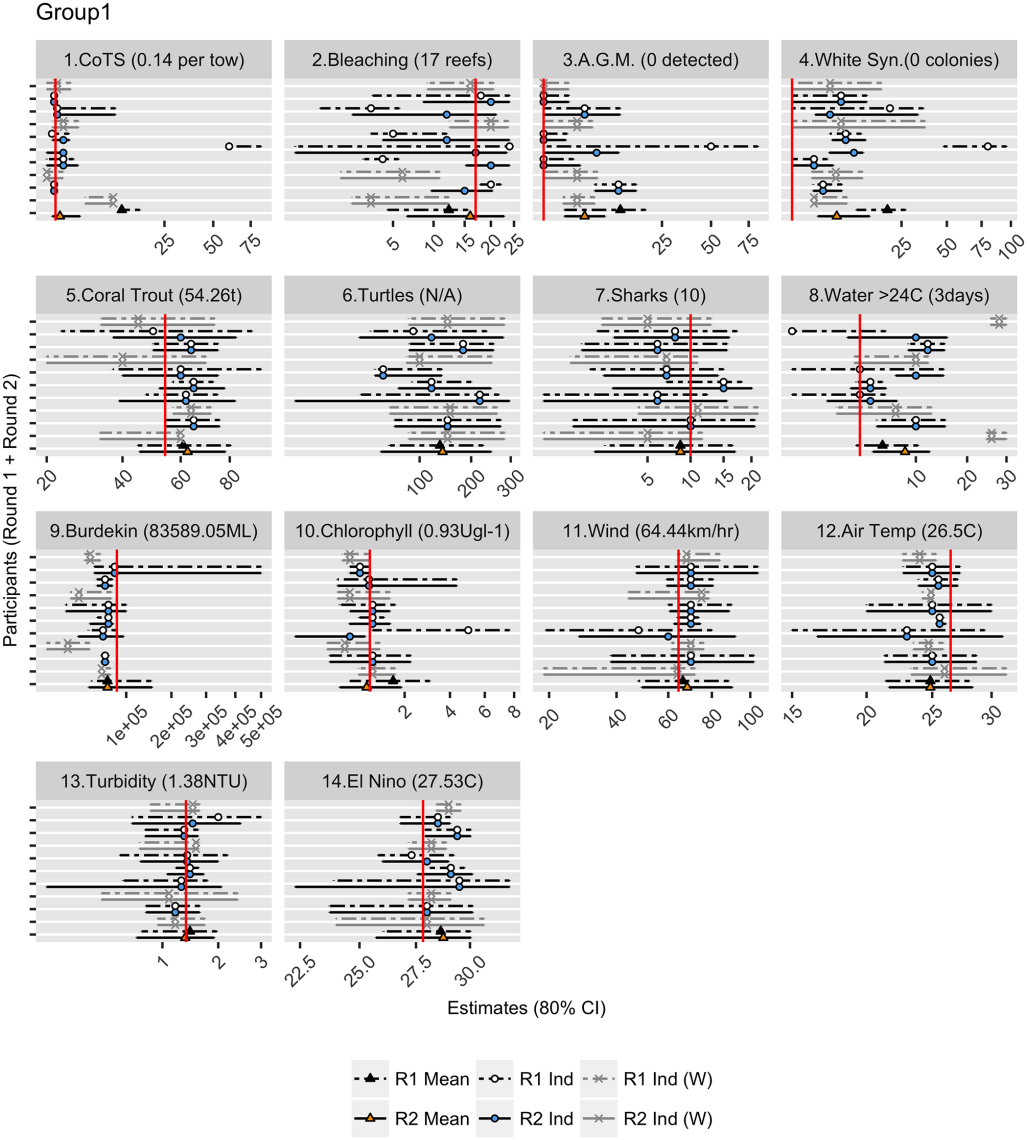
An example of feedback provided for Group 1 for each of the 14 questions. The graph shows the estimates (best estimate, with 80% upper and lower credible intervals) provided by participants in Round 1 (R1 Ind), and then estimates provided in Round 2 (R2 Ind). Participants who withdrew following Round 1 (R1 Ind (W) and R2 Ind (W)) were not included in the group aggregations (R1 Mean and R2 Mean). The realised answers for each question are displayed above the graphs and indicated by the red vertical line. Note that the scale of the x-axis is non-linear (square root) scale.

The first question was able to be validated on the 12 May 2016 (Question 8), the last questions (Questions 10 and 13) were not validated until 5 October 2016 due to problems accessing the sites where the data loggers were stored. Question 6, which related to the number of turtle strandings, was not able to be validated as the data collection and reporting methods were unexpectedly changed during the survey period. Fig 6, provides a graphical summary of quantitative judgements for each participant and each of the eight groups for both Round 1 and Round 2 against the realised truth for Question 1 relating to the density of crown-of-thorns starfish at Rib Reef. Fig 7, shows the variation in estimates of a single group for all 14 questions. We also included a ‘Super Group’, based on the aggregation of standardised estimates from all 58 participants for each question.

### Scoring assessments

Once the realised answer to the questions became available, the accuracy, calibration and informativeness of participant judgements could be calculated.

In our study, we used the four-step question format, which elicits a best estimate, and an associated credible interval, to reduce overconfidence in interval judgements, and assists in helping participants to encode their knowledge quantitatively [77]. However, it is important to note, that the method does not define what the best estimate represents (i.e. a mean, a mode, a median). Likewise, participants are only asked to describe their confidence that the truth falls between their upper and lower bounds, not to specify how the residual uncertainty may be distributed outside of their bounds (i.e. quantiles of a distribution). Therefore, the method was not designed, on its own, to elicit a probability distribution. Additional questions could provide this information.

These properties mean that our data may be inappropriate for those scoring rules in the literature which centre around continuous probability distributions, such as those employed by the Classical Model [56]. We therefore utilise scoring rules which score the participant’s ability to provide an accurate best estimate, and well-calibrated and informative interval judgements.

#### Accuracy

‘Accuracy’ is used as a measure of performance for the best estimate (a point estimate). It aims to assess the difference between the prediction *b* (the participant’s best estimate) and observed value *x*.

Commonly applied measures of accuracy include Mean Absolute Percentage Error (MAPE), which gives the average percentage difference between the prediction and observed value, and Root Mean Square Percentage Error (RMAPE), which is the square root of the MAPE [102]. Both MAPE and RMAPE are strongly affected by one or a few very divergent responses [102].

To overcome these limitations, [67] outlined an alternative approach, which we adopt. The approach involves first standardising the best estimates $b_{e}^{\prime n,r}$ from each participant *e*, for each question *n*, in each round *r* (including the realised outcome) by the range of responses for each question. This is termed ‘range-coding’ and is given by,

ben,r=(be′n,r−bmin′n)(bmax′n−bmin′n) (2)

where, $b_{e}^{n,r}$ is the range-coded response for participant e, in round r, $b_{max}^{\prime n}$ is the maximum best estimate response taken from the pool of responses (best estimates) from all participants for question *n*, across both Round 1 and Round 2, and $b_{min}^{\prime n}$ is the minimum best estimate response. Note that the realised truth (*x*′^*n*^) for each question is also range-coded using Eq 2.

Range-coding reduces the contribution of the question scales on the results. The range-coded values are then used to calculate performance using the average log-ratio error (ALRE, [67]):

ALREer=1Nr∑n=1N|log10(xn+1ben,r+1)| (3)

where, *N*^*r*^ is the number of quantities assessed in any round *r*, $b_{e}^{n,r}$ is the range-coded prediction, and *x*^*n*^ is the range-coded observed (true) value for question *n* (range-coded values are derived from Eq 2 above). A ‘1’ is added to avoid taking the log of zero (which occurs when the realisation is standardised). The log_10_ ratio provides a measure that emphasises order of magnitude errors rather than linear errors. That is, a prediction that is 10 fold greater than the observed value weighs as heavily as a prediction which is one-tenth the observed value [74]. Smaller *ALRE* scores indicate more accurate responses. For any given question, the log ratio scores have a maximum possible range of 0.31 (= log_10_(2)), which occurs when the true answer coincides with either the group minimum or group maximum.

#### Calibration

In this paper, we refer to ‘calibration’ in terms of interval judgements in which a judge is considered well-calibrated if over the long run, for all questions answered, the proportion their intervals that capture the realised truth equals the probability assigned [53, 54, 65, 103].

As the information from the four-step elicitation involves a standardisation of intervals, we use the standardised upper and lower values of those intervals and the standardised level of confidence associated with those intervals. Scoring participants on their standardised intervals is thought to be acceptable as the participants receive feedback on these standardisations between Round 1 and Round 2 and are informed they can (and should) adjust their estimates if they do not accord with their true beliefs. They are also made aware that this this is how they will be scored.

In this study, we standardised intervals to 80%, therefore a perfectly calibrated individual will capture the realised truth approximately 80% of the time. We calculated the actual number of realisations captured as,

$$C_{e}^{r}\;=\;\frac{t^{r}}{N^{r}}\;\times 100$$ (4)

where, $C_{e}^{r}\;$ is the score for calibration for participant *e* in Round *r*, while *t* is the number of standardised intervals provided by the participant which contained the realised truth, and *N*^*r*^ is the total number of questions answered by the participant in round r.

This scoring rule follows that used by [67], [77], and [104] for evaluating performance of intervals derived from the four-step elicitation. As it is possible for participants to obtain a high calibration by providing very wide (uninformative) intervals, this measure must be considered alongside informativeness (described below).

#### Informativeness

For this study, we were concerned only about the width (or precision) of the of the participant’s intervals relative to the total range provided by participants for a question. We term this ‘informativeness’ (in accordance with [52]). This differs from the relative information score [83] described by [104], which scores information within, and outside each of the participant’s quantiles (information which we did not elicit in this study) relative to a uniform or log-uniform distribution.

The informativeness of participants was given by the width of standardised intervals (e.g. 80%) supplied by participants for each question in each round:

$$w_{e}^{n,r}\;=\;u_{e}^{n,r}-l_{e}^{n,r}$$ (5)

where, $w_{e}^{n,r}\;$ is the width of the standardised interval of participant *e* for question *n*, in round *r*, while $u_{e}^{n,r}$ is the upper standardised estimate provided by participant *e* for question *n*, in Round *r*, and $l_{e}^{n,r}\;$ is the lower standardised estimate provided by participant *e* for question *n*.

For each question, a background range was also calculated

$$w_{max}^{n}\;=\;u_{max}^{n}-l_{min}^{n}$$ (6)

where $w_{max}^{n}$ is the background range created for question *n*, $u_{max}^{n}$ is the highest standardised upper bound estimate provided for question *n* across Round 1 and Round 2 by any participant, and $l_{min}^{n}$ is the lowest standardised lower bound estimate provided for question *n* across Round 1 and Round 2 by any participant. The background range included estimates from both Round 1 and Round 2 so that changes in informativeness for participants between rounds could be compared on the same scale.

The average informativeness score of each participant per round was calculated by:

$$I_{e}^{r}\;=\;\frac{1}{N^{r}}\sum_{n=1}^{N}\left|\frac{w_{e}^{n,r}}{w_{max}^{n}\;}\right|$$ (7)

where $I_{e}^{r}\;$ is the average informativeness of participant *e* in Round *r* (either Round 1 or 2) over all questions in Round *r*, ${\;w}_{e}^{n,r}$ is the width of the interval provided by participant *e* in Round *r* for question *n*, $w_{max}^{n}\;$ is the background range for question *n*, and *N*^*r*^ is the total number of questions answered in Round *r*.

Scores range between 0 (no uncertainty), to 1 (participant’s intervals were always equal to the background range of the questions). Lower scores are better.

Note that the score must be considered in conjunction with calibration as it may reward participants who report no uncertainty. In this case, unless the participant knows the truth with absolute certainty, they would be expected to have poor calibration, which is often weighted higher than informativeness by a decision maker.

#### Proper scoring rules

Proper scoring rules are those for which an assessor receives their best score if they provide their true beliefs [105, 106]. Theoretically, proper scoring rules cannot be gamed. Currently the scores for the IDEA protocol have not been assessed as to whether they meet the definition of proper scoring rules. However, participants are told that they will be assessed on their accuracy, calibration and informativeness. This makes it very difficult to game the scores, and hopefully provides adequate disincentives for any gaming behaviour.

### Analysis

#### Boxplots

To compare individual and group judgements within and between rounds, we developed boxplots to compare between samples. Each of the boxplots was constructed in R (version 3.4.1 (2017-06-30) — ‘Single Candle’), using the ggplot2 package. The boxes represent the 25^th^, 50^th^ and 75^th^ percentiles, otherwise known as the lower quartile (Q1), median (m or Q2), and the upper quartile (Q3) or interquartile range (IQR, where 50% of the data lies). The whiskers represent the spread of the data (Q1-1.5*IQR, Q3+1.5*IQR), for normally distributed data this is approximately 2.7 standard deviations, or 99.3% of the data [107].

Each boxplot included notches (calculated as $1.58\;*\;\text{IQR}/\sqrt{n}$), where *n* represents the number in a sample. Notches approximate a 95% confidence interval for the median [107], and were included to assist in judging between sample medians. In general, if the samples are normally distributed or large, and the notches do not overlap then it may be considered strong evidence that the two medians differ [107]. For small samples the notches may span a larger interval than the box. When this occurs, care needs to be taken in interpreting the results. Each boxplot was also overlaid with a dot plot where each dot represents the score of one individual or group.

#### Linear regression

To assess whether there were relationships between years of experience or self-rating of participants (1 = Low to 10 = High) with accuracy in domain, a regression analysis was undertaken. The analysis utilised the ALRE score of each of the 76 participants in Round 1 in relation to questions in five subject domains (bioinvasions (questions 1, 3, and 4), climate and weather (questions 11, 12, and 14), coral reefs (question 2), crown-of-thorns starfish (question 1), and water quality (questions 8, 9, 10, 13)). The regression analyses were undertaken using the lm() function in R, and the regression lines were overlaid on a scatter plot created in ggplot2. A two-tailed spearman’s correlation test was also performed (using the corr.test() function in R).

#### Making sense of improvements

Accuracy is a relative score, making assessment of the practical importance of any differences between individuals, groups and rounds difficult. In addition, the survey was undertaken outside a clear decision context, and each of the survey questions is measured on different scales and influenced by differing levels of background variation. For example, crown-of-thorns starfish (the subject of question 1) are considered by the Australian Institute of Marine Science to be at incipient outbreak levels when they reach densities of 0.22 per 2-minute manta tow. Judgments close to this critical threshold may be of very considerable consequence. On the other hand, the discharge of the Burdekin river is highly variable (the average discharge for April between 2010 and 2015 was 93,2520 ML with a coefficient of variation of 1.17), thus small differences in judgments would be of little consequence.

To determine whether the changes to best estimates made by participants between Rounds 1 and 2 were meaningful, we defined grain sizes for each question. We used the standard error of data collected in previous years or designated thresholds provided by data agencies to determine grain size (S3 File). Participants who made changes above the specified grain size were assumed to have made a change of some consequence.

We then calculated the number of questions for which individuals and groups improved their accuracy and the number that reduced their accuracy. We used the specified grain sizes to determine how many of these questions involved substantial improvement (or reduction) in accuracy, according to the specified grain-size (S3 File).

Individuals and groups did not always improve their accuracy through updating. Therefore, we also calculated the proportion of questions updated by individuals and groups for which they improved their accuracy. A score below 50% would indicate that individuals reduced their accuracy more often than they improved it.

## Results

### Individual performance Round 1

We aimed to determine whether performance on the questions was associated with professional or demographic data of the participants. To undertake this analysis, we utilised the responses of all 76 participants in Round 1 (prior to any interaction effect).

We found there were no clear difference in the median performance of participants in terms of accuracy, calibration or informativeness, which could be attributed to any of the following characteristics (numbers in brackets reflect number of participants in each category): whether participants had previously been asked to provide expert advice on the Great Barrier Reef (‘Yes’ (55) / ‘No’ (21)); how they had been sourced (‘Advertisement’ (7), ‘Conference’ (16), ‘Participant Recommendation’ (18), ‘Professional Networks’ (30), ‘Project Assistance’ (5)); their affiliation (‘Government’ (25) / ‘University’ (33) / or ‘Other’ (18) (e.g. Consultancies, NGOs etc)); their salutation (‘Prof / Dr.’ (41), ‘Miss / Mrs /Ms’ (13), and ‘Mr.’ (21); their nationality (identify as ‘Australian’ (38) or ‘Other’ (30)); or their Age (‘21–30’ (8), ‘31–40’ (28), ‘41–50’ (13), ‘50–60’ (21), ‘60+’(6)).

There was some evidence (indicated by non-overlapping notches (95% confidence intervals)) to suggest that those who published at least one technical report or peer-reviewed publication (54) were better calibrated than those that had not (22), with a median difference of 0.154 in calibration (the equivalent one additional realisation captured). However, a linear regression found no relationship between calibration and the number of peer reviewed or technical publications.

**Fig 8:**
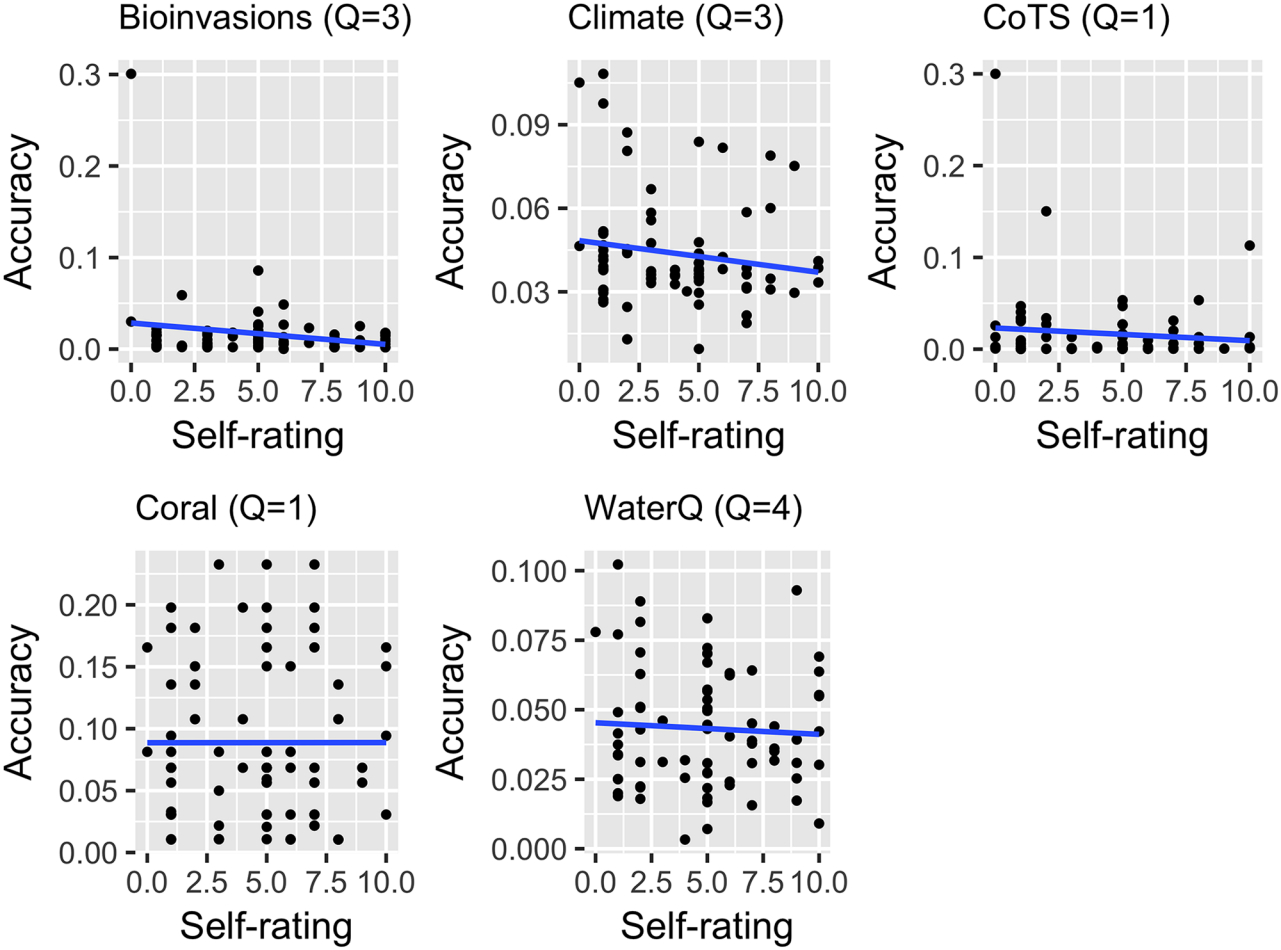
Relationship between self-rating (0 = no experience, 10 = specialist understanding (e.g. regularly collect data, prepare or sign off on reports, or provide advice on this topic) and accuracy (lower number = more accurate) for each of the 76 participants, across five different subject domains in Round 1. The ‘Q’ indicates to the number of questions from which accuracy was scored. The linear models revealed slopes between less than -0.002 and 0 and adjusted R2 values between -0.02 to 0.02 (not significant at a 0.05 level). Spearman’s rank correlations ranged between -.01 and -0.17, none of which were significant at the 0.05 level (of a two-tailed statistical test).

We also explored the correlation between years of experience and self-rating, and found that while there was a positive and asymptotic relationship between self-rating and years of experience, there was no important correlation with either factor and performance on questions in the domain of putative expertise Fig 8.

**Fig 9:**
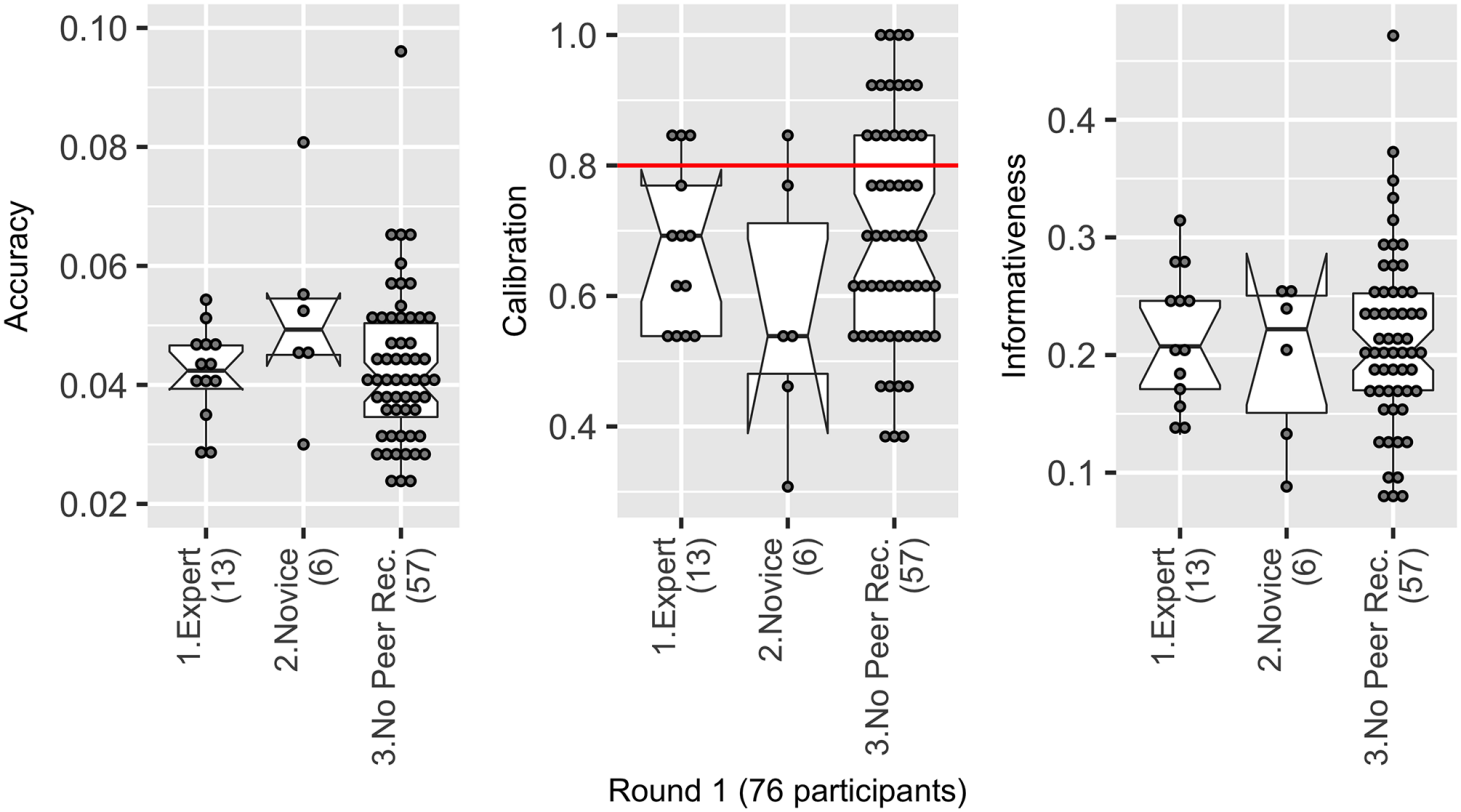
There was no detectable difference in the accuracy (ALRE), calibration or informativeness of those recommended as experts or novices. In fact, some of the most accurate (lower ALRE score) and well-calibrated (a score of 0.8 represents perfect calibration) individuals were sourced through other means.

Six participants who took part in the study had been recommended as novices, and thirteen as experts, by their peers. A comparison of medians of the two groups indicates that novices may have been slightly more overconfident, and less accurate than experts. However, the two groups display considerable variation and overlap, and the sample size of novices was small, making the evidence inconclusive (Fig 9). Interestingly, the most accurate and well-calibrated individuals were not recommended by their peers to take part in the study.

**Fig 10:**
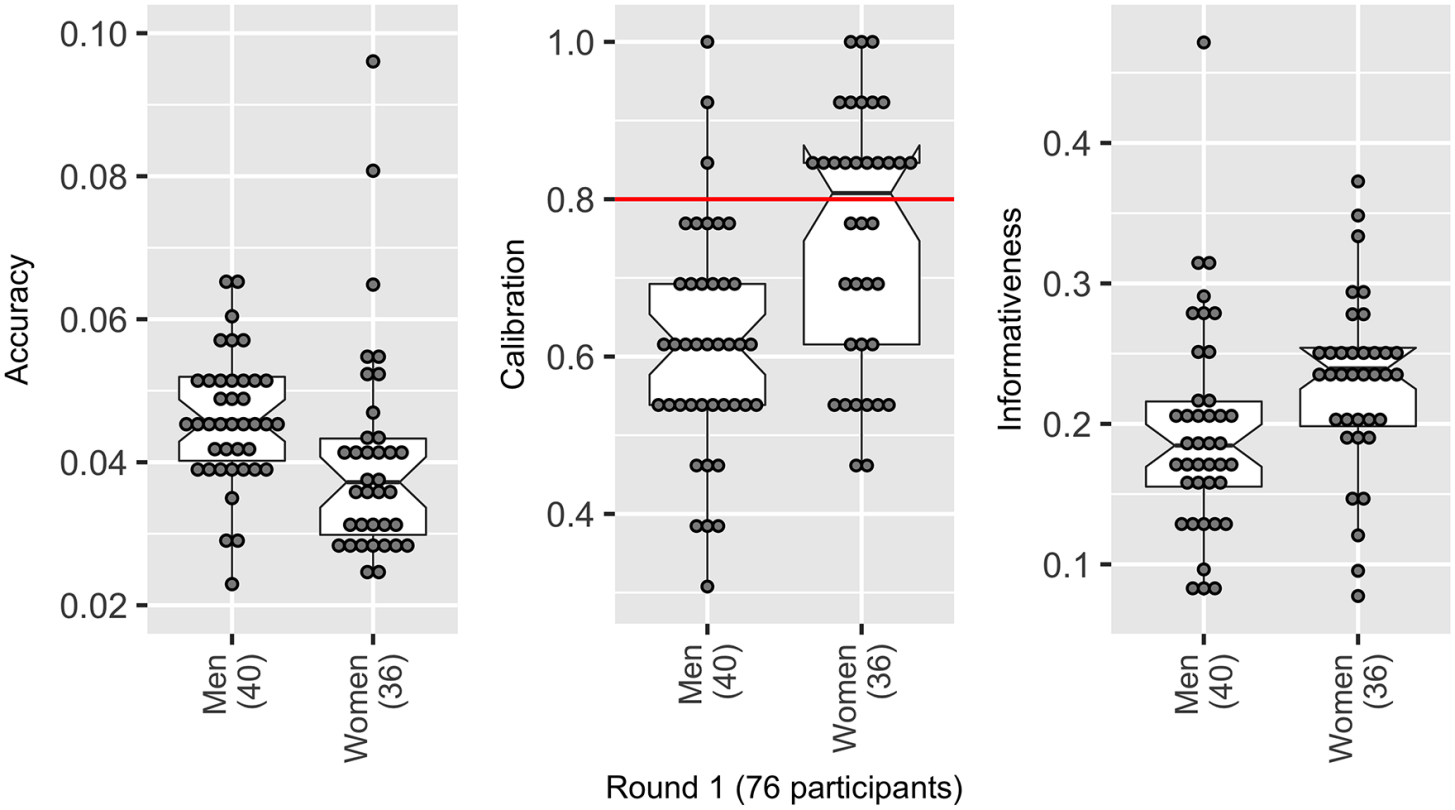
Women were on average more accurate (lower ALRE score), better calibrated (a score of 0.8 represents perfect calibration) but less informative than men (higher numbers relate to less informative individuals).

The only variable for which the analysis indicated there was strong evidence for a difference in medians, was based on gender (Fig 10). Men were on average less accurate (a difference in medians of 0.01 or 13.5% of the total range in ALRE scores (the range was 0.074)), and more overconfident (0.61, [95%CI: 0.58, 0.65]) than women (0.81, [95%CI: 0.75–0.87]), a difference in medians of 0.19 (i.e. the equivalent of 2.5 fewer realisations captured than women). Whilst women were better calibrated, they were on average less informative than men (by 0.05, or 5% of the background range).

### The ‘wisdom of the crowd’

Whilst no demographic attribute or descriptor of expertise clearly predicted better performing participants *a-priori*, randomly assigned groups (*n* = 8) each containing 5–9 participants (i.e. only those who participated both Round 1 and Round 2) performed remarkably well.

In Round 1, most groups had an accuracy that was equal to or better than the median individual (group median of 0.032 [CI95%: 0.030–0.034], compared to the median individual = 0.042, [CI95%: 0.039–0.046]), an improvement of 13.5% of the range in accuracy scores. Groups and individuals had the same calibration (0.69) and similar informativeness scores (individuals = 0.20, groups = 0.19).

**Fig 11:**
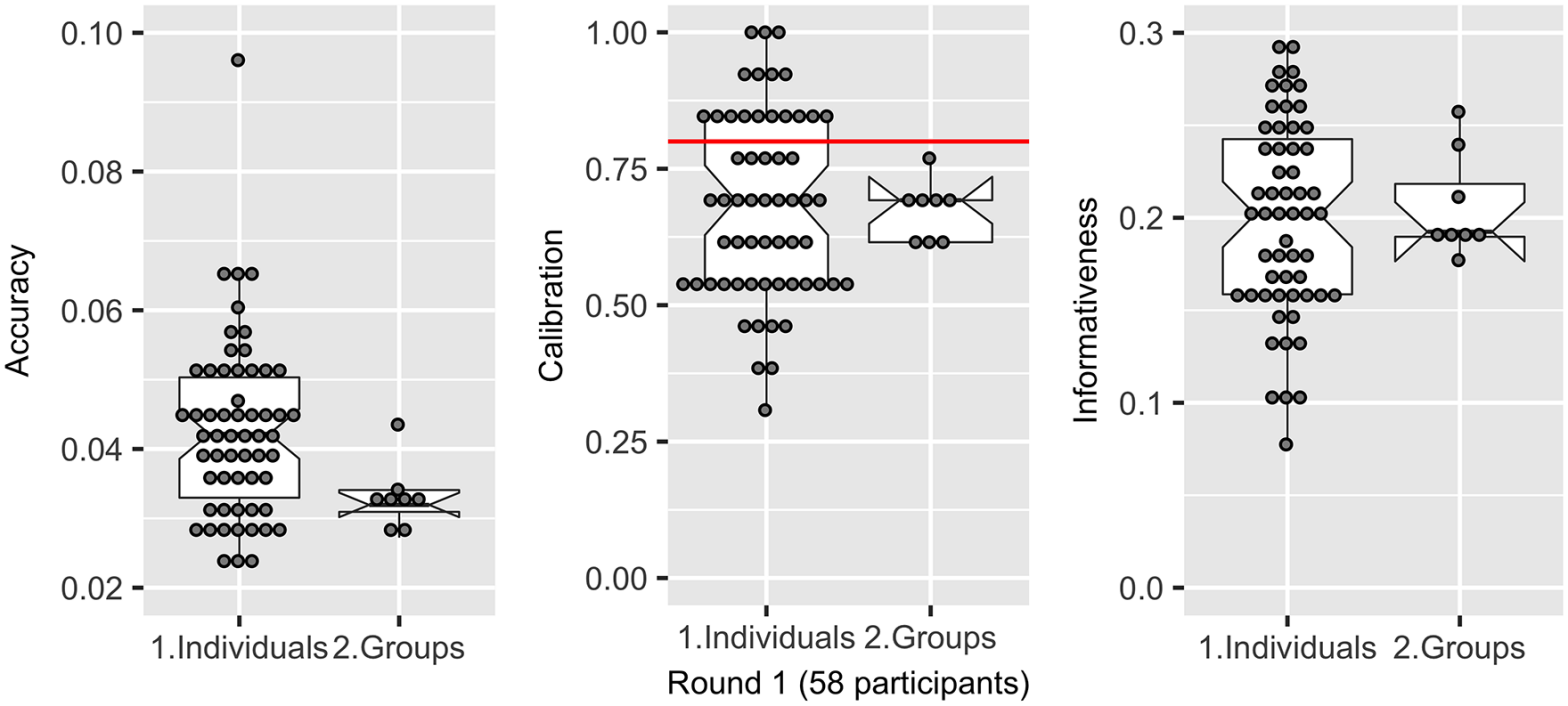
Comparison of the accuracy, calibration and informativeness of individuals and groups. The graphs show that groups were generally more accurate (lower number) than the median individual. Groups had a similar calibration and informativeness score, however, they had consistently lower variance (MAD) than individuals.

While groups were often equal to or better than the median individual in terms of accuracy, calibration and informativeness, they also had considerably less variability in their scores than individuals (indicated by lower Median Absolute Deviation MAD (as shown in Fig 11) making them a more reliable option than trying to select a single individual *a priori*.

### Individual improvement in Round 2

**Fig 12:**
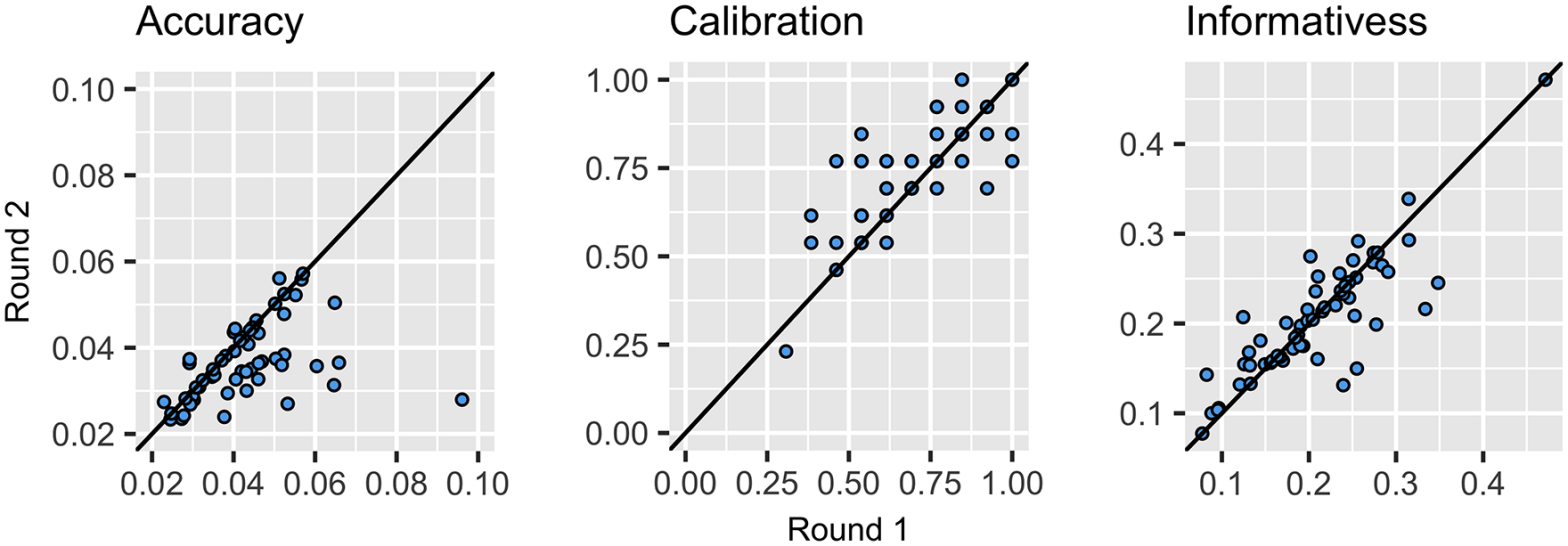
Scatterplots show the change of each individual (n = 58) in Round 2 across the three variables (accuracy, calibration, and informativeness). If dots fall below the line for accuracy or informativeness it shows that individuals improved their scores on these measures. For calibration dots above the line indicate individuals increased the number of realisations captured between their upper and lower bounds (a score of 0.80 represents perfect calibration).

Of the 58 participants who remained in Round 2, 44 updated one or more best estimates, while 45 updated their credible intervals for one or more questions (Fig 12). Participants were more likely to update their credible intervals than their best estimates, updating their intervals for a median of 7 questions (95%CI: 5–9), and their best estimates only on a median of 3 questions (95%CI: 2–4).

Fig 12 clearly illustrates an improvement in accuracy and calibration upon revisiting judgments after considering insights gained from discussion and feedback. Of those who updated their best estimates, most (36 out of 44 participants) improved their ALRE score, the median improvement was 0.008 (11% of the total range of ALRE scores, 95%CI: 0.005–0.011), whilst those that reduced their accuracy (increased ALRE scores) in Round 2 (8 participants), changed their ALRE score by a median of 0.004 (5.5% of the total range).

Approximately half of the participants who updated their credible intervals (25 out of 45), improved their calibration, improving by a median of 0.077 (equivalent to one additional realisation captured). Of the remaining participants, 12 updated at least one of their estimates but it made no difference to their calibration, and 8 decreased their calibration, by a median of 0.077 (equivalent to one less realisation captured).

Although informativeness changed on an individual level, there was no consistent direction, with some individuals becoming more informative (reducing the width of their intervals), and others less informative (increasing the width of their intervals). Overall, the median informativeness of individuals remained the same (0.20).

### Group improvements in Round 2

**Fig 13:**
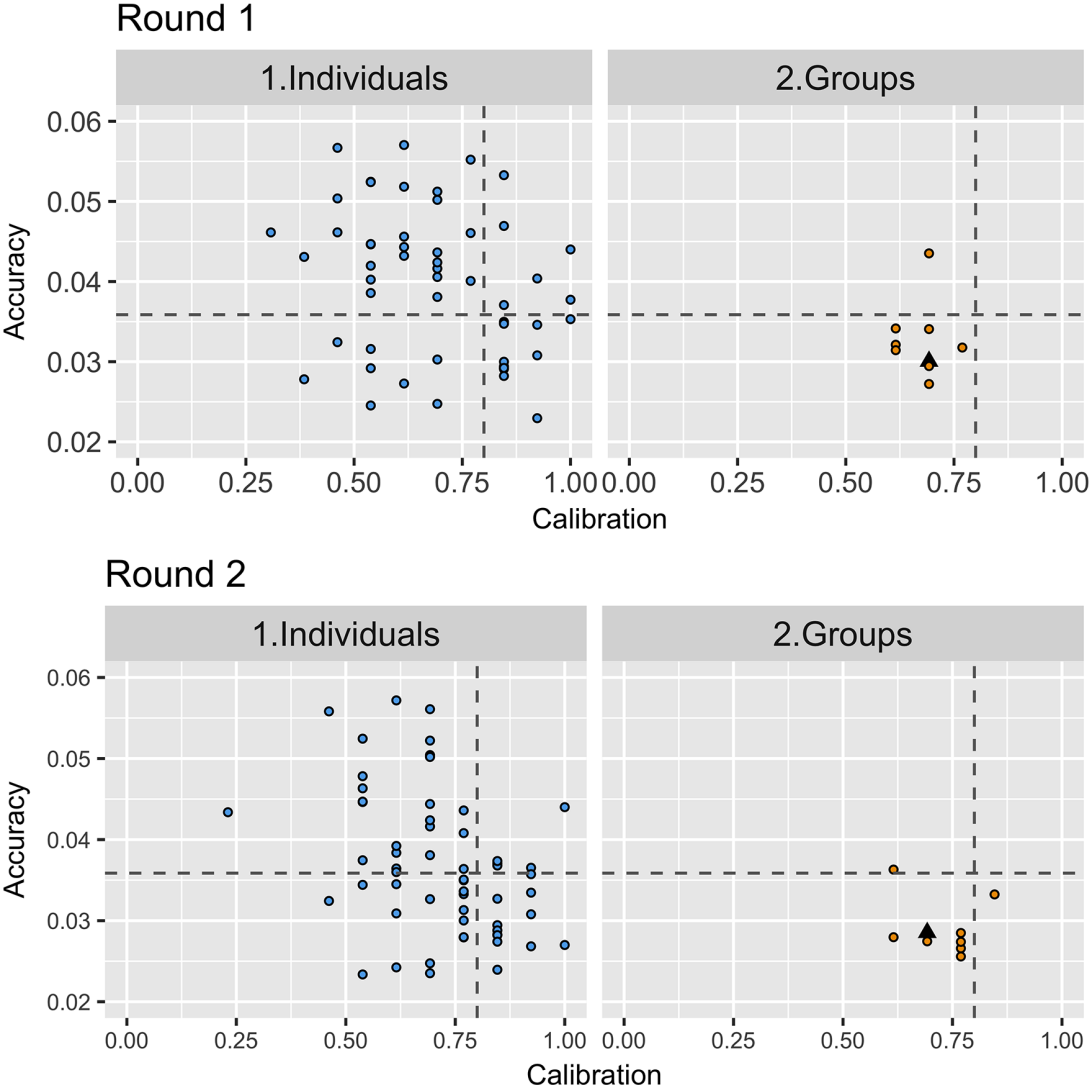
Scatterplots show the difference between groups and individuals in Round 1 and Round 2 (note only those who submitted answers in Round 1 and Round 2 were included (n = 58)). The horizontal grey line represents the median accuracy score of participants in Round 2 (lower scores are more accurate), the vertical line represents perfect calibration 0.80. Groups were on average slightly more accurate and better calibrated in Round 2 than in Round 1. The black triangle represents a super-group which is an aggregate of the estimates (arithmetic mean) of each of the 58 participants for each question before scoring the resulting estimates against the realised value.

The improvements made by individuals led to subtle and incremental improvements in the accuracy and calibration of groups (Fig 13). In total six groups improved their accuracy (lowering their ALRE score by a median of 0.003, or 4% of the total range), whilst two reduced their accuracy (increasing their ALRE score by a median of 0.001, or 1.5%). The median ALRE of groups in Round 2 was 0.027 (95%CI: 0.026–0.029), which was a slight improvement on groups in Round 1 (0.031, 95%CI:0.29–0.031), and on individuals in Round 2 (0.035, 95%CI: 0.032–0.038). Again, groups were considerably more accurate than the median individual, with six of the eight groups out-performing 75% of individuals in terms of accuracy in Round 2.

Four groups improved their median calibration, and one group reduced their calibration each by 0.077 (the equivalent to one more realisation captured / not captured). However, on average, the median calibration of groups improved from 0.692 [95%CI: 0.67–0.71]) in Round 1, to 0.77, [CI95%: 0.72, 0.81] in Round 2 (a difference of 1 additional realisation captured, and a median close to perfect calibration). Overall the median informativeness of groups remained the same in Round 2 (0.20, 95%CI: 0.17–0.20) as Round 1 (0.20 95%CI:0.18–0.22).

### Making sense of improvements in accuracy

**Fig 14:**
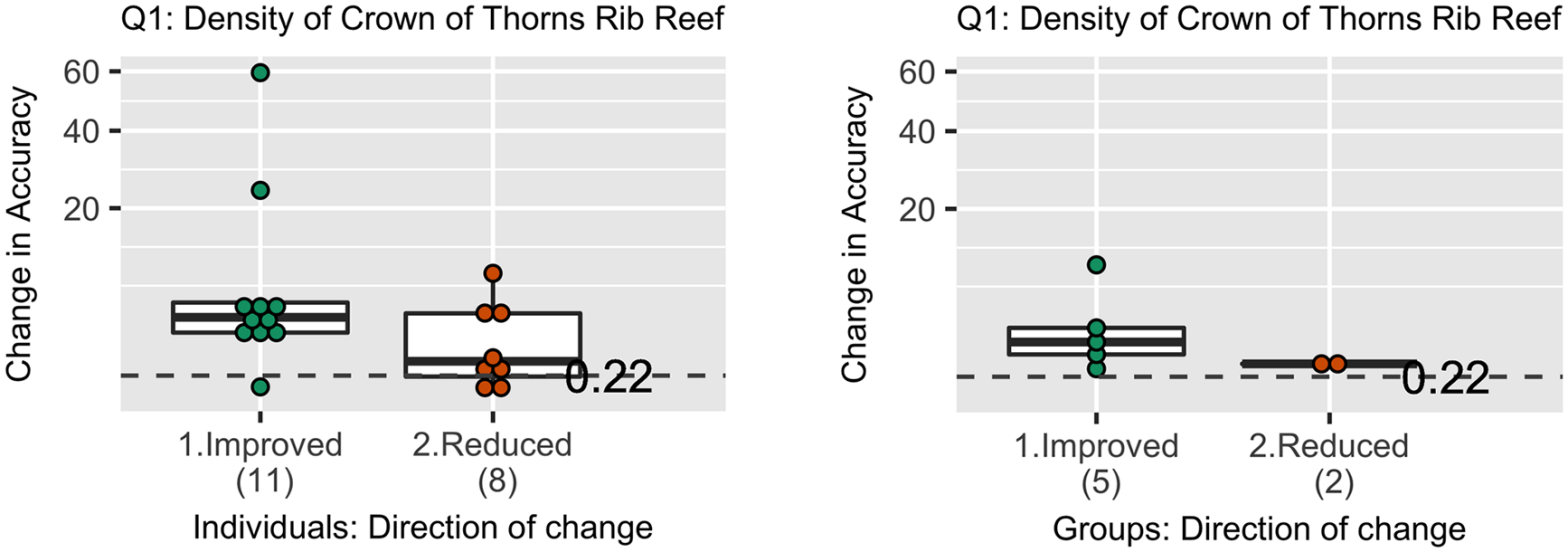
Changes in accuracy (distance from the realised truth), in Round 2, for individuals (left) and groups (right). Units for the y-axis were the density of CoTS (crown-of-thorns starfish) per 2-minute manta-tow. Note the scale is a non-linear (square root) scale. An improvement indicates revised estimates were closer to the truth than the estimates provided in Round 1. To put these numbers into perspective, minimum thresholds were developed for each question. For Question 1, the threshold was 0.22 CoTS per 2-minute manta-tow which indicates an incipient outbreak by the Australian Institute of Marine Science. Dots at or above this line indicate changes that were above this minimal threshold. The graph shows that more individuals improved their accuracy in Round 2 than those who reduced their accuracy. When changes were made they were usually above the assigned thresholds, and for some individuals their improvement in accuracy was substantial (59.90 CoTS per 2-minute manta-tow). The graph also shows that for this question more groups improved than reduced their accuracy, and the amount by which they improved was above the assigned threshold. Graphs for each of the questions can be found in S3 File.

For each question, there was a high level of variability in the amount (and direction) by which individuals changed their best estimates (Fig 14). For example, for Question 1, most participants changed their estimates by more than 0.22 (the grain size established for that question), two participants improved their accuracy substantially (by more than 20 CoTS per 2-minute manta-tow) indicating very large improvements in accuracy (usually attributed to a reduction in linguistic ambiguity). One improved their accuracy by less than 0.22 indicating an inconsequential improvement. For each question, some individuals decreased their accuracy. However, for 12 of the 13 questions the number of participants who improved their accuracy was higher than the number that reduced their accuracy. For 10 of these questions, the median improvement was above the specified grain size.

The changes made by individuals led to some changes in the accuracy of the aggregated group, although with only one or two participants in any group updating their estimates for any question, improvements were of a lesser magnitude than those of individuals. For eight out of the 13 questions, the number of groups who improved their accuracy was higher than the number of groups that reduced their accuracy, with three of these groups improving their accuracy above the specified grain-size. There was no correlation between accuracy in Round 1 and the amount by which groups and individuals changed their estimates.

**Fig 15:**
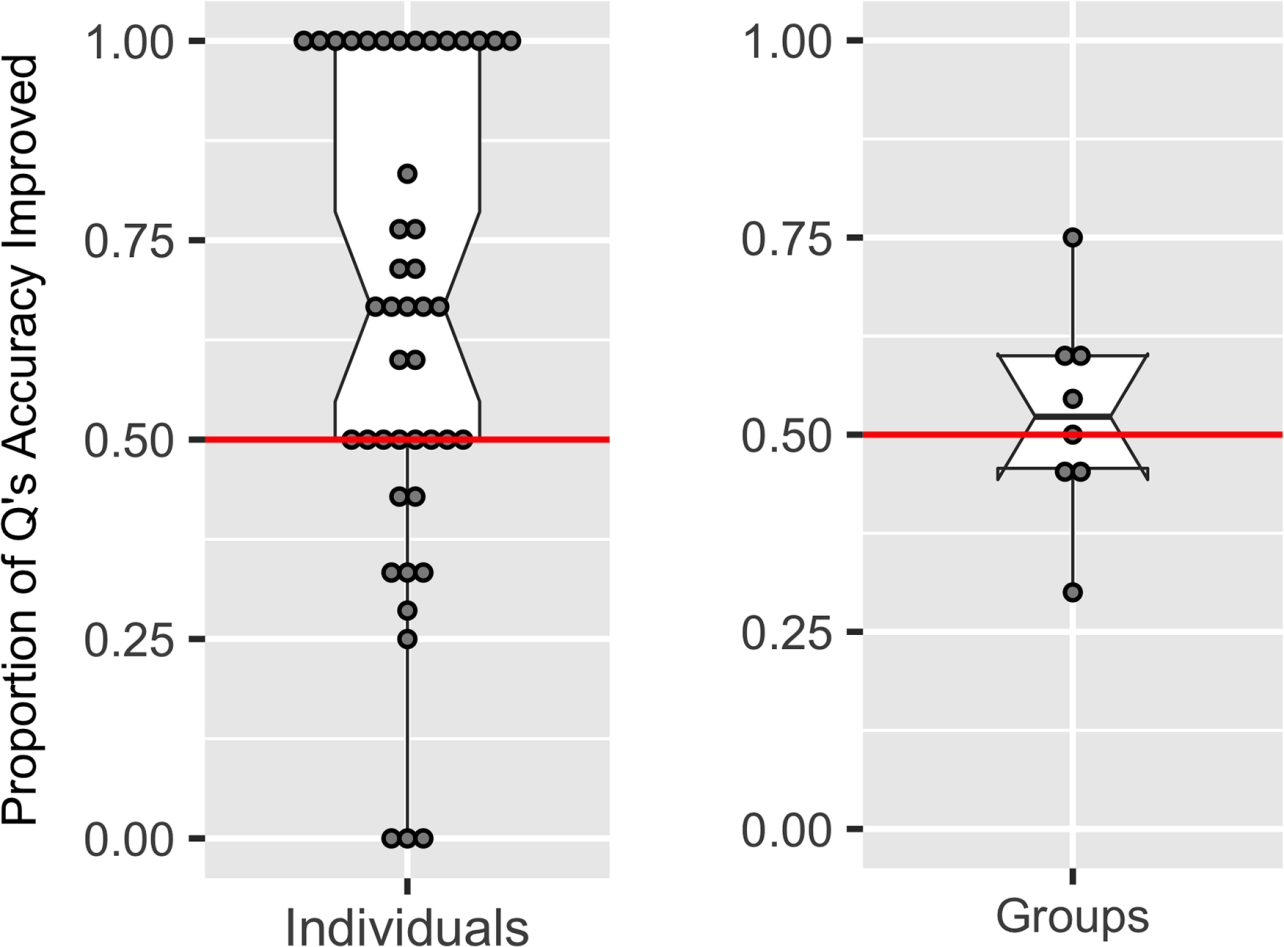
The proportion of questions where the best estimate was updated by individuals and groups for which updating improved the accuracy of the best estimate.

The results indicate that updating usually helped to improve participant and group estimates, but not consistently so Fig 15. For individuals who updated their best estimates they improved their accuracy on a median of 67% of their updates [95%CI: 55%, 79%]. For groups the effect of updating on the best estimate was less clear with improvements made on a median of 52% of updates [95%CI: 0.44%-0.60%].

### Including those who ask to withdraw

In this study, 18 participants asked to withdraw following Round 1, mostly as a result of being overcommitted. Analysts may remove their data, or use their Round 1 estimates in place of their Round 2 estimates. In the analysis above, we excluded participants who asked to withdraw from the analysis and the final (Round 2) aggregations, which reduced groups from 9–10 participants to 5–9 participants.

We explored whether the results could have been improved by including these participants in Round 2 (i.e. using their Round 1 estimates as their final estimates). We found that in Round 1 there was no difference between those participants who subsequently withdrew and those that remained (i.e. updated or reviewed their Round 1 estimates). However, in Round 2, those who updated their estimates improved their accuracy and calibration becoming more accurate and better calibrated than those who withdrew in Round 2.

**Fig 16:**
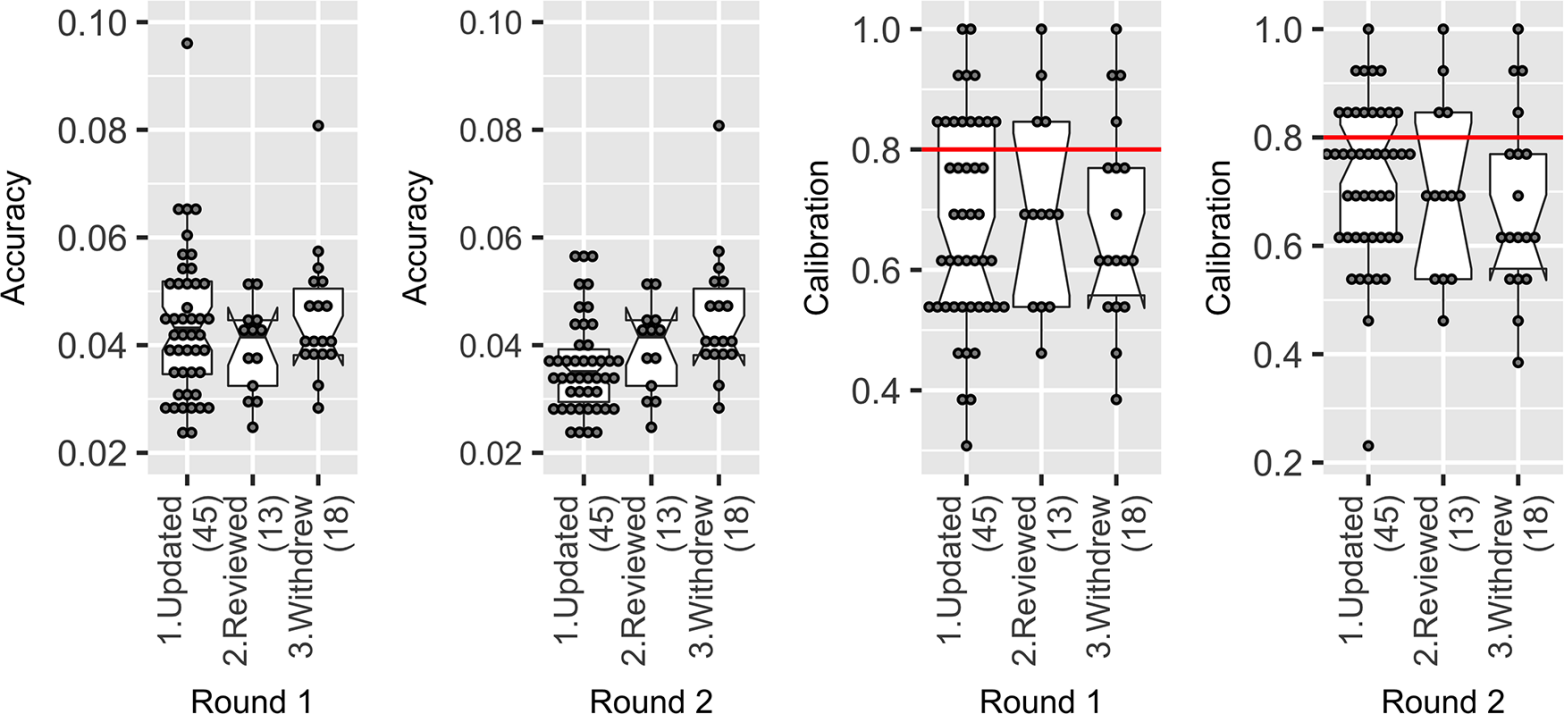
In Round 1, there was no difference in accuracy and calibration between those who withdrew and those who remained. However, those who updated their estimates in Round 2 became on average better calibrated and more accurate than those who withdrew.

Including the individuals who withdrew in Round 2 within the group aggregations resulted in worse accuracy (a difference of 0.002, or 2.5% of the range), and a reduced calibration (a difference of 0.77, or one fewer realisation captured) (Fig 16). There was no effect on informativeness. While the differences in group performance were subtle, in this study, we found there was no advantage to retaining the Round 1 estimates of these individuals in either the Round 1 or the Round 2 aggregations.

### Supersizing groups

The number of participants required to undertake a group elicitation is not precisely determined. In other studies, groups of 5–15 participants strike a sensible balance between operational constraints and performance [70, 108, 109]. In this study, we examined whether the Super Group (containing the judgements of all 58 participants) led to greater accuracy, calibration and informativeness than the eight groups (comprised 5–9 participants). Fig 16, above, shows the calibration (Round 1 and 2 = 0.69) and accuracy (Round 1 = 0.030, Round 2 = 0.029) of the Super Group in Round 1 and Round 2, against individuals and each of the eights groups (5–9 participants). As can be seen, the Super Group performed as well as the average group, but no better.

## Discussion

Expert judgement is not a substitute for the careful collection of empirical data. However, often the data required to inform critical decisions is absent or uninformative and expert judgement is unavoidable [13]. Structured protocols have been widely advocated as a means to improve the transparency, accountability and quality of expert judgements, and are used across a range of scientific domains. However, their adoption in natural resource management has been limited. We have suggested a range of possible reasons for this, including few examples demonstrating how structured protocols can be implemented within the financial and practical constraints of many natural resource problems, while leading to improvements in judgements.

We demonstrated that the IDEA protocol with remote elicitation and the four-step question format provides a practical, cost-effective and easily implementable approach to derive transparent quantitative judgements with uncertainty under the constraints of many natural resource management problems. Once questions of interest were defined, the protocol relied on nothing more than a conference call, email and PDF forms, and standard statistical analyses. The remote implementation meant that participants from across the globe could be convened to take part. While the elicitation took five weeks, the process could be expedited if funding was available to hold a two-day workshop or teleconference.

The study also confirmed that many steps in the IDEA protocol improved judgements. For example, the IDEA protocol uses a very inclusive definition of expertise, namely, anyone who has sufficient knowledge to understand the questions of interest. The reason for applying this definition is because good judgement can rarely be predicted *a-priori*, and applying additional criteria based on traditional descriptors of expertise can lead to the exclusion of potentially knowledgeable individuals [67]. Our study supports this contention, finding that the criteria used to determine whether someone should be invited to take part in an elicitation such as self-rating, affiliation, experience, and qualifications are unreliable guides to performance. Furthermore, relying on one method for the selection of experts, for example peer-recommendation, does exclude potentially knowledgeable individuals.

The one difference we found was related to gender, with men on average being more overconfident and less accurate than women, but women being less informative. This was surprising as one should not expect to see a difference in performance based on single generic demographic variable. However, the result reinforces differences between genders as a recurrent theme in the judgement and risk literature, with men on average being found to be more over-confident [65], less willing to update their estimates [110] and more risk seeking than women [47].

The result was also particularly interesting given that males were twice as likely to be recommended as experts than females. We have no data about the background distribution of genders in marine ecology, so it is not possible to say whether this difference simply reflects a gender bias in the discipline or whether there is some difference in how men and women are perceived by their peers (i.e. an unconscious bias [111]). The status-enhancement theory of overconfidence proposes that overconfidence helps people to attain higher social status. If as found in this study, males are on average overconfident, the status-enhancement theory predicts they are more likely to be perceived (and therefore recommended) as experts by their peers [112]. Whilst our study did not directly address this hypothesis, we found there was inconclusive evidence of a difference between novices and experts (if anything novices were more likely to be overconfident) which detracts from the idea of the status-enhancement theory as being a strong driver determining who people consider experts or novices.

A strong finding of this study is that judgments of randomly allocated groups outperform individuals. This finding supports the ‘wisdom of the crowd’ phenomenon [79], which specifies that the mathematical aggregation of the judgements of a group of individuals will be more accurate than the those of the average individual [113]. Our results provide yet more empirical evidence that it is better to elicit judgements from a group than from a single well-credentialed person.

These results are important, because organisations relying on expert judgment frequently seek the best, or most credentialed experts, which can lead to the reliance on a single expert [68]. However, as our study and many other studies show, these intuitions are usually based on unreliable criteria [68], and can lead to poor selection of experts and poor judgements [67, 68, 112]. Our study demonstrates, that if a relatively inclusive definition is used to source experts, then it will almost always be possible to find enough experts to form a crowd.

We also demonstrated that the benefits derived from group judgments were achieved from the elicitation of relatively few individuals (5–9 individuals), and that no further improvements derived from combining the estimates of all 58 participants who submitted estimates in both Round 1 and Round 2. These findings support those of [108], [114], and [109] who concluded that optimal group performance can be achieved with as few as 5–12 participants, with diminishing returns from the inclusion of additional individuals. We highlight that people withdrew from each of our groups (24% withdrawal rate). We recommend recruiting additional participants to anticipate such withdrawals (e.g. 10–12 participants).

The Super Group in Round 2 was combined from participants from each of the sub-groups who had interacted with one another. The results therefore may differ from what might be achieved in Round 2 had all 58 participants interacted in one forum. Regardless, the results in Round 1 were prior to interaction and do reflect that there was no clear advantage to eliciting judgements from more than 5–9 participants.

There is mixed advice in the literature as to whether interaction between experts improves or erodes the quality of expert judgements. Discussion between experts can be associated with groupthink [115, 116], social influence (diminishing the accuracy of judgements without improvement in its collective error) and overconfidence [117]. However, results by [67], [80] and [95] demonstrate that when the focus of the discussion is not to achieve consensus, but to reduce linguistic ambiguity and explore counterfactual information, as in the IDEA protocol, then the accuracy of individual judgements is improved.

In this study, we found little evidence of groupthink or social influence following discussion. Rather participants appeared to strongly anchor on their initial estimates, and were reluctant to update them, only updating on average three of their best estimates, and seven of their intervals out of the 13 questions. We found that participants did not always improve their accuracy but did so for two thirds of questions where their best estimate was updated. We found that incremental changes across questions helped to improve their calibration. We found that when participants updated their best estimates, they usually did so by a meaningful amount.

The study did not include control groups that did not see the results of the first round and did not engage in discussion. Therefore, judgements may have been improved by the feedback and discussion, or simply by asking participants to revise their estimates. However, the comments from participants suggested that the graphical feedback was particularly useful, and enabled them to clearly see where they may have misinterpreted the question, or that their views may differ from others. These findings support those by [118] and [119] that people often fail to incorporate diverse sources of evidence or consider the possibility that they may be incorrect unless encouraged to consider counterfactual evidence. The feedback process in the IDEA protocol encourages that important step.

In this study, we also explored the effect of including participants who withdrew after Round 1. The results demonstrate that there was no benefit to their inclusion. In fact, they led to slightly worse group performance. This finding supports advice provided in [87]. There are additional reasons why people who request to withdraw should always be removed from the results of an elicitation. Firstly, language-based ambiguity in questions can be pervasive, and may only be satisfactorily resolved through the process of feedback and discussion afforded in the elicitation. Secondly, estimates provided by participants must be owned by them [82, 83, 120], if participants ask to withdraw, then it is reasonable to assume they take no ownership (or accountability) over their contribution to the final results, and they should be removed.

We also note that the protocol helped to improve judgements, but it did not completely guard against misleading heuristics. We suspect that the minimal data provided led to some anchoring; a similar observation was made by [22]. In fact, the comments of some participants suggested they used these data to build a linear model and developed their estimates accordingly. In feedback surveys, some participants suggested they also sourced their own information. This may imply that in this case, participants used the anchoring heuristics relatively effectively. In other words, they used the data to inform their judgements, then looked for counterfactual or additional evidence, and in its absence, provided a judgement relatively close to previous data.

Background data are provided routinely for structured expert elicitation protocols [82, 84, 121]. However, the results of this study suggest that caution needs to be taken with the information provided to participants, and raises questions about expert judgements if the conditions they are asked to predict deviate substantially from prior data. Based on these findings, we recommend that analysts do not include background information in Round 1, so that individuals first use their own judgement. Background information can then be supplied during the discussion stage, prior to the Round 2 judgement.

There was also some evidence of the recency effect (an availability bias). This was particularly true in the case of Question 2, related to coral bleaching. Towards the end of Round 1, the first media reports in 2016 of coral bleaching on the Great Barrier Reef had begun to emerge. However, it was not until the close of the discussion phase (March 29th, 2016) that news of the bleaching events became widespread and reported in the media on a near daily basis. The media reports about the extent and degree of bleaching were conflicting, and the relevance of these reports was complicated by the fact that the reefs that were the focus of Question 2 were located in the southern part of the Great Barrier Reef which is generally cooler, while many of the reports of bleaching related to the northern sections of the reef which is generally warmer (and therefore more susceptible to bleaching). The estimates provided by participants typically increased upwards (more reefs expected to be recorded with bleaching) in relation to this question, but the intervals provided by most participants suggested there was a high degree of uncertainty around this event (Fig 7).

## Conclusion

Natural resource managers often face difficult decisions and lack empirical data to inform those decisions. While the application of models and new technologies affords increasing ways to acquire data, reliance on expert judgement appears unavoidable. Our study demonstrates that in these situations we can never guarantee that the judgements will be accurate or well-calibrated. However, through the application of structured elicitation protocols we can ensure that these judgements are as accurate and well-calibrated as possible, without incurring onerous costs. Furthermore, by applying these protocols we can apply to judgements the same requirements of review, repeatability and transparency as empirical data. The advantages of structured elicitation protocols have been identified for some time. This study demonstrates that such protocols can be applied within the financial and practical constraints of many natural resource problems, without compromising resulting judgements.

## Supporting information

S1 File Defining scoring rules. (PDF)

S2 File Elicitation documents. (PDF)

S3 File Demographic data and analysis. (PDF)
