# Supplementary material for: Eliciting improved quantitative judgements using the IDEA protocol: A case study in natural resource management
Source: PLoS One. 2018 Jun 22;13(6):e0198468. doi: 10.1371/journal.pone.0198468 (PMC6014637; doi:10.1371/journal.pone.0198468)
Supplement: S1 File — (PDF) [file pone.0198468.s001.pdf]

Appendix 3: Defining scoring rules

Quantitative judgements can be probabilities, distributions (usually continuous), point estimates, and / or interval judgements. The quality of quantitative judgements is often described by measures of 'accuracy', 'calibration' and 'informativeness', however, the meaning of these terms can differ across contexts.

'Accuracy' usually denotes the distance of the expert's best estimate (a point estimate) from the realised truth [1-3]. We use this definition in our study, but we note that accuracy has also been used in the literature to define a range of measures [4] including the proportion of correct predictions for categorical estimates [5] and rank order correlations [5, 6].

'Informativeness' is usually used to denote some measure of the width (or precision) of the intervals provided by experts [7, 8]. An expert providing narrow intervals will be considered more informative than an expert providing wide intervals. We use this definition, but again note, there are variations in the literature. For example, the Classical Model, which assesses the information of probability distributions, uses the Kullback-Liebler divergence measure [9], which is scale invariant and suited to probability distributions with defined quantiles. It takes into account the departure from an uninformative uniform, or log-uniform distribution.

'Calibration' typically relates to notions of overconfidence [10] and under-confidence. The way in which calibration is assessed differs depending on the question asked but usually includes the proportion correct answers provided by an expert relative to their assigned confidence [10, 11]. In this study, we score the calibration of interval judgements in this way. For example, for intervals assigned a confidence of 80%, we expect a well-calibrated individual to capture the realised truth in 8 out of 10 cases. If they capture fewer realisations than this, they would be considered overconfident, if they capture many more realisations they may be considered underconfident.

While we use this definition, we note variations in the literature. For example, for probabilities, if an expert states they are 80% certain an event will happen, they are considered well-calibrated if, over many predictions, the event occurs 80% of the time [12, 13]. Variants on this theme also exist for probability distributions; the proportion of realisations falling within quantiles (i.e. 5th, 50th, and 95th) can be compared to the expected proportions (i.e. less than 5% below the 5th quantile). This is the method used to calculate "calibration" [9] (also referred to as "Statistical accuracy" [14]) for the Classical Model [15].

There are notable trade-offs between calibration and informativeness when it comes interval judgements and probability distributions. By providing very wide intervals, an expert can achieve near perfect calibration, but they will be uninformative [9]. Likewise, by providing very narrow intervals, they will have a high level of informativeness, but at the risk of being overconfident [16]. Ideally the expert should be both well-calibrated and informative [9].

In this study, we ask experts to provide best estimates of an unknown fact together with an interval defined by credible lower and upper bounds that correspond to a given degree of belief [17]. We therefore define good judgement as experts who are capable of providing accurate point estimates, as well as well-calibrated and informative interval ranges (Fig 1).

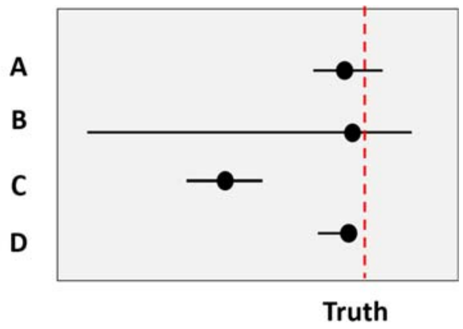

Fig 1. Accuracy, calibration and informativeness for the IDEA protocol explained. The graph shows four hypothetical experts, their best estimates (black dot), and their credible intervals. The red dashed line represents the realised truth. Expert A has a best estimate close to the truth, and their intervals capture the truth (which over many questions contributes towards their calibration), they are also informative (narrower intervals) relative to Expert B, although

Expert B is more accurate. Expert C is informative but is not accurate and does not capture the true estimate (calibration). Expert D is accurate and informative. However, their bounds do not encapsulate the truth (calibration).

References:

1. Larrick RP, Soll JB. Intuitions about combining opinions: Misappreciation of the averaging principle. Management science. 2006;52(1):111-27.
2. Galton F. Vox populi (The wisdom of crowds). Nature. 1907;75(7):450-1.
3. Einhorn HJ, Hogarth RM, Klempner E. Quality of group judgment. Psychological Bulletin. 1977;84(1):158.
4. Gigone D, Hastie R. Proper analysis of the accuracy of group judgments. Psychological Bulletin. 1997;121(1):149.
5. Kahneman D, Tversky A. On the psychology of prediction. Psychological Review. 1973;80(4):237.
6. Gordon K. Group Judgments in the Field of Lifted Weights. Journal of Experimental Psychology. 1924;7(5):398.
7. Yaniv I. Weighting and trimming: Heuristics for aggregating judgments under uncertainty. Organizational behavior and human decision processes. 1997;69(3):237-49.
8. Yaniv I, Foster DP. Graininess of judgment under uncertainty: An accuracy-informativeness trade-off. Journal of Experimental Psychology: General. 1995;124(4):424.
9. Quigley J, Colson A, Aspinall W, Cooke RM. Elicitation in the Classical Model. In: Dias LC, Morton A, Quigley J, editors. Elicitation: The Science and Art of Structuring Judgement. Cham: Springer International Publishing; 2018. p. 15-36.
10. Lin S-W, Bier VM. A study of expert overconfidence. Reliability Engineering & System Safety. 2008;93(5):711-21. doi: http://dx.doi.org/10.1016/j.ress.2007.03.014.
11. Lichtenstein S, Fischhoff B, Phillips LD. Calibration of probabilities: The state of the art. Decision making and change in human affairs: Springer; 1977. p. 275-324.
12. Tetlock P, Gardner D. Superforecasting: The art and science of prediction. New York: Random House; 2015. 340 p.
13. Clemen RT, Winkler RL. Calibrating and combining precipitation probability forecasts. Probability and Bayesian statistics: Springer; 1987. p. 97-110.
14. Cooke RM. Validation in the Classical Model. In: Dias LC, Morton A, Quigley J, editors. Elicitation: The Science and Art of Structuring Judgement. Cham: Springer International Publishing; 2018. p. 37-59.
15. Cooke RM. Experts in uncertainty: Opinion and subjective probability in science. Sharader-Frechette K, editor. New York: Oxford University Press; 1991.
16. Yaniv I, Foster DP. Precision and accuracy of judgmental estimation. Journal of behavioral decision making. 1997;10(1):21-32.
17. Soll JB, Klayman J. Overconfidence in interval estimates. Journal of Experimental Psychology: Learning, Memory, and Cognition. 2004;30(2):299.
