# Supplementary material for: Eliciting improved quantitative judgements using the IDEA protocol: A case study in natural resource management
Source: PLoS One. 2018 Jun 22;13(6):e0198468. doi: 10.1371/journal.pone.0198468 (PMC6014637; doi:10.1371/journal.pone.0198468)
Supplement: S2 File — (PDF) [file pone.0198468.s002.pdf]

PLAIN LANGUAGE STATEMENT

"The Great Barrier Reef Intelligence Games"

Researchers: Victoria Hemming, Professor Mark Burgman, Dr Terry Walshe. Dr Anca Hanea.
School of Biosciences, University of Melbourne

Project aim

The main aim of this project is to determine whether structured expert elicitation can be used to yield relatively accurate and informative judgements from i) individuals and ii) groups. The project focuses specifically on the prediction of biotic and abiotic events on the Great Barrier Reef in 2016. We have also included some questions on geo-political events to determine whether people and groups with good judgement can extend their knowledge to new domains.

Background

Accurate and informed expert judgement is critical in fields such as conservation which are characterised by high uncertainty, lack of data and pressure to make a decision.

However, the quality of expert judgement can be subject to various biases, including over-confidence and group-think. These biases can be exacerbated by poorly designed elicitation methods.

This project applies a structured approach for eliciting expert judgment to determine whether it can be used to yield better judgements from individuals and groups within a domain of conservation.

Under our overarching aim there are several questions this study seeks to answer:

- Whether the performance of a group outperforms individuals.
- Whether good expert judgement is narrowly confined to a small sub-domain of knowledge.
- Whether group performance improves following feedback.
- Whether weighting individuals in groups based on performance yields better group judgements.
- Whether expert performance is correlated with common selection criteria such as age, experience, and peer-recommendation.

What will I be asked to do?

If you agree to participate, you will be asked to partake in the tasks outlined in Table 1, the most important being answering the survey of 20 questions.

Except for the teleconference, all other tasks can be completed in your own time within the designated time-frames.

The survey will ask you 20 questions mostly related to biotic and abiotic events on the Great Barrier Reef. We expect you to take the survey seriously, however, we don't want you to spend more than 5 minutes on each question, or 2 hours in total providing initial estimates - you will be able to revise these estimates later, so please just provide your best estimates.

You will be able to use whatever publicly available information you have access to, however, you will be asked not to discuss the questions or answers with anyone. We will provide an opportunity for you to discuss the questions between your Round 1 and Round 2 estimates.

Please note three of these tasks are required for our analysis, the other two will help you to make better judgements and we hope you can find the time to participate in these non-critical tasks.

Responsible Researcher: Professor Mark Burgman, University of Melbourne, Ph: +61 3 8344 7151 Email: markab@unimelb.edu.au
Co-researcher: Victoria Hemming, University of Melbourne, Ph: +61 3 6232 5044 Email: hemmingv@student.unimelb.edu.au
Co-researcher: Dr Terry Walshe, Australian Institute of Marine Sciences, Ph: + (03) 9035 6413, Email: t.walshe@aims.gov.au
This research has been approved by the Human Ethics Committee of The University of Melbourne (HREC Project Number: 1546009.1). If you have any concerns about the conduct of this study that the researchers have not been able to answer to your satisfaction, you may contact the Executive Office, Human Research Ethics, The University of Melbourne, 03 8344 2073 (phone) or 03 9347 6739 (fax).

THE UNIVERSITY OF MELBOURNE

cebra Centre of Excellence for Biosecurity Risk Analysis

Table 1 Important steps required as part of this survey.

Important dates	Task	Time allowance	Required or recommended step
**29th February, 2016**	Complete short demographic survey	10 minutes	Required
**1st March 2016** (11am Melbourne time)	Attend a teleconference to explain the purpose of the study and the method we'd like you to use.	0.5 – 1 hour	Recommended
**1st -10th March 2016**	Round 1: Provide initial estimates in response to the questions, return survey	1-2 hours	Required
**15th – 22nd March 2016**	Check your answers, participate in an online conversation about the questions	0.5 – 1 hour	Recommended
**22nd - 25th March 2016**	Round 2: Revise estimates	0.5 hours	Required
**26th March, 2016**	Survey closes		

How will my confidentiality be protected?

We will protect your anonymity to the fullest possible extent within the limits of the law. Your signed consent form, demographic information and questionnaire with responses will be kept in locked filing cabinets and password protected spreadsheets on computers in the School of Botany, at the University of Melbourne, Parkville.

Your name will be disaggregated from your answers, with only a number assigned so that we can examine common trends in age, experience, and culture in relation to performance.

The results will be published in a peer-review journal. Your name will not be listed next to your answers. We will seek your permission to include your name in the acknowledgements section. You can withdraw consent at any time prior to publication, in which case you will not be listed in the acknowledgements, and you are free to do so without prejudice.

Your responses and any notes from the workshop will be kept for five years after publication, after which time they will be destroyed.

Will participation prejudice me in any way?

Your participation in this study is completely voluntary. Should you wish to withdraw at any stage, or to withdraw any comments that you have supplied, you are free to do so without prejudice.

Where can I get further information?

This research has been approved by the Human Ethics Committee of The University of Melbourne (HREC Project Number: 1546009.1). Should you require any further information, or have any concerns, please do not hesitate to contact Victoria Hemming, Dr Terry Walshe, or Professor Mark Burgman (contact details below), or If you have any concerns about the conduct of this study that the researchers have not been able to answer to your satisfaction, you may contact the Executive Office, Human Research Ethics, The University of Melbourne, 03 8344 2073 (phone) or 03 9347 6739 (fax).

How do I agree to participate?

If you would like to participate, please indicate that you have read and understood this information by signing the accompanying Consent Form and returning it to one of the listed researchers.

Responsible Researcher: Professor Mark Burgman, University of Melbourne, contact details omitted

Co-researcher: Victoria Hemming, University of Melbourne, contact details omitted

Co-researcher: Dr Terry Walshe, Australian Institute of Marine Sciences,contact details omitted

This research has been approved by the Human Ethics Committee of The University of Melbourne (HREC Project Number: 1546009.1). If you have any concerns about the conduct of this study that the researchers have not been able to answer to your satisfaction, you may contact the Executive Office, Human Research Ethics, The University of Melbourne, contact details omitted

PLAIN LANGUAGE STATEMENT

"The Great Barrier Reef Intelligence Game, 2016"

Victoria Hemming, Mark Burgman, Terry Walshe, Anca Hanea.

School of Biosciences, University of Melbourne

Participant Name: *(please enter your full name into text box)* **>**

Email Address: *(please enter your email address into text box)* **>**

1. I consent to participate in this project, the details of which have been explained to me, and I have been provided with a written Plain Language Statement to keep.

2. I understand that my participation will attendance at a short tele-conference, the completion of a survey of approximately 25 questions relating to the Great Barrier Reef and Crown of Thorns Starfish, and an opportunity to discuss and revise my estimates. My participation will also involve the completion of a short demographic survey. I agree that the researchers may use the results as described in the Plain Language Statement.

3. I acknowledge that:
  - a) the project is for the purpose of research;
  - b) I have been informed that my involvement in the project is voluntary and that I am free to withdraw at any time, and am free to withdraw any unprocessed identifiable data previously supplied;
  - c) I will only be asked to provide my judgement for quantities relating to data that will be collected for the Great Barrier Reef and some geo-political events;
  - d) I have been informed that the basic demographic information will be used to make general comparisons between participants;
  - e) I am aware that I will remain anonymous, in this research, and my responses will only be attributed to my demographic information;
  - f) I am aware that I may be asked to provide a brief description of my expertise relating to survey in a demographic survey;
  - g) I have been informed that this signed consent form and any comments/data I provide will only be accessed by the named researchers, kept in a secure place, and will be destroyed five years after publication;
  - h) I have been informed that the confidentiality of the information I provide will be safeguarded subject to any legal requirements;
  - I) Once I AGREE to take part and return this consent form, it will be retained by the listed researchers.

I have read and acknowledged the above and I AGREE to take part in this survey

(To confirm please select from drop down list) **>** No

Optional: I consent to my name being listed in the acknowledgements section of any publications resulting from the research (Please select) > No

Click 'next' to proceed to demographic survey and submit >> **Next >**

Responsible Researcher: Prof Mark Burgman, University of Melbourne, contact details omitted **Co-researcher:** Victoria Hemming, University of Melbourne, contact details omitted **Co-researcher:** Dr Terry Walshe, Australian Institute of Marine Sciences, contact details omitted Email: t.walshe@aims.gov.au This research has been approved by the Human Ethics Committee of The University of Melbourne (HREC Project Number: 1546009.1). If you have any concerns about the conduct of this study that the researchers have not been able to answer to your satisfaction, you may contact the Executive Office, Human Research Ethics, The University of Melbourne, contact details omitted

DEMOGRAPHIC INFORMATION
"The Great Barrier Reef Intelligence Game, 2016"
Victoria Hemming, Mark Burgman, Terry Walshe. Anca Hanea.
School of Biosciences, University of Melbourne

We are collecting some basic information to help profile your particular area of expertise. This will not be used to identify you.

1. Please create a unique code name (please aim for 4-6 digits).
   Note: The name you assign yourself will be visible to other participants. *(Please enter)* >
2. Salutation? *(Please select from drop-down list)* >
3. Gender? *(Please enter)* >
4. Age? *(Please select from drop-down list)* >
5. What is the highest level of education you have successfully completed? *(Please enter)* >

   If other, please specify >
6. Current occupation title?
   (e.g. fisherman, botanist, accountant, PhD Candidate)
7. Current employment sector?

   If other, please specify >

The following questions aim to understand your professional experience relevant to the survey questions.

8. This question relates to Table 1 below:
   In column A), please indicate how many years of professional or postgraduate experience you have for each of the topics (e.g. 2.5 years). If you have no relevant experience, please enter '0'.
   In column B) rate your knowledge between 0 and 10.
   0 - No prior knowledge or understanding.
   1- Basic understanding, (e.g. I have read reports, news articles, but no working / study experience).
   5- Intermediate understanding (e.g. you have relevant work or postgraduate research experience).
   10- Specialist understanding (e.g. you regularly collect data, prepare or sign off on reports, give advice to the public and clients on this topic)

Table 1: Topics	A (Years Exp.)	B (self-rating) 1-10
Crown of Thorns Starfish (*Acanthaster planci*)		
Coral reef ecology		
Marine bio-invasions (other than Crown of Thorns)		
Water quality		
Commercial fisheries		
Climate, rainfall, weather forecasting.		

9. Have you published technical or peer-reviewed reports on the topics listed in Table 1, Question 8?
If yes please provide an approximate number of:
a. Peer-reviewed journal articles
b. Technical reports

10. Are you a member of a committee or advisory panel relevant to the topics listed in Table 1 , Question 8?

11. Have you provided your professional advice to a client or a member of the public about any of the topics listed in Table 1, Question 8?

The following set of questions aim to determine your familiarity with the Great Barrier Reef (GBR)

13. Have you ever been snorkelling / diving on the GBR?

14. Have you undertaken volunteer work or undergraduate study on the GBR?

15. Do you have professional or post-graduate experience related to the GBR?

16. Have ever been involved in data collection for the AIMS Long Term Monitoring Program?

17. Have you ever seen a Crown of Thorns Starfish (*Acanthaster planci*)?

Can you please recommend some people to be involved in the study?

Can you recommend another 3 people who you would consider to be **experts / or highly experienced** in the topics listed in Question 8?

18.

Name	Surname	Email	Organisation

19. Can you recommend another 3 people who **may not be considered experts** but have at least some basic knowledge of the topics listed in Question 8?

Name	Surname	Email	Organisation

Thank you!

Please remember to **save** this form and **return to**
hemmingv@student.unimelb.edu.au

Instructions and Frequently Asked Questions

"The Great Barrier Reef Intelligence Game"

searchers: Victoria Hemming, Professor Mark Burgman, Dr Terry Walshe. Dr Anca Hanea.
School of Biosciences, University of Melbourne

Welcome to the start of the Great Barrier Reef Intelligence Game!

We are hoping this will be a bit of fun, but also with any study we are hoping to make some useful insights. The aims of this study are outlined in the Plain Language Statement. Any findings we make from this experiment will be shared with you, so that you can use them in your own line of work.

1. Some important rules:

A Golden Rule

Please do not discuss the questions with people involved in the Intelligence Game. This is particularly important as we are specifically testing for individual judgement in Round 1, and then for the effect of controlled group discussion in Round 2. You will have an opportunity to discuss your judgements with an allocated group in Round 2.

Important dates

Important dates	Task	Time allowance	Required or recommended step
**1st -10th March 2016**	**Round 1:** Provide initial estimates in response to the questions, return survey	1-2 hours	Required
**15th – 22nd March 2016**	Check your answers, participate in an online conversation about the questions	0.5 – 1 hour	Recommended
**22nd - 25th March 2016**	**Round 2:** Revise estimates	0.5 hours	Required
**26th March, 2016**	Survey closes		

How much time to spend on each question?

There is no time limit, however, as guideline we ask you to manage your time so that you are able to answer all 21 questions in 1.5 – 2 hours, this equates to approximately 5-10 minutes per question.

Where previous monitoring data is available we have provided this to you. The data has been included to reduce the demand on your time. You are welcome to use it or dismiss it.

What resources can I use to answer the question?

Not permitted: Please do not discuss the questions with people involved in the Intelligence Game.

Responsible Researcher: Professor Mark Burgman, University of Melbourne, contact details omitted
Co-researcher: Victoria Hemming, University of Melbourne, contact details omitted
Co-researcher: Dr Terry Walshe, Australian Institute of Marine Sciences, contact details omitted

This research has been approved by the Human Ethics Committee of The University of Melbourne (HREC Project Number: 1546009.1). If you have any concerns about the conduct of this study that the researchers have not been able to answer to your satisfaction, you may contact the Executive Office, Human Research Ethics, The University of Melbourne,contact details omitted

Permitted: You are welcome to use any source available to you (people outside of the intelligence game, internet, references, books, news articles). If you do have additional information to share with your group between Rounds 1 and 2 please make a note of it in the space provided next to each question.

2. Answering the questions.

Using the spreadsheet

You require EXCEL 2010 or above

You have been provided with 21 questions in an EXCEL spreadsheet. This has been formatted for EXCEL 2010 and above. If you have an older version of EXCEL please try opening and saving the document, it should still work but may be formatted slightly different. If you have any problems, contact Victoria and she will send you an alternative version. If you would prefer to work from a PDF, this can also be provided.

Why we formatted the survey in EXCEL

We have chosen to distribute the survey using EXCEL because it is a program which most people have access to, and can be accessed offline and online. It also allows us to incorporate graphs to provide you instant feedback of your intervals, as well as how we will standardise your intervals for Round 2. Again, if you have any problems, contact Victoria and she will send you an alternative version. If you would prefer to work from a PDF, this can also be provided.

Navigating between questions

Please use the tabs at the bottom of the page to navigate between questions, keeping track of questions you have answered.

How do I answer the question?

Each question has four parts we ask you to answer (please note the inclusion of comments and links is optional). Please pay attention to the order and particular wording of the questions (it was developed by some psychologists, they've told us the order and wording is important… so we'll aim not to mess with it for now).

1. Realistically what do you think the lowest plausible value for X will be?
2. Realistically what do you think the highest plausible value for X will be?
3. Realistically what is your best guess for X?
4. How confident are you that your interval, from lowest to highest, could capture X? Please enter a number between 50 and 100%.

searchers: Victoria Hemming, Professor Mark Burgman, Dr Terry Walshe. Dr Anca Hanea.
School of Biosciences, University of Melbourne

These four estimates are then combined into an interval judgement. You will be able to see your interval judgements next to each question, and will be able to see how they match other participants' judgements in Round 2 (Figure 1).

We recently used this method and found that a group of botanists from Melbourne beat the US Intelligence Agency at their own game on geopolitical events.

Think about your intervals.

People commonly get confused about what their confidence level means:

- Your stated confidence reflects how sure you are that the truth is contained between your lower and upper estimates.
- Your confidence should fall between 50% and 100% for the following reasons:
  - If you state your confidence is less than 50% it means you are more certain that the truth is located outside of your interval than it being captured by your interval. In this case it would be sensible to make your lower and upper estimates wider and increase your level of confidence that you have captured the truth.
  - If you state your confidence is 100% it means you are absolutely certain that the truth could not fall outside your upper and lower estimates. It means you should be willing to bet your house or job on it because there is no plausible way the truth could be located outside your interval.

3. Other Frequently Asked Questions

How will I be scored?

You will be scored on three things:

- Firstly, that the truth is contained in your lower and upper interval. So think carefully about your level of confidence.
- Secondly, how informative your intervals are. This is a trade-off with the point above. If you have a narrow interval capturing the truth you will score better than a wide interval that captures the truth.
- Thirdly how close your best guess is to the truth.

In Figure 1 below, expert A is both accurate and informative- they have a narrow interval that captures the truth. They also happen to have a best guess close to the truth. Expert B captures the truth, however, their wide interval is not very informative. This wide interval communicates to us that the person is highly uncertain. This could be due to their lack of understanding of the system, or they could understand the system really well and be conveying that there is a high degree of natural variation that makes them less confident. Expert C is someone we'd like you to try and avoid being. Expert C is very confident that the truth lies in a very narrow range, however, they have failed to capture the truth. If we were to use the advice of Expert C we could run into some serious trouble.

Instructions and Frequently Asked Questions

"The Great Barrier Reef Intelligence Game"

searchers: Victoria Hemming, Professor Mark Burgman, Dr Terry Walshe. Dr Anca Hanea.
School of Biosciences, University of Melbourne

Why have you chosen to ask me about abiotic events, biotic events and geo-political events.

There are a few reasons why we have chosen to ask you about abiotic, biotic and geo-political events. Firstly we want to see whether or not good expert judgement of individuals and groups is sensitive to subject matter. Secondly, we are keen to determine whether weighted combinations for groups can yield even better group judgements and for this we need to determine how closely questions used to derive weights need to be linked to a question of interest.

What makes someone an expert?

For this study we believe that if you can read and understand the question you have sufficient experience to help us answer the question.

Good expert performance is about:

- Having a holistic understanding of the subject matter
- Always seeking the truth
- Knowing the limitations of your knowledge
- Producing success when practicing your expertise

We want you to have a go at every question, but we want you to use our question format to be able to convey your relative uncertainty about your estimates.

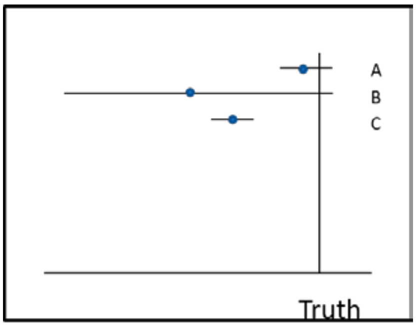

Figure 1 An example of how your judgements will be displayed against the truth (when it becomes known) and in comparison to other participants. Your intervals should always aim to capture the truth.

Responsible Researcher: Professor Mark Burgman, University of Melbourne, contact details omitted

Co-researcher: Victoria Hemming, University of Melbourne, contact details omitted

Co-researcher: Dr Terry Walshe, Australian Institute of Marine Sciences, contact details omitted

This research has been approved by the Human Ethics Committee of The University of Melbourne (HREC Project Number: 1546009.1). If you have any concerns about the conduct of this study that the researchers have not been able to answer to your satisfaction, you may contact the Executive Office, Human Research Ethics, contact details omitted

Instructions and Frequently Asked Questions

"The Great Barrier Reef Intelligence Game"
searchers: Victoria Hemming, Professor Mark Burgman, Dr Terry Walshe. Dr Anca Hanea.
School of Biosciences, University of Melbourne

What if I don't understand the question?

We acknowledge that some people may not have a good understanding of the question, but we would like to compare how well you do at these questions against those that regularly work in this space. Each question contains substantial detail in an attempt to minimise language-based ambiguity. Do yourbest to understand the question; remember you can look up additional information if you feel it will help. Also remember you can communicate your uncertainty by increasing the interval between your lower and upper bounds. You also have an opportunity to revise your estimates in Round 2.

The questions are impossible!

We have tried to make the questions as clear as possible by only asking about one data point or time period, for events occurring in the next three months. However, there is always variability, particularly in natural systems. This is why we ask you to communicate your uncertainty to us by communicating a realistic upper and lower bound that would capture this uncertainty. We then ask you to think about what the most likely outcome will be and communicate this to us as your best guess.

Can I ask questions about the questions?

Feel free to email Victoria Hemming if you have any queries about the wording of the question.

I have comments and suggestions to make about this study.

Please raise any issues with Victoria or the listed researchers. We will also send a feedback form to you following the workshop to gather your thoughts and insults.

"The Great Barrier Reef Intelligence Game, 2016"
Victoria Hemming, Mark Burgman, Terry Walshe. Anca Hanea.
School of Biosciences, University of Melbourne

Welcome! And Thank You!

Welcome to the start of the Great Barrier Reef Intelligence Game!

We are hoping this will be a bit of fun, but also with any study we are hoping to make some useful insights. The aims of this study are outlined in the Plain Language Statement. Any findings we make from this experiment will be shared with you, so that you can use them in your own line of work.

**Code Name**	*Please enter your code name* >	

i. How many years of professional or postgraduate experience do you have analysing and predicting geo-political events? If you have no relevant experience, please enter '0'.

ii. Please rate your knowledge between 0 and 10 of geo-political events, 0= no understanding / experience, 1= occasionally observe geopolitical events in the news, , 5= Some work or postgraduate research experience 10= Regularly prepare reports and advice on this topic.

Some importants rules

Please do not discuss the questions with people involved in the Intelligence Game. This is particularly important as we are specifically testing for individual judgement in Round 1, and then for the effect of controlled group discussion in Round 2. You will have an opportunity to discuss your judgements with an allocated group in Round 2.

How much time to spend on each question?

There is no time limit, however, as guideline we ask you to manage your time so that you are able to answer all 21 questions in 1.5 – 2 hours, this equates to approximately 5-10 minutes per question.
Where previous monitoring data is available we have provided this to you. The data has been included to reduce the demand on your time. You are welcome to use it or dismiss it.

What resources can I use to answer the question?

Not permitted : Please do not discuss the questions with people involved in the Intelligence Game.

Permitted: You are welcome to use any source available to you (people outside of the intelligence game, internet, references, books, news articles). If you do have additional information to share with your group between Rounds 1 and 2 please make a note of it in the space provided next to each question.

Important Timelines:

You have until 17:00 on 10 March, 2016 to submit your round 1 estimates.
Round 2 will commence on 15 March, 2016 at 10:00 (UTC).
Your final estimates are due 17:00 25 March, 2016.

Table of Contents

Instructions **3**

Questions **6**

1 *Density of Crown of Thorns Starfish* (Acanthaster planci) 7
2 *Coral Bleaching across the Great Barrier Reef* 9
3 *Asian Green Mussel Detections in Queensland* 11
4 *Prevalence of White Syndrome Coral Disease on Reef 21060* 13
5 *Commercial Catch of Coral Trout* 15
6 *Marine Turtles* 17
7 *Shark Control in the Mackay Region* 19
8 *Water Temperature in the Southern Great Barrier Reef* 21
9 *Discharge volume (Mega litres) from the Burdekin River* 23
10 *Chlorophyll Levels Detected at Pine Island* 25
11 *Wind Speed at Davies Reef* 27
12 *Average Maximum Air Temperature Hamilton Island* 29
13 *Turbidity in the Wet Tropics* 31
14 *El Nino Events* 33
15 *The Spread of Zika Virus Throughout the European Union* 35
16 *The Price of Gold* 37
17 *The UK Referendum* 39
18 *The Stock Price of Twitter* 41
19 *The Throughput (tonnes) from Abbot Point Port.* 43
20 *People Held in Nauru Regional Processing Centre* 45
21 *Launches to Space in May 2016* 47

Appendix A **49**

Ethics **50**

Instructions

How to answer the questions?

Each question has four parts we ask you to answer (please note the inclusion of comments and links is optional). Please pay attention to the order and particular wording of the questions (it was developed by some psychologists, they've told us the order and wording is important… so we'll aim not to mess with it for now).

1. Realistically what do you think the lowest plausible value for X will be?
2. Realistically what do you think the highest plausible value for X will be?
3. Realistically what is your best guess for X?
4. How confident are you that your interval, from lowest to highest, could capture X? Please enter a number between 50 and 100%.

These four estimates are then combined into an interval judgement. In Round 2, you will be able to see how they match other participants judgements (similar to Figure 1). You have also been provided with an EXCEL spreadsheet. If you are interested to see how your estimate for any question will appear in Round 2 you can have a go at entering your estimates for any of the questions and observing the graph.

We recently used this method and found that a group of botanists from Melbourne beat the US Intelligence Agency at their own game on geopolitical events.

Think about your intervals.

People commonly get confused about what their confidence level means:

- Your stated confidence reflects how sure you are that the truth is contained between your lower and upper estimates.
- Your confidence should fall between 50% and 100% for the following reasons:
    - If you state your confidence is less than 50% it means you are more certain that the truth is located outside of your interval than it being captured by your interval. In this case it would be sensible to make your lower and upper estimates wider and increase your level of confidence that you have captured the truth.
    - If you state your confidence is 100% it means you are absolutely certain that the truth could not fall outside your upper and lower estimates. It means you should be willing to bet your house or job on it because there is no plausible way the truth could be located outside your confidence intervals.

Additional Questions and Comments

How will I be scored?

You will be scored on three things:

- Firstly, that the truth is contained in your lower and upper interval. So think carefully about your level of confidence.
- Secondly, how informative your intervals are. This is a trade-off with the point above. If you have a narrow interval capturing the truth you will score better than a wide interval that captures the truth.
- Thirdly how close your best guess is to the truth.

In Figure 1 (below), Expert A is both accurate and informative- they have a narrow interval that captures the truth. They also happen to have a best guess close to the truth. Expert B captures the truth, however, their wide interval is not very informative. This wide interval communicates to us that the person is highly uncertain. This could be due to their lack of understanding of the system, or they could understand the system really well and be conveying that there is a high degree of natural variation that makes them less confident. Expert C is someone we'd like you to try and avoid being. Expert C is very confident that the truth lies in a very narrow range, however, they have failed to capture the truth. If we were to use the advice of Expert C we could run into some serious trouble.

What makes someone an expert?

For this study we believe that if you can read and understand the question you have sufficient experience to help us answer the question.
Good expert performance is about:

- Having a holistic understanding of the subject matter
- Always seeking the truth
- Knowing the limitations of your knowledge
- Producing success when practicing your expertise

We want you to have a go at every question, but we want you to use our question format to be able to convey your relative uncertainty about your estimates.

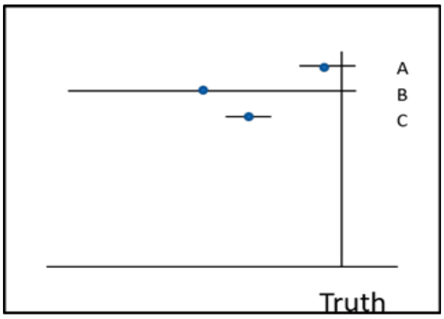

Figure 1 An example of how your judgements will be displayed against the truth (when it becomes known) and in comparison to other participants. Your intervals should always aim to capture the truth.

What if I don't understand the question?

We acknowledge that some people may not have a good understanding of the question, but we would like to compare how well you do at these questions against those that regularly work in this space. Each question contains substantial detail in an attempt to minimise language-based ambiguity. Do your best to understand the question; remember you can look up additional information if you feel it will help. Also remember you can communicate your uncertainty by increasing the interval between your lower and upper bounds. You also have an opportunity to revise your estimates in Round 2.

The questions are impossible!

We have tried to make the questions as clear as possible by only asking about one data point or time period, for events occurring in the next three months. However, there is always variability, particularly in natural systems. This is why we ask you to communicate your uncertainty to us by communicating a realistic upper and lower bound that would capture this uncertainty. We then ask you to think about what the most likely outcome will be and communicate this to us as your best guess.

Can I ask questions about the questions?

Feel free to email Victoria Hemming if you have any queries about the wording of the question.

I have comments and suggestions to make about this study.

Please raise any issues with Victoria or the listed researchers. We will also send a feedback form to you following the workshop to gather your thoughts and insults.

Ready to start?

Please save this document to your computer.

Then proceed to the questions on the next page!

Save this file

Question 1 Density of Crown of Thorns Starfish (*Acanthaster planci*)

"What will be the average density of Crown of Thorns Starfish (Acanthaster planci) *detected per 2 minute manta-tow at Rib Reef, in the Townsville region, as surveyed by the Australian Institute of Marine Science (AIMS) as part of the Long-term Monitoring Program between 1 March, 2016 and 30 June, 2016 (inclusive)?"*

Clarification: Crown of Thorns Starfish *(Acanthaster planci) (* CoTS) are found at numerous coral reef ecosystems, including the Great Barrier Reef. They consume hard corals, and are the focus of manta-tow surveys undertaken by AIMS as part of the Long Term Monitoring Program (LTMP).

This question relates specifically to the density of CoTS per two minute manta-tow that will be detected by AIMS during surveys at Rib Reef between 1 March 2016 and 30 June 2016 (inclusive). Rib Reef is located in the Townsville region of the Great Barrier Reef (GBR) (Appendix A). The average density per 2 minute manta tow, is a standard metric used to compare between reefs and years. The average density of CoTS per 2 minute manta tow refers to the total number of CoTS that are detected by AIMS during manta-tow surveys, divided by the total number of manta-tow surveys undertaken at Rib Reef. We will accept survey results for Rib Reef recorded between 1 March, 2016 and 30 June 2016 (inclusive). If the survey does not occur, or occurs outside of this period, the question will be void. As with all monitoring data, it is important to note that this question relates specifically to the number of CoTS detected and reported, not necessarily the actual number of CoTS present at Rib Reef.

Resolution: The question will be resolved when the report for the Townsville section for the 2015/2016 monitoring period is published online by AIMS (see latest surveys in useful links).

Additional Information:

a. Historical density of CoTS per 2 minute manta tow at Rib Reef recorded by the AIMS LTMP

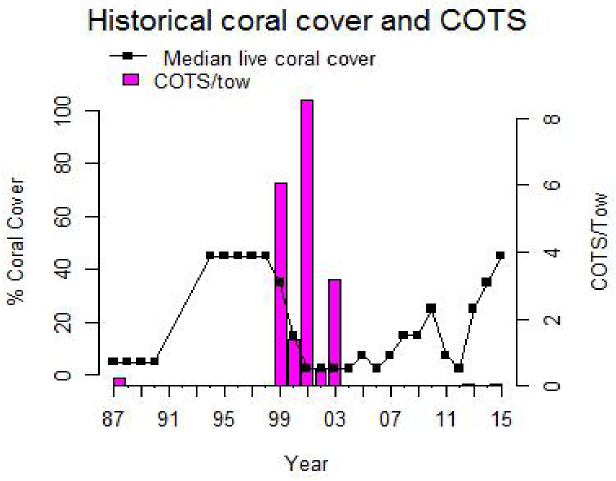

Useful Links:

i. Rib Reef	http://data.aims.gov.au/reefpage2/reefpage.jsp?fullReefID=18032S
ii. Latest surveys	http://www.aims.gov.au/docs/research/monitoring/reef/latest-surveys.html
iii. Survey methods	http://www.aims.gov.au/documents/30301/20e3bf4f-4b3b-4808-ac02-c15c2912c3f2
iv. Map of LTMP regions	Appendix A

THE UNIVERSITY OF MELBOURNE

“The Great Barrier Reef Intelligence Game, 2016”
Victoria Hemming, Mark Burgman, Terry Walshe. Anca Hanea.
School of Biosciences, University of Melbourne

cebra
Centre of Excellence for Biosecurity Risk Analysis

1. Your estimate for Question 1

		Please enter your estimate (2 decimal places)
i	Realistically, what do you think the **lowest** plausible value for the reported average density of CoTS, per 2 minute manta-tow, at Rib Reef will be?	1
ii	Realistically, what do you think the **highest** plausible value for the reported average density of CoTS, per 2 minute manta-tow, at Rib Reef will be?	2
iii	Realistically, what is your **best guess** for the reported average density of CoTS, per 2 minute manta-tow, at Rib Reef ?	3
iv	**How confident are you** that your interval, from lowest to highest, could capture the reported density of CoTs at Rib Reef? Please enter a number between 50 and 100%	%

2. Comments

Please enter any comments, additional knowledge or justification that you have about this question and /or your estimate. This will be shared with the group in Round 2.

3. Useful Links

Please enter any useful web links (URLs) you would like to contribute to your group's estimate

1		4	
2		5	
3		6	

Question 2 Coral Bleaching across the Great Barrier Reef

"How many of the 24 reefs listed in Table 1 below will be reported with at least 1% bleaching of hard corals by the Australian Institute of Marine Science (AIMS) during SCUBA surveys undertaken between 1 March, 2016 and 30 June 2016 as part of the Long Term Monitoring Program (LTMP)?"

Clarification: This question asks how many of the 24 reefs listed in Table 1 you believe will realistically be recorded as having at least 1% coral bleaching when surveyed by SCUBA surveys between 1 March 2016 and 30 June 2016 (inclusive) by AIMS as part of the LTMP. The 24 reefs listed in Table 1 are located in two regions of the Great Barrier Reef: the Townsville Region, and the Mackay / Pompey region (Appendix A). For each SCUBA search the percentage of hard coral cover which is bleached white, or near white or has a lurid appearance is recorded according to the categories in Table 2. Only reefs listed in Table 1 which are surveyed between 1 March 2016 and 30 June 2016 (inclusive) will be included in the calculation. If none of the 24 reefs are surveyed then the question will be voided.

Resolution: The question will be resolved when by the AIMS LTMP program by 1 September, 2016.

Additional Information:

a. Table 1: List of reefs scheduled for survey between 1 June 2016 and 30 June 2016

REEF_ID	REEF_NAME	RAP_REGION	REEF_ID	REEF_NAME	RAP_REGION
21060	21060S	Mackay / Pompey	18030	KELSO REEF	Townsville
20351	POMPEY REEF (NO 1)	Mackay / Pompey	18042	ROXBURGH REEF	Townsville
21591	21591S	Mackay / Pompey	18076	HELIX REEF	Townsville
20351	POMPEY REEF (NO 2)	Mackay / Pompey	18032	RIB REEF	Townsville
20348	20348S	Mackay / Pompey	18043	FORE AND AFT REEF	Townsville
21062	21062S	Mackay / Pompey	18031	LITTLE KELSO REEF	Townsville
20353	20353S	Mackay / Pompey	18083	FORK REEF	Townsville
21064	21064S	Mackay / Pompey	18077	GRUB REEF(18077)	Townsville
21139	21139S	Mackay / Pompey	18086	CHICKEN REEF	Townsville
21187	21187S	Mackay / Pompey	18081	KNIFE REEF	Townsville
21025	PENRITH REEF	Mackay / Pompey	18088	CENTIPEDE REEF	Townsville
20309	TERN REEF(20309)	Mackay / Pompey	18091	LYNCHS REEF	Townsville

b. Coral bleaching categories : sources AIMS survey procedures

% COVER OF BLEACHING FOR HARD CORALS	
0%	10-30%
0+ Individual colonies (>1% total hard coral cover)	30-50%
1-5%	50-75%
5-10%	75-100%

Useful Links:

i. Coral reef bleaching — http://www.gbrmpa.gov.au/managing-the-reef/threats-to-the-reef/climate-change/what-does-this-mean-for-species/corals/what-is-coral-bleaching

ii. AIMS Survey procedure — http://www.aims.gov.au/documents/30301/20e3bf4f-4b3b-4808-ac02-c15c2912c3f2

iii. Reefs under survey — http://data.aims.gov.au/reefpage2/allreefs.jsp

iv. LTMP map — Appendix A

1. Your estimate for Question 2

		Please enter your estimate (whole number)
i	Realistically, what do you think the **lowest** plausible value for the number of reefs reported with at least 1% bleaching of hard coral will be?	
ii	Realistically, what do you think the **highest** plausible value for the number of reefs reported with at least 1% bleaching of hard coral will be?	
iii	Realistically, what is your **best guess** for the number of reefs that will be reported to have at least 1% bleaching of hard coral?	
iv	**How confident are you** that your interval, from lowest to highest, could capture the reported number of reefs with at least 1% bleaching of hard coral? Please enter a number between 50 and 100%	%

2. Comments

Please enter any comments, additional knowledge or justification that you have about this question and / or your estimate. This will be shared with the group in Round 2.

3. Useful Links

Please enter any useful web links (URLs) you would like to contribute to your group

1		4	
2		5	
3		6	

Question 3 Asian Green Mussel Detections in Queensland

“How many unique detections of Asian Green Mussel (Perna viridis) *will be recorded by the Queensland Department of Agriculture and Fisheries between 1 March 2016 and 30 June 2016".*

Clarification: *Asian Green Mussel (Perna viridis) is an invasive species which can be carried in ballast water. The Queensland Department of Agriculture and Fisheries (DAF) has been collecting records of Asian Green Mussel detections in Queensland since 2001. Reports of detections are generally submitted to the DAF by people involved in routine slipping of ships and vessels at Queensland Ports. For this question we want you to estimate how many unique detections of AGM will be reported and subsequently recorded by DAF between 1 March 2016 and 30 June 2016 (inclusive). For this question we are not interested in whether the pest subsequently establishes simply that it is detected and recorded by DAF. Note that multiple reports to DAF of the same incident will only count as one record. Also the detection in not made in Queensland, then it will not count (e.g. if a vessel has visited QLD but the detection was not made until it moved to another state or country).*

Resolution: *The question will be resolved by Queensland Department of Agriculture and Fisheries on 15 July 2016.*

Additional Information:

a. Since 2001 there have been ***10 reports*** *of Asian Green Mussels recorded by the Queensland Department of Agriculture and Fisheries.*

Useful Links:

i. AGM Fact sheet https://www.daf.qld.gov.au/plants/weeds-pest-animals-ants/legislation-policies-permits/legislation/faqs/asian-mussels

1. Your estimate for Question 3

		Please enter your estimate (a whole number)	
i	Realistically, what do you think the **lowest** plausible number of unique detections of Asian Green Mussels will be?		
ii	Realistically, what do you think the **highest** number of unique detections of Asian Green Mussels will be?		
iii	Realistically, what is your **best guess** for the number of unique detections of Asian Green Mussels?		
iv	**How confident are you** that your interval, from lowest to highest, could capture the number of unique detections of Asian Green Mussels? Please enter a number between 50 and 100%		%

2. Comments

Please enter any comments, additional knowledge or justification that you have about this question and /or your estimate. This will be shared with the group in Round 2.

3. Useful Links

Please enter any useful web links (URLs) you would like to contribute to your group

1		4	
2		5	
3		6	

Question 4 Prevalence of White Syndrome Coral Disease on Reef 21060

"What will be the total number of coral colonies reported with White Syndrome (a coral disease) on Reef 21060 in the Mackay-Pompey Region, by the Australian Institute of Marine Science (AIMS) during SCUBA surveys undertaken between 1 March and 30 June, 2016?"

Clarification: White Syndrome is a coral disease present on the Great Barrier Reef. This question aims to determine the extent to which Reef 21060 will be affected by White Syndrome when next surveyed by AIMS in 2016. Surveys for White Syndrome are undertaken by AIM as part of the Long Term Monitoring Porgram, using SCUBA searches along fixed transects. During SCUBA surveys a 2 m belt (1 m either side of the central tape measure) is visually searched along 50 m fixed line transects, and the number of coral colonies which are detected to have White Syndrome are recorded. Reef 21060 is located in the Mackay Pompey Region, and is currently scheduled to be surveyed in March, 2016, however, the survey realistically may take place anytime between 1 March, 2016 and 30 June , 2016. During these surveys the total number of coral colonies with signs of White Syndrome will be recorded.

Resolution: The answer to this question will be resolved when AIMS publishes data for Reef 21060 as part of their reporting for the Mackay / Pompey region for the 2015 / 2016 financial year. See "previous reports" in useful links.

Additional Information:

a. Previous trends in White Syndrome for Reef 21060 as detected by AIMS during SCUBA searches.

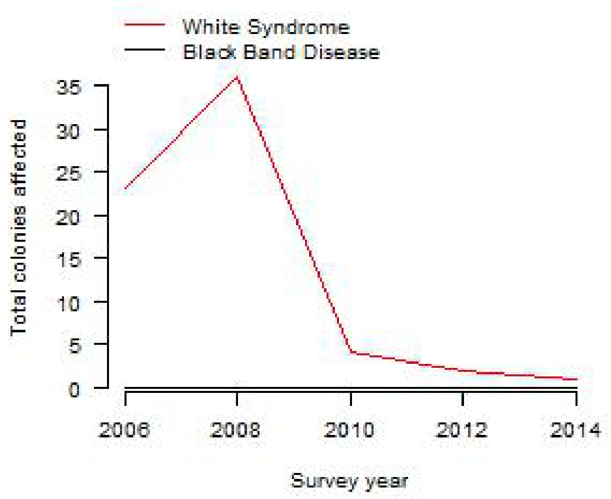

Useful Links:

i. Previous data for Reef 21060 http://data.aims.gov.au/reefpage2/rpdetail.jsp?fullReefID=21060S&sampleType=VPOINT

ii. Previous reports for the LTMP http://www.aims.gov.au/docs/research/monitoring/reef/latest-surveys.html

iii. Survey procedure http://www.aims.gov.au/documents/30301/20e3bf4f-4b3b-4808-ac02-c15c2912c3f2

1. Your estimate for Question 4

		Please enter your estimate (a whole number)
i	Realistically, what do you think the **lowest** plausible number of coral colonies detected with White Syndrome on Reef 21060 will be?	
ii	Realistically, what do you think the **highest** plausible number of coral colonies detected with White Syndrome on Reef 21060 will be?	
iii	Realistically, what is your **best guess** for the number of coral colonies that will be detected with White Syndrome on Reef 21060?	
iv	**How confident are you** that your interval, from lowest to highest, could capture the actual number of coral colonies that will be detected with White Syndrome on Reef 21060? Please enter a number between 50 and 100%	%

2. Comments

Please enter any comments, additional knowledge or justification that you have about this question and /or your estimate. This will be shared with the group in Round 2.

3. Useful Links

Please enter any useful web links (URLs) you would like to contribute to your group

1		4	
2		5	
3		6	

Question 5 Commercial Catch of Coral Trout

"How many tonnes of Coral Trout will be r caught in Queensland by the Commercial Line Fishery in April 2016?"

Clarification: Coral Trout is a species which is commercially caught by the Coral Sea Fishery in Queensland. Each month the tonnage of catch is reported to Fisheries Queensland, who publicly report this quantity through their QFISH database. This question asks how many tonnes of Coral Trout will be reported to be caught from 1 April 2016 to 30 April 2016. Only the tonnes of Coral Trout which are caught by the commercial line fishery and reported to Fisheries Queensland and subsequently reported on the QFISH database are included in the estimate.

Resolution: This question will be resolved by QFISH an online database hosted by Fisheries Queensland on 1 September 2016. The following options will be selected: Logbook type: "Commercial", Fishing Method "Line", Species Group: "Coral Trout", and then only selecting "Coral Trout", Calendar Year "2016", Month " 04_April". Weight (t).

Additional Information:

a. Previous harvest of Coral Trout for the Commercial Line Fishery from QFISH.

Calendar Year	Month	Licences	Weight (t)
2012	04 - April	86	70.53
2013	04 - April	93	69.23
2014	04 - April	77	53.92

Useful Links:

i. QFISH http://qfish.fisheries.qld.gov.au/Query/ViewResults?CubeId=7&PredefinedQueryId=cbb0d21f-ed24-4fb7-b0b4-d7422c561e9b&ViewKind=Pivot

1. Your estimate for Question 5

		Please enter your estimate (2 decimal places)	
i	Realistically, what do you think the **lowest** plausible catch (tonnes) of coral trout will be?		
ii	Realistically, what do you think the **highest** plausible catch (tonnes) of coral trout will be?		
iii	Realistically, what is your **best guess** for the catch (tonnes) of coral trout?		
iv	**How confident are you** that your interval, from lowest to highest, could capture the reported catch (tonnes) of coral trout? Please enter a number between 50 and 100%		%

2. Comments

Please enter any comments, additional knowledge or justification that you have about this question and /or your estimate. This will be shared with the group in Round 2.

3. Useful Links

Please enter any useful web links (URLs) you would like to contribute to your group

1		4	
2		5	
3		6	

Question 6 Marine Turtles

“How many turtles will be reported and confirmed as stranded by the Queensland Department of Environment and Heritage Protection for the whole of the Queensland East Coast between 1 January 2016 to 31 March 2016?”

Clarification: Each year the number of marine turtles which end up stranded on Queensland’s east coast are recorded by the Queensland Department of Environment and Heritage Protection in their strandings database. The strandings database includes sick, injured, debilitated or dead marine turtles. The East Coast of Queensland refers to entire eastern coastline of Queensland adjacent to the Coral Sea. This question refers to the number of marine turtles which will be found, and reported to the Queensland Department of Environment and Heritage Protection as stranded, and subsequently confirmed to be a stranded turtle, for the period between 1 January and 31 March 2016.

Resolution: The question will be resolved by the Queensland Department of Environment and Heritage Protection on their strandings database for “Turtle Strandings for the period of 1 January to 31 March 2016” at some time before 1 September 2016.

Additional Information:

a. Turtle strandings for the period 1 January to 31 March 2015 for previous years

Location	2015	2014	2013	2012	2011	2010	2009
Total for east coast of Queensland	92	217	192	251	182	166	177

Useful Links:

i. Marine turtle strandings https://www.ehp.qld.gov.au/wildlife/caring-for-wildlife/marine-strandings-update.html
ii. Marine Turtle report for 2011 https://www.ehp.qld.gov.au/wildlife/caring-for-wildlife/pdfs/turtle-report-2011.pdf

1. Your estimate for Question 6

		Please enter your estimate (whole number)	
i	Realistically, what do you think the **lowest** number of turtles confirmed to be stranded will be?		
ii	Realistically, what do you think the **highest** number of turtle confirmed to be stranded will be?		
iii	Realistically, what is your **best guess** for the number of turtles that will be confirmed to be stranded?		
iv	**How confident are you** that your interval, from lowest to highest, could capture the confirmed number of turtles reported to be stranded? Please enter a number between 50 and 100%		%

2. Comments

Please enter any comments, additional knowledge or justification that you have about this question and /or your estimate. This will be shared with the group in Round 2.

3. Useful Links

Please enter any useful web links (URLs) you would like to contribute to your group

1		4	
2		5	
3		6	

Question 7 Shark Control in the Mackay region

"How many individual sharks (target species only) will be caught by the Queensland shark control program in May 2016?"

Clarification: The Queensland Shark Control Program has been operating since 2001, however, nets and drumlines have been in place in the Mackay region since 1963. The nets and drumlines in the Mackay region are checked every second day weather permitting, whereupon the number of target and non-target species caught in the nets and drumlines is recorded. This question seeks your opinion on what you realistically think the total number of target sharks caught in the Mackay region will be in May 2016? Target shark species are mainly Tiger Sharks, Bull Sharks and Whalers, but include most shark species (see Target Species in useful links). All sharks whether dead or alive are recorded. However, in order to be included the Queensland Shark Control Program would need to detect the shark in the drumlines or nets and subsequently identify it. Note that this question is asking for the total number of individuals rather than the number of different species. Only sharks which are detected during surveys between the 1 May 2016 and the 31 May 2016 will be included in the count.

Resolution: This question will be resolved when the Queensland Government release their Shark Control Program Shark Catch Statistics on their website (see useful links).

Additional Information:

a. Previous number of sharks caught for the Mackay area during the month of May 2001-2015

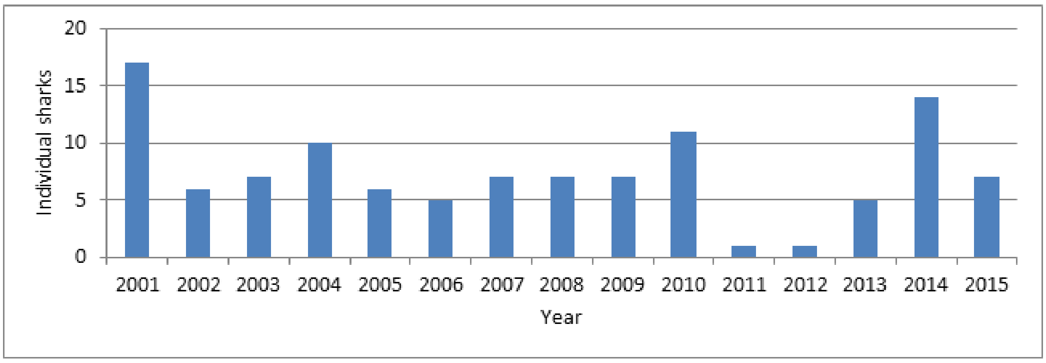

Useful Links:

i. Location of shark nets — https://www.daf.qld.gov.au/__data/assets/pdf_file/0005/69566/scp-equip-location-mackay-edit.pdf
ii. About the shark program — https://www.daf.qld.gov.au/fisheries/services/shark-control-program
iii. Previous data — https://www.daf.qld.gov.au/fisheries/services/shark-control-program/catch-numbers
iv. Target species, Appendix C — https://www.daf.qld.gov.au/__data/assets/pdf_file/0007/310939/public-information-package-shark-control-program.pdf

1. Your estimate for Question 7

		Please enter your estimate (whole number)	
i	Realistically, what do you think the **lowest** number of sharks caught will be?		
ii	Realistically, what do you think the **highest** number of sharks caught will be?		
iii	Realistically, what is your **best guess** for the number of sharks that will be caught?		
iv	**How confident are you** that your interval, from lowest to highest, could capture the number of sharks caught? Please enter a number between 50 and 100%		%

2. Comments

Please enter any comments, additional knowledge or justification that you have about this question and /or your estimate. This will be shared with the group in Round 2.

3. Useful Links

Please enter any useful web links (URLs) you would like to contribute to your group

1		4	
2		5	
3		6	

Question 8 Water Temperature in the Southern Great Barrier Reef

"How many days in April 2016 will the maximum water temperature reach 28.0° Celsius or above at Heron Island?"

Clarification: This question refers specifically to the water temperature (Celsius) recorded at Heron Island by "Heron Island Sensor Float 1", which is positioned 0.3 metres below the sea-surface, between 1st April 2016 and 30th April 2016 (inclusive). If the weather station fails to record a reading for a day it will not be included in the maximum. If no readings are recorded by the Heron Island Sensor Float 1, between 1 April 2016 and 30 April 2016 then the question will be void.

Resolution: The data to resolve this question will be found on the historic data tool on the AIMS website (see useful links), for "Heron Island Sensor Float 1", and selecting the daily maximum for April 2016 (from the 1 – 30 April inclusive). The data will be obtained from a Rule-Based Quality Control data set which aims to avoid illogical values.

Additional Information:

a. Daily maximum water temperature recorded by Sensor Float 1 at Heron Island in Apirl 2015, from the AIMS weather station.

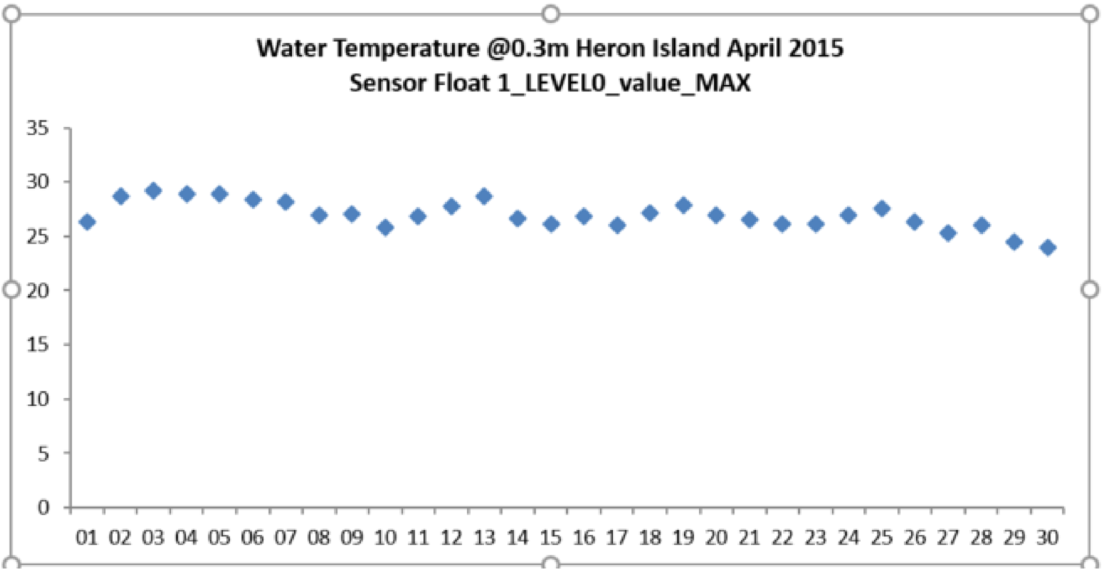

Useful Links:

i. Daily readings http://weather.aims.gov.au/#/station/130

ii. Heron Island http://maps.aims.gov.au/index.html?intro=false&z=11&ll=151.98%2C-23.45&l0=ea_World_NE2-coast-cities-reefs_Baselayer%2Caims_aims%3AWeatherStation

iii. Long term dataset http://data.aims.gov.au/aimsrtds/datatool.xhtml?from=2011-02-05&thru=2016-02-06&period=DAY&aggregations=MAX&channels=19

1. Your estimate for Question 8

		Please enter your estimate (1 decimal place)	
i	Realistically, what do you think the **lowest** number of days the water temperature will reach 28.0° Celsius or above at Heron Island?"		
ii	Realistically, what do you think the **highest** number of days the water temperature will reach 28.0° Celsius or above at Heron Island?"		
iii	Realistically, what is your **best guess** for the number of days the water temperature will reach 28.0° Celsius or above at Heron Island?"		
iv	**How confident are you** that your interval, from lowest to highest, could capture the number of days the water temperature will reach 28.0° Celsius or above at Heron Island?" Please enter a number between 50 and 100%		%

2. Comments

Please enter any comments, additional knowledge or justification that you have about this question and /or your estimate. This will be shared with the group in Round 2.

3. Useful Links

Please enter any useful web links (URLs) you would like to contribute to your group

1		4	
2		5	
3		6	

Question 9 Discharge Volume (Megalitres) from the Burdekin River

"What will be the total discharge volume (Megalitres) for the Burdekin River, Queensland in April 2016?"

Clarification: This question relates specifically to the "Stream Discharge Volume (Megalitres)" recorded ONLY at the monitoring station "120006B Burdekin River at Clare in the Burdekin region" from 1 April to 30 April 2016 (inclusive) as reported on the Water Monitoring Information Portal hosted by the Queensland Government.

Resolution: This question will be resolved on 15 May 2016 by going to the "120006B Burdekin River at Clare" station (refer to useful links), and selecting "Stream Discharge Volume" (Megalitres), and selecting "Custom" for the "Period" column, "Download" for the "Output" column and "Daily" for the "Data Interval" column. The start date will be entered as 00:01_01/04/2016, and the end date will be entered as 00:00_30/04/2016. The sum of all of the daily totals will be used to calculate total discharge for the month.

Additional Information:

a. Total volume of discharge in April 2015 = **64,685 Megalitres**

b. Cumulative volume discharge (Megalitres) at the Burdekin river during April 2015.

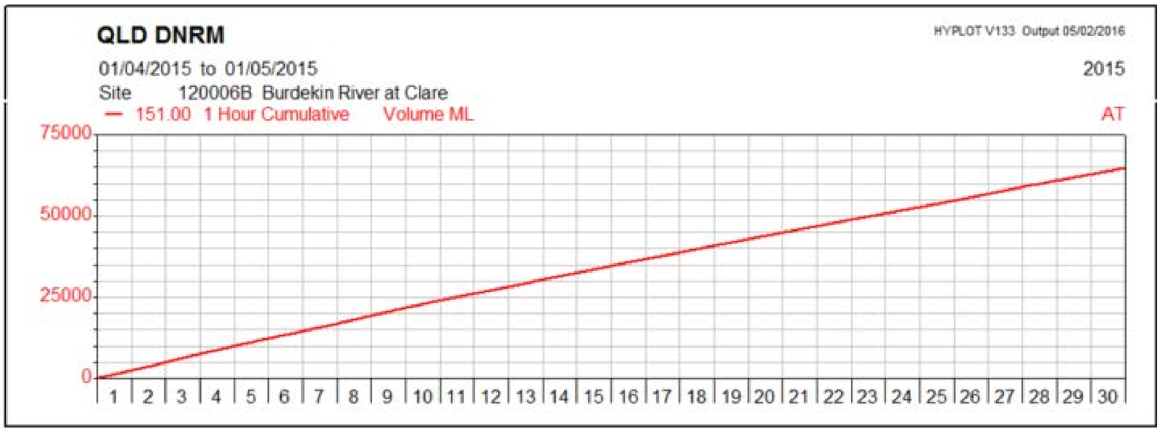

Useful Links:

i. "120006B Burdekin River at Clare" https://water-monitoring.information.qld.gov.au?ppbm=120006B&rs&1&rscf_org

ii. AIMS inshore Water Quality monitoring reports http://eatlas.org.au/rrmmp/gbr-aims-inshore-water-quality

1. Your estimate for Question 9

		Please enter your estimate (whole number)
i	Realistically, what do you think the **lowest** reported discharge volume (megalitres) from the Burdekin River will be?	
ii	Realistically, what do you think the **highest** reported discharge volume (megalitres) from the Burdekin River will be?	
iii	Realistically, what is your **best guess** for the reported discharge volume (megalitres) from the Burdekin River?	
iv	**How confident are you** that your interval, from lowest to highest, could capture the reported discharge volume (megalitres) from the Burdekin River? Please enter a number between 50 and 100%	%

2. Comments

Please enter any comments, additional knowledge or justification that you have about this question and /or your estimate. This will be shared with the group in Round 2.

3. Useful Links

Please enter any useful web links (URLs) you would like to contribute to your group

1		4	
2		5	
3		6	

Question 10 Chlorophyll Levels Detected at Pine Island

"What will be the average Chlorophyll level (ugL $^{-1}$) for at Pine Island in the Mackay Whitsunday region, in March 2016 recorded by the Wet Labs Eco FLNTUSB?"

Clarification: The Australian Institute of Marine Science measure the Chlorophyll levels at Pine Island in the Mackay –Whitsunday region of Queensland using Eco FLNTUSB instruments. The results inform the Marine Monitoring Program's (MMP) Inshore Water Quality Monitoring (more information provided below). This question relates specifically to the Eco FLNTUSB instrument currently deployed at Pine Island in the Mackay Whitsunday region. We are interested in what the average daily chlorophyll level will be for March 2016 (averaged from 1 March, 2016 to 31 March 2016 (inclusive)).

Resolution: This question will be resolved on 1 July 2016 by the AIMS Marine Monitoring Program.

Additional Information:

a. Eco FLNTUSB chlorophyll monitoring.

The Eco FLNTUSB instruments deployed by the AIMS MMP record in situ measurements of chlorophyll fluorescence, turbidity and temperature. The term "Chlorophyll" is used by AIMS rather than "Chlorophyll-α", because the Eco FLNTUSB instruments can only measure the fluorescence from the chlorophyll pigments AND their degradation products, rather than specifically measuring Chlorophyll-α levels. Each instrument is checked prior to deployment and on collection to ensure that measurements are calibrated to maximum and minimum levels of chlorophyll. After retrieval from the field locations, the instruments are cleaned and data downloaded and converted from raw instrumental records into actual measurement units (μg L^{-1} for chlorophyll fluorescence) according to standard procedures by the manufacturer. Deployment information and all raw and converted instrumental records are then stored in an Oracle-based data management system developed by AIMS. Records are quality-checked using a time-series data editing software. Instrumental data are validated by comparison with chlorophyll and suspended solid concentrations obtained by analyses of water samples collected close to the instruments, during change over.

b. Previous trends at Pine Island (source AIMS 2011 inshore water quality program, see useful links)

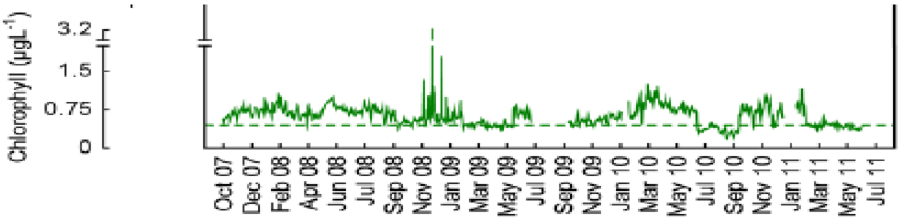

Useful Links:

i. Chlorophyll monitoring — http://www.aims.gov.au/docs/data-centre/chlorophyllmonitoring.html

ii. AIMS 2011 Inshore Water Quality Program — http://eatlas.org.au/rrmmp/gbr-aims-inshore-water-quality

1. Your estimate for Question 10

		Please enter your estimate (2 decimal place)
i	Realistically, what do you think the **lowest** average chlorophyll level (ugL^{-1}) will be?	
ii	Realistically, what do you think the **highest** average chlorophyll level (ugL^{-1}) will be?	
iii	Realistically, what is your **best guess** for the average chlorophyll level (ugL^{-1})?	
iv	**How confident are you** that your interval, from lowest to highest, could capture the average chlorophyll level (ugL^{-1}) for Pine Island? Please enter a number between 50 and 100%	%

2. Comments

Please enter any comments, additional knowledge or justification that you have about this question and /or your estimate. This will be shared with the group in Round 2.

3. Useful Links

Please enter any useful web links (URLs) you would like to contribute to your group

1		4	
2		5	
3		6	

Question 11 Wind Speed at Davies Reef

"What will be the highest maximum daily wind-speed (averaged maximum, km/hr) recorded for Davies Reef in May 2016?"

Clarification: This question asks what the highest maximum daily wind-speed (averaged maximum, km/hr) will be for Davies Reef for May 2016 as recorded by the Australian Institute of Marine Science. Note that four readings for the maximum wind-speed are recorded each day at Davies Reef. For the purpose of this questions, the **"maximum daily"** wind-speed is actually an average across these four readings. This question is asking what the **highest** "maximum daily" (averaged maximum) wind-speed will be in May 2015. It is possible that the recording instruments will fail, in such a case, only the days from 1 May - 31 May (inclusive) which are recorded will be used to verify the question.

Resolution: The question will be resolved on 6 June 2016. It will be resolved by the AIMS Historic Data Tool (see useful links), and selecting 1 May to 31 May 2016, by selecting quality controlled data, grouping: day + maximum, selecting data: Wind, Davies Reef, Platform Speed (scalar avg 10 min), and exporting data. For each day, the four readings will be averaged. The highest of these averaged wind speeds will be taken as the answer.

Additional information:

a. In May 2015 the highest maximum daily windspeed (averaged maximum) was 69.00 km / hr

Useful Links:

i. Wind speed (past month) — http://data.aims.gov.au/aimsrtds/datatool.xhtml?from=2016-02-18&thru=2016-026&qc=LEVEL1&period=DAY&aggregations=MAX&channels=73

ii. Wind Speed Previous five years — http://data.aims.gov.au/aimsrtds/datatool.xhtml?from=2011-02-25&thru=2016-02-26&qc=LEVEL1&period=DAY&aggregations=MAX&channels=73

1. Your estimate for Question 11

		Please enter your estimate (2 decimal places)
i	Realistically, what do you think the **lowest** value for the highest maximum daily wind speed (averaged maximum, km /hr) will be?	
ii	Realistically, what do you think the **highest** value for the highest maximum daily wind speed (averaged maximum, km /hr) will be?	
iii	Realistically, what is your **best guess** for the value for the highest maximum daily wind speed (averaged maximum, km /hr)?	
iv	**How confident are you** that your interval, from lowest to highest, could capture the maximum daily wind speed (averaged, maximum km /hr) for Davies Reef? Please enter a number between 50 and 100%	%

2. Comments

Please enter any comments, additional knowledge or justification that you have about this question and /or your estimate. This will be shared with the group in Round 2.

3. Useful Links

Please enter any useful web links (URLs) you would like to contribute to your group

1		4	
2		5	
3		6	

Question 12 Average maximum Air Temperature Hamilton Island

"What will be the average maximum air temperature (°C) recorded by the Australian Bureau of Meteorology at Hamilton Island for the month of May, 2016?"

Clarification: The Australian Bureau of Meteorology has a weather station at Hamilton Island on the Great Barrier Reef. The weather station records the daily minimum and maximum temperature for each day of the month. This question asks specifically what you believe will be the average of daily maximum temperatures from 1 May - 31 May 2016. We would like you to provide your answer to one decimal place.

Resolution: The question will be resolved in 15th June, 2016 by the Australian Bureau of Meteorology on their page for Hamilton Island, under "other times and other places" and for "May 16". The value for the mean of the maximum temperature will be taken as the truth.

Additional Information:

a. The average maximum May 2015 was **24.8 C**

Useful Links:

i. Latest weather observations for Hamilton Island: http://www.bom.gov.au/climate/dwo/IDCJDW4054.latest.shtml

ii. Weather observations for April 2015 at Hamilton Island http://www.bom.gov.au/climate/dwo/201504/html/IDCJDW4054.201504.shtml

1. Your estimate for Question 12

		Please enter your estimate (1 decimal places)
i	Realistically, what do you think the **lowest** average maximum air temperature will be for May 2016?	
ii	Realistically, what do you think the **highest** average maximum air temperature will be for May 2016?	
iii	Realistically, what is your **best guess** for the average maximum air temperature in May 2016?	
iv	**How confident are you** that your interval, from lowest to highest, could capture the average maximum air temperature in May 2016? Please enter a number between 50 and 100%	%

2. Comments

Please enter any comments, additional knowledge or justification that you have about this question and /or your estimate. This will be shared with the group in Round 2.

3. Useful Links

Please enter any useful web links (URLs) you would like to contribute to your group

1		4	
2		5	
3		6	

Question 13 Turbidity in the Wet Tropics

"What will be the mean turbidity (NTU) for High West (located on High Island) for the month of April, 2016, as recorded by the Australian Institute of Marine Science using their ECO FLNTUSB instruments?"

Clarification: The Australian Institute of Marine Science measures the turbidity levels at High Island, the sampling site is referred to as "High West". High Island is located in the Wet Tropics region of Queensland. The Eco FLNTUSB instruments used by AIMS inform the Marine Monitoring Program's (MMP) Inshore Water Quality Monitoring (more information provided below). This question relates specifically to the Eco FLNTUSB instrument currently deployed at High West on High Island in the Wet Tropics region.

Resolution: This question will be resolved by 30 June 2016 by the AIMS Marine Monitoring Program.

Additional Information:

a. Annual Mean Turbidity: taken from the MMP AIMS Inshore monitoring report for 2013-2014 for High West, on High Island in the Wet Tropics region, Queensland.

Monitoring period	Annual Mean Turbidity (NTU)	Number of sampling days
Oct 2007 – Sep 2008	0.81	356
Oct 2008 – Sep 2009	0.84	365
Oct 2009 – Sep 2010	1.20	365
Oct 2010 – Sep 2011	1.56	365
Oct 2011 – Sep 2012	1.08	366
Oct 2012 – Sep 2013	1.55	365
Oct 2013 – Sep 2014	1.27	213

Useful Links:

i. Marine Monitoring Program report for 2013-2014 http://elibrary.gbrmpa.gov.au/jspui/handle/11017/2975

1. Your estimate for Question 13

		Please enter your estimate (2 decimal places)	
i	Realistically, what do you think the **lowest** mean turbidity (NTU) will be?		
ii	Realistically, what do you think the **highest** mean turbidity (NTU) will be?		
iii	Realistically, what is your **best guess** for the mean turbidity (NTU)?		
iv	**How confident are you** that your interval, from lowest to highest, could capture the reported turbidity (NTU) ? Please enter a number between 50 and 100%		%

2. Comments

Please enter any comments, additional knowledge or justification that you have about this question and /or your estimate. This will be shared with the group in Round 2.

3. Useful Links

Please enter any useful web links (URLs) you would like to contribute to your group

1		4	
2		5	
3		6	

Question 14 EL Nino Events

"What will be the average sea-surface temperature (°C) for the month of June 2016 within the Nino 3.4 region as reported by the Climate Prediction Center of the National Oceanic and Atmospheric Administration (NOAA)?"

Clarification: The Nino 3.4 region is shown on the map below. The average sea-surface temperature of the Niño 3.4 region is used to detect whether the equatorial Pacific region has entered an El Niño or La Niña weather pattern. This question seeks to understand what you think the average sea-surface temperature will be for the Month of June 2016.

Resolution: The answer will be reported by the National Weather Service Climate Prediction Center of NOAA, in their ongoing monthly monitoring program for Sea-Surface Temperature "Monthly- ERSSTv4 (1981-2010 base period), Niño 3.4 (5°North-5°South)(170-120°West)" (links below), in the "NINO 3.4" column and the row for the year "2016", and month "6". The data will be downloaded on 15 July, 2016.

Additional Information:

a. The Nino 3.4 region

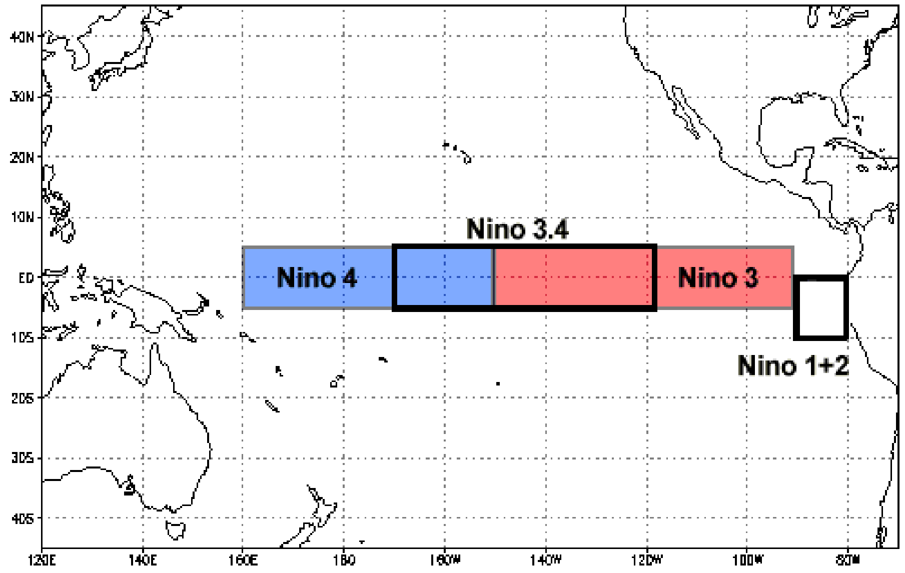

b. The average sea-surface temperature for June 2015 was 28.70 °C

Useful Links:

i. NOAA https://www.ncdc.noaa.gov/teleconnections/enso/indicators/sst.php

ii. Previous trends http://www.cpc.ncep.noaa.gov/data/indices/ersst4.nino.mth.81-10.ascii

THE UNIVERSITY OF MELBOURNE

cebra Centre of Excellence for Biosecurity Risk Analysis

1. Your estimate for Question 14

		Please enter your estimate (2 decimal places)
i	Realistically, what do you think the **lowest** average sea-surface temperature will be?	
ii	Realistically, what do you think the **highest** average sea-surface temperature will be?	
iii	Realistically, what is your **best guess** for the average sea-surface temperature?	
iv	**How confident are you** that your interval, from lowest to highest, could capture the reported sea-surface temperature? Please enter a number between 50 and 100%	%

2. Comments

Please enter any comments, additional knowledge or justification that you have about this question and /or your estimate. This will be shared with the group in Round 2.

3. Useful Links

Please enter any useful web links (URLs) you would like to contribute to your group

1		4	
2		5	
3		6	

Question 15 The Spread of Zika Virus Throughout the European Union

"How many European Union member states will the World Health Organization report as having at least one laboratory-confirmed human case of any strain of Zika virus for the month of April 2016?"*

Clarification: The World Health Organization (WHO) held a special session on 4 February 2016 on the Zika virus for quick action against the infection linked to thousands of birth defects in Brazil that is spreading through Latin America and the Caribbean (Reuters- see useful links). The virus, carried by the *Aedes aegypti* mosquito, was originally found in tropical and subtropical zones, but is today found on all continents except Antarctica.

*European patients whose cases of Zika virus have been confirmed as part of the outbreak in the Americas and are transported back to Europe for treatment will not count. Cases reported as "suspected" prior to the question's closing date will not count, even if they are "confirmed" after the question's closing date. For a list of the 28 European Union member states (see useful links). Please note this question refers to all reports confirmed by the World Health Organisation (WHO) during the month of April (including those made for member states which have previously been confirmed to contain Zika virus).

Resolution: Outcome will be determined by WHO's Disease outbreak news for Zika virus infection, found at the World Health Organisation (see useful links) or in credible open source media reports (e.g., Reuters, BBC, AP).

Additional Information: None

Useful Links:

i.	World Health Organisation Reports for Zika Virus	http://www.who.int/csr/don/archive/disease/zika-virus-infection/en/
ii.	List of 28 member countries for the EU	http://europa.eu/about-eu/countries/member-countries/index_en.htm
iii.	News article about Zika virus	http://www.reuters.com/article/us-health-zika-idUSKCN0V523W

1. Your estimate for Question 15

		Please enter your estimate (whole number)
i	Realistically, what do you think will be the **lowest** number of EU member states reported to have at least one laboratory-confirmed human case of any strain of Zika virus for the month of April, 2016?	
ii	Realistically, what do you think will be the **highest** number of EU member states reported to have at least one laboratory-confirmed human case of any strain of Zika virus for the month of April, 2016?	
iii	Realistically, what is your **best guess** for the number of EU member states that will be reported to have at least one laboratory-confirmed human case of any strain of Zika virus for the month of April, 2016?	
iv	**How confident are you** that your interval, from lowest to highest, could capture the number of EU member states will be reported to have at least one laboratory-confirmed human case of any strain of Zika virus for the month of April, 2016? Please enter a number between 50 and 100%	%

2. Comments

Please enter any comments, additional knowledge or justification that you have about this question and /or your estimate. This will be shared with the group in Round 2.

3. Useful Links

Please enter any useful web links (URLs) you would like to contribute to your group

1		4	
2		5	
3		6	

Question 16 The Price of Gold

"What will be the closing spot price of gold on 30 May 2016?"

Clarification: Gold prices declined in 2015 as the Federal Reserve System signaled its first interest-rate increase in nearly a decade, but has recovered recently amid global financial market turmoil and fears that economic growth is slowing (WSJ- see useful links). We are interested in what you think the spot price of gold will be at the end of the day (EST) on the 30 May, 2016.

Resolution: Outcome will be determined by the end-of-day (i.e., 23:59:59 EST on 30 May 2016) closing spot price for gold in U.S. dollars according to Bloomberg, at http://www.bloomberg.com/quote/xauusd:cur. In case of delayed reporting or problems with the Bloomberg website, reporting by other credible open sources may be used.

Useful Links:

i. WSJ News article http://www.wsj.com/articles/gold-gains-as-global-markets-plunge-1453292225?tesla=y

ii. Spot price of Gold http://www.bloomberg.com/quote/xauusd:cur

1. Your estimate for Question 16

		Please enter your estimate (2 decimal places)
i	Realistically, what do you think the **lowest** spot price of gold will be?	
ii	Realistically, what do you think the **highest** spot price of gold will be?	
iii	Realistically, what is your **best guess** for the spot price of gold?	
iv	**How confident are you** that your interval, from lowest to highest, could capture the reported spot price of gold? Please enter a number between 50 and 100%	%

2. Comments

Please enter any comments, additional knowledge or justification that you have about this question and /or your estimate. This will be shared with the group in Round 2.

3. Useful Links

Please enter any useful web links (URLs) you would like to contribute to your group

1		4	
2		5	
3		6	

Question 17 The UK Referendum

*"What will be the final percentage of votes made **in favour** of the United Kingdom remaining a member of the European Union during the UK referendum to be held on 23 June, 2016?"*

Clarification: On Saturday the 20th February, 2016, David Cameron, the Prime Minister of the United Kingdom called a referendum to determine whether the United Kingdom should remain in the European Union or leave the European Union. The referendum will take place on 23 June 2016.

This question aims to determine what percentage of people who choose and are permitted to vote in the upcoming United Kingdom referendum to be held on 23 June, 2016, will vote in favour of the United Kingdom remaining a member of the European Union. Please note that only valid votes will be included in the final percentage.

Resolution: This answer to this question will be resolved by the United Kingdom Electoral Commission when they announce the final result of the referendum either on their webpage or in credible open source media reports (e.g., Reuters, BBC, AP).

Useful Links:

i.	About the Referendum	http://www.bbc.com/news/uk-politics-32810887
ii.	Opinion Polling	http://whatukthinks.org/eu/opinion-polls/poll-of-polls/
iii.	The UK Electoral Commission	http://www.electoralcommission.org.uk/find-information-by-subject/elections-and-referendums/upcoming-elections-and-referendums/eu-referendum

1. Your estimate for Question 17

		Please enter your estimate (2 decimal places)
i	Realistically, what do you think the **lowest** percentage of people voting in **favour** of the UK remaining a member of the EU will be?	
ii	Realistically, what do you think the **highest** percentage of people voting in favour of the UK remaining a member of the EU will be?	
iii	Realistically, what is your **best guess** for the percentage of people voting in favour of the UK remaining a member of the EU?	
iv	**How confident are you** that your interval, from lowest to highest, could capture the percentage of people voting in favour of the UK remaining a member of the EU? Please enter a number between 50 and 100%	%

2. Comments

Please enter any comments, additional knowledge or justification that you have about this question and /or your estimate. This will be shared with the group in Round 2.

3. Useful Links

Please enter any useful web links (URLs) you would like to contribute to your group

1		4	
2		5	
3		6	

Question 18 The Stock Price of Twitter

"What will Twitter's end-of-day stock price be on 30 May 2016?"

Clarification: Twitter's stock price is reported on Nasdaq.com (see useful links). We are interested in what you realistically think the stock price will be at the end of the day on the 30 of May 2016?

Resolution: The outcome will be determined by the end-of-day (i.e., 15:59:59 EST on 30 May 2016) stock price for Twitter (TWTR) in U.S. dollars according to Nasdaq National Market, at http://www.nasdaq.com/symbol/twtr. In case of delayed reporting or problems with the Nasdaq website, reporting by other credible open sources may be used.

Useful Links:

i. News reports https://www.technologyreview.com/s/546286/is-facebook-about-to-kill-off-twitter/
ii. Twitter's Stock Price http://www.nasdaq.com/symbol/twtr.

1. Your estimate for Question 18

		Please enter your estimate (2 decimal places)
i	Realistically, what do you think the **lowest** end-of-day stock price (USD) for twitter will be?	
ii	Realistically, what do you think the **highest** end-of-day stock price (USD) for twitter will be?	
iii	Realistically, what is your **best guess** end-of-day stock price (USD) for twitter will be?	
iv	**How confident are you** that your interval, from lowest to highest, could capture the end-of-day stock price (USD) for twitter? Please enter a number between 50 and 100%	%

2. Comments

Please enter any comments, additional knowledge or justification that you have about this question and /or your estimate. This will be shared with the group in Round 2.

3. Useful Links

Please enter any useful web links (URLs) you would like to contribute to your group

1		4	
2		5	
3		6	

Question 19 The Throughput (tonnes) from Abbot Point Port.

“What will be throughput (tonnes) from Abbot Point Port in May 2016 as reported by the North Queensland Bulk Ports Corporation?”

Clarification: Abbot Point is a bulk coal terminal located between Townsville and Mackay. It is operated by the North Queensland Bulk Ports Corporation (NQBP). Each month the amount of coal (tonnes) exported from Abbot Point is publicly reported by NQBP (referred to as “throughput”). For this question we would like you to tell us what the throughput (tonnes) will be for May 2016, as reported by North Queensland Bulk Ports Corporation for their Abbot Point Port.

Resolution: The question will be resolved when North Queensland Bulk Ports Corporation publish the throughput for their Abbot point port for May 2016. Currently this information is provided on the NQBP webpage for Abbot Point Port (see useful links).

Additional Information:

a. Monthly throughput (tonnes) of Abbot Point Port reported by North Queensland Bulk Ports Corporation 2011-2016.

	2011/12	2012/13	2013/14	2014/15	2015/16
**July**	1,082,351	1,061,932	1,914,825	2,549,533	2,137,081
**Aug**	1,374,398	1,434,153	2,071,065	2,689,449	2,325,753
**Sep**	1,295,225	1,173,161	1,542,640	2,388,651	3,071,717
**Oct**	1,369,123	1,128,813	2,012,186	2,594,124	2,063,059
**Nov**	1,150,840	1,361,358	1,991,152	2,447,860	2,104,546
**Dec**	1,045,067	1,737,743	2,032,412	2,546,134	2,011,407
**Jan**	1,161,504	1,879,052	1,487,780	2,031,489	2,162,742
**Feb**	865,328	1,237,236	2,019,920	2,091,416	
**Mar**	970,632	1,377,904	1,439,730	2,676,541	
**Apr**	1,183,926	1,795,160	1,808,698	2,425,631	
**May**	1,186,281	1,881,360	2,166,790	1,908,950	?
**June**	917,462	1,676,749	2,408,353	2,380,601	
	**13,602,137**	**17,744,621**	**22,895,551**	**28,730,365**	

Useful Links:

i. Throughput to Abbot Point http://www.nqbp.com.au/ports-throughput/

"The Great Barrier Reef Intelligence Game, 2016"
Victoria Hemming, Mark Burgman, Terry Walshe. Anca Hanea.
School of Biosciences, University of Melbourne

1. Your estimate for Question 19

		Please enter your estimate (whole number)
i	Realistically, what do you think the **lowest** throughput (tonnes) from Abbot Point will be?	
ii	Realistically, what do you think the **highest** throughput (tonnes) from Abbot Point will be?	
iii	Realistically, what is your **best guess** for the throughput (tonnes) from Abbot Point?	
iv	**How confident are you** that your interval, from lowest to highest, could capture the throughput (tonnes) from Abbot Point? Please enter a number between 50 and 100%	%

2. Comments

Please enter any comments, additional knowledge or justification that you have about this question and /or your estimate. This will be shared with the group in Round 2.

3. Useful Links

Please enter any useful web links (URLs) you would like to contribute to your group

1		4	
2		5	
3		6	

Question 20 People Held in Nauru Regional Processing Centre, April 2016

"How many people will be held in the Republic of Nauru Regional Processing Centre in April 2016?"

Clarification: The Australian Government has established a regional processing centre in the Republic of Nauru which holds people "who have arrived without a visa, overstayed their visa or have had their visa cancelled". As at 31 January 2016 there were 484 people held in the Republic of Nauru Regional Processing Centre. This question seeks to understand how many people you believe will be recorded in the Republic of Nauru Regional Processing Centre as declared by the Australian Department of Immigration and Border Protection in their report for "Immigration Detention and Community Statistics Summary" for 30 April 2016.

Resolution: This question will be resolved by the Australian Department of Immigration and Border Protection in their report for "Immigration Detention and Community Statistics Summary for 30 April 2016".

Additional Information:

a. The last report, 31 January, 2016 declared there are **484 people** currently in the Republic of Nauru regional processing centre

Useful Links:

i. Immigration detention statistics http://www.border.gov.au/about/reports-publications/research-statistics/statistics/live-in-australia/immigration-detention

ii. About Immigration detention http://www.border.gov.au/Busi/Comp/Immigration-detention

1. Your estimate for Question 20

		Please enter your estimate (whole number)
i	Realistically, what do you think the **lowest** number of people will be?	
ii	Realistically, what do you think the **highest** number of people will be?	
iii	Realistically, what is your **best guess** for the number of people?	
iv	**How confident are you** that your interval, from lowest to highest, could capture the actual number of people? Please enter a number between 50 and 100%	%

2. Comments

Please enter any comments, additional knowledge or justification that you have about this question and /or your estimate. This will be shared with the group in Round 2.

3. Useful Links

Please enter any useful web links (URLs) you would like to contribute to your group

1		4	
2		5	
3		6	

Question 21 Launches to Space in May 2016

"How many space launches will take place in May 2016?"

Clarification: This question seeks to understand how many space launches you realistically think will take place between 1 May 2016 and 30 May 2016 across the globe. Space launches are most commonly satellites, however, can include the launch of supplies for space stations, science exploration and other reasons. For this question we will include anything considered a space launch by the "space launch report". We will include anything that is launched regardless of whether it subsequently fails or succeeds its intended mission.

Resolution: This question will be resolved by the space launch report

Additional Information:

a. Recent space launches for 2016

01/20/16, 04:01 UTC, PSLV-XL with IRNSS 1E from SR 2 to GTO-
01/27/16, 23:20 UTC, Ariane 5-ECA with Intelsat 29e from KO 3 to GTO
01/29/16, 22:20 UTC, Proton M/Briz M with Eutelsat 9B from TB 200/39 to GTO+
02/01/16, 07:29 UTC, CZ-3C/YZ-1 with Biedou M3-S from XC 2 to MEO
02/05/16, 13:38 UTC, Atlas 5 with GPS 2F-12 from CC 41 to MEO
02/07/16, 00:21 UTC, Soyuz 2-1b/Fregat with Glonass M from PL 43//4 to MEO
02/07/16, 00:30 UTC, Unha 3 with Kwangmyongsong 4 from SO to LEO
02/10/16, 11:40 UTC, Delta 4+5,4 with NROL-45 from VA 6 to LEO/R
02/16/16, 17:57 UTC, Rokot/Briz KM with Sentinel 3A from PL 133/3 to LEO/S
02/17/16, 08:45 UTC, H-2A-202 with ASTRO-H from TA Y1 to LEO

Useful Links:

i. The space launch report http://www.spacelaunchreport.com/

1. Your estimate for Question 21

		Please enter your estimate (whole number)	
i	Realistically, what do you think the **lowest** number of space launches will be?		
ii	Realistically, what do you think the **highest** number of spaces launches will be?		
iii	Realistically, what is your **best guess** for the number of space launches?		
iv	**How confident are you** that your interval, from lowest to highest, could capture the number of space launches? Please enter a number between 50 and 100%		%

2. Comments

Please enter any comments, additional knowledge or justification that you have about this question and /or your estimate. This will be shared with the group in Round 2.

3. Useful Links

Please enter any useful web links (URLs) you would like to contribute to your group

1		4	
2		5	
3		6	

This completes Round 1! WELL DONE!
Please make sure you have completed all of your estimates.
Save the form and submit!
hemmingv@student.unimelb.edu.au

Appendix A: Locations of LTMP monitoring.

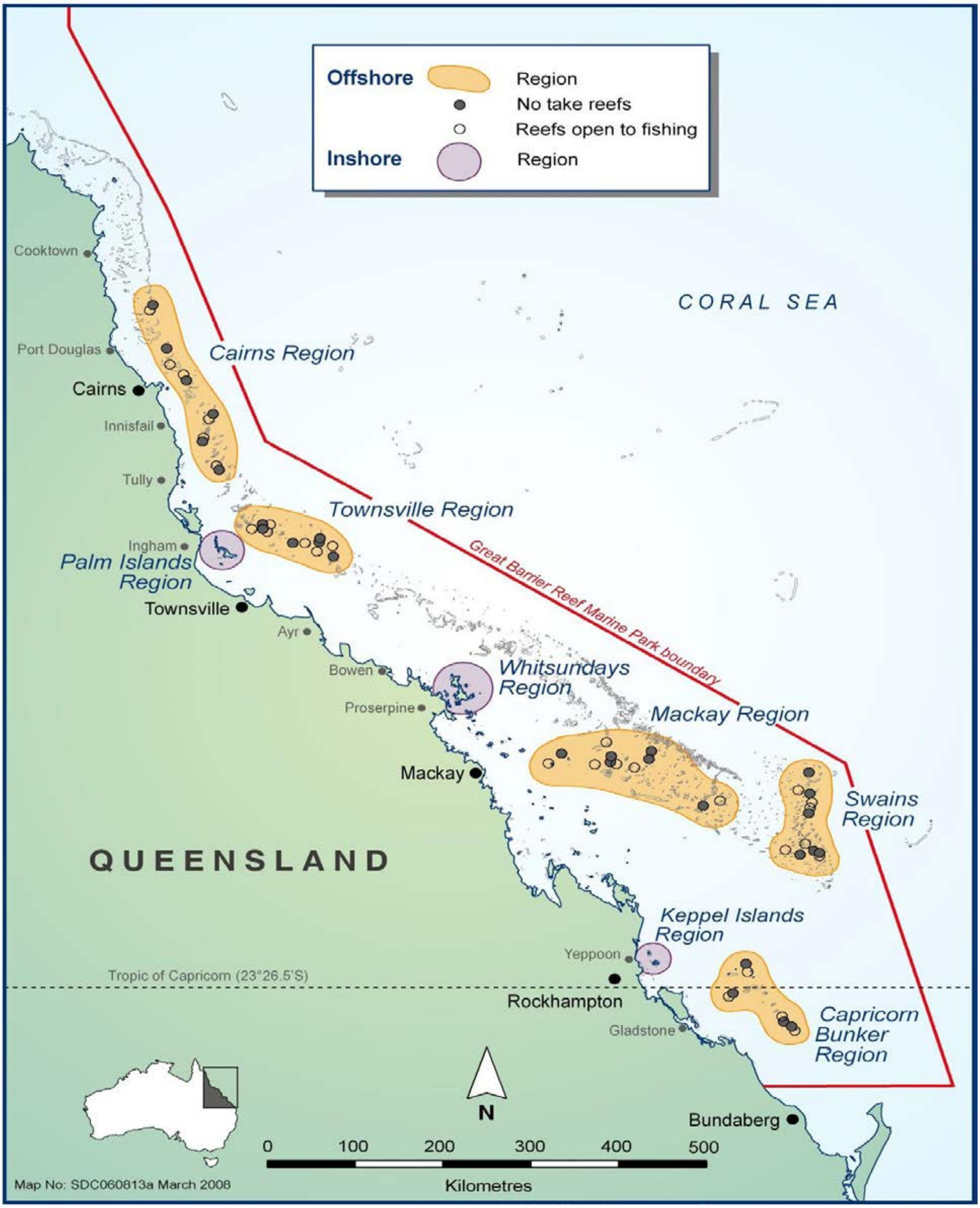

Note: The Pompey region is located adjacent to the Mackay region.

"The Great Barrier Reef Intelligence Game, 2016"
Victoria Hemming, Mark Burgman, Terry Walshe, Anca Hanea.
School of Biosciences, University of Melbourne

Responsible Researcher: Prof Mark Burgman, University of Melbourne, contact details omitted
Co-researcher: Victoria Hemming, University of Melbourne, contact details omitted
Co-researcher: Dr Terry Walshe, Australian Institute of Marine Sciences, contact details omitted
This research has been approved by the Human Ethics Committee of The University of Melbourne (HREC Project Number: 1546009.1). If you have any concerns about the conduct of this study that the researchers have not been able to answer to your satisfaction, you may contact the Executive Office, Human Research Ethics, The University of Melbourne, contact details omitted

Round 1 Feedback for your group: Group X

Code Name	

About this document

The following document provides feedback for you and a group 8-9 other people who participated in Round 1 of the Great Barrier Reef Intelligence Games. There are 8 groups in total (76 participants).

Instructions

This stage is a discussion phase and we believe it is one of the MOST critical parts of the method.

We would now like you to look over these preliminary results and compare your estimates with others in your group.

Please reflect on your initial judgements, and any comments provided by participants, and think about whether there might be reasons that you would need to update your judgements. Please try to consider counterfactuals that may make your revised estimate higher or lower than your first estimate.

Some things to be aware of

1. In order to provide group judgements we have standardised your confidence levels to 80%. This will mean your higher and lower estimates will appear higher or lower than your original estimates.
2. Some questions had maximum logical bounds (e.g. question 2). Your estimate was capped to be constrained within these logical bounds. Please check your initial estimates and make sure you have correctly interpreted the question.
3. I've done my best to generate the reports and analysis in R, however, I ran out of time to dynamically program feedback to the correct number of decimal places. Therefore everything is presented to 2 decimal places.
4. A few questions have variations over an order of magnitude, this might be information you possess, or it could be a mistake, if you notice you are an outlier could you please use the comments section to clarify.

Adding comments

1. Use this form: Spaces have been provided to add additional comments and links. You will need to save this form and return to Victoria Hemming. She will collate the additional comments and links and circulate them to your group through the discussion phase.
2. Email: Any comments and additional links can be provided to Victoria Hemming who will collate additional information for your group and circulate it on a daily basis.

The discussion phase will finish on 9am Wednesday 23 March, 2016 (Melbourne time).

Revising your estimates from Round 1

A form will be sent through on Wednesday 23rd for you to revise your estimates.

Due to the delay in feedback we have extended the time for you to revise your deadline and now

Round 2 estimates will be due 1pm Wednesday 30th March, 2016.

Code names removed

Table of Contents

Questions

1	*Density of Crown of Thorns Starfish (Acanthaster planci)*	3
2	*Coral Bleaching across the Great Barrier Reef*	5
3	*Asian Green Mussel Detections in Queensland*	7
4	*Prevalence of White Syndrome Coral Disease on Reef 21060*	9
5	*Commercial Catch of Coral Trout*	11
6	*Marine Turtles*	13
7	*Shark Control in the Mackay Region*	15
8	*Water Temperature in the Southern Great Barrier Reef*	17
9	*Discharge volume (Mega litres) from the Burdekin River*	19
10	*Chlorophyll Levels Detected at Pine Island*	21
11	*Wind Speed at Davies Reef*	23
12	*Average Maximum Air Temperature Hamilton Island*	25
13	*Turbidity in the Wet Tropics*	27
14	*El Nino Events*	29
15	*The Spread of Zika Virus Throughout the European Union*	31
16	*The Price of Gold*	33
17	*The UK Referendum*	35
18	*The Stock Price of Twitter*	37
19	*The Throughput (tonnes) from Abbot Point Port.*	39
20	*People Held in Nauru Regional Processing Centre*	41
21	*Launches to Space in May 2016*	43
	Ethics	45

Code names removed

Question 1 Density of Crown of Thorns Starfish (*Acanthaster planci*)

"What will be the average density of Crown of Thorns Starfish (Acanthaster planci) *detected per 2 minute manta-tow at Rib Reef, in the Townsville region, as surveyed by the Australian Institute of Marine Science (AIMS) as part of the Long-term Monitoring Program between 1 March, 2016 and 30 June, 2016 (inclusive)?"*

Clarification: Crown of Thorns Starfish *(Acanthaster planci) (* CoTS) are found at numerous coral reef ecosystems, including the Great Barrier Reef. They consume hard corals, and are the focus of manta-tow surveys undertaken by AIMS as part of the Long Term Monitoring Program (LTMP).

This question relates specifically to the average density of CoTS per two minute manta-tow that will be detected by AIMS during surveys at Rib Reef between 1 March 2016 and 30 June 2016 (inclusive). Rib Reef is located in the Townsville region of the Great Barrier Reef (GBR) (Appendix A). The average density per 2 minute manta tow, is a standard metric used to compare between reefs and years. The average density of CoTS per 2 minute manta tow refers to the total number of CoTS that are detected by AIMS during manta-tow surveys, divided by the total number of manta-tow surveys undertaken at Rib Reef. We will accept survey results for Rib Reef recorded between 1 March, 2016 and 30 June, 2016 (inclusive). If the survey does not occur, or occurs outside of this period, the question will be void. As with all monitoring data, it is important to note that this question relates specifically to the number of CoTS detected and reported, not necessarily the actual number of CoTS present at Rib Reef.

Resolution: The question will be resolved when the report for the Townsville section for the 2015/2016 monitoring period is published online by AIMS (see latest surveys in useful links).

Additional Information:

a. Historical density of CoTS per 2 minute manta tow at Rib Reef recorded by the AIMS LTMP

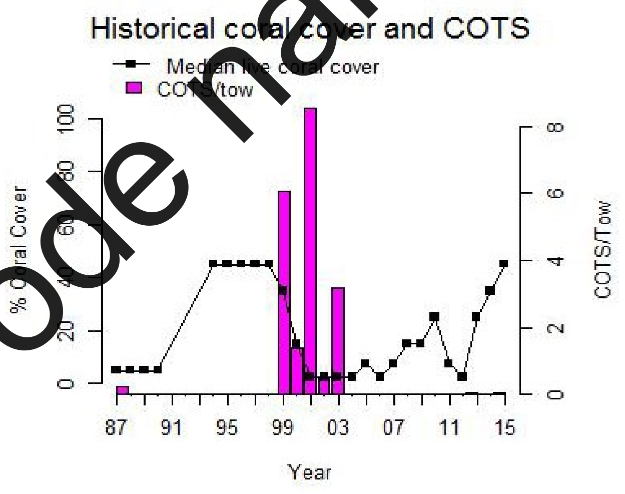

Useful Links:

i.	Rib Reef	http://data.aims.gov.au/reefpage2/reefpage.jsp?fullReefID=18032S
ii.	Latest surveys	http://www.aims.gov.au/docs/research/monitoring/reef/latest-surveys.html
iii.	Survey methods	http://www.aims.gov.au/documents/30301/20e3bf4f-4b3b-4808-ac02-c15c2912c3f2
iv.	Map of LTMP regions	Appendix A

Code names removed

Question 1 Results

Your estimate compared to your group (# Confidence intervals have been standardized to 80%)

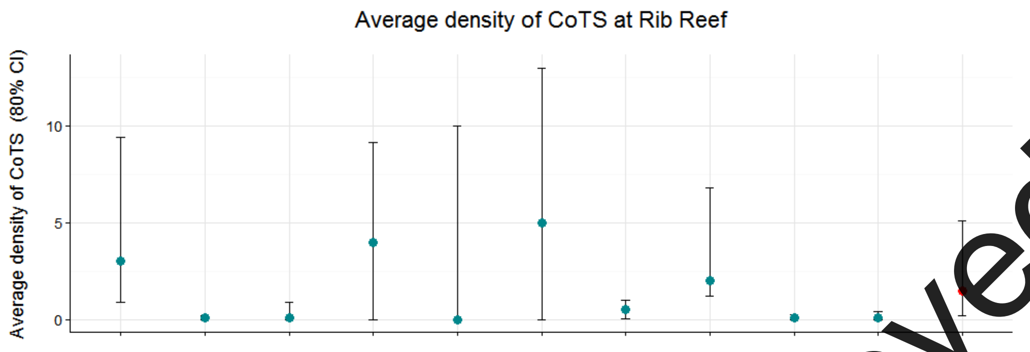

Best	3.00	0.08	0.10	4.00	0.00	5.00	0.50	2.00	0.08	0.10	1.49
Low	0.87	0.00	0.00	0.00	0.00	0.00	0.05	1.20	0.00	0.00	0.21
High	9.40	0.18	0.90	9.14	10.00	13.00	1.00	6.80	0.24	0.44	5.11

Code names removed

Comments and questions

Name	Comments
	Looked on AIMS COTS page at average density time series, took into consideration 2015 outbreak status at Rib Reef, looked at RR broadscale survey result figures, guessed avg density and #tows was likely to be roughly the same as 2015, guessed 1 COTS in 4 month period @ ~ 3 tows per month x 4 mos
	examined the latest surveys document (link provided) and looked at COT 'outbreak' status for reefs below and above Rib reef. I also looked at what 'active' or 'incipient' outbreak seemed to imply in terms of COTs/tow and historical trends. It seemed plausible that an outbreak of up to 1 CoTS/tow could occur without any detection in the previous
	I have analysed data on CoTS distributions during the current outbreak and have not heard reports of CoTS outbreaks in the Townsville sector, making their absence on Rib this year more likely than an outbreak
	Historical average densities range between 0 and 1 for the reef as a whole although this is spatially variable. No COTS were detected in 2015 in this region, but some evidence (feeding scars) recorded of their presence. One detected in 2014 and none in 2013. Evidence would suggest they are present, but in low numbers. No evidence of widespread outbreaks elsewhere, although a few isolated areas of outbreaks
Facilitator	Some substantial comments here. Good to see your reasoning. Does anyone have any knowledge of the current extent of CoTS?
Comments?	

Additional Links

#	links
1	http://data.aims.gov.au/waCOTSPage/cotspage.jsp
2	http://www.aims.gov.au/web/guest/reef-monitoring/cairns-and-innisfail-and-townsville-sectors-2014
Links	

Question 2 Coral Bleaching across the Great Barrier Reef

"How many of the 24 reefs listed in Table 1 below will be reported with at least 1% bleaching of hard corals by the Australian Institute of Marine Science (AIMS) during SCUBA surveys undertaken between 1 March, 2016 and 30 June 2016 as part of the Long Term Monitoring Program (LTMP)?"

Clarification: This question asks how many of the 24 reefs listed in Table i you believe will realistically be recorded as having at least 1% coral bleaching when surveyed by SCUBA surveys between 1 March 2016 and 30 June 2016 (inclusive) by AIMS as part of the LTMP. The 24 reefs listed in Table 1 are located in two regions of the Great Barrier Reef: the Townsville Region, and the Mackay / Pompey region (Appendix A). For each SCUBA search the percentage of hard coral cover which is bleached white, or near white or has a lurid appearance is recorded according to the categories in Table 2. Only reefs listed in Table 1 which are surveyed between 1 March 2016 and 30 June 2016 (inclusive) will be included in the calculation. If none of the 24 reefs are surveyed then the question will be voided.

Resolution: The question will be resolved when by the AIMS LTMP program by 1 September, 2016.

Additional Information:

a. Table i: List of reefs scheduled for survey between 1 June 2016 and 30 June 2016

REEF_ID	REEF_NAME	RAP_REGION	REEF_ID	REEF_NAME	RAP_REGION
21060	21060S	Mackay / Pompey	18030	KELSO REEF	Townsville
20351	POMPEY REEF (NO 1)	Mackay / Pompey	18042	ROXBURGH REEF	Townsville
21591	21591S	Mackay / Pompey	18076	HELIX REEF	Townsville
20351	POMPEY REEF (NO 2)	Mackay / Pompey	18032	RIB REEF	Townsville
20348	20348S	Mackay / Pompey	18043	FORE AND AFT REEF	Townsville
21062	21062S	Mackay / Pompey	18031	LITTLE KELSO REEF	Townsville
20353	20353S	Mackay / Pompey	18083	FORK REEF	Townsville
21064	21064S	Mackay / Pompey	18077	GRUB REEF(18077)	Townsville
21139	21139S	Mackay / Pompey	18086	CHICKEN REEF	Townsville
21187	21187S	Mackay / Pompey	18081	KNIFE REEF	Townsville
21025	PENRITH REEF	Mackay / Pompey	18088	CENTIPEDE REEF	Townsville
20309	TERN REEF(20309)	Mackay / Pompey	18091	LYNCHS REEF	Townsville

b. Coral bleaching categories : sources AIMS survey procedures

# Please note this table confused a lot of people. To clarify, it shows the 8 categories used by AIMS when assessing the level of bleaching to hard coral. It was split into two columns to fit on the page.

% COVER OF BLEACHING FOR HARD CORALS	
0%	10-30%
0+ Individual colonies (>1% total hard coral cover)	30-50%
1-5%	50-75%
5-10%	75-100%

Useful Links:

i.	Coral reef bleaching	http://www.gbrmpa.gov.au/managing-the-reef/threats-to-the-reef/climate-change/what-does-this-mean-for-species/corals/what-is-coral-bleaching
ii.	AIMS Survey procedure	http://www.aims.gov.au/documents/30301/20e3bf4f-4b3b-4808-ac02-c15c2912c3f2
iii.	Reefs under survey	http://data.aims.gov.au/reefpage2/allreefs.jsp
iv.	LTMP map	Appendix A

Code names removed

Question 2 Results

Your estimate compared to your group (# Confidence intervals have been standardized to 80% , Maximum capped at 24 reefs).

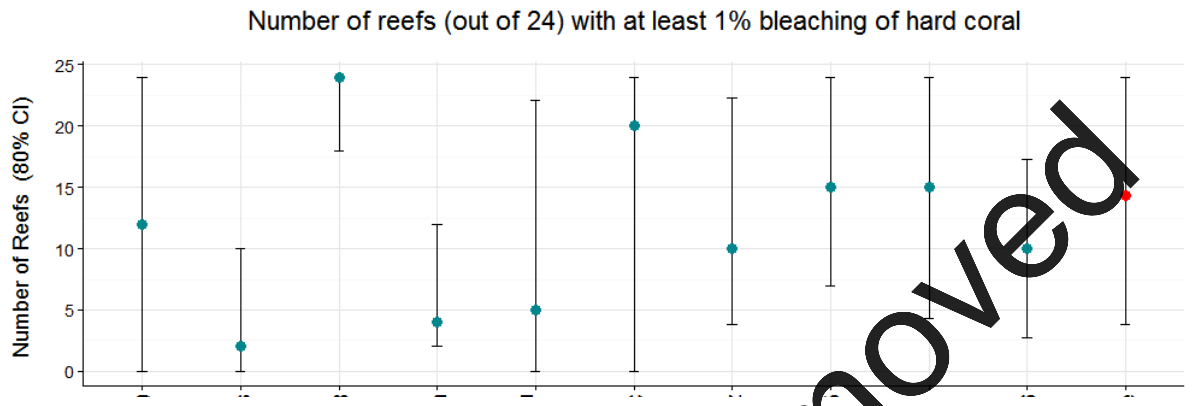

Best	12.00	2.00	50.00	4.00	5.00	20.00	10.00	15.00	15.00	10.00	14.30
Low	0.00	0.00	18.00	2.00	0.00	0.00	3.85	7.00	4.33	2.73	3.79
High	25.33	10.00	76.67	12.00	22.14	26.40	22.31	39.00	24.60	17.27	27.57

Comments and questions

Name	Comments
	I'm not sure what the current bleaching records for these reefs indicate, so my lower bound is not really informed by data. My upper bounds are informed by past bleaching events, and current media<br>around a high likelihood for another mass bleaching event.
	Not really sure about this one.
Facilitator	Seems to be a lot of variation about this one. Does anyone have anything to add about recent bleaching events on the reef, and whether they would affect any of these reefs? Or whether these reefs have residual bleaching?
**Comments?**	

Additional Links

#	links
1	http://statements.qld.gov.au/Statement/2016/3/1/new-reports-show-lowlevel-coral-bleaching-on-great-barrier-reef
2	http://www.smh.com.au/federal-politics/political-news/next-few-weeks-critical-as-great-barrier-reef-suffers-tragic-coral-bleaching-event-20160301-gn7l0f.html
3	http://www.aims.gov.au/docs/research/climate-change/coral-bleaching/bleaching-events.html
**Links?**	

Code names removed

Question 3 Asian Green Mussel Detections in Queensland

"How many unique detections of Asian Green Mussel (Perna viridis) *will be recorded by the Queensland Department of Agriculture and Fisheries between 1 March 2016 and 30 June 2016".*

Clarification: *Asian Green Mussel (Perna viridis) is an invasive species which can be carried in ballast water. The Queensland Department of Agriculture and Fisheries (DAF) has been collecting records of Asian Green Mussel detections in Queensland since 2001. Reports of detections are generally submitted to the DAF by people involved in routine slipping of ships and vessels at Queensland Ports. For this question we want you to estimate how many unique detections of AGM will be reported and subsequently recorded by DAF between 1 March 2016 and 30 June 2016 (inclusive). For this question we are not interested in whether the pest subsequently establishes simply that it is detected and recorded by DAF. Note that multiple reports to DAF of the same incident will only count as one record. Also the detection in not made in Queensland, then it will not count (e.g. if a vessel has visited QLD but the detection was not made until it moved to another state or country).*

Resolution: *The question will be resolved by Queensland Department of Agriculture and Fisheries on 15 July 2016.*

Additional Information:

*a. Since 2001 there have been **10 reports** of Asian Green Mussels recorded by the Queensland Department of Agriculture and Fisheries.*

Code names removed

Useful Links:

i. AGM Fact sheet https://www.daf.qld.gov.au/plants/weeds-pest-animals-ants/legislation-policies-permits/legislation/faqs/asian-mussels

"The Great Barrier Reef Intelligence Game, 2016"
Victoria Hemming, Mark Burgman, Terry Walshe. Anca Hanea.
School of Biosciences, University of Melbourne

Question 3 Results

Your estimate compared to your group (# Confidence intervals have been standardized to 80%)

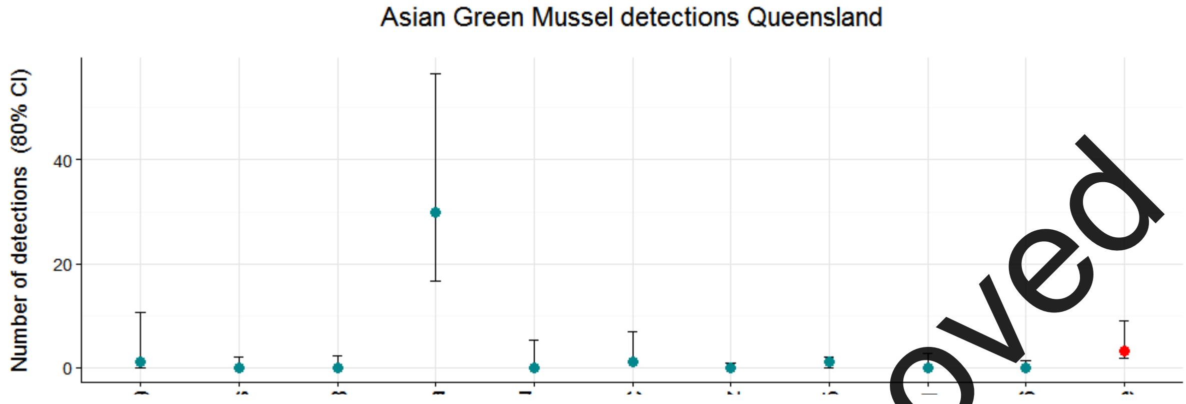

Best	1.00	0.00	0.00	30.00	0.00	1.00	0.00	1.00	0.00	0.00	3.30
Low	0.00	0.00	0.00	16.67	0.00	1.00	0.00	0.00	0.00	0.00	1.77
High	10.60	2.00	2.29	56.67	5.33	7.00	0.80	2.07	2.67	1.33	9.08

Comments and questions

Name	Comments
	I couldn't find any websites recording new sightings of the mussel, apart from a 2011 outbreak in WA. However, I am not sure what would trigger a media alert.
Facilitator	Most people appear to believe the number of detections will remain low. Is there any reason to think that the current status quo could change in the coming months? Participant 4 can you tell us about your estimate?
Comments?	

Additional Links

#	links
1	http://www.pbcrc.com.au/publications/pbcrc1403
Links?	

Code names removed

“The Great Barrier Reef Intelligence Game, 2016”
Victoria Hemming, Mark Burgman, Terry Walshe. Anca Hanea.
School of Biosciences, University of Melbourne

Question 4 Prevalence of White Syndrome Coral Disease on Reef 21060

“What will be the total number of coral colonies reported with White Syndrome (a coral disease) on Reef 21060 in the Mackay-Pompey Region, by the Australian Institute of Marine Science (AIMS) during SCUBA surveys undertaken between 1 March and 30 June, 2016?”

Clarification: White Syndrome is a coral disease present on the Great Barrier Reef. This question aims to determine the extent to which Reef 21060 will be affected by White Syndrome when next surveyed by AIMS in 2016. Surveys for White Syndrome are undertaken by AIM as part of the Long Term Monitoring Porgram, using SCUBA searches along fixed transects. During SCUBA surveys a 2 m belt (1 m either side of the central tape measure) is visually searched along 50 m fixed line transects, and the number of coral colonies which are detected to have White Syndrome are recorded. Reef 21060 is located in the Mackay Pompey Region, and is currently scheduled to be surveyed in March, 2016, however, the survey realistically may take place anytime between 1 March, 2016 and 30 June , 2016. During these surveys the total number of coral colonies with signs of White Syndrome will be recorded.

Resolution: The answer to this question will be resolved when AIMS publishes data for Reef 21060 as part of their reporting for the Mackay / Pompey region for the 2015 / 2016 financial year. See "previous reports" in useful links.

Additional Information:

a. Previous trends in White Syndrome for Reef 21060 as detected by AIMS during SCUBA searches.

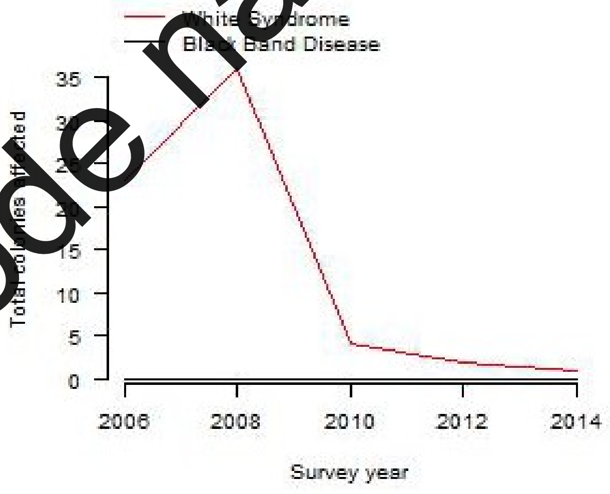

Code names removed

Useful Links:

i. Previous data for Reef 21060 http://data.aims.gov.au/reefpage2/rpdetail.jsp?fullReefID=21060S&sampleType=VPOINT

ii. Previous reports for the LTMP http://www.aims.gov.au/docs/research/monitoring/reef/latest-surveys.html

iii. Survey procedure http://www.aims.gov.au/documents/30301/20e3bf4f-4b3b-4808-ac02-c15c2912c3f2

Question 4 Results

Your estimate compared to your group (# Confidence intervals have been standardized to 80%)

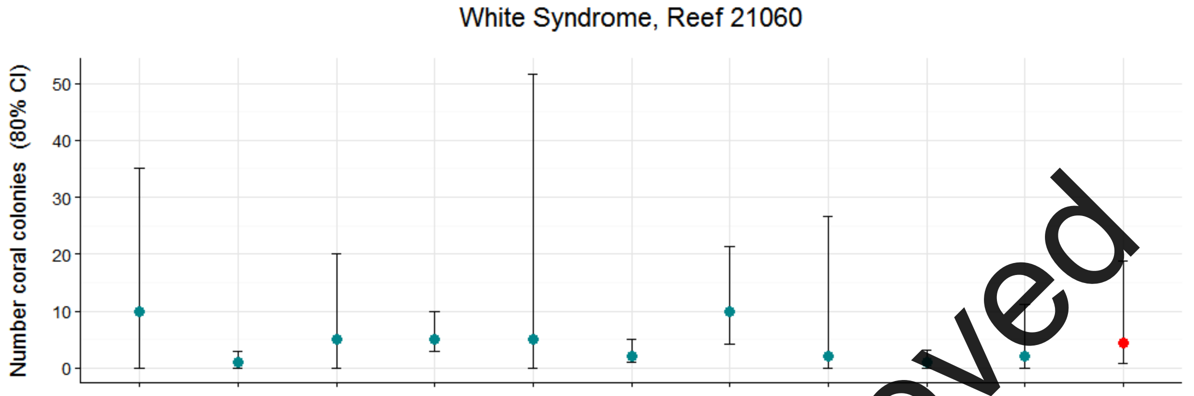

Best	10.00	1.00	5.00	5.00	5.00	2.00	10.00	2.00	1.00	2.00	4.30
Low	0.00	0.00	0.00	3.00	0.00	1.00	4.29	0.00	0.00	0.00	0.83
High	35.00	3.00	20.00	10.00	51.67	5.00	21.43	26.53	3.13	11.14	18.69

Code names removed

Comments and questions

Name	Comments
	Potential links to high sea temperatures (see link 1 below). However, during last outbreak (2008) the Mackay reefs were not really affected by white syndrome (scattered colonies on back reef only). Coral cover on these reefs is under 10% due to COT (http://data.aims.gov.au/waAesthetics/servaesthetics?fullReefID=21060S&zone=4) but I'm unsure
	To answer this more accurately I would need to know the average number of colonies per transect.
	These are not diseases, rather clinical signs. Their expression is dependent on the causative agent which has not been identified, therefore prediction of prevalence is completely guesswork.
	White syndrome rare for the site. Low numbers (<5 colonies) in past 4 years.
Facilitator	Some good comments here. The group average appears to indicate that the recorded prevalence will remain much the same as the last survey period. Is there any reason to believe it might be substantially more?
**Comments?**	

Additional Links

#	links
1	http://data.aims.gov.au/waAesthetics/servaesthetics?fullReefID=21060S&zone=4
2	http://www.ncbi.nlm.nih.gov/pmc/articles/PMC1865566/
3	http://www.aims.gov.au/documents/30301/e055df14-24d0-40ca-b46d-4ae1988da07c
4	http://data.aims.gov.au/waAesthetics/servaesthetics?fullReefID=21060S&zone=4
**Additional links?**	

Question 5 Commercial Catch of Coral Trout

"How many tonnes of Coral Trout will be caught in Queensland by the Commercial Line Fishery in April 2016?"

Clarification: Coral Trout is a species which is commercially caught by the Coral Sea Fishery in Queensland. Each month the tonnage of catch is reported to Fisheries Queensland, who publicly report this quantity through their QFISH database. This question asks how many tonnes of Coral Trout will be reported to be caught from 1 April 2016 to 30 April 2016. Only the tonnes of Coral Trout which are caught by the commercial line fishery and reported to Fisheries Queensland and subsequently reported on the QFISH database are included in the estimate.

Resolution: This question will be resolved by QFISH an online database hosted by Fisheries Queensland on 1 September 2016. The following options will be selected: Logbook type: "Commercial", Fishing Method "Line", Species Group: "Coral Trout", and then only selecting "Coral Trout", Calendar Year "2016", Month " 04_April". Weight (t).

Additional Information:

a. Previous harvest of Coral Trout for the Commercial Line Fishery from QFISH.

Calendar Year	Month	Licences	Weight (t)
2012	04 - April	86	70.53
2013	04 - April	93	69.23
2014	04 - April	77	53.92

Code names removed

Useful Links:

i. QFISH http://qfish.fisheries.qld.gov.au/Query/ViewResults?CubeId=7&PredefinedQueryId=cbb0d21f-ed24-4fb7-b0b4-d7422c561e9b&ViewKind=Pivot

"The Great Barrier Reef Intelligence Game, 2016"
Victoria Hemming, Mark Burgman, Terry Walshe. Anca Hanea.
School of Biosciences, University of Melbourne

Question 5 Results

Your estimate compared to your group (# Confidence intervals have been standardized to 80%)

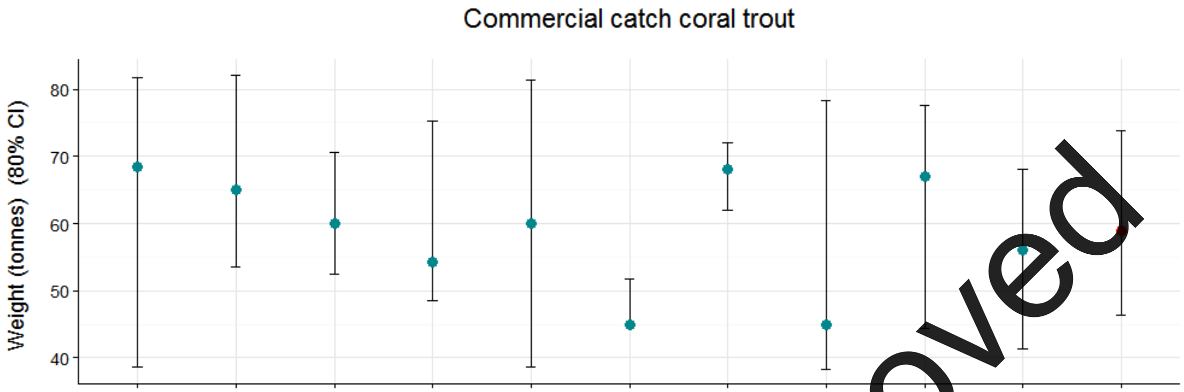

Best	68.46	65.00	60.00	54.20	60.00	45.00	68.00	45.00	67.00	56.00	58.87
Low	38.64	53.57	52.53	48.60	38.67	45.00	62.00	38.33	44.33	41.33	46.30
High	81.71	82.14	70.67	75.27	81.33	51.67	72.00	78.33	77.67	68.00	73.88

Comments and questions

Name	Comments
	It is dependent on the number of licenses active. Based on the reduction in licenses from 2012 to 2013 and similar catches the reduction in in licenses in 2014 and a proportionate reduction in catches means that there should have been less licenses issued in years after 2014. I made the assumption that there were approx. 60 licenses in 2016.
Facilitator	Some fluctuation about the best guesses here, as well as some large upper and lower intervals. Is anyone able to elaborate?
**Comments?**	

Additional Links

#	links
1	http://qfish.fisheries.qld.gov.au/Query/ViewResults?CubeId=7&PredefinedQueryId=cbb0d21f-ed24-4fb7-b0b4-d7422c561e9b&ViewKind=Pivot
**Links?**	

Code names removed

“The Great Barrier Reef Intelligence Game, 2016”
Victoria Hemming, Mark Burgman, Terry Walshe. Anca Hanea.
School of Biosciences, University of Melbourne

Question 6 Marine Turtles

“How many turtles will be reported and confirmed as stranded by the Queensland Department of Environment and Heritage Protection for the whole of the Queensland East Coast between 1 January 2016 to 31 March 2016?”

Clarification: Each year the number of marine turtles which end up stranded on Queensland’s east coast are recorded by the Queensland Department of Environment and Heritage Protection in their strandings database. The strandings database includes sick, injured, debilitated or dead marine turtles. The East Coast of Queensland refers to entire eastern coastline of Queensland adjacent to the Coral Sea. This question refers to the number of marine turtles which will be found, and reported to the Queensland Department of Environment and Heritage Protection as stranded, and subsequently confirmed to be a stranded turtle, for the period between 1 January and 31 March 2016.

Resolution: The question will be resolved by the Queensland Department of Environment and Heritage Protection on their strandings database for “Turtle Strandings for the period of 1 January to 31 March 2016” at some time before 1 September 2016.

Additional Information:

a. Turtle strandings for the period 1 January to 31 March 2015 for previous years

Location	2015	2014	2013	2012	2011	2010	2009
Total for east coast of Queensland	92	217	192	251	182	166	177

Code names removed

Useful Links:

i. Marine turtle strandings https://www.ehp.qld.gov.au/wildlife/caring-for-wildlife/marine-strandings-update.html

ii. Marine Turtle report for 2011 https://www.ehp.qld.gov.au/wildlife/caring-for-wildlife/pdfs/turtle-report-2011.pdf

Question 6 Results

Your estimate compared to your group (# Confidence intervals have been standardized to 80%)

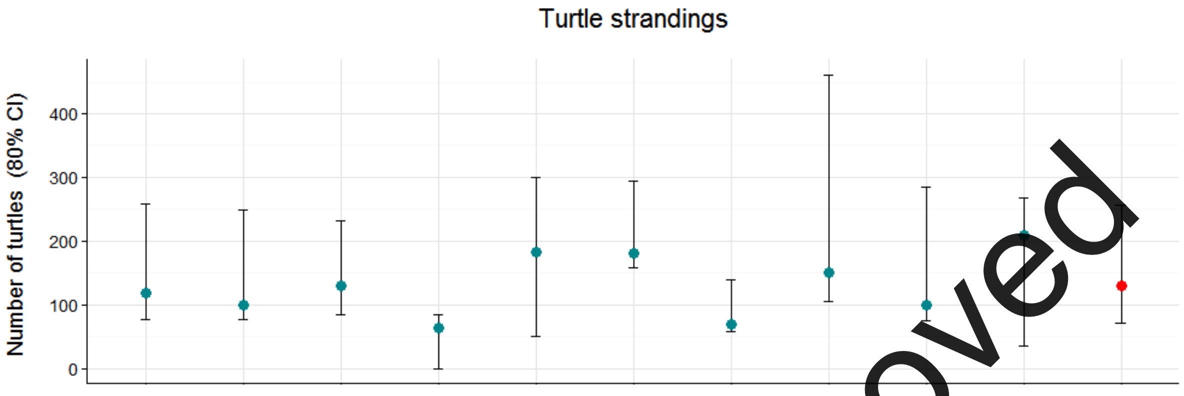

Best	118.00	100.00	130.00	63.00	182.00	180.00	70.00	150.00	100.00	210.00	130.30
Low	77.47	77.14	84.29	0.00	50.00	157.60	58.57	105.56	75.38	35.45	72.15
High	258.80	248.57	232.86	83.92	300.00	293.60	138.57	461.11	284.62	268.18	257.02

Comments and questions

Name	Comments
	Extreme conditions (i.e. water temp, pollution etc) leading to decline in seagrass condition could be a factor, but I anticipate there might be a lag
	Damn turtles
	There is data missing for the 2 most recent years so then estimates may not reflect the true average stranding.
Facilitator	Does anyone have a feeling whether the past three months have been particularly good or bad for turtle strandings?
**Comments?**	

Additional Links

#	links
1	http://www.hindawi.com/journals/jmb/2015/848923/
2	http://www.abc.net.au/news/2014-10-22/study-looks-into-great-barrier-reef-turtle-strandings/5833588
3	http://www.gbrmpa.gov.au/managing-the-reef/threats-to-the-reef/extreme-weather/ecosystem-impacts
**Links?**	

Code names removed

"The Great Barrier Reef Intelligence Game, 2016"
Victoria Hemming, Mark Burgman, Terry Walshe. Anca Hanea.
School of Biosciences, University of Melbourne

Question 7 Shark Control in the Mackay region

"How many individual sharks (target species only) will be caught by the Queensland shark control program in May 2016?"

Clarification: The Queensland Shark Control Program has been operating since 2001, however, nets and drumlines have been in place in the Mackay region since 1963. The nets and drumlines in the Mackay region are checked every second day weather permitting, whereupon the number of target and non-target species caught in the nets and drumlines is recorded. This question seeks your opinion on what you realistically think the total number of target sharks caught in the Mackay region will be in May 2016? Target shark species are mainly Tiger Sharks, Bull Sharks and Whalers, but include most shark species (see Target Species in useful links). All sharks whether dead or alive are recorded. However, in order to be included the Queensland Shark Control Program would need to detect the shark in the drumlines or nets and subsequently identify it. Note that this question is asking for the total number of individuals rather than the number of different species. Only sharks which are detected during surveys between the 1 May 2016 and the 31 May 2016 will be included in the count.

Resolution: This question will be resolved when the Queensland Government release their Shark Control Program Shark Catch Statistics on their website (see useful links).

Additional Information:

a. Previous number of sharks caught for the Mackay area during the month of May 2001-2015

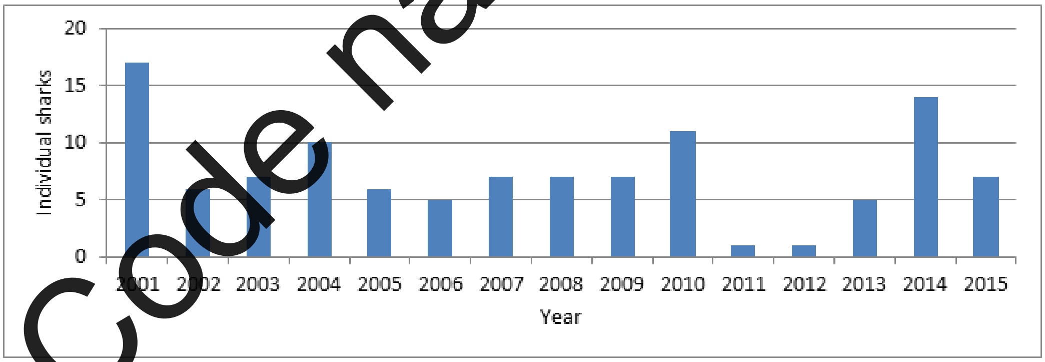

Code names removed

Useful Links:

i. Location of shark nets — https://www.daf.qld.gov.au/ data/assets/pdf_file/0005/69566/scp-equip-location-mackay-edit.pdf
ii. About the shark program — https://www.daf.qld.gov.au/fisheries/services/shark-control-program
iii. Previous data — https://www.daf.qld.gov.au/fisheries/services/shark-control-program/catch-numbers
iv. Target species, Appendix C — https://www.daf.qld.gov.au/ data/assets/pdf_file/0007/310939/public-information-package-shark-control-program.pdf

Question 7 Results

Your estimate compared to your group (# Confidence intervals have been standardized to 80%)

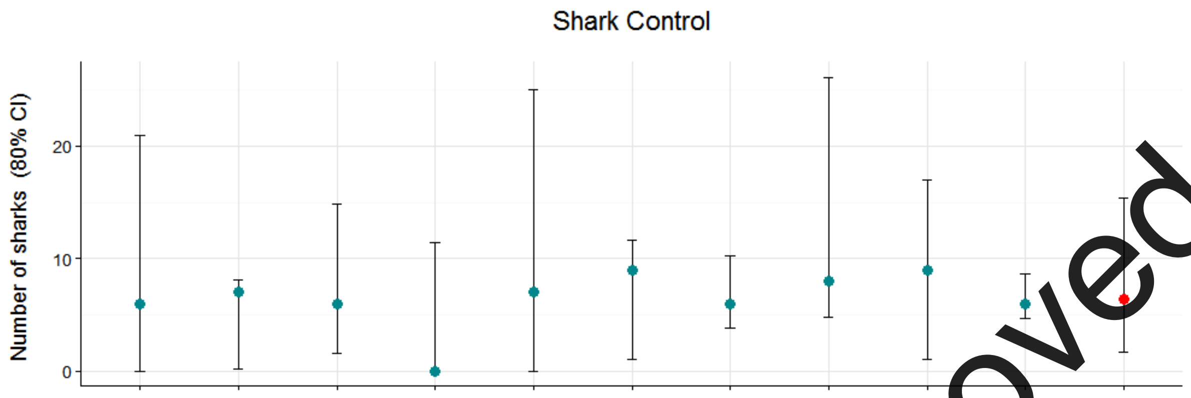

Best	6.00	7.00	6.00	0.00	7.00	9.00	6.00	8.00	9.00	6.00	6.40
Low	0.00	0.14	1.56	0.00	0.00	1.00	3.87	4.80	1.00	4.67	1.70
High	20.93	8.14	14.89	11.43	25.00	11.67	10.27	26.13	17.00	8.67	15.41

Comments and questions

Name	Comments
Facilitator	Best guesses are consistent with previous years. Is there any reason to believe the number of sharks caught will be higher or lower than average?
Comments?	

Additional Links

#	links
Links?	

.

Code names removed

Question 8 Water Temperature in the Southern Great Barrier Reef

"How many days in April 2016 will the maximum water temperature reach 28.0° Celsius or above at Heron Island?"

Clarification: This question refers specifically to the water temperature (Celsius) recorded at Heron Island by "Heron Island Sensor Float 1", which is positioned 0.3 metres below the sea-surface, between 1st April 2016 and 30th April 2016 (inclusive). If the weather station fails to record a reading for a day it will not be included in the maximum. If no readings are recorded by the Heron Island Sensor Float 1, between 1 April 2016 and 30 April 2016 then the question will be void.

Resolution: The data to resolve this question will be found on the historic data tool on the AIMS website (see useful links), for "Heron Island Sensor Float 1", and selecting the daily maximum for April 2016 (from the 1 – 30 April inclusive). The data will be obtained from a Rule-Based Quality Control data set which aims to avoid illogical values.

Additional Information:

a. Daily maximum water temperature recorded by Sensor Float 1 at Heron Island in Apirl 2015, from the AIMS weather station.

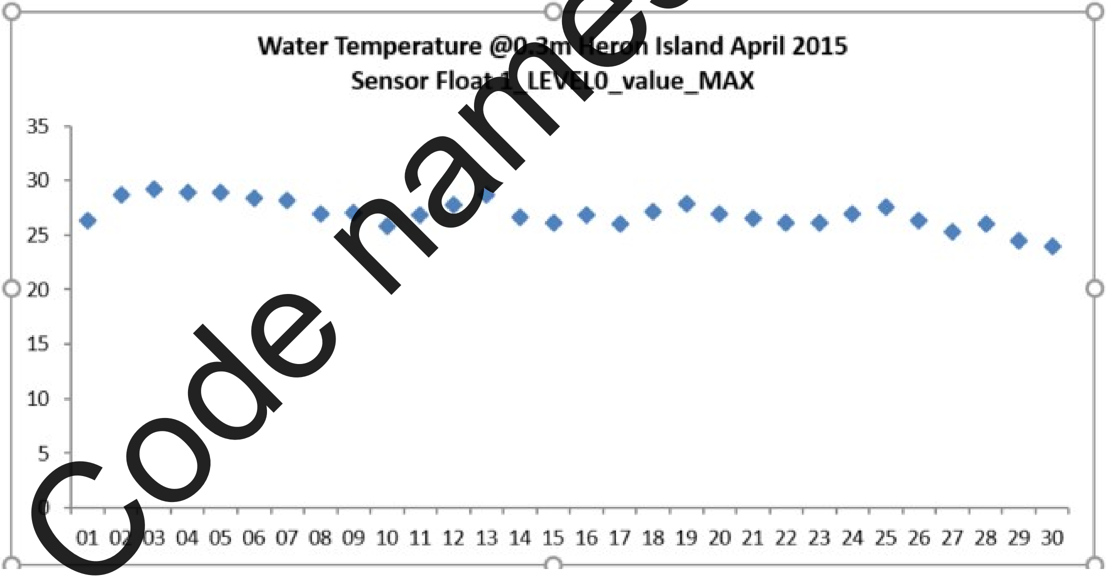

Code names removed

Useful Links:

i. Daily readings — http://weather.aims.gov.au/#/station/130

ii. Heron Island — http://maps.aims.gov.au/index.html?intro=false&z=11&ll=151.98%2C-23.45&l0=ea_World_NE2-coast-cities-reefs_Baselayer%2Caims_aims%3AWeatherStation

iii. Long term dataset — http://data.aims.gov.au/aimsrtds/datatool.xhtml?from=2011-02-05&thru=2016-02-06&period=DAY&aggregations=MAX&channels=19

Question 8 Results

Your estimate compared to your group (# Confidence intervals have been standardized to 80%, Maximum capped to 30 days of April).

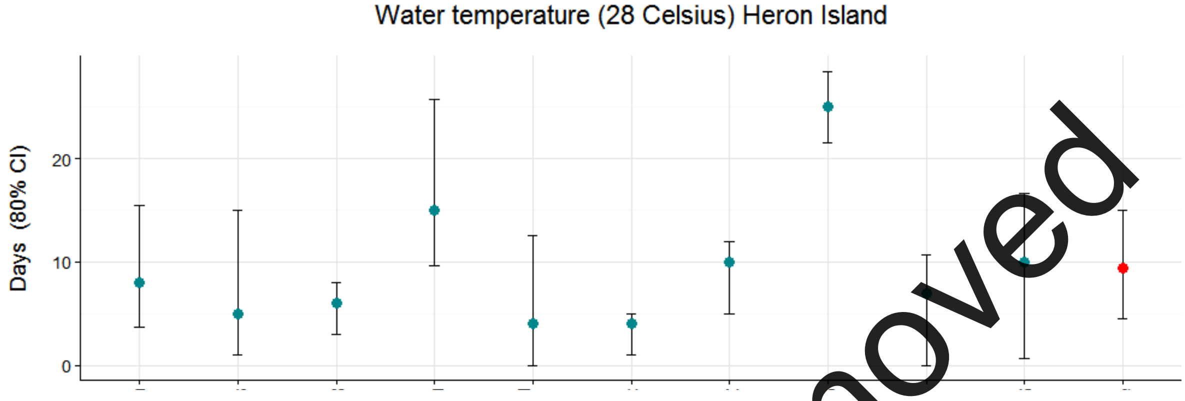

Best	8.00	5.00	6.00	15.00	4.00	4.00	10.00	25.00	7.00	10.00	9.40
Low	3.73	1.00	3.00	9.67	0.00	1.00	5.00	21.57	0.00	0.67	4.56
High	15.47	15.00	8.00	25.67	12.53	5.00	12.00	28.43	10.69	16.67	14.95

Comments and questions

Name	Comments
	Suggestions this year will have higher recorded sea temps (see previous reports)
Facilitator	Some variation here in estimates. MF75 and KBGC I’m keen to hear from you.
Comments?	

Additional Links

#	links
1	http://www.bom.gov.au/marinewaterquality/
2	http://data.aims.gov.au/aimsrtds/coralbleaching.xhtml
Links	

Code names removed

Question 9 Discharge Volume (Megalitres) from the Burdekin River

"What will be the total discharge volume (Megalitres) for the Burdekin River, Queensland in April 2016?"

Clarification: This question relates specifically to the "Stream Discharge Volume (Megalitres)" recorded ONLY at the monitoring station "120006B Burdekin River at Clare in the Burdekin region" from 1 April to 30 April 2016 (inclusive) as reported on the Water Monitoring Information Portal hosted by the Queensland Government.

Resolution: This question will be resolved on 15 May 2016 by going to the "120006B Burdekin River at Clare" station (refer to useful links), and selecting "Stream Discharge Volume" (Megalitres), and selecting "Custom" for the "Period" column, "Download" for the "Output" column and "Daily" for the "Data Interval" column. The start date will be entered as 00:01_01/04/2016, and the end date will be entered as 00:00_30/04/2016. The sum of all of the daily totals will be used to calculate total discharge for the month.

Additional Information:

a. Total volume of discharge in April 2015 = **64,685 Megalitres**

b. Cumulative volume discharge (Megalitres) at the Burdekin river during April 2015. *# I included this graph as it was output from the monitoring site, but I agree it is not very helpful and may be unnecessary information.*

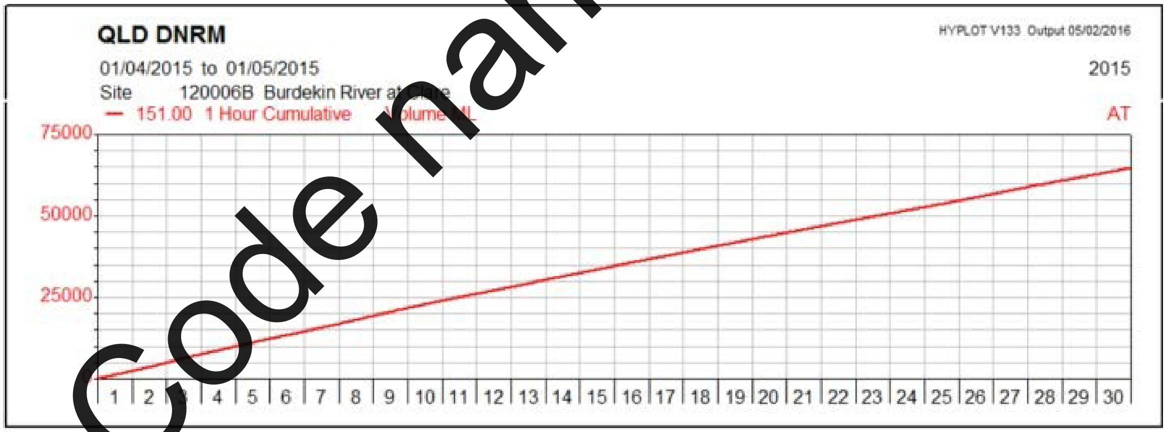

Useful Links:

i. "120006B Burdekin River at Clare" https://water-monitoring.information.qld.gov.au?ppbm=120006B&rs&1&rscf_org

ii. AIMS inshore Water Quality monitoring reports http://eatlas.org.au/rrmmp/gbr-aims-inshore-water-quality

Code names removed

Question 9 Results

Your estimate compared to your group (# Confidence intervals have been standardized to 80%)

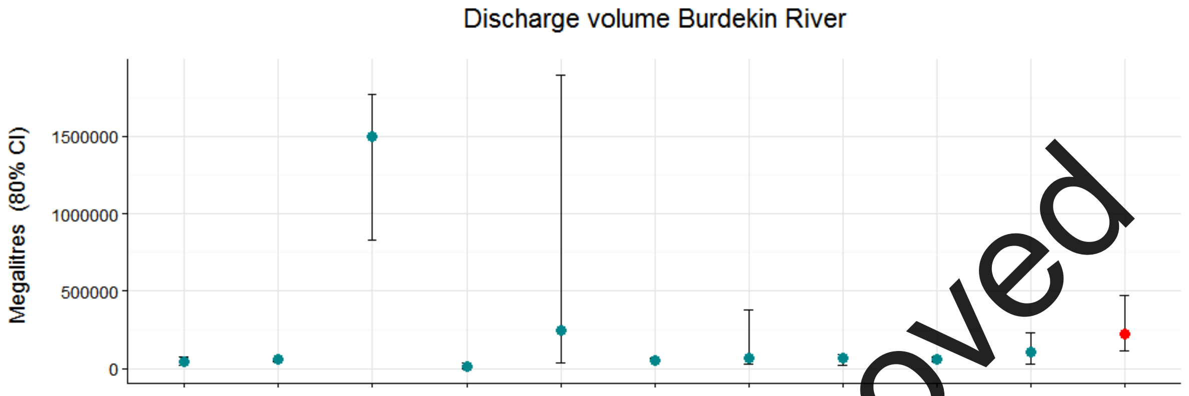

Best	48000.00	64000.00	1500000.00	15000.00	250000.00	55000.00	70000.00	65000.00	60000.00	110000.00	223700.00
Low	24533.33	45333.33	833333.33	0.00	38235.29	48333.33	30000.00	25000.00	46666.67	30000.00	112143.53
High	76800.00	72000.00	1766666.67	39000.00	1897058.82	68333.33	376666.67	89000.00	80000.00	230000.00	469552.55

Code names removed

Comments and questions

Name	Comments
	Discharge this year seems considerable greater than last (for same time of year).
	i'm sorry, but i'm not able to answer.
	Used past 10 years of data to estimate
	Supposed to be less storms and cyclones this year, so less rain water contributing to the river and its outflow
	Bureau outlook for March to May is that rainfall will be drier than average. Long term average: 550503 ML; median 179,727 ML
Facilitator	Some good comments! Any additional comments?
**Comments?**	

Additional Links

#	links
1	https://water-monitoring.information.qld.gov.au/?ppbm=120006B&rs&1&rscf_org
2	http://www.bom.gov.au/climate/outlooks/#/rainfall/total/75/seasonal/0
3	http://www.bom.gov.au/climate/outlooks/#/rainfall/summary
4	http://www.bom.gov.au/jsp/ncc/cdio/weatherData/av?p_nccObsCode=139&p_display_type=dataFile&p_stn_num=033035
**Links?**	

“The Great Barrier Reef Intelligence Game, 2016”
Victoria Hemming, Mark Burgman, Terry Walshe. Anca Hanea.
School of Biosciences, University of Melbourne

Question 10 Chlorophyll Levels Detected at Pine Island

“What will be the average Chlorophyll level (ugL $^{-1}$) for at Pine Island in the Mackay Whitsunday region, in March 2016 recorded by the Wet Labs Eco FLNTUSB?”

Clarification: The Australian Institute of Marine Science measure the Chlorophyll levels at Pine Island in the Mackay –Whitsunday region of Queensland using Eco FLNTUSB instruments. The results inform the Marine Monitoring Program’s (MMP) Inshore Water Quality Monitoring (more information provided below). This question relates specifically to the Eco FLNTUSB instrument currently deployed at Pine Island in the Mackay Whitsunday region. We are interested in what the average daily chlorophyll level will be for March 2016 (averaged from 1 March, 2016 to 31 March 2016 (inclusive)).

Resolution: This question will be resolved on 1 July 2016 by the AIMS Marine Monitoring Program.

Additional Information:

a. Eco FLNTUSB chlorophyll monitoring.

The Eco FLNTUSB instruments deployed by the AIMS MMP record in situ measurements of chlorophyll fluorescence, turbidity and temperature. The term “Chlorophyll” is used by AIMS rather than “Chlorophyll-α”, because the Eco FLNTUSB instruments can only measure the fluorescence from the chlorophyll pigments AND their degradation products, rather than specifically measuring Chlorophyll-α levels. Each instrument is checked prior to deployment and on collection to ensure that measurements are calibrated to maximum and minimum levels of chlorophyll. After retrieval from the field locations, the instruments are cleaned and data downloaded and converted from raw instrumental records into actual measurement units (µg L $^{-1}$ for chlorophyll fluorescence) according to standard procedures by the manufacturer. Deployment information and all raw and converted instrumental records are then stored in an Oracle-based data management system developed by AIMS. Records are quality-checked using a time-series data editing software. Instrumental data are validated by comparison with chlorophyll and suspended solid concentrations obtained by analyses of water samples collected close to the instruments, during change over.

b. Previous trends at Pine Island (source AIMS 2011 inshore water quality program, see useful links)

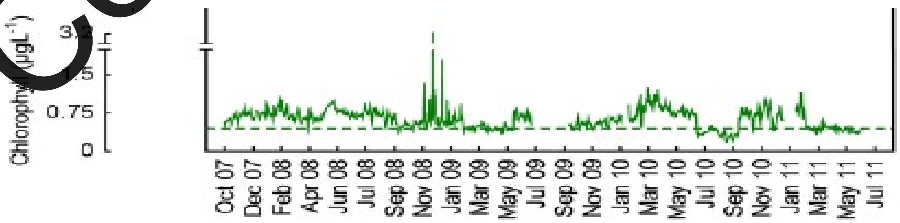

Code names removed

Useful Links:

i. Chlorophyll monitoring http://www.aims.gov.au/docs/data-centre/chlorophyllmonitoring.html

ii. AIMS 2011 Inshore Water Quality Program http://eatlas.org.au/rrmmp/gbr-aims-inshore-water-quality

Question 10 Results

Your estimate compared to your group (# Confidence intervals have been standardized to 80%)

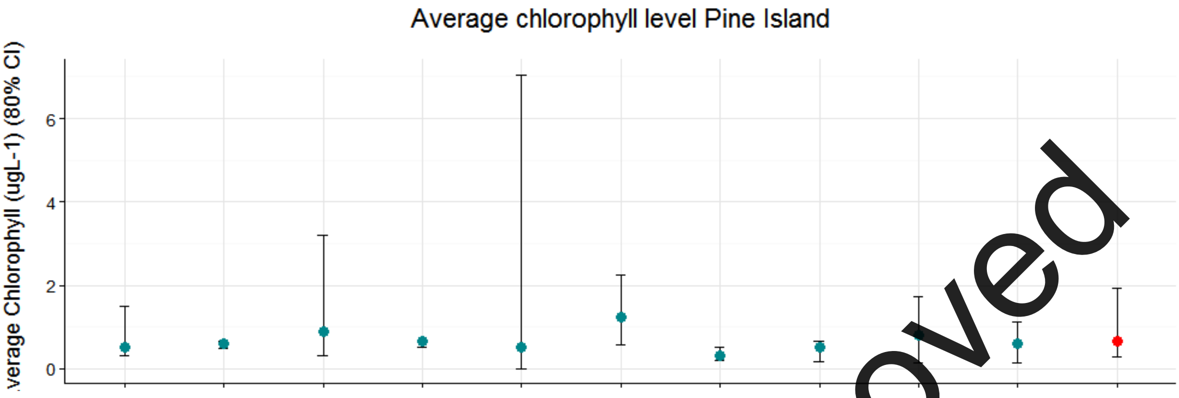

Best	0.50	0.59	0.90	0.65	0.50	1.25	0.30	0.50	0.80	0.60	0.66
Low	0.30	0.48	0.30	0.50	0.00	0.58	0.20	0.18	0.13	0.13	0.28
High	1.50	0.66	3.20	0.70	7.05	2.25	0.50	0.66	1.73	1.13	1.94

Comments and questions

Name	Comments
KBGC	El Nino year means less nutrification, I make the assumption that this will decrease the overall bloom potential of the phytoplankton population in the Pacific
	Does anyone have an understanding of what might influence chlorophyll levels at Pine Island?
**Comments?**	

Additional Links

#	links
1	http://www.bom.gov.au/marinewaterquality/
**Links?**	

Code names removed

Victoria Hemming, Mark Burgman, Terry Walshe. Anca Hanea.
School of Biosciences, University of Melbourne

Question 11 Wind Speed at Davies Reef

"What will be the highest maximum daily wind-speed (averaged maximum, km/hr) recorded for Davies Reef in May 2016?"

Clarification: This question asks what the highest maximum daily wind-speed (averaged maximum, km/hr) will be for Davies Reef for May 2016 as recorded by the Australian Institute of Marine Science. Note that four readings for the maximum wind-speed are recorded each day at Davies Reef. For the purpose of this questions, the **"maximum daily"** wind-speed is actually an average across these four readings. This question is asking what the **highest** "maximum daily" (averaged maximum) wind-speed will be in May 2015. It is possible that the recording instruments will fail, in such a case, only the days from 1 May - 31 May (inclusive) which are recorded will be used to verify the question.

Resolution: The question will be resolved on 6 June 2016. It will be resolved by the AIMS Historic Data Tool (see useful links), and selecting 1 May to 31 May 2016, by selecting quality controlled data, grouping: day + maximum, selecting data: Wind, Davies Reef, Platform Speed (scalar avg 10 min), and exporting data. For each day, the four readings will be averaged. The highest of these averaged wind speeds will be taken as the answer.

Additional information:

a. In May 2015 the highest maximum daily windspeed (averaged maximum) was 69.00 km / hr

Code names removed

Useful Links:

i. Wind speed (past month) http://data.aims.gov.au/aimsrtds/datatool.xhtml?from=2016-02-18&thru=2016-026&qc=LEVEL1&period=DAY&aggregations=MAX&channels=73

ii. Wind Speed Previous five years http://data.aims.gov.au/aimsrtds/datatool.xhtml?from=2011-02-25&thru=2016-02-26&qc=LEVEL1&period=DAY&aggregations=MAX&channels=73

Question 11 Results

Your estimate compared to your group (# Confidence intervals have been standardized to 80%)

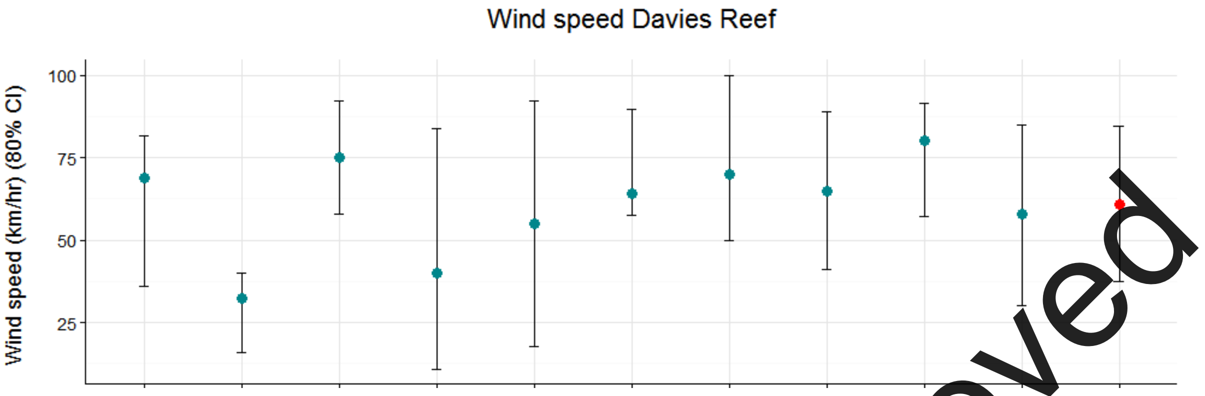

Best	69.00	32.50	75.00	40.00	55.00	64.00	70.00	65.00	80.00	58.00	60.85
Low	35.86	16.00	57.86	10.91	17.67	57.60	50.00	41.00	57.14	30.00	37.40
High	81.57	40.00	92.14	83.64	92.33	89.60	100.00	89.00	91.43	85.00	84.47

Comments and questions

Name	Comments
	First link doesn't work
Facilitator	I'm keen to hear people's thoughts on what would lead to daily maximum windspeed, lower than 60 or above 60.
**Comments?**	

Additional Links

#	links
1	http://data.aims.gov.au/aimsrtds/datatool.xhtml?from=2011-02-25&thru=2016-02-26&qc=LEVEL1&period=DAY&aggregations=MAX&channels=73
**Links?**	

Code names removed

Victoria Hemming, Mark Burgman, Terry Walshe. Anca Hanea.
School of Biosciences, University of Melbourne

Question 12 Average maximum Air Temperature Hamilton Island

"What will be the average maximum air temperature (°C) recorded by the Australian Bureau of Meteorology at Hamilton Island for the month of May, 2016?"

Clarification: The Australian Bureau of Meteorology has a weather station at Hamilton Island on the Great Barrier Reef. The weather station records the daily minimum and maximum temperature for each day of the month. This question asks specifically what you believe will be the average of daily maximum temperatures from 1 May - 31 May 2016. We would like you to provide your answer to one decimal place.

Resolution: The question will be resolved in 15th June, 2016 by the Australian Bureau of Meteorology on their page for Hamilton Island, under "other times and other places" and for "May 16". The value for the mean of the maximum temperature will be taken as the truth.

Additional Information:

a. The average maximum May 2015 was **24.8 C**

Code names removed

Useful Links:

i. Latest weather observations for Hamilton Island: http://www.bom.gov.au/climate/dwo/IDCJDW4054.latest.shtml

ii. Weather observations for April 2015 at Hamilton Island http://www.bom.gov.au/climate/dwo/201504/html/IDCJDW4054.201504.shtml

Question 12 Results

Your estimate compared to your group (# Confidence intervals have been standardized to 80%)

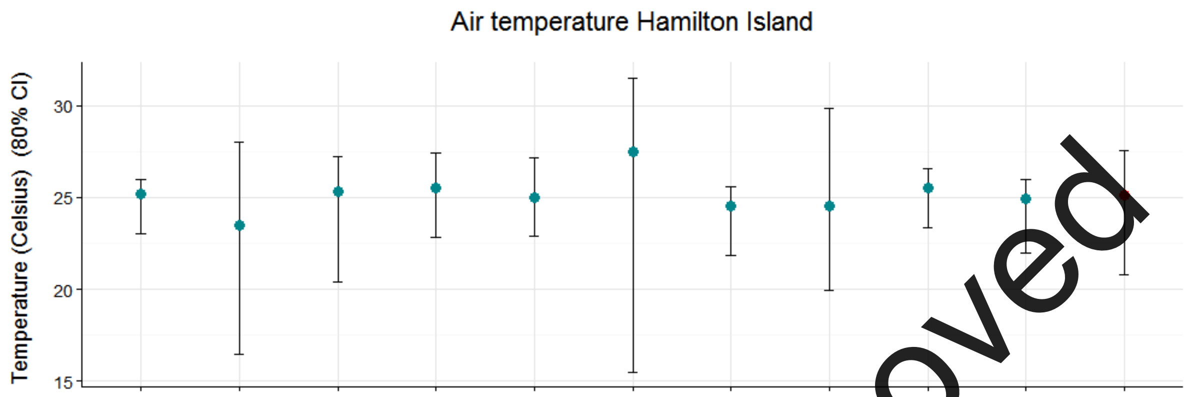

Best	25.20	23.50	25.30	25.50	25.00	27.50	24.50	24.50	25.50	24.90	25.14
Low	23.00	16.50	20.39	22.83	22.87	15.50	21.83	19.97	23.37	22.00	20.83
High	26.00	28.00	27.24	27.42	27.13	31.50	25.57	29.83	26.57	26.00	27.53

Comments and questions

Name	Comments
	Average maximum temperatures in May are very consistent year to year.
Facilitator	The best guess for the group seems to be consistent, however, I'm interested in the lower and upper estimates provided by the group. What events would lead to a substantially decreased temperature, or increased temperature?
**Comments?**	

Additional Links

#	links
1	http://www.bom.gov.au/jsp/ncc/cdio/weatherData/av?p_nccObsCode=122&p_display_type=dailyDataFile&p_startYear=2002&p_c=-219203665&p_stn_num=033106
**Links?**	

Code names removed

Question 13 Turbidity in the Wet Tropics

"What will be the mean turbidity (NTU) for High West (located on High Island) for the month of April, 2016, as recorded by the Australian Institute of Marine Science using their ECO FLNTUSB instruments?"

Clarification: The Australian Institute of Marine Science measures the turbidity levels at High Island, the sampling site is referred to as "High West". High Island is located in the Wet Tropics region of Queensland. The Eco FLNTUSB instruments used by AIMS inform the Marine Monitoring Program's (MMP) Inshore Water Quality Monitoring (more information provided below). This question relates specifically to the Eco FLNTUSB instrument currently deployed at High West on High Island in the Wet Tropics region.

Resolution: This question will be resolved by 30 June 2016 by the AIMS Marine Monitoring Program.

Additional Information:

a. Annual Mean Turbidity: taken from the MMP AIMS Inshore monitoring report for 2013-2014 for High West, on High Island in the Wet Tropics region, Queensland.

Monitoring period	Annual Mean Turbidity (NTU)	Number of sampling days
Oct 2007 – Sep 2008	0.81	356
Oct 2008 – Sep 2009	0.84	365
Oct 2009 – Sep 2010	1.20	365
Oct 2010 – Sep 2011	1.56	365
Oct 2011 – Sep 2012	1.08	366
Oct 2012 – Sep 2013	1.55	365
Oct 2013 – Sep 2014	1.27	213

Code names removed

Useful Links:

i. Marine Monitoring Program report for 2013-2014 http://elibrary.gbrmpa.gov.au/jspui/handle/11017/2975

Question 13 Results

Your estimate compared to your group (# Confidence intervals have been standardized to 80%)

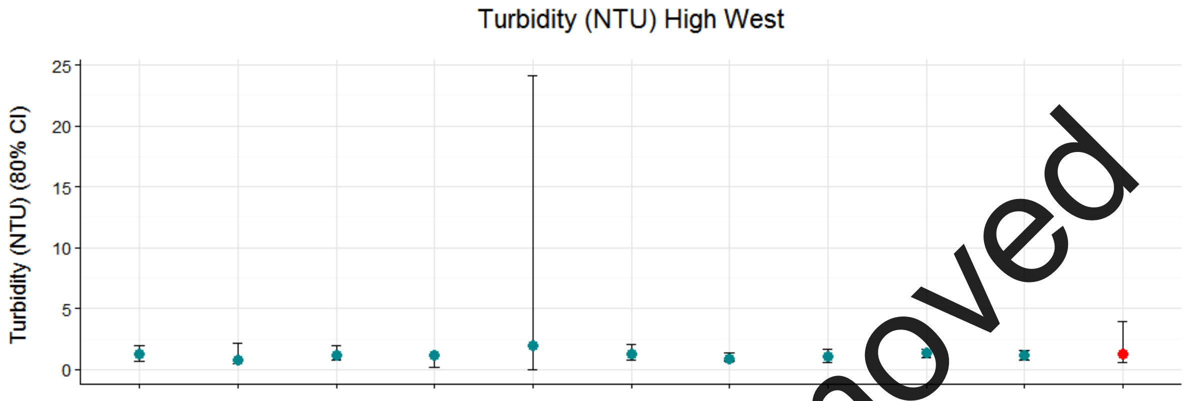

Best	1.30	0.77	1.20	1.16	2.00	1.80	0.90	1.10	1.35	1.21	1.23
Low	0.70	0.46	0.80	0.16	0.00	0.82	0.69	0.57	0.94	0.80	0.59
High	2.00	2.18	2.00	1.22	24.15	2.02	1.33	1.63	1.70	1.60	3.98

Comments and questions

Name	Comments
	Most recent sampling days data is incomplete compared to previous years, excluding this year I estimated trends and patterns
Facilitator	The group seems to be pretty consistent. Emmylou, can you provide some clarification about your estimates?
**Comments?**	

Additional Links

#	links
**Links?**	

Code names removed

“The Great Barrier Reef Intelligence Game, 2016”
Victoria Hemming, Mark Burgman, Terry Walshe. Anca Hanea.
School of Biosciences, University of Melbourne

Question 14 EL Nino Events

“What will be the average sea-surface temperature (°C) for the month of June 2016 within the Nino 3.4 region as reported by the Climate Prediction Center of the National Oceanic and Atmospheric Administration (NOAA)?”

Clarification: The Nino 3.4 region is shown on the map below. The average sea-surface temperature of the Niño 3.4 region is used to detect whether the equatorial Pacific region has entered an El Niño or La Nina weather pattern. This question seeks to understand what you think the average sea-surface temperature will be for the Month of June 2016.

Resolution: The answer will be reported by the National Weather Service Climate Prediction Center of NOAA, in their ongoing monthly monitoring program for Sea-Surface Temperature “Monthly- ERSSTv4 (1981-2010 base period), Niño 3.4 (5°North-5°South)(170-120°West)” (links below), in the “NINO 3.4” column and the row for the year “2016”, and month “6”. The data will be downloaded on 15 July, 2016.

Additional Information:

a. The Nino 3.4 region

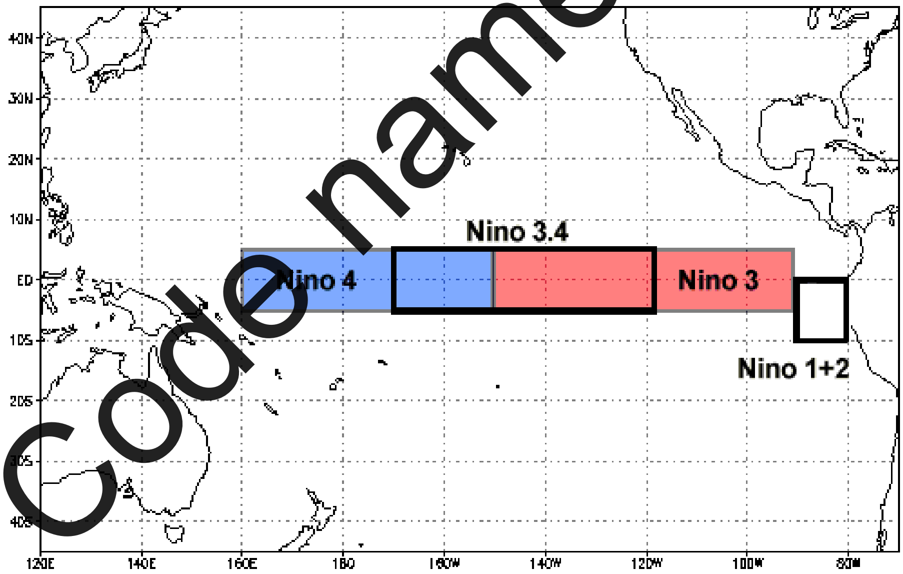

b. The average sea-surface temperature for June 2015 was 28.70 °C

Useful Links:

i. NOAA https://www.ncdc.noaa.gov/teleconnections/enso/indicators/sst.php

ii. Previous trends http://www.cpc.ncep.noaa.gov/data/indices/ersst4.nino.mth.81-10.ascii

Code names removed

Question 14 Results

Your estimate compared to your group (# Confidence intervals have been standardized to 80%)

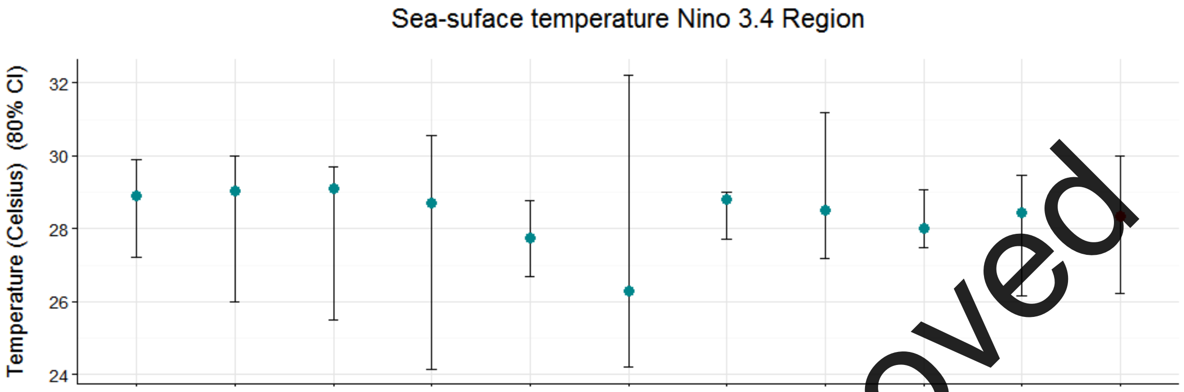

Best	28.90	29.05	29.10	28.70	27.73	26.30	28.80	28.50	28.00	28.43	28.35
Low	27.21	26.00	25.50	24.15	26.69	24.22	27.70	27.17	27.47	26.16	26.23
High	29.88	30.00	29.70	30.55	28.76	32.22	29.00	31.17	29.07	29.46	29.98

Comments and questions

Name	Comments
	I looked at the June 1998 temperatures and I might have created a small linear model using the January values to predict the June values in each year.
Facilitator	Seems to be some minor variation, but most people indicating only minor fluctuations from last year. Participant 6 are you able to provide some comment on

Additional Links

#	links

Code names removed

Victoria Hemming, Mark Burgman, Terry Walshe. Anca Hanea.
School of Biosciences, University of Melbourne

Question 15 The Spread of Zika Virus Throughout the European Union

"How many European Union member states will the World Health Organization report as having at least one laboratory-confirmed human case of any strain of Zika virus for the month of April 2016?"*

Clarification: The World Health Organization (WHO) held a special session on 4 February 2016 on the Zika virus for quick action against the infection linked to thousands of birth defects in Brazil that is spreading through Latin America and the Caribbean (Reuters- see useful links). The virus, carried by the *Aedes aegypti* mosquito, was originally found in tropical and subtropical zones, but is today found on all continents except Antarctica.

*European patients whose cases of Zika virus have been confirmed as part of the outbreak in the Americas and are transported back to Europe for treatment will not count. Cases reported as "suspected" prior to the question's closing date will not count, even if they are "confirmed" after the question's closing date. For a list of the 28 European Union member states (see useful links). Please note this question refers to all reports confirmed by the World Health Organisation (WHO) during the month of April (including those made for member states which have previously been confirmed to contain Zika Virus).

Resolution: Outcome will be determined by WHO's Disease outbreak news for Zika virus infection, found at the World Health Organisation (see useful links) or in credible open source media reports (e.g., Reuters, BBC, AP).

Additional Information: None

Code names removed

Useful Links:

i. World Health Organisation Reports for Zika Virus — http://www.who.int/csr/don/archive/disease/zika-virus-infection/en/

ii. List of 28 member countries for the EU — http://europa.eu/about-eu/countries/member-countries/index_en.htm

iii. News article about Zika virus — http://www.reuters.com/article/us-health-zika-idUSKCN0V523W

THE UNIVERSITY OF MELBOURNE

"The Great Barrier Reef Intelligence Game, 2016"
Victoria Hemming, Mark Burgman, Terry Walshe. Anca Hanea.
School of Biosciences, University of Melbourne

cebra Centre of Excellence for Biosecurity Risk Analysis

Question 15 Results

Your estimate compared to your group (# Confidence intervals have been standardized to 80%, maximum capped at 28 EU member states (each member state can only be counted once).

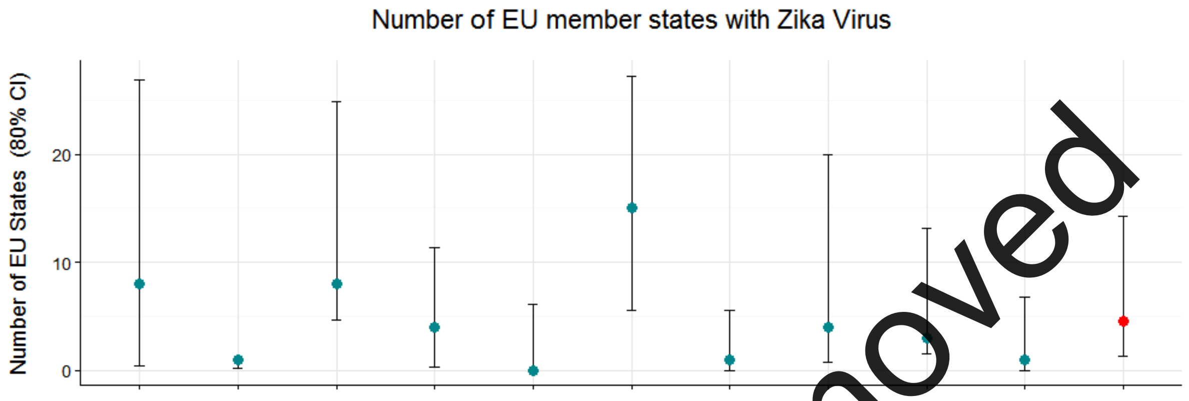

Best	8.00	1.00	8.00	4.00	0.00	15.00	1.00	4.00	3.00	1.00	4.50
Low	0.47	0.16	4.63	0.31	0.00	5.59	0.00	0.80	1.55	0.00	1.35
High	26.82	1.00	24.84	11.38	6.15	27.24	5.57	20.00	13.18	6.82	14.30

Code names removed

Comments and questions

Name	Comments
	Am assuming this includes overseas territories of France, Netherlands, Britain, in Latin Am/Caribbean, only French and Dutch territories have outbreaks now, only some of the French territories have EU/OMR status, am guessing given the number of islands involved there will still be some cases by April even if caution/measures improve
	I assume you don't include EU member state overseas holdings. French overseas territories are regarded as part of France and consequently part of the EU.
Facilitator	Yes, to clarify, for the purpose of this question we are only interested in the number of cases reported for member countries on continental Europe. What would need to be true for some of the **maximum estimates** to be realized? What would need to be true for some of the **minimum estimates**?
**Comments?**	

Additional Links

#	links
1	http://www.europarl.europa.eu/atyourservice/en/displayFtu.html?ftuId=FTU_5.1.7.html
2	http://ecdc.europa.eu/en/healthtopics/zika_virus_infection/zika-outbreak/Pages/Zika-countries-with-transmission.aspx
**Links?**	

“The Great Barrier Reef Intelligence Game, 2016”
Victoria Hemming, Mark Burgman, Terry Walshe. Anca Hanea.
School of Biosciences, University of Melbourne

Question 16 The Price of Gold

“What will be the closing spot price of gold on 30 May 2016?”

Clarification: Gold prices declined in 2015 as the Federal Reserve System signaled its first interest-rate increase in nearly a decade, but has recovered recently amid global financial market turmoil and fears that economic growth is slowing (WSJ- see useful links). We are interested in what you think the spot price of gold will be at the end of the day (EST) on the 30 May, 2016.

Resolution: Outcome will be determined by the end-of-day (i.e., 23:59:59 EST on 30 May 2016) closing spot price for gold in U.S. dollars according to Bloomberg, at http://www.bloomberg.com/quote/xauusd:cur. In case of delayed reporting or problems with the Bloomberg website, reporting by other credible open sources may be used.

Code names removed

Useful Links:

i. WSJ News article http://www.wsj.com/articles/gold-gains-as-global-markets-plunge-1453292225?tesla=y

ii. Spot price of Gold http://www.bloomberg.com/quote/xauusd:cur

THE UNIVERSITY OF MELBOURNE

"The Great Barrier Reef Intelligence Game, 2016"
Victoria Hemming, Mark Burgman, Terry Walshe. Anca Hanea.
School of Biosciences, University of Melbourne

cebra
Centre of Excellence for Biosecurity Risk Analysis

Question 16 Results

Your estimate compared to your group (# Confidence intervals have been standardized to 80%)

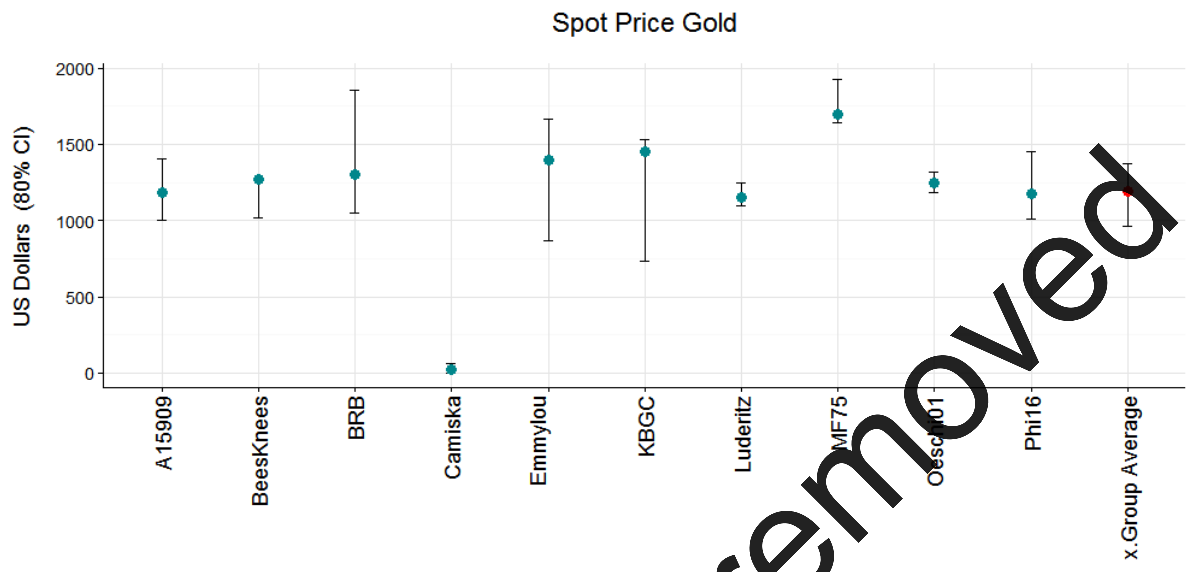

	A15909	BeesKnees	BRB	Camiska	Emmylou	KBGC	Luderitz	MF75	Oeschi01	Phi16	x.Group Average
Best	1180.20	1272.30	1300.00	25.00	1400.00	1450.00	1150.00	1700.00	1250.00	1175.50	1190.30
Low	1002.80	1019.50	1050.00	1.00	866.67	730.00	1100.00	1642.86	1183.33	1008.17	960.43
High	1400.80	1281.33	1855.00	65.00	1666.67	1530.00	1250.00	1928.57	1316.67	1450.83	1374.49

Comments and questions

Name	Comments
BeesKnees	looked at bloomberg business sport price trends and at some of the factors affecting gold price (e.g. see sites below), assume the price might rally a little but not much (also it is a US election year), best guess is a bit of a guess
BRB	Highest estimate obtained from 5 year figures.
Camiska	i'm sorry, but i'm not able to answer.
Facilitator	The stock market is difficult. Does anyone have a comment as to what might create a rise or fall in the price of Gold?
Comments?	

Additional Links

#	links
1	http://www.sbcgold.com/blog/10-factors-regularly-influence-gold-prices/
2	http://www.forbes.com/sites/investor/2013/08/06/whats-moving-the-price-of-gold/#407653c15ae6
Links?	

Code names removed

THE UNIVERSITY OF MELBOURNE

"The Great Barrier Reef Intelligence Game, 2016"
Victoria Hemming, Mark Burgman, Terry Walshe. Anca Hanea.
School of Biosciences, University of Melbourne

cebra Centre of Excellence for Biosecurity Risk Analysis

Question 17 The UK Referendum

"What will be the final percentage of votes made ***in favour*** *of the United Kingdom remaining a member of the European Union during the UK referendum to be held on 23 June, 2016?"*

Clarification: On Saturday the 20th February, 2016, David Cameron, the Prime Minister of the United Kingdom called a referendum to determine whether the United Kingdom should remain in the European Union or leave the European Union. The referendum will take place on 23 June 2016.

This question aims to determine what percentage of people who choose and are permitted to vote in the upcoming United Kingdom referendum to be held on 23 June, 2016, will vote in favour of the United Kingdom remaining a member of the European Union. Please note that only valid votes will be included in the final percentage.

Resolution: This answer to this question will be resolved by the United Kingdom Electoral Commission when they announce the final result of the referendum either on their webpage or in credible open source media reports (e.g., Reuters, BBC, AP).

Code names removed

Useful Links:

i.	About the Referendum	http://www.bbc.com/news/uk-politics-32810887
ii.	Opinion Polling	http://whatukthinks.org/eu/opinion-polls/poll-of-polls/
iii.	The UK Electoral Commission	http://www.electoralcommission.org.uk/find-information-by-subject/elections-and-referendums/upcoming-elections-and-referendums/eu-referendum

Question 17 Results

Your estimate compared to your group (# Confidence intervals have been standardized to 80%, maximum capped at 100%).

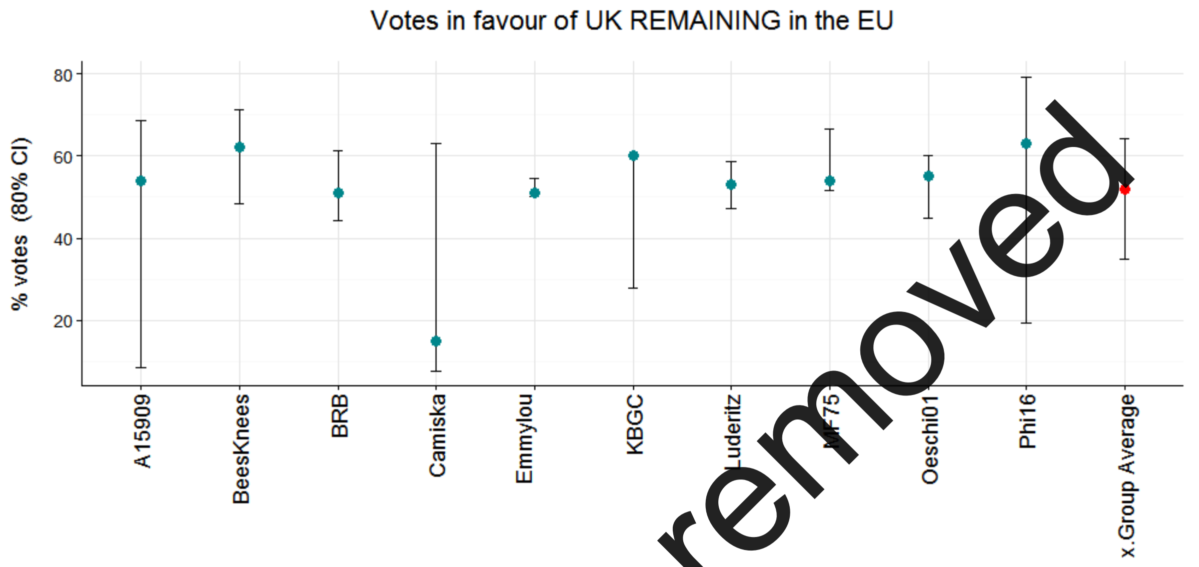

	A15909	BeesKnees	BRB	Camiska	Emmylou	KBGC	Luderitz	MF75	Oeschi01	Phi16	x.Group Average
Best	54.00	62.00	51.00	15.00	51.00	60.00	53.00	54.00	55.00	63.12	51.81
Low	8.67	48.29	44.14	7.73	50.11	28.00	47.29	51.71	45.00	19.36	35.03
High	68.67	71.14	61.29	63.00	54.56	60.00	58.71	66.57	60.00	79.23	64.32

Comments and questions

Name	Comments
	rough guesses based off of polls and assuming some margin of error and that some "i don't know"s become Yes', assume maintaining status quo is preferred
	This shit always ends up in a narrow split vote in the UK
Facilitator	Seems to be a narrow split for the number of votes in favour of staying in the EU. What might cause this to change in the next few months?
**Comments?**	

Additional Links

#	links
**Links?**	

Code names removed

Question 18 The Stock Price of Twitter

“What will Twitter’s end-of-day stock price be on 30 May 2016?”

Clarification: Twitter’s stock price is reported on Nasdaq.com (see useful links). We are interested in what you realistically think the stock price will be at the end of the day on the 30 of May 2016?

Resolution: The outcome will be determined by the end-of-day (i.e., 15:59:59 EST on 30 May 2016) stock price for Twitter (TWTR) in U.S. dollars according to Nasdaq National Market, at http://www.nasdaq.com/symbol/twtr. In case of delayed reporting or problems with the Nasdaq website, reporting by other credible open sources may be used.

Code names removed

Useful Links:

i. News reports https://www.technologyreview.com/s/546286/is-facebook-about-to-kill-off-twitter/

ii. Twitter's Stock Price http://www.nasdaq.com/symbol/twtr.

Question 18 Results

Your estimate compared to your group (# Confidence intervals have been standardized to 80%)

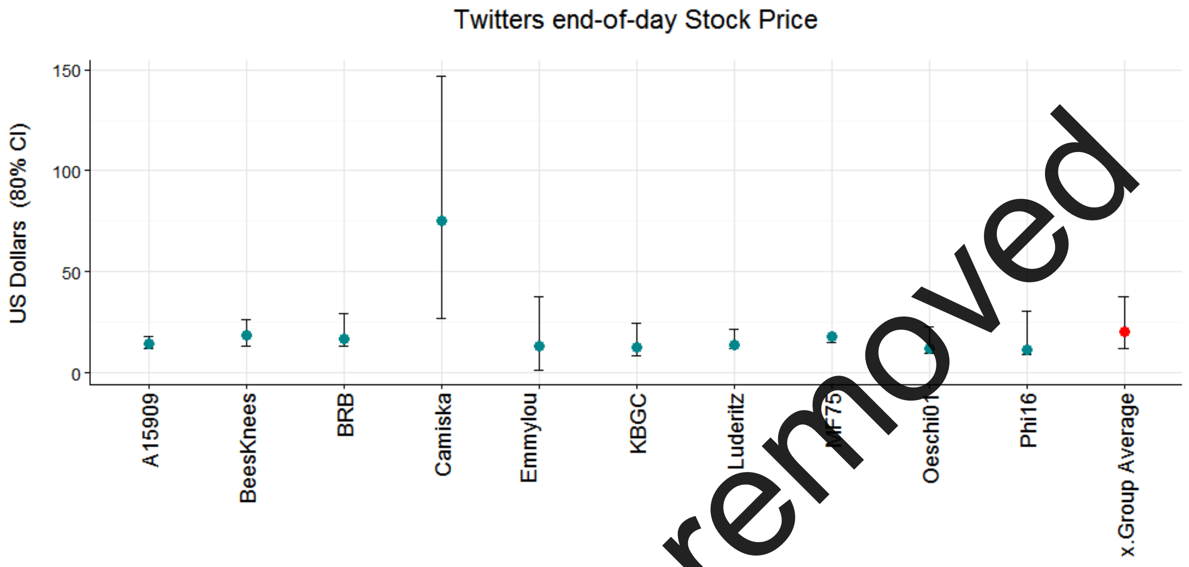

	A15909	BeesKnees	BRB	Camiska	Emmylou	KBGC	Luderitz	MF75	Oeschi01	Phi16	x.Group Average
Best	14.56	18.60	17.00	75.00	13.00	12.50	14.00	17.80	12.00	11.12	20.56
Low	11.83	13.24	13.24	27.00	1.36	8.50	11.87	14.73	9.33	8.89	12.00
High	18.23	26.49	29.24	147.00	37.73	24.50	21.47	18.40	22.67	30.19	37.59

Comments and questions

Name	Comments
	i'm sorry, but i'm not able to answer.
	Again… the stock market. Notoriously difficult to estimate. Does anyone have any information about what might cause fluctuations in Twitter’s stock?
**Comments?**	

Additional Links

#	links
1	http://marketrealist.com/2016/02/analysts-recommend-twitter/?source=nasdaq
2	http://seekingalpha.com/article/3953036-twitter-19-scream-buy
3	http://www.nasdaq.com/symbol/twtr/interactive-chart?timeframe=1y&charttype=line
**Links?**	

Code names removed

Question 19 The Throughput (tonnes) from Abbot Point Port.

"What will be throughput (tonnes) from Abbot Point Port in May 2016 as reported by the North Queensland Bulk Ports Corporation?"

Clarification: Abbot Point is a bulk coal terminal located between Townsville and Mackay. It is operated by the North Queensland Bulk Ports Corporation (NQBP). Each month the amount of coal (tonnes) exported from Abbot Point is publicly reported by NQBP (referred to as "throughput"). For this question we would like you to tell us what the throughput (tonnes) will be for May 2016, as reported by North Queensland Bulk Ports Corporation for their Abbot Point Port.

Resolution: The question will be resolved when North Queensland Bulk Ports Corporation publish the throughput for their Abbot point port for May 2016. Currently this information is provided on the NQBP webpage for Abbot Point Port (see useful links).

Additional Information:

a. Monthly throughput (tonnes) of Abbot Point Port reported by North Queensland Bulk Ports Corporation 2011-2016.

	2011/12	2012/13	2013/14	2014/15	2015/16
July	1,082,351	1,061,932	1,914,825	2,549,533	2,137,081
Aug	1,374,398	1,434,153	2,071,065	2,689,449	2,325,753
Sep	1,295,225	1,173,161	1,542,640	2,388,651	3,071,717
Oct	1,369,123	1,128,913	2,012,186	2,594,124	2,063,059
Nov	1,150,840	1,361,358	1,991,152	2,447,860	2,104,546
Dec	1,045,067	1,737,743	2,032,412	2,546,134	2,011,407
Jan	1,161,504	1,879,052	1,487,780	2,031,489	2,162,742
Feb	865,328	1,237,236	2,019,920	2,091,416	
Mar	970,632	1,377,904	1,439,730	2,676,541	
Apr	1,183,926	1,795,160	1,808,698	2,425,631	
May	1,186,281	1,881,360	2,166,790	1,908,950	?
June	917,462	1,676,749	2,408,353	2,380,601	
	**13,602,137**	**17,744,621**	**22,895,551**	**28,730,365**	

Useful Links:

i. Throughput to Abbot Point http://www.nqbp.com.au/ports-throughput/

Code names removed

Question 19 Results

Your estimate compared to your group (# Confidence intervals have been standardized to 80%)

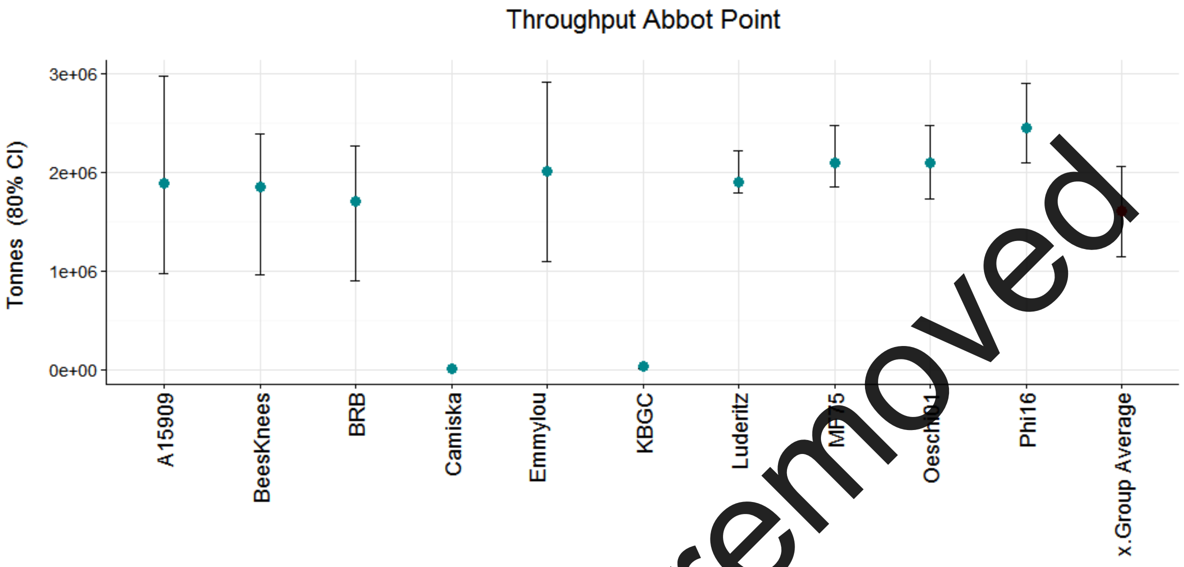

	A15909	BeesKnees	BRB	Camiska	Emmylou	KBGC	Luderitz	MF75	Oeschi01	Phi16	x.Group Average
Best	1886432.00	1850000.00	1700000.00	5000.00	2003787.00	29500.00	1900000.00	2100000.00	2100000.00	2450000.00	1602472.00
Low	971189.30	963333.30	900000.00	200.00	1094072.90	14300.00	1793333.30	1853846.10	1730769.20	2100000.00	1142104.40
High	2977269.00	2383333.00	2271429.00	9800.00	2913501.00	30300.00	2220000.00	2469231.00	2469231.00	2900000.00	2064409.00

Comments and questions

Name	Comments
	Used the 'forecast' package in R
	i'm sorry, but i'm not able to answer.
Facilitator	The group average seems to be in line with previous trends. Are there any events in the world of coal that might substantially reduce or increase the tonnage from Abbot Point?
**Comments?**	

Additional Links

#	links
**Links?**	

Code names removed

“The Great Barrier Reef Intelligence Game, 2016”
Victoria Hemming, Mark Burgman, Terry Walshe. Anca Hanea.
School of Biosciences, University of Melbourne

Question 20 People Held in Nauru Regional Processing Centre, April 2016

“How many people will be held in the Republic of Nauru Regional Processing Centre in April 2016?”

Clarification: The Australian Government has established a regional processing centre in the Republic of Nauru which holds people “who have arrived without a visa, overstayed their visa or have had their visa cancelled”. As at 31 January 2016 there were 484 people held in the Republic of Nauru Regional Processing Centre. This question seeks to understand how many people you believe will be recorded in the Republic of Nauru Regional Processing Centre as declared by the Australian Department of Immigration and Border Protection in their report for “Immigration Detention and Community Statistics Summary” for 30 April 2016.

Resolution: This question will be resolved by the Australian Department of Immigration and Border Protection in their report for “Immigration Detention and Community Statistics Summary for 30 April 2016”.

Additional Information:

a. The last report, 31 January, 2016 declared there are **484 people** currently in the Republic of Nauru regional processing centre

Code names removed

Useful Links:

i. Immigration detention statistics http://www.border.gov.au/about/reports-publications/research-statistics/statistics/live-in-australia/immigration-detention

ii. About Immigration detention http://www.border.gov.au/Busi/Comp/Immigration-detention

Question 20 Results

Your estimate compared to your group (# Confidence intervals have been standardized to 80%)

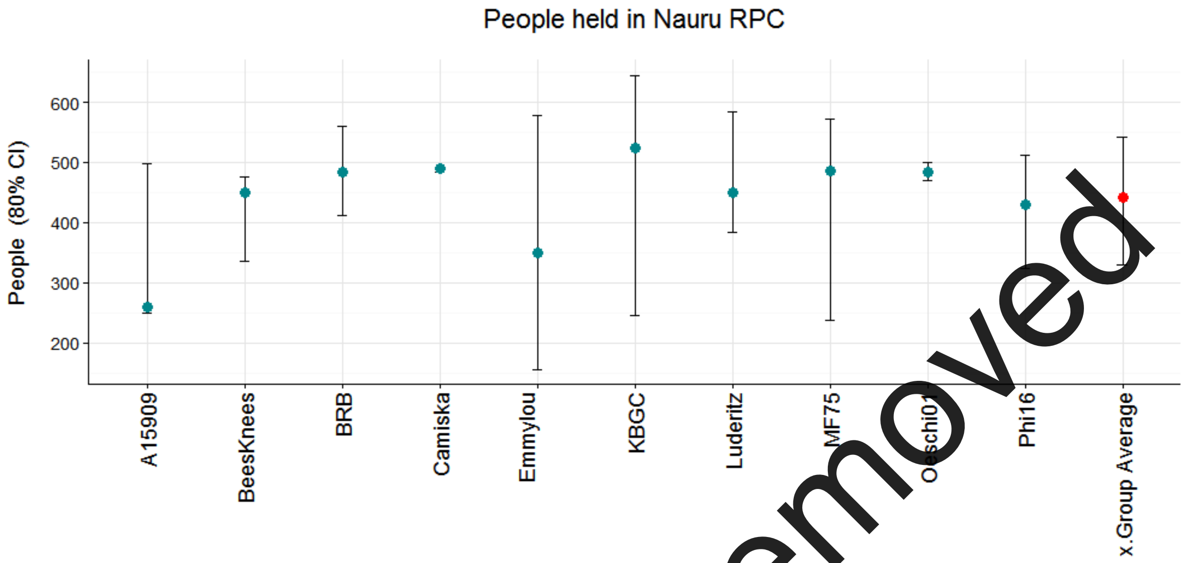

	A15909	BeesKnees	BRB	Camiska	Emmylou	KBGC	Luderitz	MF75	Oeschi01	Phi16	x.Group Average
Best	260.00	450.00	484.00	490.00	350.00	525.00	450.00	486.00	484.00	430.00	440.90
Low	249.33	336.67	410.86	483.14	155.71	245.00	383.33	238.00	470.00	323.33	329.54
High	498.93	476.67	559.43	490.00	578.57	645.00	583.33	571.33	500.00	512.67	541.59

Comments and questions

Name	Comments
	Estimate based on the average net monthly change in detainees for the past 15 months
	I predict increased immigration, but the system might be ready for a higher influx and process people faster to handle the increased volume of people in
Facilitator	There seems to be a lot of variation in our group estimates. What are people’s thoughts on potential increased and decreased in the number of people held at Nauru RPC?
**Comments?**	

Additional Links

#	links
1	http://parlinfo.aph.gov.au/parlInfo/download/committees/estimate/06e2d3df-3fe5-4bce-b155-8e43d929d5ea/toc_pdf/Legal%20and%20Constitutional%20Affairs%20Legislation%20Committee_2016_02_08_4126.pdf;fileType=application%2Fpdf
**Links?**	

Code names removed

Question 21 Launches to Space in May 2016

“How many space launches will take place in May 2016?”

Clarification: This question seeks to understand how many space launches you realistically think will take place between 1 May 2016 and 30 May 2016 across the globe. Space launches are most commonly satellites, however, can include the launch of supplies for space stations, science exploration and other reasons. For this question we will include anything considered a space launch by the “space launch report”. We will include anything that is launched regardless of whether it subsequently fails or succeeds its intended mission.

Resolution: This question will be resolved by the space launch report

Additional Information:

a. Recent space launches for 2016

01/20/16, 04:01 UTC, PSLV-XL with IRNSS 1E from SR 2 to GTO-
01/27/16, 23:20 UTC, Ariane 5-ECA with Intelsat 29e from KO 3 to GTO
01/29/16, 22:20 UTC, Proton M/Briz M with Eutelsat 9B from TB 200/39 to GTO+
02/01/16, 07:29 UTC, CZ-3C/YZ-1 with Biedou M3-S from XC 2 to MEO
02/05/16, 13:38 UTC, Atlas 5 with GPS 2F-12 from CC 41 to MEO
02/07/16, 00:21 UTC, Soyuz 2-1b/Fregat with Glonass M from PL 43//4 to MEO
02/07/16, 00:30 UTC, Unha 3 with Kwangmyongsong 4 from SO to LEO
02/10/16, 11:40 UTC, Delta 4+5,4 with NROL-45 from VA 6 to LEO/R
02/16/16, 17:57 UTC, Rokot/Briz KM with Sentinel 3A from PL 133/3 to LEO/S
02/17/16, 08:45 UTC, H-2A-202 with ASTRO-H from TA Y1 to LEO

Code names removed

Useful Links:

i. The space launch report http://www.spacelaunchreport.com/

Question 21 Results

Your estimate compared to your group (# Confidence intervals have been standardized to 80%)

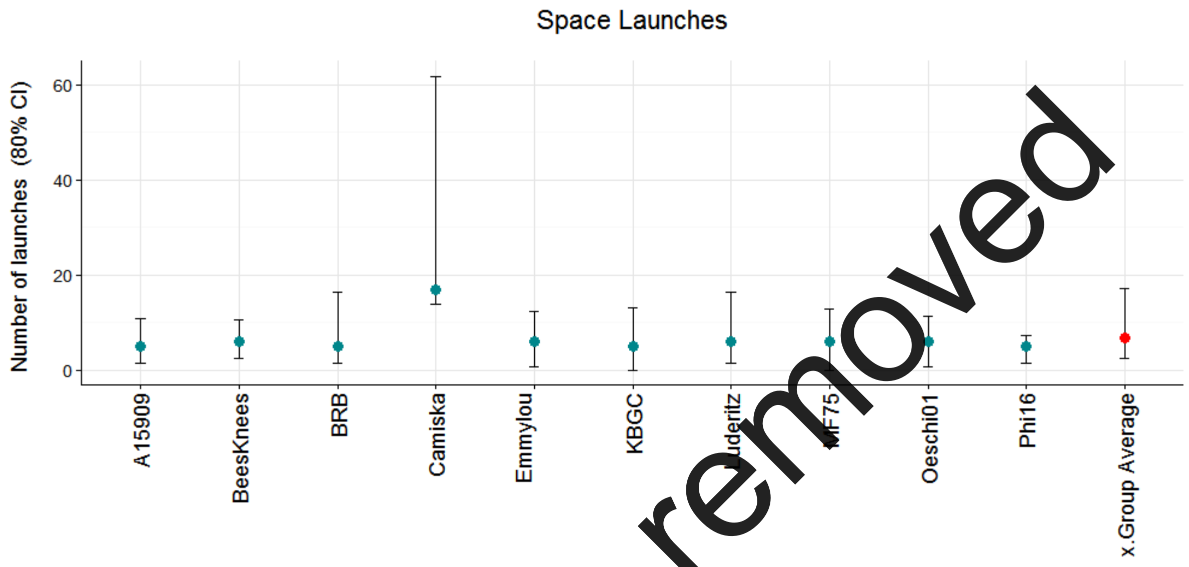

	A15909	BeesKnees	BRB	Camiska	Emmylou	KBGC	Luderitz	MF75	Oeschi01	Phi16	x.Group Average
Best	5.00	6.00	5.00	17.00	6.00	5.00	6.00	6.00	6.00	5.00	6.70
Low	1.57	2.57	1.57	13.80	0.67	0.00	1.43	0.00	0.67	1.57	2.38
High	10.71	10.57	16.43	61.80	12.40	13.00	16.29	12.86	11.33	7.29	17.27

Comments and questions

Name	Comments
	looked at launch logs for 2010-2015, looked at number of launches per month in general and for May specifically, took an average for best guess, kept an eye on who was launching in case trends looked<br>like they might change suddenly
	i'm sorry, but i'm not able to answer.
	Based on distribution of May launches, 2006-2015
Facilitator	The group average appears to be consistent with previous trends. Is there anything happening in the world that might lead the number to be substantially more or less?
**Comments?**	

Additional Links

#	links
**Links?**	

Code names removed

"The Great Barrier Reef Intelligence Game, 2016"
Victoria Hemming, Mark Burgman, Terry Walshe, Anca Hanea.
School of Biosciences, University of Melbourne

Responsible Researcher: Prof Mark Burgman, University of Melbourne, Ph: contact details omitted
Co-researcher: Victoria Hemming, University of Melbourne,contact details omitted
Co-researcher: Dr Terry Walshe, Australian Institute of Marine Sciences, contact details omitted

This research has been approved by the Human Ethics Committee of The University of Melbourne (HREC Project Number: 1546009.1). If you have any concerns about the conduct of this study that the researchers have not been able to answer to your satisfaction, you may contact the Executive Office, Human Research Ethics, The University of Melbourne, 03 8344 2073 (phone) or 03 9347 6739 (fax).

Code names removed

Contents

Group Discussion from Round 1 Results: 2

Additional clarification and edits from Victoria. 10

Question 2: Number of reefs with at least 1% coral bleaching. 10

Question 8: Number of days the water temperature would be at least 28°C or more...... 11

Question 15: Number of member states reported to have Zika Virus 13

Question 17: Percentage of Votes in favour of the UK remaining in the EU..................... 14

Group Discussion from Round 1 Results:

Question	Code Name	Correction / Comment	Date
**Q1 Crown of Thorns densities**	3	I looked at the links for some of the surrounding reefs and it would not appear the current extent of COTS outbreaks is that great. I may have misinterpreted the data and definition of active vs incipient outbreaks though.	21/03/2016
**Q2 Number of reefs with at least 1% coral bleaching**	3	If anyone could help with information about current status of bleaching on the reef that would be really helpful. It does seem there is a lot of media at the moment concerning risk of another bleaching event, similar to past events	21/03/2016
2	10	The current media on bleaching seems to indicate widespread bleaching is occurring, but my reading of past data is that it is highly variable. If I'm reading the records correctly, some of these reefs have some (fairly minor) evidence of bleaching but some do not. I would suspect that conditions are good for bleaching.	21/03/2016
2	5	Residual bleaching I don't think so - other than it being an El Nino and there being a high likelihood of GBR-wide bleaching, it is difficult to predict	22/03/2016
**Q3 Asian Green Mussel Detections**	10	I used the reporting data to estimate this one. I can't think of any reasons why it would change in the coming months.	21/03/2016
**Q4 White Syndrome**	3	I think if there is a link to higher sea temperatures it's possible that prevalence might be a bit higher, but I'm unsure what the strength of the relationship is.	21/03/2016

**Q5 Commercial catch Coral Trout**	3	I was unsure about this one...	21/03/2016
Q5	10	I had similar reasoning to participant 6, although not as significant reduction in the catch (based on the historical catch data).	21/03/2016
Q5	8	I estimated a wide range as final catch dependent on the number of active licenses, the duration of the season, weather during that period and fish population during last 12 months. All of these factors have to be estimated.	23/03/2016
**Q6 Turtle Strandings**	3	No idea!!	21/03/2016
Q6	10	Not really.	21/03/2016
Q6	8	Higher upper limit was to account for potential summer disease outbreak, as was seen in previous years that may cause a localised mass stranding.	23/03/2016

Q6	Facilitator	Interesting. Do you know which years this was? And what the disease was?<br><br>So this question asks about 1 January 2016 – 31 March. Which is almost over.<br><br>This question has a number of prior states and conditional probabilities including: the residual turtle population (more turtles = more strandings), Sources of mortality (disease, cyclones, by-catch, food, predation, vessel strikes , any others?), and then whether the turtle is seen and then reported, and then confirmed by EHP.<br><br>Does anyone have a sense of whether any of these variables might have changed over the past year? E.g. has there been a large decline in turtles, or an outbreak of disease, or are the public less likely to report a stranding, has EHP funding been cut which might mean less reports are investigated.<br><br>There are endless possibilities, a little bit of local knowledge might help here!	23/03/2016
**Q7 Sharks caught**	10	I would not have thought them to be as low as 2011-2012 which were years of significant floods. Is there a possible causal link with floods and low numbers of sharks or am I just seeing an accident of the data?	21/03/2016
Q7	5	There should be no change in shark catch; nothing to suggest their population or behaviour would change radically	22/03/2016
**Q8 Days where water temperature would be at least 28°C or above**	3	Yes, looking at the long term monitoring data for the station, I think I was too conservative with my upper bound. Especially given the 2015 records	21/03/2016
Q8	10	I would expect that sea temps will be higher this year given reports of higher temperatures everywhere. I've used historical data and increased temps by 1 and 2 degrees on historical change the probability of occurrence and obtain my estimate.	21/03/2016

Q8	Facilitator	I've included some extra information about this question in the second part of this document.	21/03/2016
Q8	8	Reconsidering- was an overestimation based on a prolonged summer given extended seasons of last couple of years.	23/03/2016
**Q9 Discharge Burdekin River**	3	ARGH! I tried to look at the discharge volume for this year, but perhaps I chose the wrong criteria. Has anyone else taken a look at this?	21/03/2016
Q9	Facilitator	Could your estimate have been a median? I'd need to check but it still may be within the range of previous years.	21/03/2016
**Q10 Chlorophyll levels Pine Island**	3	Sorry, I was really unsure about this. Any extra info would be really appreciated.	21/03/2016
Q10	10	Higher sea temperatures likely to increase the bloom potential.	21/03/2016
**Q11 Maximum daily wind speed Davies Reef**	3	I looked at the long-term figures and figured anything below 60 in May was unlikely (but not out of the question).<br><br>http://data.aims.gov.au/aimsrtds/datatool.xhtml?from=2011-02-25&thru=2016-02-27&qc=LEVEL1&period=MONTH&aggregations=MAX&channel	21/03/2016
**Q11**	**2**	I've clearly misunderstood some aspect of the data in this question, haha. No secret insights about windspeed here. I'll likely have a rethink and adjust my answer upward in the next round	21/03/2016

**Q12 Average maximum air temperature**	3	I think I got drawn too much into the range, rather than the average (so my lower bound is perhaps too low).	21/03/2016
Q12	2	I don’t know much about Hamilton island but it did have some past lower temperatures if I remember. Seems like most of the group’s version of ‘low’ still sits above 20 so I’d probably adjust my lower bound upward	21/03/2016
**Q13 Turbidity High West** (New)	5	I have looked at historical turbidity data and know that turbidity can spike randomly - my take is that people's estimates are too narrow.	22/03/2016
**Q15 Zika Virus**	3	So, for upper bound to be 28 someone from each of those states must have travelled to an area with the Zika virus and been infected. I think that's possible? And I don't know what the detectability rate for the virus is.	21/03/2016
15	10	Disease spread is a function of disease density and vector density. Even if an infected traveller were to enter the EU there would need to be sufficient vectors around to facilitate spread. Vector numbers are likely to be low to the end of April (coming out of winter).	21/03/2016

15	2	My interpretation of this question is that you are looking for the number of member states with cases not the number of cases. Also, I understood that if someone caught this virus in the Americas but went back to say, the UK, where it was identified and treated, then this would not count as a case in the UK.<br><br>If this is the case, then almost everyone's answer is way too high, because only one EU country has islands with official EU member status that I know of in the Americas and the Pacific and that's France. In particular many people's high bounds are super high!<br>If I have misunderstood what counts and doesn't count and a case identified in the UK but originating somewhere else counts as a case for the UK, then I would adjust my estimates upwards by a few countries. But I still think most people's high bound estimates are way too high.<br><br>And I haven't heard of any cases where the virus has spread to other people in the EU from Europeans returning from overseas. If the group knows otherwise, I would like to hear about it and might adjust my answers slightly.	21/03/2016
Q15	Facilitator	This question was perhaps poorly worded. Let's be clear, we are only talking about cases which are caught in Europe. We are also only counting the number of member states that report a case which was caught in Continental Europe, NOT the number of people who catch Zika Virus.	21/03/2016
**Q16 Price of Gold**	3	http://www.sbcgold.com/blog/10-factors-regularly-influence-gold-prices/<br><br>http://www.theweek.co.uk/gold-price/61682/gold-price-hits-two-week-low-ahead-of-fed-meeting<br><br>http://www.bloombergview.com/quicktake/the-fall-of-gold<br><br>In summary. ARGH! It could rise dramatically, it might not. Depends (it seems) on a bunch of factors (see articles).	21/03/2016

Q16	10	Instability in other investments tends to redirect focus to gold and increase prices. Probably not much reason to expect a return to gold investment at the moment. Other than that, just guessing!	21/03/2016
**Q17 EU Referendum**	3	Jobs (certainty of) might favour leaving? I can't see that improving though. Does anyone else have an idea?<br><br>http://www.bbc.com/news/uk-politics-32793642<br>http://news.sky.com/story/1488832/eu-membership-reasons-for-and-against-leaving	21/03/2016
Q17	10	EU response to refugees might see a change - with a reduction in the number of Yes votes. General change inertia acting against it. Polls are also influenced by the weather.......	21/03/2016
**Q18 Twitter's stock price**	10	Unlikely that social media trends will change in the next 2 months and stocks likely to remain consistent with a longer term trend of a reduction in value of Twitter stock as people look for the next best social media investment.	21/03/2016
**Q19 Throughput from Abbot Point**	10	Even if there were world events that would reduce demand, I suspect that the lag time experienced between demand reduction and throughput that we might see in the timeframe we're being asked about means that trends are likely to remain consistent for now.	21/03/2016
**Q21 Space Launches**	10	Similar approach to that used by Participants 2 & 5! Looked at launch logs, looked at trends and averages, and guessed. Nasty weather patterns likely to be the main cause of fewer launches.	21/03/2016

Additional clarification and edits from Victoria.

Question 2: Number of reefs with at least 1% coral bleaching.

Clarification: For this question, we were interested in the number of reefs out of 24 reefs that would have at least 1% coral bleaching. As only 24 reefs were included, any value that you entered that exceeded this maximum was constrained to 24.

Correction:

Q2: Graph

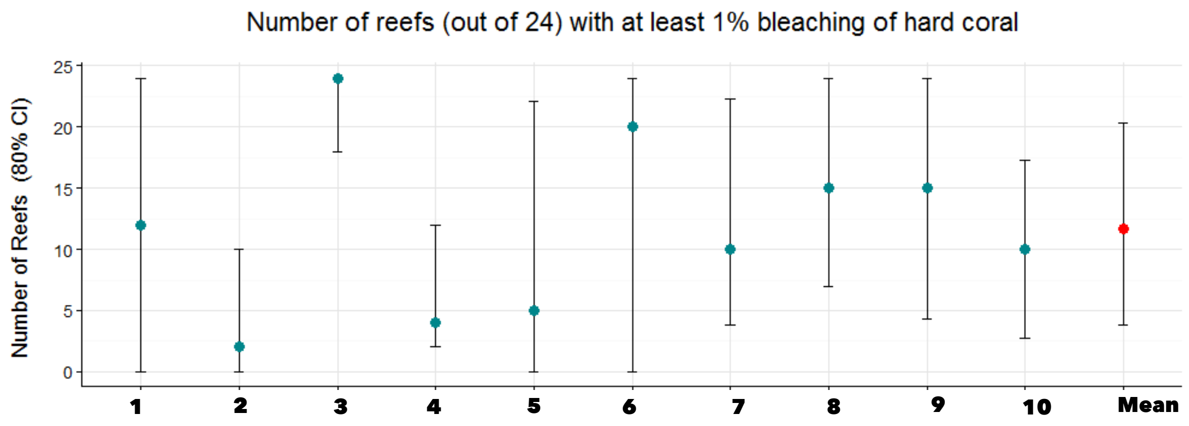

Q2: Table of results (constrained values)

	1	2	3	4	5	6	7	8	9	10	Mean
Best	12.00	2.00	24.00	4.00	5.00	20.00	10.00	15.00	15.00	10.00	11.70
Low	0.00	0.00	18.00	2.00	0.00	0.00	3.85	7.00	4.33	2.73	3.79
High	24.00	10.00	24.00	12.00	22.14	24.00	22.31	24.00	24.00	17.27	20.37

Question 8: Number of days the water temperature would be at least 28°C or more.

Clarification: For this question, we were interested in the number of days in April that the water temperature would exceed 28°C. As there are only 30 days in April any value that you entered that exceeded this maximum was constrained to 30.

Please also note, that we believe the graph provided in the question may have been misleading, this was not intentional, and an improved graph has been provided.

a. In 2015 there were 7 days where the water temperature was 28°C or more

b. Graph showing the days in April 2015 for which the water temperature was above 28°C

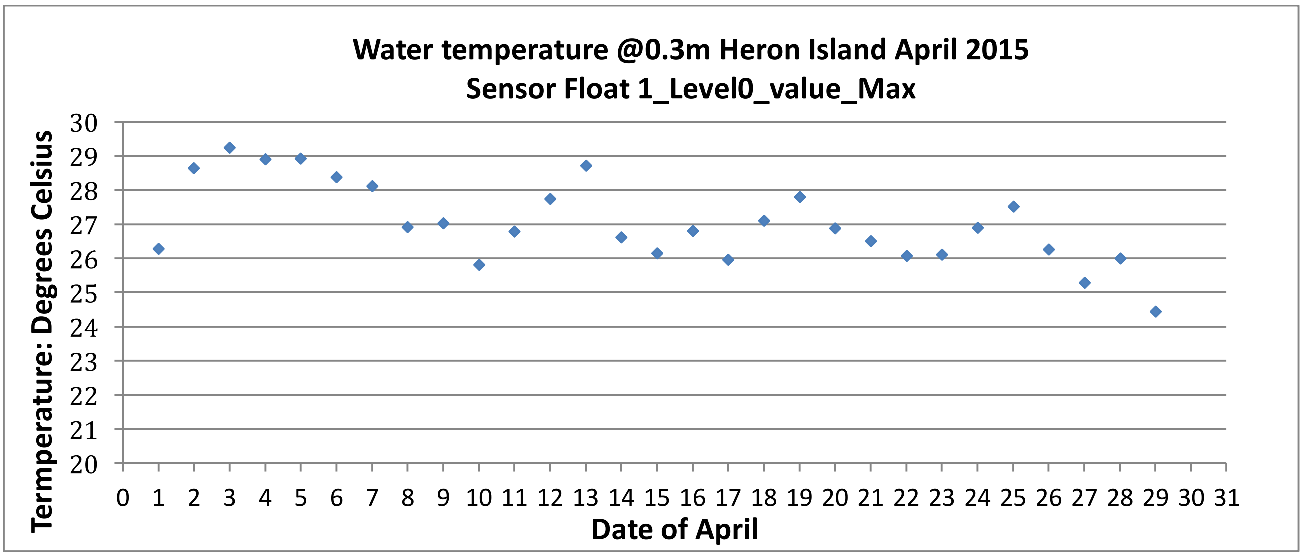

Correction: Please note for this question, in the original feedback sheets, the average was not reflective of constrained values. We have updated the feedback graphs and tables, sorry for the inconvenience.

Q8 Graph

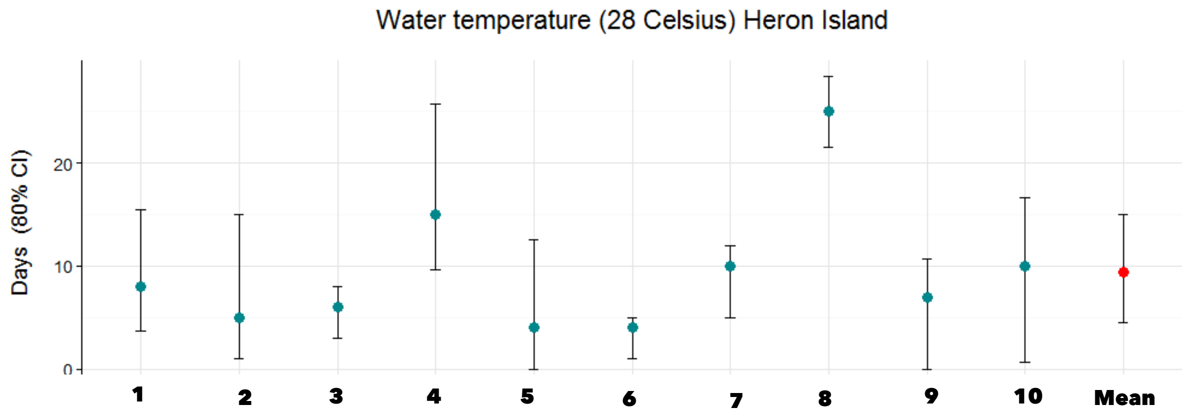

Q8 Table of results (constrained values)

	1	2	3	4	5	6	7	8	9	10	Mean
Best	8.00	5.00	6.00	15.00	4.00	4.00	10.00	25.00	7.00	10.00	9.40
Low	3.73	1.00	3.00	9.67	0.00	1.00	5.00	21.57	0.00	0.67	4.56
High	15.47	15.00	8.00	25.67	12.53	5.00	12.00	28.43	10.69	16.67	14.95

Question 15: Number of member states reported to have Zika Virus

Clarification: For this question, we were interested in the number of member states WITHIN continental Europe the World Health Organization will report as having at least one laboratory-confirmed human case of any strain of Zika virus for the month of April 2016. As only 28 member states are located within continental Europe any value that you entered that exceeded this maximum was constrained to 28.

Please note that the Virus must have been contracted WITHIN Europe- see the clarification in the question.

Correction: Please note for this question, in the original feedback sheets, the average was not reflective of constrained values. We have updated the feedback graphs, sorry for the inconvenience.

Q15 Graph

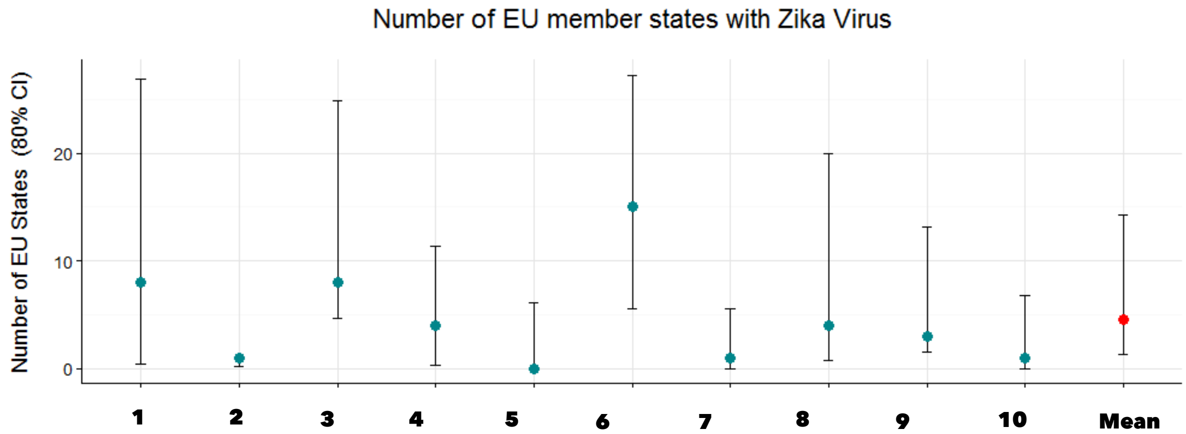

Q15 Table of results

	1	2	3	4	5	6	7	8	9	10	Mean
Best	8.00	1.00	8.00	4.00	0.00	15.00	1.00	4.00	3.00	1.00	4.50
Low	0.47	0.16	4.63	0.31	0.00	5.59	0.00	0.80	1.55	0.00	1.35
High	26.82	1.00	24.84	11.38	6.15	27.24	5.57	20.00	13.18	6.82	14.30

Question 17: Percentage of Votes in favour of the UK remaining in the EU

Clarification: This question is a percentage the maximum is 100%, any value that you entered that exceeded this maximum was constrained to 100%.

Correction: We don't think this has affected the group average for any group, however, we have fixed the glitch, and re-run the analysis for this question. We have updated the feedback graphs and tables, sorry for any inconvenience.

Q17 Graph

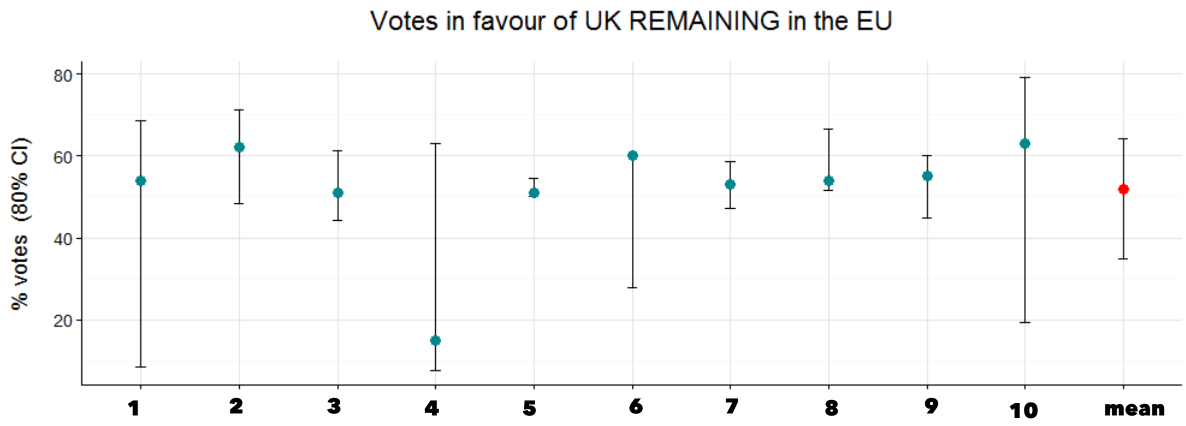

Q17 Table of results

	1	2	3	4	5	6	7	8	9	10	mean
Best	54.00	62.00	51.00	15.00	51.00	60.00	53.00	54.00	55.00	63.12	51.81
Low	8.67	48.29	44.14	7.73	50.11	28.00	47.29	51.71	45.00	19.36	35.03
High	68.67	71.14	61.29	63.00	54.56	60.00	58.71	66.57	60.00	79.23	64.32

Round 2 estimates: Great Barrier Reef Intelligence Game!

You have nearly reached the end of the Great Barrier Reef Intelligence Game! Well done. We cannot thank-you enough for your time and patience with this study.

**Code Name**	

**Were you present during the teleconference held on 1st March, 2016 before Round 1?**	

About this form:

This form provides an opportunity to adjust your estimates from Round 1.

Adjusting your estimates is completely optional, but given the questions and discussion surrounding many of the questions, we recommend you take a moment to reflect whether your first round answers provided realistic bounds, and whether your best guess needs to be adjusted in light of clarification and points raised by your group.

Additional documents you may require:

This document only provides space to enter your revised estimates. The following documents will help you to make your round 2 estimates.

1. Your estimates from Round 1.
2. The feedback provided by us to your group with results from Round 1.
3. The ongoing discussion document which is updated and emailed daily with new comments from your group.

Discussion:

Remember we have allowed additional time for discussion between your group. You will receive the final comments Tuesday 29th March 2016 (17:00) :

What if I don't revise my estimates?

Revising your estimates is completely optional. If you choose not to revise your estimates then your estimates from Round 1 will be used as your final estimates for each question.

We will accept your Round 2 estimates any time between now and **1pm Thursday 31st of March, 2016.**

Contents

Question 1: Average Density of Crown of Thorns at Rib Reef in 2016. 3
Question 2: How many of the 24 reefs listed will be detected to have at least 1% bleaching of hard corals? 4
Question 3: Number of detections of Asian Green Mussels. 5
Question 4: Number of Coral Colonies with White Syndrome on Reef 21060 6
Question 5: Commercial Catch of Coral Trout (tonnes). 7
Question 6: Marine Turtle strandings 1 January to 31 March, 2016?.................................8
Question 7: Sharks control in the Mackay region...9
Question 8: The number of days in April where the maximum water temperature will reach 28°C 10
Question 9: Total discharge volume Burdekin River, April 2016..11
Question 10: Chlorophyll levels detected at Pine Island March 2016................................12
Question 11: Maximum daily wind speed Davies Reef May 2016? 13
Question 12: Average maximum Air Temperature Hamilton Island? 14
Question 13: Mean turbidity (NTU) for High West in April 2016? 15
Question 14: El Nino events..16
Question 15: Zika Virus 17
Question 16: Price of Gold (USD)..18
Question 17: EU Referendum 19
Question 18: The stock price of Twitter 20
Question 19: The Throughput (tonnes) from Abbot Point Port 21
Question 20: People held in Nauru Regional Processing Centre, April 2016. 22
Question 21: Launches to space in May 2016? 23

Question 1: Average Density of Crown of Thorns at Rib Reef in 2016.

"What will be the average density of Crown of Thorns Starfish (Acanthaster planci) *detected per 2 minute manta-tow at Rib Reef, in the Townsville region, as surveyed by the Australian Institute of Marine Science (AIMS) as part of the Long-term Monitoring Program between 1 March, 2016 and 30 June, 2016 (inclusive)?"*

i.	Realistically, what do you think the **lowest** plausible value for the reported average density of CoTS, per 2 minute manta-tow, at Rib Reef will be?	

ii.	Realistically, what do you think the **highest** plausible value for the reported average density of CoTS, per 2 minute manta-tow, at Rib Reef will be?	

iii.	Realistically, what is your **best guess** for the reported average density of CoTS, per 2 minute manta-tow, at Rib Reef?	

iv.	**How confident are you** that your interval, from lowest to highest, could capture the reported density of CoTs at Rib Reef? Please enter a number between 50 and 100%	%

Question 2: How many of the 24 reefs listed will be detected to have at least 1% bleaching of hard corals?

"How many of the 24 reefs listed in Table 1 below will be reported with at least 1% bleaching of hard corals by the Australian Institute of Marine Science (AIMS) during SCUBA surveys undertaken between 1 March, 2016 and 30 June 2016 as part of the Long Term Monitoring Program (LTMP)?"

i.	Realistically, what do you think the **lowest** plausible value for the number of reefs reported with at least 1% bleaching of hard coral will be?	

ii.	Realistically, what do you think the **highest** plausible value for the number of reefs reported with at least 1% bleaching of hard coral will be?	

iii.	Realistically, what is your **best guess** for the number of reefs that will be reported to have at least 1% bleaching of hard coral?	

iv.	**How confident are you** that your interval, from lowest to highest, could capture the reported number of reefs with at least 1% bleaching of hard coral? Please enter a number between 50 and 100%	%

Question 3: Number of detections of Asian Green Mussels.

"How many unique detections of Asian Green Mussel (Perna viridis) *will be recorded by the Queensland Department of Agriculture and Fisheries between 1 March 2016 and 30 June 2016".*

i.	Realistically, what do you think the **lowest** plausible number of unique detections of Asian Green Mussels will be?	

ii.	Realistically, what do you think the **highest** number of unique detections of Asian Green Mussels will be?	

iii.	Realistically, what is your **best guess** for the number of unique detections of Asian Green Mussels?	

iv.	**How confident are you** that your interval, from lowest to highest, could capture the number of unique detections of Asian Green Mussels? Please enter a number between 50 and 100%	%

Question 4: Number of Coral Colonies with White Syndrome on Reef 21060

"What will be the total number of coral colonies reported with White Syndrome (a coral disease) on Reef 21060 in the Mackay-Pompey Region, by the Australian Institute of Marine Science (AIMS) during SCUBA surveys undertaken between 1 March and 30 June, 2016?"

i.	Realistically, what do you think the **lowest** plausible number of coral colonies detected with White Syndrome on Reef 21060 will be?	

ii.	Realistically, what do you think the **highest** plausible number of coral colonies detected with White Syndrome on Reef 21060 will be?	

iii.	Realistically, what is your **best guess** for the number of coral colonies that will be detected with White Syndrome on Reef 21060?	

iv.	**How confident are you** that your interval, from lowest to highest, could capture the actual number of coral colonies that will be detected with White Syndrome on Reef 21060? Please enter a number between 50 and 100%	%

Question 5: Commercial Catch of Coral Trout (tonnes).

"How many tonnes of Coral Trout will be caught in Queensland by the Commercial Line Fishery in April 2016?"

i.	Realistically, what do you think the **lowest** plausible catch (tonnes) of coral trout will be?	
ii.	Realistically, what do you think the **highest** plausible catch (tonnes) of coral trout will be?	
iii.	Realistically, what is your **best guess** for the catch (tonnes) of coral trout?	
iv.	**How confident are you** that your interval, from lowest to highest, could capture the reported catch (tonnes) of coral trout? Please enter a number between 50 and 100%	%

Question 6: Marine Turtle strandings 1 January to 31 March, 2016?

"How many turtles will be reported and confirmed as stranded by the Queensland Department of Environment and Heritage Protection for the whole of the Queensland East Coast between 1 January 2016 to 31 March 2016?"

i.	Realistically, what do you think the **lowest** number of turtles confirmed to be stranded will be?	

ii.	Realistically, what do you think the **highest** number of turtle confirmed to be stranded will be?	

iii.	Realistically, what is your **best guess** for the number of turtles that will be confirmed to be stranded?	

iv.	**How confident are you** that your interval, from lowest to highest, could capture the confirmed number of turtles reported to be stranded? Please enter a number between 50 and 100%	%

Question 7: Sharks control in the Mackay region

"How many individual sharks (target species only) will be caught by the Queensland shark control program in May 2016?"

i.	Realistically, what do you think the **lowest** number of sharks caught will be?	
ii.	Realistically, what do you think the **highest** number of sharks caught will be?	
iii.	Realistically, what is your **best guess** for the number of sharks that will be caught?	
iv.	**How confident are you** that your interval, from lowest to highest, could capture the number of sharks caught? Please enter a number between 50 and 100%	%

Question 8: The number of days in April where the maximum water temperature will reach 28°C

"<u>How many days</u> in April 2016 (30 days in total) <u>will the maximum water temperature reach 28.0° Celsius or above</u> at Heron Island?"

i.	Realistically, what do you think the **lowest** number of days the water temperature will reach 28.0° Celsius or above at Heron Island?	

ii.	Realistically, what do you think the **highest** number of days the water temperature will reach 28.0° Celsius or above at Heron Island?"	

iii.	Realistically, what is your **best guess** for the number of days the water temperature will reach 28.0° Celsius or above at Heron Island?"	

iv.	**How confident are you** that your interval, from lowest to highest, could capture the number of days the water temperature will reach 28.0° Celsius or above at Heron Island?" Please enter a number between 50 and 100%	%

Question 9: Total discharge volume Burdekin River, April 2016

"What will be the total discharge volume (Megalitres) for the Burdekin River, Queensland in April 2016?"

i.	Realistically, what do you think the **lowest** reported discharge volume (megalitres) from the Burdekin River will be?	

ii.	Realistically, what do you think the **highest** reported discharge volume (megalitres) from the Burdekin River will be?	

iii.	Realistically, what is your **best guess** for the reported discharge volume (megalitres) from the Burdekin River?	

iv.	**How confident are you** that your interval, from lowest to highest, could capture the reported discharge volume (megalitres) from the Burdekin River? Please enter a number between 50 and 100%	%

Question 10: Chlorophyll levels detected at Pine Island March 2016.

"What will be the average Chlorophyll level (ugL^{-1}) for Pine Island in the Mackay Whitsunday region in March 2016 recorded by the Wet Labs Eco FLNTUSB?"

i.	Realistically, what do you think the **lowest** average chlorophyll level (ugL-1) will be?	

ii.	Realistically, what do you think the **highest** average chlorophyll level (ugL-1) will be?	

iii.	Realistically, what is your **best guess** for the average chlorophyll level (ugL-1)?	

iv.	**How confident are you** that your interval, from lowest to highest, could capture the average chlorophyll level (ugL-1) for Pine Island? Please enter a number between 50 and 100%	%

Question 11: Maximum daily wind speed Davies Reef May 2016?

"What will be the highest maximum daily wind-speed (averaged maximum, km/hr) recorded for Davies Reef in May 2016?"

i.	Realistically, what do you think the **lowest** value for the highest maximum daily wind speed (averaged maximum, km /hr) will be?	
ii.	Realistically, what do you think the **highest** value for the highest maximum daily wind speed (averaged maximum, km /hr) will be?	
iii.	Realistically, what is your **best guess** for the value for the highest maximum daily wind speed (averaged maximum, km /hr)?	
iv.	**How confident are you** that your interval, from lowest to highest, could capture the maximum daily wind speed (averaged, maximum km /hr) for Davies Reef? Please enter a number between 50 and 100%	%

Question 12: Average maximum Air Temperature Hamilton Island?

"What will be the average maximum air temperature (°C) recorded by the Australian Bureau of Meteorology at Hamilton Island for the month of May, 2016?"

i.	Realistically, what do you think the **lowest** average maximum air temperature will be at Hamilton Island for May 2016?	

ii.	Realistically, what do you think the **highest** average maximum air temperature will be at Hamilton Island for May 2016?	

iii.	Realistically, what is your **best guess** for the average maximum air temperature at Hamilton Island in May 2016?	

iv.	**How confident are you** that your interval, from lowest to highest, could capture the average maximum air temperature at Hamilton Island in May 2016? Please enter a number between 50 and 100%	%

Question 13: Mean turbidity (NTU) for High West in April 2016?

"What will be the mean turbidity (NTU) for High West (located on High Island) for the month of April, 2016, as recorded by the Australian Institute of Marine Science using their ECO FLNTUSB instruments?"

i.	Realistically, what do you think the **lowest** mean turbidity (NTU) will be?	
ii.	Realistically, what do you think the **highest** mean turbidity (NTU) will be?	
iii.	Realistically, what is your **best guess** for the mean turbidity (NTU)?	
iv.	**How confident are you** that your interval, from lowest to highest, could capture the reported turbidity (NTU) ? Please enter a number between 50 and 100%	%

Question 14: El Nino events

"What will be the average sea-surface temperature (°C) for the month of June 2016 within the Nino 3.4 region as reported by the Climate Prediction Center of the National Oceanic and Atmospheric Administration (NOAA)?"

i.	Realistically, what do you think the **lowest** average sea-surface temperature will be?	

ii.	Realistically, what do you think the **highest** average sea-surface temperature will be?	

iii.	Realistically, what is your **best guess** for the average sea-surface temperature?	

iv.	**How confident are you** that your interval, from lowest to highest, could capture the reported sea-surface temperature? Please enter a number between 50 and 100%	%

Question 15: Zika Virus

"How many European Union member states (located in Continental Europe – 28 in total) will the World Health Organization report as having at least one laboratory-confirmed human case of any strain of Zika virus for the month of April 2016? Please note that the strain must have been contracted outside of the America's.*

i.	Realistically, what do you think will be the **lowest** number of EU member states reported to have at least one laboratory-confirmed human case of any strain of Zika virus for the month of April, 2016?	

ii.	Realistically, what do you think will be the **highest** number of EU member states reported to have at least one laboratory-confirmed human case of any strain of Zika virus for the month of April, 2016?	

iii.	Realistically, what is your **best guess** for the number of EU member states that will be reported to have at least one laboratory-confirmed human case of any strain of Zika virus for the month of April, 2016?	

iv.	**How confident are you** that your interval, from lowest to highest, could capture the number of EU member states will be reported to have at least one laboratory-confirmed human case of any strain of Zika virus for the month of April, 2016? Please enter a number between 50 and 100%	%

Question 16: Price of Gold (USD).

"What will be the closing spot price of gold (USD) on May 30 2016?"

i.	Realistically, what do you think the **lowest** spot price of gold will be?	

ii.	Realistically, what do you think the **highest** spot price of gold will be?	

iii.	Realistically, what is your **best guess** for the spot price of gold?	

iv.	**How confident are you** that your interval, from lowest to highest, could capture the reported spot price of gold? Please enter a number between 50 and 100%	%

Question 17: EU Referendum

"What will be the final percentage of votes made ***in favour*** *of the United Kingdom remaining a member of the European Union during the UK referendum to be held on 23 June, 2016?"*

i.	Realistically, what do you think the **lowest** percentage of people voting in favour of the UK remaining a member of the EU will be?	

ii.	Realistically, what do you think the **highest** percentage of people voting in favour of the UK remaining a member of the EU will be?	

iii.	Realistically, what is your **best guess** for the percentage of people voting in favour of the UK remaining a member of the EU?	

iv.	**How confident are you** that your interval, from lowest to highest, could capture the percentage of people voting in favour of the UK remaining a member of the EU? Please enter a number between 50 and 100%	%

Question 18: The stock price of Twitter

"What will Twitter's end-of-day stock price be on 30 May 2016?"

i.	Realistically, what do you think the **lowest** end-of-day stock price (USD) for twitter will be?	

ii.	Realistically, what do you think the **highest** end-of-day stock price (USD) for twitter will be?	

iii.	Realistically, what is your **best guess** for the end-of-day stock price (USD) for twitter will be?	

iv.	**How confident are you** that your interval, from lowest to highest, could capture the end-of-day stock price (USD) for twitter? Please enter a number between 50 and 100%	%

Question 19: The Throughput (tonnes) from Abbot Point Port

"What will be throughput (tonnes) from Abbot Point Port in May 2016 as reported by the North Queensland Bulk Ports Corporation?"

i.	Realistically, what do you think the **lowest** throughput (tonnes) from Abbot Point will be?	
ii.	Realistically, what do you think the **highest** throughput (tonnes) from Abbot Point will be?	
iii.	Realistically, what is your **best guess** for the throughput (tonnes) from Abbot Point?	
iv.	**How confident are you** that your interval, from lowest to highest, could capture the throughput (tonnes) from Abbot Point? Please enter a number between 50 and 100%	%

Question 20: People held in Nauru Regional Processing Centre, April 2016.

"How many people will be held in the Republic of Nauru Regional Processing Centre in April 2016?"

i.	Realistically, what do you think the **lowest** number of people will be?	
ii.	Realistically, what do you think the **highest** number of people will be?	
iii.	Realistically, what is your **best guess** for the number of people?	
iv.	**How confident are you** that your interval, from lowest to highest, could capture the actual number of people? Please enter a number between 50 and 100%	%

Question 21: Launches to space in May 2016?

"How many space launches will take place in May 2016?"

i.	Realistically, what do you think the **lowest** number of space launches will be?	
ii.	Realistically, what do you think the **highest** number of spaces launches will be?	
iii.	Realistically, what is your **best guess** for the number of space launches?	
iv.	**How confident are you** that your interval, from lowest to highest, could capture the number of space launches? Please enter a number between 50 and 100%	%

Feedback form.

Please use the following form to provide feedback on your experience in the Great Barrier Reef Intelligence Game.

1 *Firstly, was there anything that you <u>didn't you like</u> about the exercise?*

2 *Were there any aspects that you <u>did like</u>?*

3 *How did the process compare to other elicitations that you have been involved in?*

4 *To what extent were you influenced by the data and graphs included in each question? In other words, did you rely on the data provided or did you attempt to source new information?*

i. Please select > Other

ii. Please use the space provided to elaborate

5 *The method contains steps to help reduce the influence of a number of biases and heuristics that commonly affect expert judgement.*

These steps included:

- *Questions related to verifiable facts (to avoid subjective value judgements).*
- *The use of the four-point elicitation method (to reduce over-confidence).*
- *Individual private and anonymous estimates- (to reduce deference to authority).*

- *Allowing discussion between participants between rounds (to clarify linguistic uncertainty, encourage critical thinking, and to consider counterfactual evidence).*
- *Mathematical aggregation- (to enable empirical control over the final aggregation).*

i. Do you think the above steps helped to reduce biases and heuristics? ***Please select >***

ii. Please use the space provided to elaborate

6 ***Please provide any additional comments / suggestions.***

Thank-you!

THE UNIVERSITY OF MELBOURNE

An Invitation to the Great Barrier Reef Intelligence Game- March 2016

F 18, 2017 at 12:25 PM

Dear %First Name%,

We were provided your name and contact details by %Peer Name%, as someone who should be involved in a project we are about to launch.

On the 1st March 2016 we will be commencing the "Great Barrier Reef Intelligence Game". We would really like you to be involved in this study. You will learn about eliciting expert judgement, and about the strengths and limitations of your own judgement.

We apologise in advance for the short notice. If you would like to participate but cannot make the teleconference we can arrange to bring you up-to-date with the content covered in the teleconference.

What is involved? The project will involve you, using your expert judgement to answer 20 questions about predictions of abiotic and biotic events on the Great Barrier Reef. Just to make things interesting we've also included some questions about geopolitical events. All of these events will be verified by March 2017, so you will be able to compare your estimates to the truth. More detail about what is involved is outlined in the Plain Language Statement (attached) and will be explained further during a short teleconference on the 1st of March, 2016.

Why we need you? We have had strong interest in the study to date. Thirty marine ecologists from Australia and around the globe have registered to participate. We've also have a group of intelligence agents from the Australian Department of Defence interested in participating. We're interested in the characteristics of better performing experts, and to that end, we need broad involvement across a range of experience and qualifications. We would like you (and any peers you can recommend) to partake in the survey and bring your working knowledge (no matter how scant) of the Great Barrier Reef to the survey.

Can we include you as a participant? Please reply indicating your interest by return email by **Monday 29th February.**

Many thanks

Victoria Hemming (PhD candidate) contact details omitted

Terry Walshe (supervisor) contact details omitted

Mark Burgman (supervisor) contact details omitted

Victoria Hemming

PhD Candidate
CEBRA
School of BioSciences
University of Melbourne
Parkville, 3010

---

2 attachments

Great Barrier Reef Intelligence Games_Participant consent_Pro Version_distributed.pdf
293K

Plain Language.pdf
151K

Teleconference Details: The Great Barrier Reef Intelligence Game 11:00 (UTC+11) Tuesday 1 March 2016

Fri, F 26, 2017 at :08 PM

Hi %First Name%,

Below are the details you will need to follow in order to join the teleconference on Tuesday 11am (Melbourne time, UTC+11).

We hope you can make it for at least the first 30 minutes as it is a really important part of the study.

A few things to note about Tuesday's teleconference:

There will be at least 30 other participants also joining in on the teleconference. To make it run smoothly we have set the following agenda:

- **10:55-11:05-** Participant dial in.
- **11:05-11:30-** Mark, Terry and myself will introduce the project, and explain its importance. Mark will also explain the method, and provide a few tips on how to approach the questions to get your best possible score.
- **11:30-** We will answer any questions**.** You can leave at this point if you need to.

If after the teleconference you have any further comments or questions that we could not address, please do not hesitate to contact me.

Please try to submit your consent form prior to the teleconference.

Kind regards,

Victoria, Terry and Mark.

Teleconference instructions

· Between 10:55am and 11:00am, dial your dial-in number as listed below

· When prompted, enter your conference pass code:**details omitted**

· If asked for a name, either skip, or enter the code name you supplied us in your consent form.

Please note your toll-free dial-in number to participate in the conference call:

<Contact details omitted>

If you are calling from outside one of the above listed numbers please let me know so that I can arrange for you to dial in.

--
Victoria Hemming

PhD Candidate
CEBRA
School of BioSciences
University of Melbourne
Parkville, 3010

Round 1 of the Great Barrier Reef Intelligence Games! Your Questions

, Mar 1, 2016 at 5:21 PM

Dear <<Code_Name>>

Let Round 1 of the Great Barrier Reef Intelligence Game begin!

Attached is:

1. A PDF form which includes:

- Instructions & rules: You should read this (particularly if you could not make the teleconference)
- The amazing 21 questions.

2. An EXCEL spreadsheet which you can use to visualise how your intervals will be extrapolated in Round 2.

Remember:

To save the document as you go along.

Please do not speak with other participants about your estimates.

And do not hesitate to contact Victoria if you have any additional questions or comments.

Best of luck!

You have until 17:00 on 10 March to return your estimates.

Victoria, Mark and Terry

--
Victoria Hemming

PhD Candidate
CEBRA
School of BioSciences
University of Melbourne
Parkville, 3010

2 attachments

Round 1 Questions for the Great Barrier Reef Intelligence Game March_distributed.pdf
983K

Practice Feedback.xlsx
59K

The University of Melbourne

Important: Update to Question 13 and additional clarification: Round 1 GBR Intelligence Game

, Mar 2, 2016 at 1:1 PM

Hi %Code_Name%

Just to let you know, we have had to change the monitoring point referred to in Question 13. Please note, that Question 13 now relates to High West, on High Island (not Snapper North)

We have also responded to some questions (these can be found below). All amendments relating to questions received overnight have been now included in the updated survey form (attached).

Here's what to do:

> **If you have already begun:** please continue to complete your existing form taking note that Question 13 now relates to "High West", a sampling location on High Island (not Snapper North). Please also note the minor amendments and clarification that has been added to the UPDATED document (attached)- these are listed below.
>
> **If you have not begun:** I've updated the survey form with the below amendments. Please use the survey form attached "Round 1 (UPDATED)" instead of the form sent yesterday. Please also see the clarifications that have been provided at the end of this email.

Apologies in advance for any inconvenience this might cause.

My aim is to keep emails to a minimum, and I'll avoid changing the form again.

If you have any more questions please let me know.

Otherwise....Happy estimating, and speak soon.

Kind regards, Victoria.

Amendments made 02/03/2016:

Question 13: The question cannot be answered, so needs to be changed.

Question 13 now relates to a new location **"High West"** (a sampling point on High Island) *instead* of **"Snapper Reef North"**. This is because the instruments on Snapper Reef North are no longer deployed. As the games have only just begun, we have revised the question. The question now asks:

"What will be the mean turbidity (NTU) for High West (located on High Island) for the month of April, 2016, as recorded by the Australian Institute of Marine Science using their ECO FLNTUSB instruments?"

The below table shows the turbidity readings (NTU) for High West on from 2007 - 2014.

Monitoring period	Annual Mean Turbidity (NTU)	Number of sampling days
Oct 2007 – Sep 2008	0.81	356
Oct 2008 – Sep 2009	0.84	365
Oct 2009 – Sep 2010	1.20	365
Oct 2010 – Sep 2011	1.56	365
Oct 2011 – Sep 2012	1.08	366
Oct 2012 – Sep 2013	1.55	365
Oct 2013 – Sep 2014	1.27	213
Oct 2014 – Sep 2015	1.74	169

If you are using the original document sent yesterday please, continue, but enter your estimates for High West in the spaces provided for Question 13.

Question 14: Clarification has been sought as to what this question is asking.

This question seeks to understand what you think the average sea-surface temperature will be for the Month of June 2016 over the Nino 3.4 region.

We have added information: "*b. The average sea-surface temperature for June 2015 was 28.70 °C*"

Question 15: Links appear to be broken

Please try these links:

i. World Health Organisation Reports for Zika virus: http://www.who.int/csr/don/archive/disease/zika-virus-infection/en/

ii. List of 28 member countries for the EU: http://europa.eu/about-eu/countries/member-countries/index_en.htm

iii. News article about Zika virus: http://www.reuters.com/article/us-health-zika-idUSKCN0V523W

Additional comments:

Error- Page 3: Section: "Think about your intervals". Note the last sentence should read: *It means you should be willing to bet your house or job on it because there is no plausible way the truth could be located outside of your intervals.*

Question- What is the EXCEL spreadsheet for? The excel spreadsheet is for the super keen, it's a one page document, which will show you how your estimates will appear when you plug in your estimates for a question. It does not need to be completed.

Which questions do I absolutely have to answer? You need to answer all 21 questions, and complete at least your four estimates for each of them. I've now made this clear in the UPDATED pdf (attached), the required questions are now highlighted in red. We hope you will find time to add any comments or additional knowledge in the provided spaces.

I need a map: Please note, most places in question can be accessed through the links provided, or by entering the location into Google Maps.

What is my code name?: You were requested to create a unique code name in your consent form. The name you entered is the name I've used to address this email to, and how you will be referred to during the Intelligence Game. This name will be the name visible to other participants, so we suggest you make it something recognisable by you but not others. If you would like to change your code name, you can do this in the survey form (a space has been provided).

Formatting: Note I've also added a blank page to the document after the instructions. This was to improve

readability for you. It now means if you open the PDF up, and view two pages side by side, you will be able to see the question and the answer pages, for the one question side by side.

--
Victoria Hemming

PhD Candidate
CEBRA
School of BioSciences
University of Melbourne
Parkville, 3010

Round 1 (UPDATED) Questions for the Great Barrier Reef Intelligence Game March_distributed.pdf
981K

Due this Thursday: Round 1 estimates Great Barrier Reef Intelligence Game.

M , Mar , 2016 at 12:28 PM

Hi %Code Name%

I hope you had a wonderful weekend.

A gentle reminder that your estimates for Round 1 of the Great Barrier Reef Intelligence Game are **due this Thursday, 10 March at 5 pm.** The form to be completed was sent in a previous email last week, and is also attached below.

Please let me know if you anticipate any trouble completing the form by this deadline.

I'm happy to answer any queries you might have.

Kind regards,

--
Victoria Hemming

PhD Candidate
CEBRA
School of BioSciences
University of Melbourne
Parkville, 3010.

Round 1 (UPDATED) Questions for the Great Barrier Reef Intelligence Game March_distributed.pdf
981K

Round 1 Complete! Thank you!

M , Mar 14, 2016 at 8:0 PM

Dear <Code_Name>

Round 1 of the Great Barrier Reef Intelligence Game is now complete!

Mark, Terry and I, would like to thank-you for taking part. Whilst some people thought it was a bit of fun, we understand that the survey had some complex questions which required some thinking, and also required you to take time from your ever expanding workload, and your own personal commitments. We really appreciate you making the time to have a go, and we look forward to sharing the results of the survey, and some lessons learnt.

I'm currently crunching the numbers for Round 1 and compiling them into an easy to read format. There is a slight delay, however, I will aim to have your feedback to you by this Wednesday 5 pm (Melbourne Time).

I'm hoping you will appreciate the time out, but please let me know if this inconveniences you in the slightest and I'll see what I can do.

Speak soon!

--

Victoria Hemming

PhD Candidate
CEBRA
School of BioSciences
University of Melbourne
Parkville, 3010.

Are you still interested in being involved in the GBR Intelligence Game?

Fri, Mar 11, 2016 at 5:02 PM

Hi %Code_Name%

A few people have requested extensions. So the deadline for submission of the GBR Intelligence Game has been extended until this **Sunday 13 March at 11:59 pm (Melbourne time)**

We are really keen to have you involved in the survey, and to get your response so if you're still able to submit your form to me over the weekend that would be excellent.

Unfortunately, I do need to run the analysis on Monday so I won't be able to accept submissions past this date.

Please let me know if I should wait for your response.

Kind regards,

--
Victoria Hemming

PhD Candidate
CEBRA
School of BioSciences
University of Melbourne
Parkville, 3010.

THE UNIVERSITY OF MELBOURNE

A query about one of your responses.

Sun, Mar 1 , 2016 at 10: 0 M

Hi %Code_Name%,

Thanks for your form. I just have a query about one of your responses. For Question 19, you entered 2000, 8000, and 5000 for the throughput in tonnes from Abbot Point in Queensland. Did you mean this be 2 000 000, 8 000 000, 5 000 000?

Or are you predicting that the throughput for this year will be several orders of magnitude smaller than previous years?

Please let me know if you would like me to amend.

Kind regards.

--
Victoria Hemming

PhD Candidate
CEBRA
School of BioSciences
University of Melbourne
Parkville, 3010.

THE UNIVERSITY OF MELBOURNE

Results of Round 1 of the GBR Intelligence Game 2016

Fri, Mar 18, 2016 at 12:44 PM

Dear %Code_Name%

Your preliminary results are in!   Thank you for your patience. You now have until 9am next Wednesday (Melbourne time) to discuss your responses with your allocated group.

Can you please:

- Look over your group results and comments in the attached form
- Email Victoria any comments or questions you have for your group. This can also be done using the interactive boxes of the attached form.
- Victoria will collate your comments on a daily basis and send to the group for further discussion.
- The discussion ends at 9am Wednesday 23 March 2016 (Melbourne time).
- **Please try to send through one or two comments before then.**

You will then be provided with an opportunity to revise your estimates (Wednesday 23 March 2016 - Wednesday 30th March 2016).

About the analysis so far:

Your original estimates have been standardised to 80% confidence intervals. This means your best guess should align with what you provided in Round1, but your lower and upper intervals may vary. Please let me know if any of your results are erroneous.

For some questions there was an upper limit- for example question 2 and 9. For these questions if your estimates exceed the upper limit your answers were capped at the upper limit. I’ve just realised that the average was not adjusted accordingly.

All data has been presented to 2 decimal places. I’ll try to correct this for the final estimates.

Have fun! And feel free to email me as regularly as you would like to contribute your comments and

questions to the discussion.

--
Victoria Hemming

PhD Candidate
CEBRA
School of BioSciences
University of Melbourne
Parkville, 3010

Round 1 Feedback_Group2_Form_distributed_0002.pdf
2263K

Today's discussion and clarification from your group.

M , Mar 21, 2016 at :42 PM

Dear %Code_Name%,

We've received some more comments from members in your group. These have been added to any comment received over the past few days and are attached.

Some of these comments may be directly asking you a question, so please check them.

New comments will be highlighted with a star.

Please try to make any further comments by tomorrow, before the discussion phase ends.

In addition, I've just realised that something strange happened to the group average for question 2, 8, 15, and potentially 17. I've corrected the mistake in my code. Whilst this generally does not affect your individual estimates (unless you provided very large estimates above the plausible maximum values) it may affect the group average you saw for these questions . I've re-printed the tables and graphs in the attached form for your information.

Please remember to email me any comments or replies.

Kind regards,
--
Victoria Hemming

PhD Candidate
CEBRA
School of BioSciences
University of Melbourne
Parkville, 3010.

Group 8 Discussion and clarification.pdf
187K

Extension to the discussion phase.

, Mar 22, 2016 at 6:22 PM

Hi %Code_Name%

The discussion phase is going well. However, some people have felt that it is too short, and they would like it to be extended.

I've spoken to Mark, and he believes it is important for the group to have the time it needs. However, we also note that you have been exceedingly generous with your time thus far. Therefore we propose the following:

Round 2 forms will still be sent out tomorrow. These can be submitted any time from tomorrow, until the close of Round 2.

The discussion phase will be extended for those who are keen to keep going. **Please note it is completely optional** whether you choose to continue to partake in the discussion. I completely understand if you are wanting to get away for Easter and finish up the survey prior to Good Friday, in which case you can submit your answers from tomorrow.

New milestones (Melbourne Time)

Wednesday 23 March (12:00): Round 2 forms sent out for you to revise your estimates.
Tuesday 29th March, 2016 (9:00): Final comments must be received.
Tuesday 29th March 2016 (17:00) : Final set of comments sent out for your group.
Thursday 31st March 2016 (13:00): Round 2 closes, Final estimates due.

I'd like to thank you for your efforts so far, and please let me know if you have any concerns about the extension.

--
Victoria Hemming

PhD Candidate
CEBRA
School of BioSciences
University of Melbourne
Parkville, 3010.

New comments received from your group or the facilitator.

Sun, Mar 20, 2016 at :1 PM

Dear %Code_Name%

You have new comments for discussion from your group or the facilitator.

Don't forget to make any comments about the Round 1 results in the next day or two before the discussion phase ends.

You can make comments, by emailing me directly, or adding to the Round 1 results.

Kind regards,

--
Victoria Hemming

PhD Candidate
CEBRA
School of BioSciences
University of Melbourne
Parkville, 3010.

Group 1 Additional Comments.docx
96K

THE UNIVERSITY OF MELBOURNE

Round 2: You can now revise your estimates!

, Mar 2 , 2016 at 12: 4 PM

Dear %Code_Name%,

Round 2 has commenced.

Attached is a form for you to revise your Round 1 estimates in light of new information, clarification and ongoing discussion. Remember this is optional, but highly recommended.

Also attached is the latest comments from your group (to 22/03/2016).

Revising your estimates:

When revising your estimates we recommend you reflect on the following documents:

1. Your estimates from Round 1
2. The feedback provided by us to your group with results from Round 1
3. The ongoing discussion document which is updated and emailed daily with new comments from your group (the latest document for your group is attached).

Submission:

Final submissions must be received by Thursday 31st March 1pm (Melbourne Time)

Discussion:

Be aware that discussion for your group will continue until Tuesday 29th March, 2016 (5pm Melbourne time).

Comments and suggestions for improvement:

Following the closure of Round 2 we will send out your final results. We will also send out a feedback form to get your thoughts on the study and the method. So please make a note of anything that you'd liked or has frustrated you.

--
Victoria Hemming

PhD Candidate
CEBRA
School of BioSciences
University of Melbourne
Parkville, 3010.

2 attachments

Round 2 estimates_form_distributed.pdf
290K

Group 5 Discussion and clarification.pdf
225K

Discussion phase complete! An extension for your final estimates. THANK YOU.

, Mar 0, 2016 at 12:42 PM

Dear 71789,

The discussion phase is now complete. There were no new comments from your group.

Please review your Round 1 estimates and adjust accordingly using the form attached. This will mark the end of your involvement in the study.

An extension is available:

A few people have requested a small extension to submit their final estimates. I really appreciate your commitment to this study, which has been above and beyond.

I've therefore extended the final deadline to Monday 4th April at 11:59 pm (Melbourne time). I hope this will provide you with the necessary time to reflect on new information and adjust your initial estimates.

One word of caution with this extension- it is possible that some of the questions will be resolved by Monday. If this occurs, then your Round 1 estimates will need to be used for these questions. Hopefully, this will not occur (my main concern is Question 6).

If you are having trouble meeting this new deadline, please let me know.

I want to thank you sincerely for your participation, and I look forward to sharing the final results with you.

Kind regards,

--
Victoria Hemming

PhD Candidate
CEBRA
School of BioSciences
University of Melbourne
Parkville, 3010.

Round 2 estimates_formV2_distributed.pdf
290K

THE UNIVERSITY OF MELBOURNE

Final estimates due tonight.

M , r 4, 2016 at :14 PM

Dear 23631,

My records show that I have not received your final estimates for the GBR Intelligence game. A gentle reminder that any revised estimates are due tonight: 11:59 pm Melbourne time (in approximately 15 hours).

Revising estimates?

To revise estimates please use the form attached. If there are any questions for which you do not want to revise your estimates, you can leave the spaces blank.

No changes to be made to any questions?

If you have no changes to be made to ANY of your Round 1 estimates, please confirm you are happy for me to use all of your initial estimates made in Round 1 as your final answer. Please make sure you have checked the feedback form for your group first.

This is the final task of this survey and will mark the end of the GBR Intelligence Game. I'd like to thank you for your involvement and interest in this research.

Kind regards,

--
Victoria Hemming

PhD Candidate
CEBRA
School of BioSciences
University of Melbourne
Parkville, 3010.

Round 1 Feedback_Group1_Updated.pdf
4414K

Round 2 estimates_formV2_distributed.pdf
290K

The GBR Intelligence game- demographic data.

, Ma 26, 2017 at 4:26 PM

Hi %Code_Name%

I hope you are well, I just have a quick question for you.

I'm tidying up the demographic data that was collected as part of the Great Barrier Reef Intelligence Game. **Could you please confirm:**

1. Your NATIONALITY

2. Your current COUNTRY of residence.

3. Your native LANGUAGE / S

This data will be used to provide an overview of the diversity of people who took part in the survey.

Also, a quick update, two of the questions have been resolved, with more to be resolved in the next fortnight. I will send through some results in the coming weeks. Exciting times.

Kind regards,

--
Victoria Hemming

PhD Candidate
CEBRA
School of BioSciences
University of Melbourne
Parkville, 3010.

THE UNIVERSITY OF MELBOURNE

Round 2 Results. Great Barrier Reef Intelligence Game.

Fri, 2 , 2016 at :45 PM

Dear %Code_Name%,

Attached is a report of the results so far. Thank you again for taking part.

Unfortunately, five questions are still to be resolved. I will send through an update if and when these questions are resolved.

In the next week I also will send a feedback form which will allow you to provide me with any thoughts or comments in regard to your involvement with this elicitation.

Once again, thank-you for taking part in what was a time consuming but hopefully thought provoking task. If I can assist you at all with your own research please let me know.

Kind regards

--
Victoria Hemming

PhD Candidate
CEBRA
School of BioSciences
University of Melbourne
Parkville, 3010.

Group 3 report.pdf
819K

Your Feedback + Updates

, , 2016 at 1: 8 PM

Dear %Code_Name%,

You may have picked up a slight anomaly in the results for Question 8 regarding windspeed. And you would be right!

This was due to a bug in the database which mislabelled the data columns. I've now amended this. In the process, I realised that three questions had been excluded from the scoring. I've updated the reports (attached).

Please do not take the results too seriously, the aim was to improve the method for expert elicitation not to judge your expertise- hence the diverse array of questions. In addition, the results are preliminary, and just an overview.

Please send me your feedback! (attached)

I would really love **YOUR feedback** on your experience with the method (what you liked, what you hated, how it could be improved), even if you were not able to complete both rounds. Your comments (no matter how brief) are completely anonymous, and will be used to help improve the method.

--
Victoria Hemming

PhD Candidate
CEBRA
School of BioSciences
University of Melbourne
Parkville, 3010.

Feedback Form Great Barrier Reef Intelligence Game_distributed.pdf
126K

Group 8 report.pdf
811K
