# Supplementary material for: Eliciting improved quantitative judgements using the IDEA protocol: A case study in natural resource management
Source: PLoS One. 2018 Jun 22;13(6):e0198468. doi: 10.1371/journal.pone.0198468 (PMC6014637; doi:10.1371/journal.pone.0198468)
Supplement: S3 File — (PDF) [file pone.0198468.s003.pdf]

# Analysis of Great Barrier Reef Elicitation Data 2016

## Table of Contents


**Demographic data** .......... **2**
Sourced .......... 2
Peer recommendation .......... 2
Gender .......... 2
Occupation .......... 2
Age .......... 4
Salutation .......... 4
Updated Estimates .......... 4
Nationality .......... 5
First Language .......... 5
Sector .......... 5

**Participant responses** .......... **7**
Graphs for each question .......... 7

**All 76 participants Round 1 and Round 2** .......... **22**
**Round 1: Demographics and performance** .......... **23**
Round 1: Sector vs Accuracy, Calibration, Informativeness .......... 23
Round 1: Age vs Accuracy, Calibration, Informativeness .......... 24
Round 1: Professional Experience vs Accuracy, Calibration, Informativeness .......... 25
Round 1: Committee Membership vs Accuracy, Calibration, Informativeness .......... 26
Round 1: Profession of Expert advice vs Accuracy, Calibration, Informativeness .......... 27
Round 1: Salutation vs Accuracy, Calibration, Informativeness .......... 28
Round 1: Sourced vs Accuracy, Calibration, Informativeness .......... 29
Round 1: First language vs Accuracy, Calibration, Informativeness .......... 31
Round 1: Nationality vs Accuracy, Calibration, Informativeness .......... 32
Round 1: Peer recommendation vs Accuracy, Calibration, Informativeness .......... 33
Round 1: Publications vs Accuracy, Calibration, Informativeness .......... 34
Round 1: Gender vs Accuracy, Calibration, Informativeness .......... 35
Round 1: Updated vs Accuracy, Calibration, Informativeness .......... 36
**Linear Models** .......... **37**
Number of Publications .......... 37
Self-rating and accuracy in a domain (Round 1) .......... 44
Self-rating vs years of experience .......... 48
**Round 2: Demographics and performance** .......... **50**
Round 2: Updated vs Accuracy, Calibration, Informativeness .......... 50
**Correlation between Round 1 and Round 2** .......... **51**
**Change in Accuracy** .......... **53**

**Change in calibration (distance from 0.80) vs direction of change** .......... **53**

**All 76 participants: Group vs individual performance** .......... **55**

**58 Participants: Group vs individual performance (removing those who withdrew)** **59**
**Changes in accuracy + thresholds** .......... **64**

## Demographic data

*Table A1: How participants were sourced and their participation in each stage of the elicitation*

| Sourced | Invited | ConsentForm | Round1 | Round2 |
|---|---|---|---|---|
| Advertisment | 10 | 8 | 7 | 4 |
| Conference | 42 | 21 | 16 | 14 |
| Participant recommendation | 150 | 29 | 18 | 13 |
| Professional Networks | 82 | 36 | 30 | 23 |
| Project Assistance | 21 | 7 | 5 | 4 |
| Total | 305 | 101 | 76 | 58 |

*Table A2: Whether participants were recommended as Experts or Novices and their participation in each stage of the elicitation*

| Peer-recommendation | Invited | ConsentForm | Round1 | Round2 |
|---|---|---|---|---|
| Not-recommended | 159 | 71 | 57 | 44 |
| Expert | 92 | 22 | 13 | 9 |
| Novice | 54 | 8 | 6 | 5 |
| Total | 305 | 101 | 76 | 58 |

*Table A3: Gender of participants and their participation in each stage of the elicitation*

| Gender | Invited | ConsentForm | Round1 | Round2 |
|---|---|---|---|---|
| Women | NA | 47 | 36 | 27 |
| Men | NA | 54 | 40 | 31 |
| Total | NA | 101 | 76 | 58 |

*Table A4: Occupation of participants their participation in each stage of the elicitation*

| Title | ConsentForm | Round1 | Round2 |
|---|---|---|---|
| Academic | 2 | 1 | 1 |
| Assoc Prof / Prof. | 2 | 2 | 2 |
| Associate Research Fellow | 1 | 1 | 1 |
| Ballast water & hull fouling coordinator | 1 | NA | NA |
| Benthic ecologist | 1 | 1 | 1 |
| Biologist and technical adviser | 1 | 1 | 1 |
| Conservation Scientist | 1 | NA | NA |
| Dean of Science | 1 | NA | NA |
| Director of Government Relations | 1 | NA | NA |

| | | | |
|---|---|---|---|
| Ecologist | 1 | 1 | 1 |
| Environmental consultant | 1 | 1 | 1 |
| Environmental Scientist | 1 | NA | NA |
| Financial Controller | 1 | 1 | NA |
| Fisheries Consultant | 1 | 1 | 1 |
| Fisheries Scientist | 3 | 2 | 2 |
| Fisheries Technologist | 1 | 1 | 1 |
| Fisherman | 1 | NA | NA |
| Fishery Manager | 1 | 1 | 1 |
| GBR Manager (Social & Economic Sciences) | 1 | NA | NA |
| Industry Engagement | 1 | NA | NA |
| Lecturer | 1 | 1 | NA |
| Manager | 1 | NA | NA |
| Marine Biologist | 1 | 1 | NA |
| Marine ecologist | 2 | 2 | 2 |
| Marine Incursion Investigator | 1 | 1 | 1 |
| Marine Park Manager | 1 | 1 | 1 |
| Marine Scientist | 3 | 2 | 2 |
| Physicist | 1 | 1 | 1 |
| Policy Officer | 1 | 1 | 1 |
| Post-graduate Student | 9 | 9 | 7 |
| Postdoc | 5 | 3 | 2 |
| PostDoc marine biologist | 1 | 1 | 1 |
| Principal Scientist | 1 | NA | NA |
| Professor of Philosophy | 1 | 1 | 1 |
| Program Manager | 1 | NA | NA |
| Public Servant | 2 | 2 | 2 |
| Research Assistant | 1 | 1 | NA |
| Research consultant | 1 | NA | NA |
| Research Fellow | 3 | 3 | 2 |
| Research Manager | 1 | 1 | 1 |
| Research Officer | 1 | NA | NA |
| Research Scientist | 5 | 3 | 2 |
| Researcher | 4 | 4 | 1 |
| retired | 1 | 1 | 1 |
| Science consultant | 1 | 1 | 1 |
| Scientific researcher | 1 | 1 | NA |
| Scientist | 6 | 4 | 3 |
| Senior Biosecurity Officer | 1 | 1 | 1 |
| Senior Lecturer | 2 | 2 | 1 |

| | | | |
|---|---|---|---|
| Senior Management | 6 | 4 | 3 |
| Senior Policy Officer | 2 | 2 | 1 |
| Senior Research Scientist | 1 | 1 | 1 |
| Senior Scientist | 2 | 2 | 2 |
| Strategic Reform Policy Officer | 1 | 1 | 1 |
| Trainee | 1 | 1 | 1 |
| University lecturer | 1 | 1 | NA |
| Veterinarian, Aquatic Researcher | 1 | NA | NA |
| Veterinary Epidemiologist | 1 | 1 | 1 |
| Wildlife Disease Specialist | 1 | 1 | 1 |
| Total | 101 | 76 | 58 |

*Table A5: Age of participants and their level of participation*

| Age | ConsentForm | Round1 | Round2 |
|---|---|---|---|
| 21-30 | 11 | 8 | 4 |
| 31-40 | 35 | 28 | 23 |
| 41-50 | 20 | 13 | 10 |
| 50-60 | 28 | 21 | 16 |
| 60+ | 7 | 6 | 5 |
| Total | 101 | 76 | 58 |

*Table A6: Salutation of participants and their level of participation*

| Salutation | ConsentForm | Round1 | Round2 |
|---|---|---|---|
| Dr. / Prof. | 59 | 46 | 38 |
| Miss / Ms / Mrs | 21 | 17 | 11 |
| Mr | 20 | 13 | 9 |
| Other | 1 | NA | NA |
| Total | 101 | 76 | 58 |

*Table A7: Level of  participants and gender of participants*

| | 1.Updated | 2.Reviewed | 3.Withdrew |
|---|---|---|---|
| **Female** | 21 | 6 | 9 |
| **Male** | 24 | 7 | 9 |
| **Total** | 45 | 13 | 18 |

*Table A7: Participation and nationality of participants*

| Nationality | Round1 | Round2 |
|---|---|---|
| American | 4 | 4 |
| Australian | 38 | 29 |
| Canadian | 7 | 6 |
| Chilean | 1 | 1 |
| Croatian | 1 | NA |
| Ecuadorian | 1 | 1 |
| German | 2 | 2 |
| Indonesian | 1 | 1 |
| Irish | 1 | 1 |
| Italian | 7 | 4 |
| Japanese | 2 | 2 |
| Mexican | 1 | 1 |
| New Zealand | 2 | 2 |
| TBC | 8 | 4 |
| Total | 76 | 58 |

*Table A8: First Language of participants.*

| First Language | Round 1 | Round2 |
|---|---|---|
| Bahasa indonesia | 1 | 1 |
| Croatian | 1 | NA |
| English | 52 | 42 |
| French | 1 | 1 |
| German | 1 | 1 |
| Italian | 7 | 4 |
| Japanese | 2 | 2 |
| Spanish | 3 | 3 |
| TBC | 8 | 4 |
| Total | 76 | 58 |

*Table A9: Occupational sector and participation of participants*

| Sector | Round 1 | Round2 |
|---|---|---|
| - | 1 | NA |
| Conservation NGO | 1 | NA |
| Consultant | 4 | NA |
| Government Authority | 25 | 20 |
| Other | 3 | 13 |
| Private Enterprise | 4 | NA |
| Publicly Funded Research | 5 | NA |
| University | 33 | 25 |
| Total | 76 | 58 |

# Participant responses

## Graphs for each question

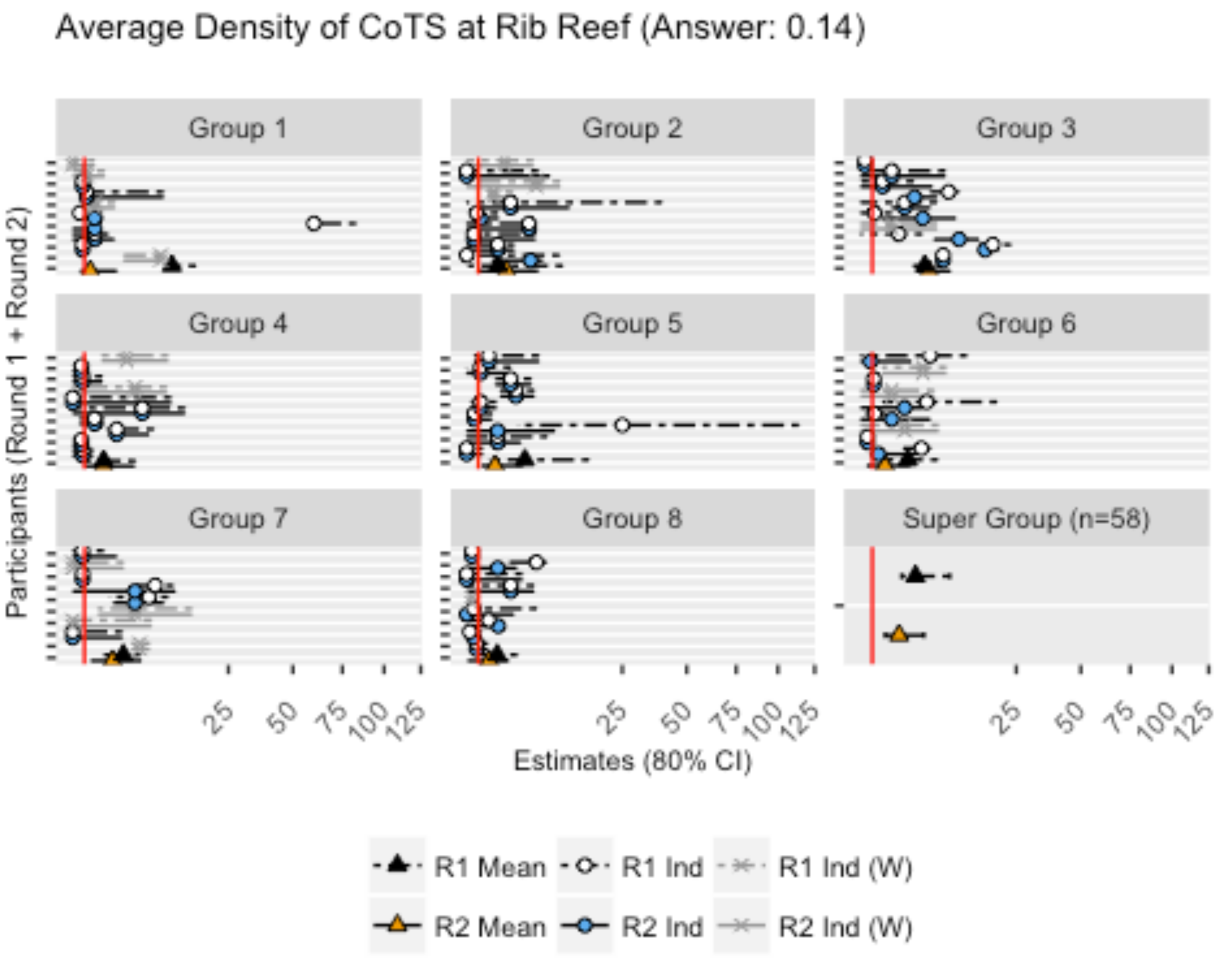
Average Density of CoTS at Rib Reef (Answer: 0.14)
Group 1
Group 2
Group 3
Group 4
Group 5
Group 6
Group 7
Group 8
Super Group (n=58)
Participants (Round 1 + Round 2)
25
50
75
100
125
Estimates (80% CI)
R1 Mean
R1 Ind
R1 Ind (W)
R2 Mean
R2 Ind
R2 Ind (W)

Reefs (out of 24) with 1% Bleaching Hard Coral (Answer: 17)

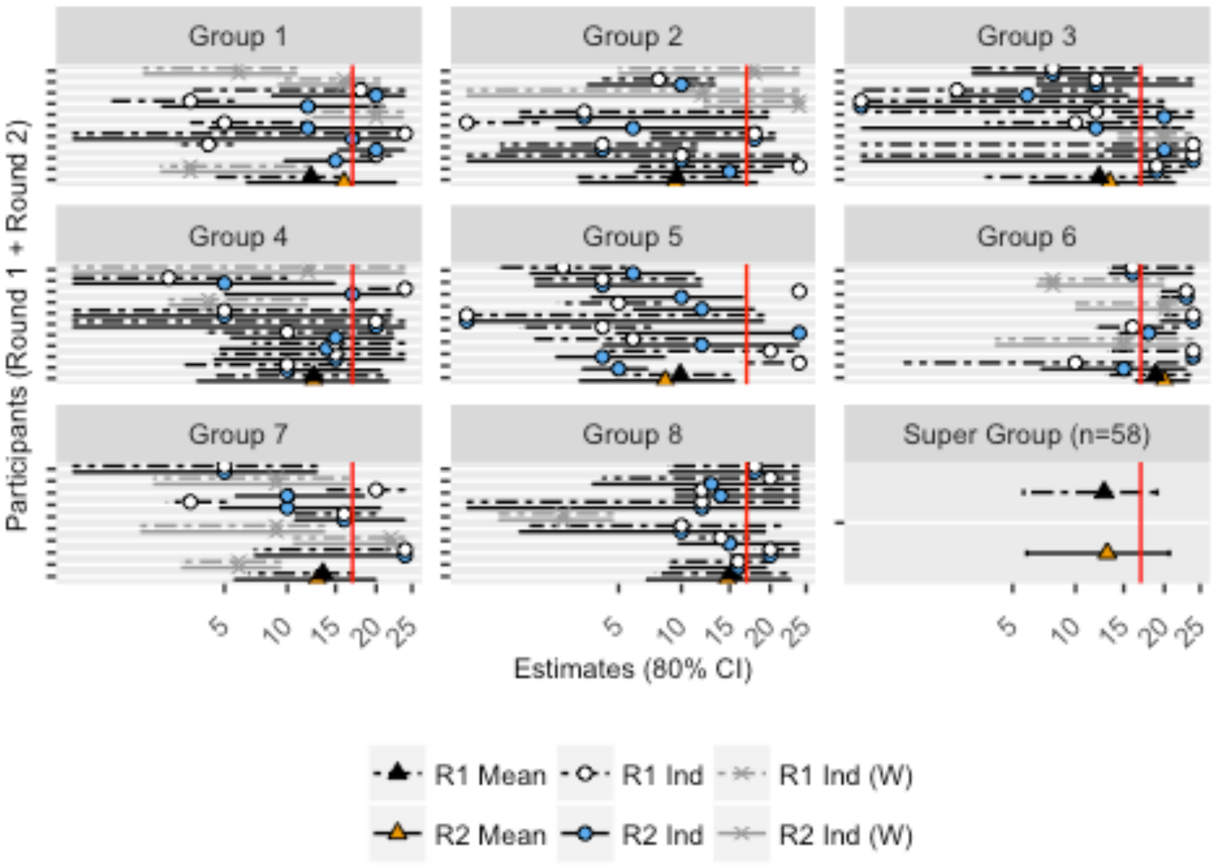

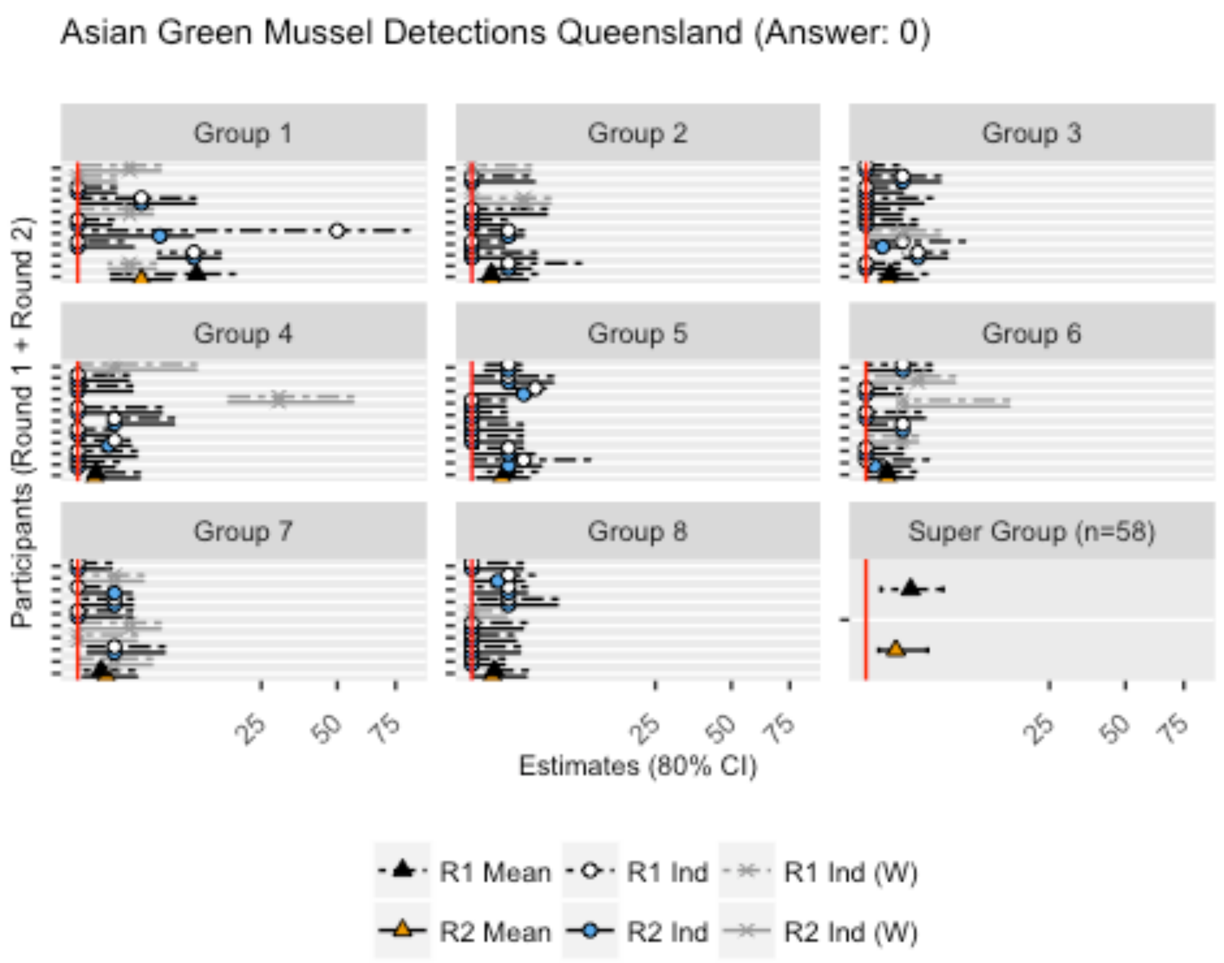

Asian Green Mussel Detections Queensland (Answer: 0)
Group 1
Group 2
Group 3
Group 4
Group 5
Group 6
Group 7
Group 8
Super Group (n=58)
Participants (Round 1 + Round 2)
25
50
75
Estimates (80% CI)
R1 Mean
R1 Ind
R1 Ind (W)
R2 Mean
R2 Ind
R2 Ind (W)

White Syndrome, Reef 21060 (Answer: 0)

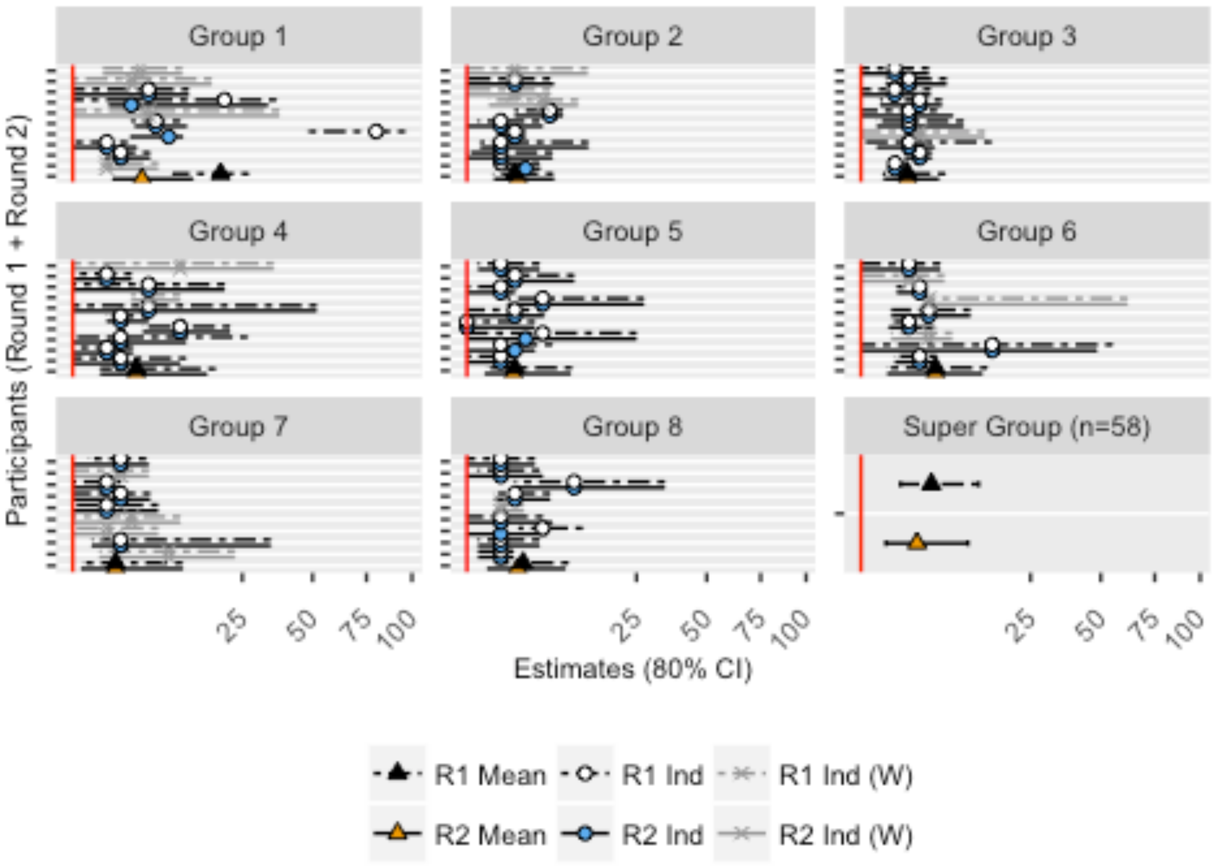

Commercial Catch Coral Trout (Answer: 54.26 tons)

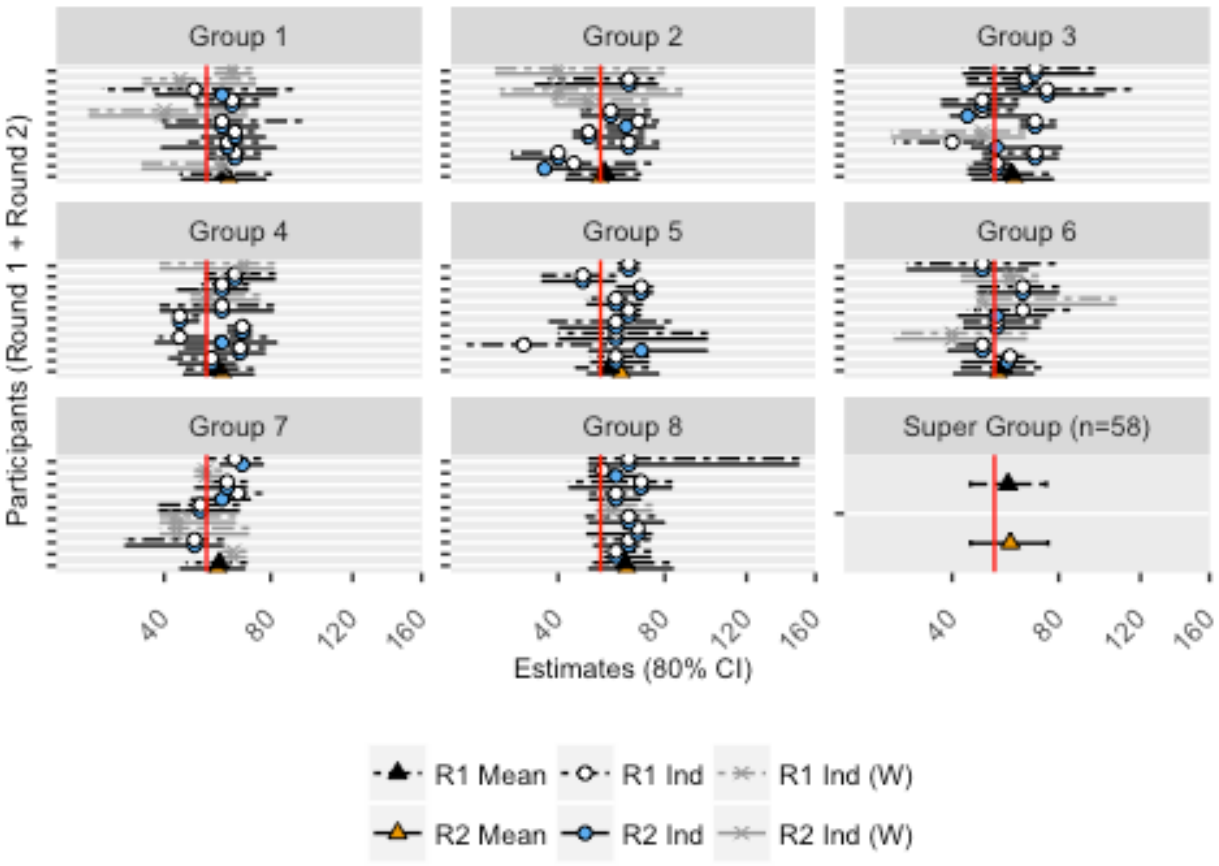

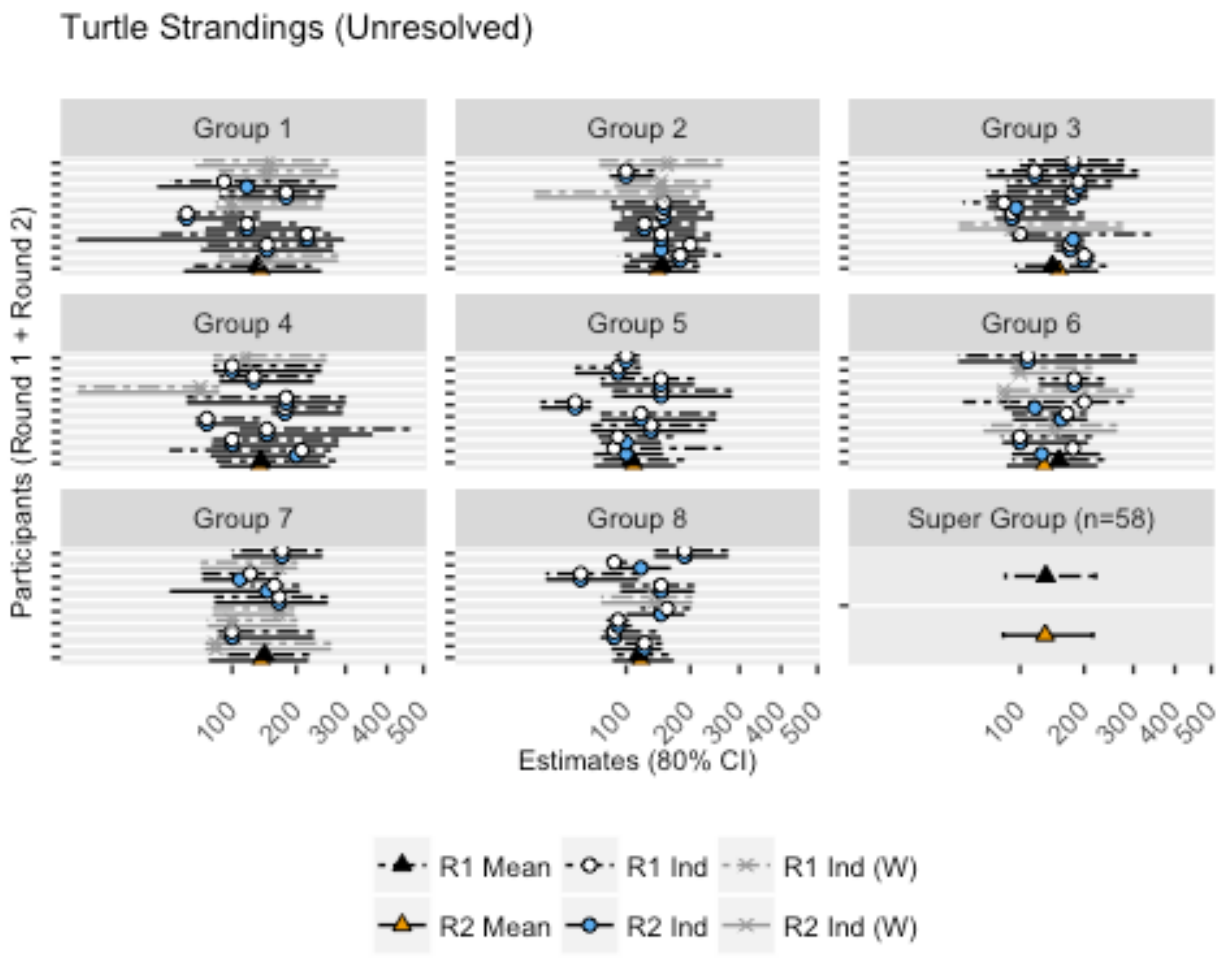
Turtle Strandings (Unresolved)
Group 1
Group 2
Group 3
Group 4
Group 5
Group 6
Group 7
Group 8
Super Group (n=58)
Participants (Round 1 + Round 2)
100
200
300
400
500
Estimates (80% CI)
R1 Mean
R1 Ind
R1 Ind (W)
R2 Mean
R2 Ind
R2 Ind (W)

Shark Control (Answer: 10 Sharks)

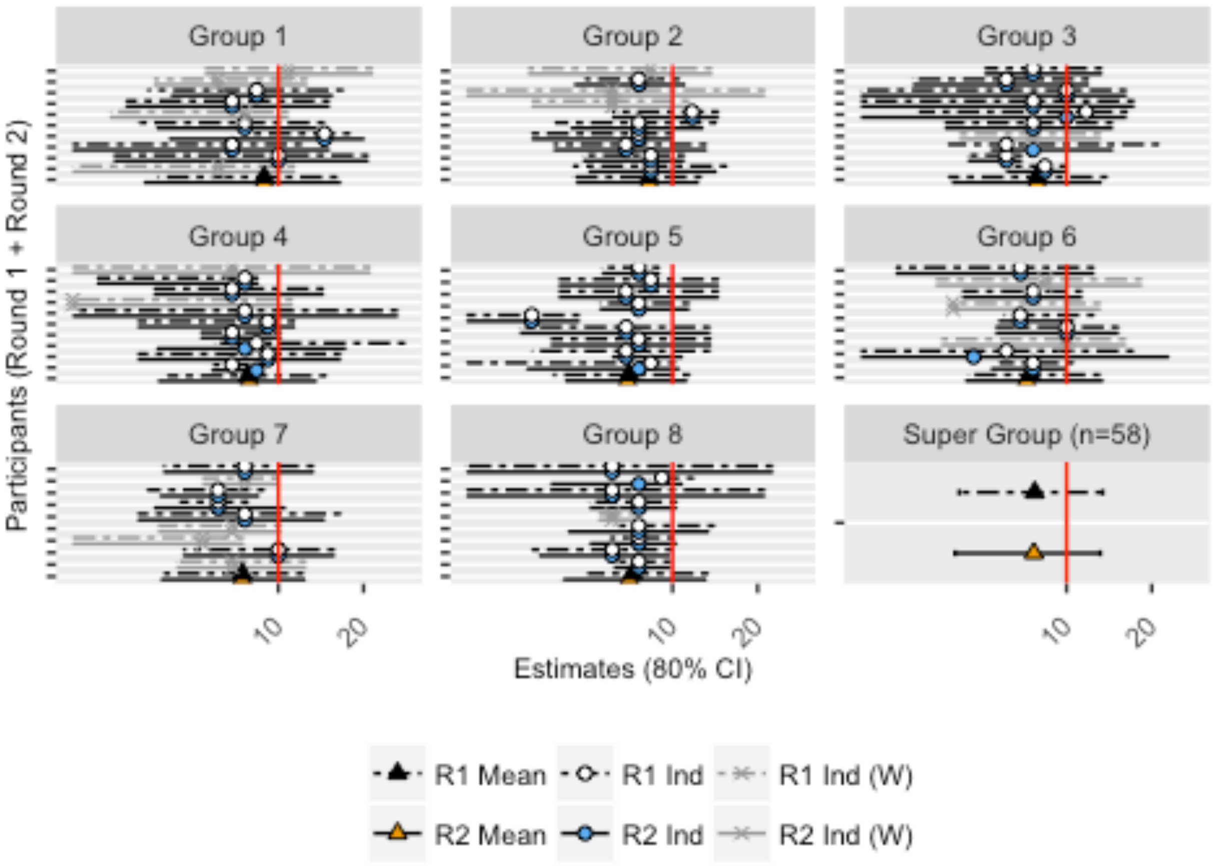

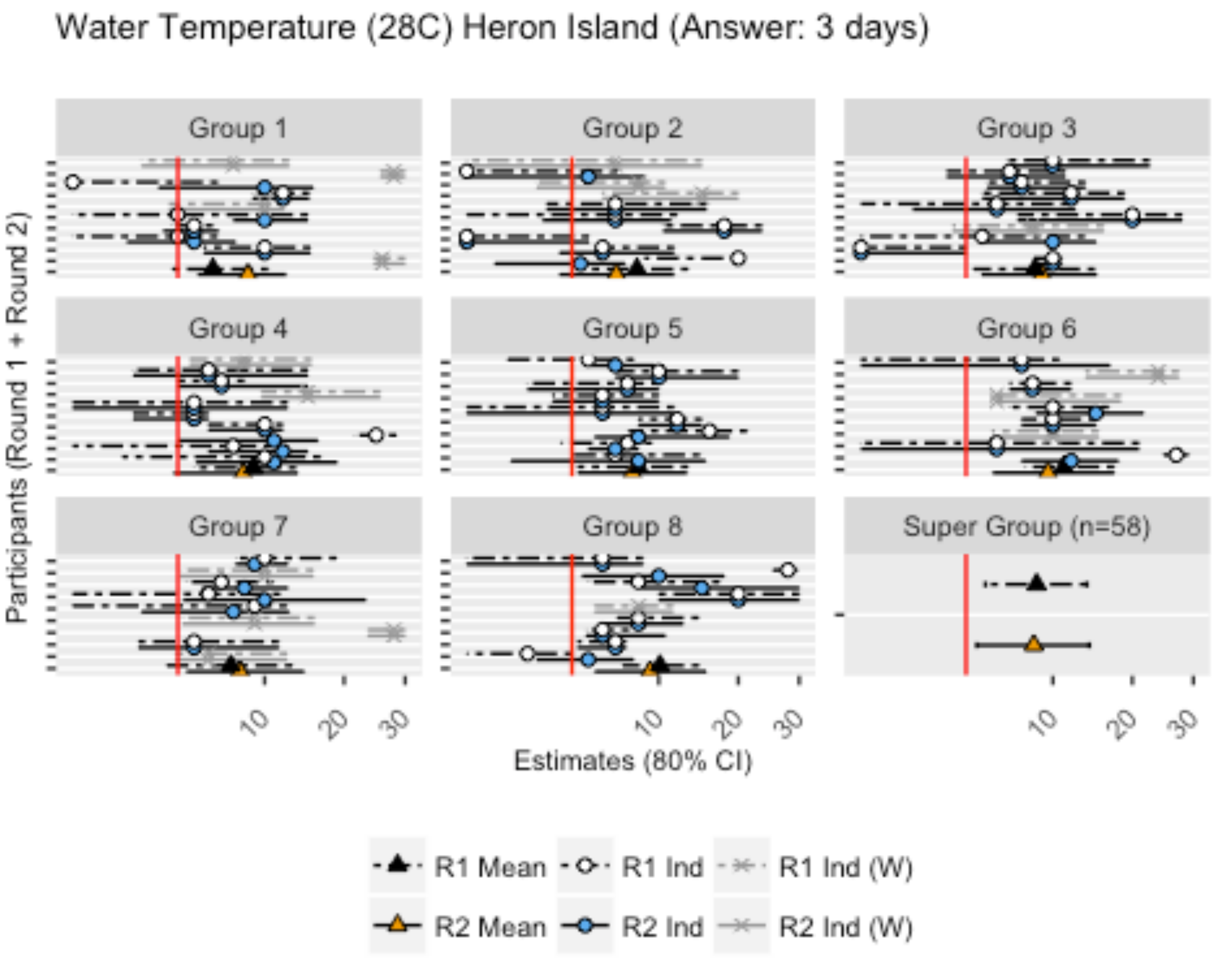

Water Temperature (28C) Heron Island (Answer: 3 days)
Group 1
Group 2
Group 3
Group 4
Group 5
Group 6
Group 7
Group 8
Super Group (n=58)
Participants (Round 1 + Round 2)
10
20
30
Estimates (80% CI)
R1 Mean
R1 Ind
R1 Ind (W)
R2 Mean
R2 Ind
R2 Ind (W)

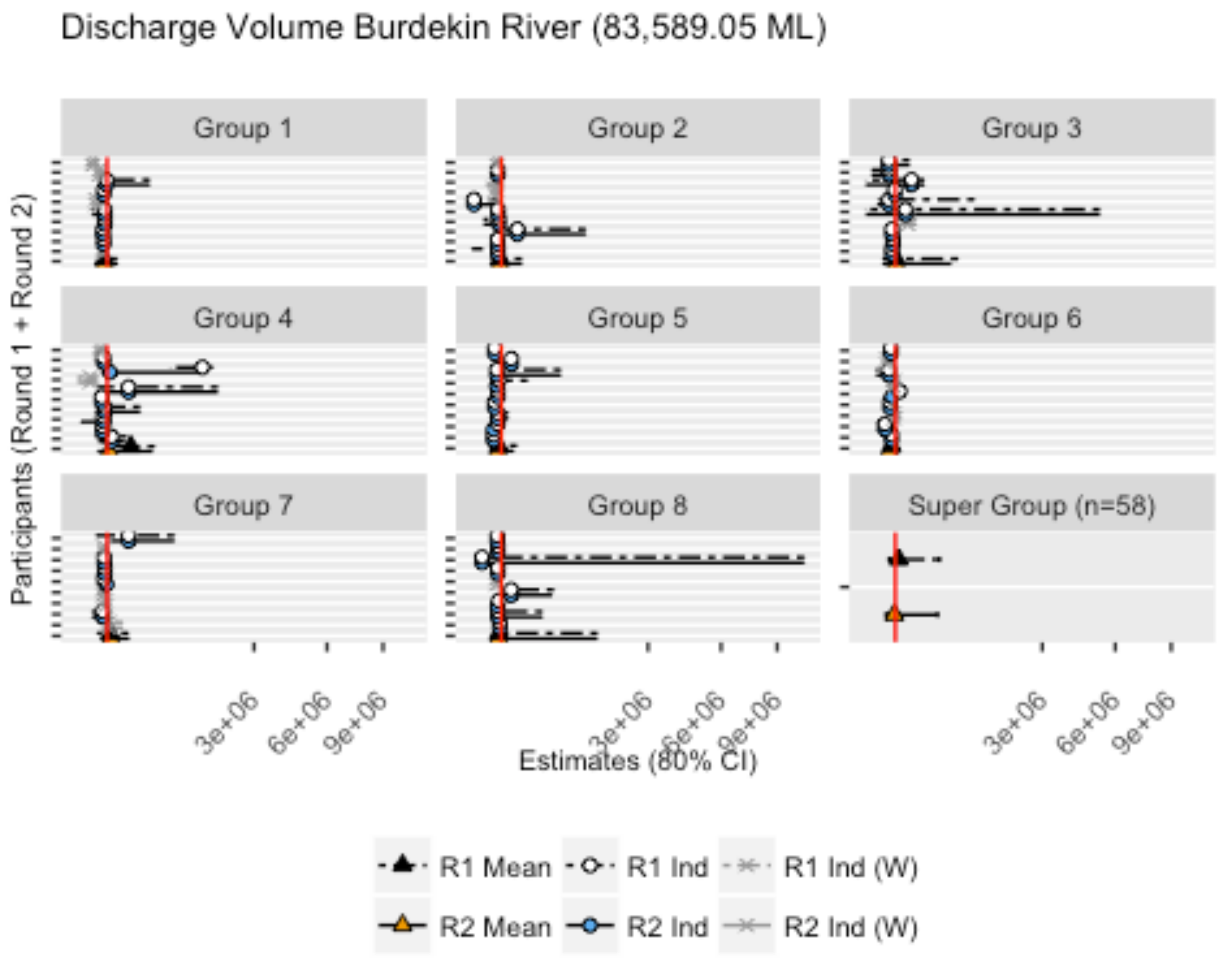
Discharge Volume Burdekin River (83,589.05 ML)
Group 1
Group 2
Group 3
Group 4
Group 5
Group 6
Group 7
Group 8
Super Group (n=58)
Participants (Round 1 + Round 2)
3e+06
6e+06
9e+06
Estimates (80% CI)
R1 Mean
R1 Ind
R1 Ind (W)
R2 Mean
R2 Ind
R2 Ind (W)

Average Chlorophyll Pine Island (Answer: 0.93)

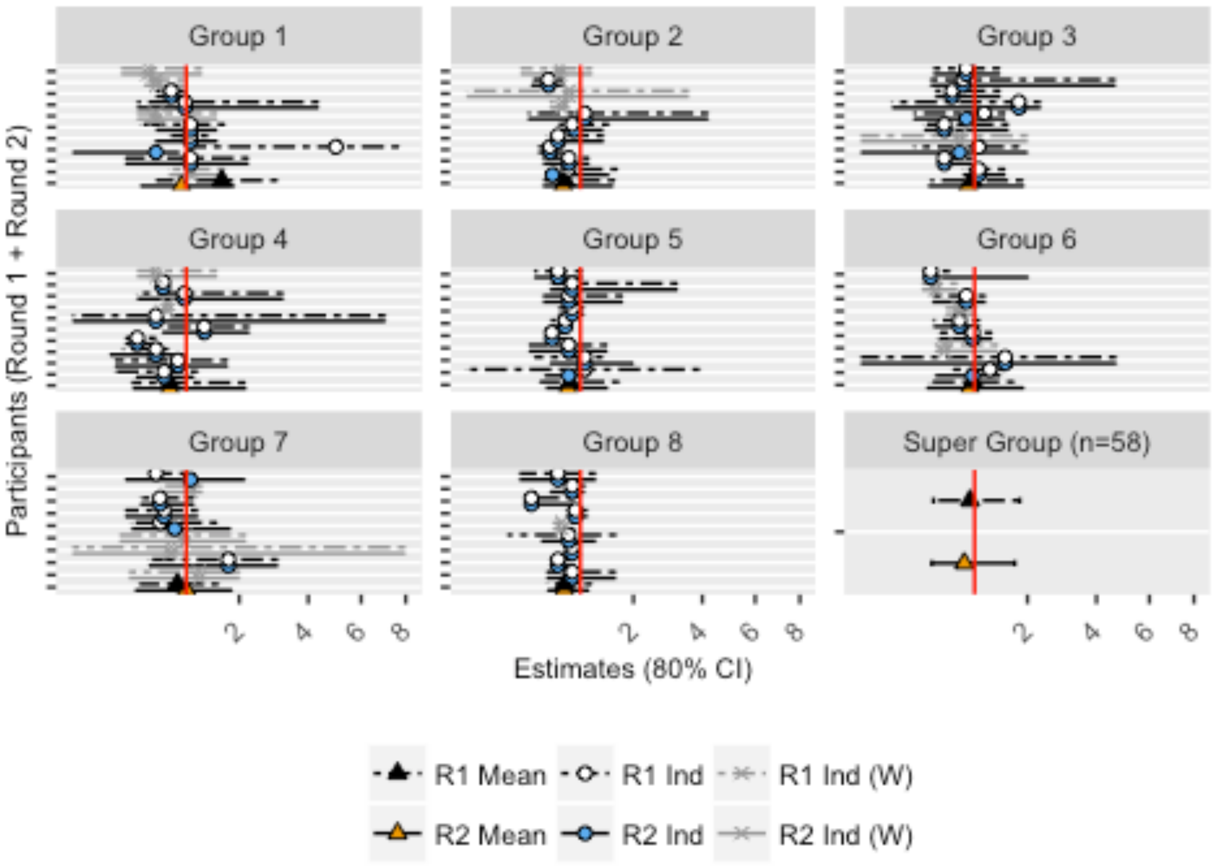

## Wind Speed Davies Reef (Answer: 64.44 km/hr)

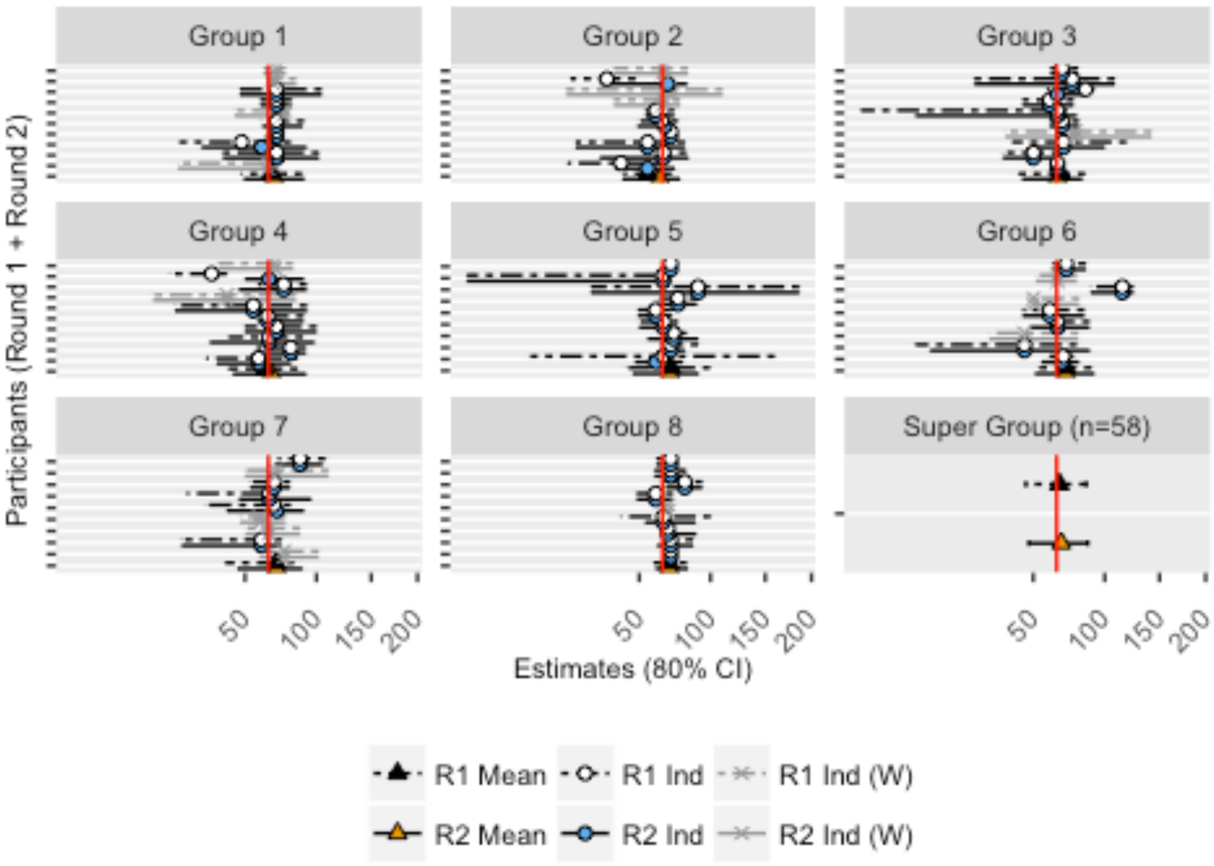

Air Temp. Hamilton Island (Answer: 26.5C)

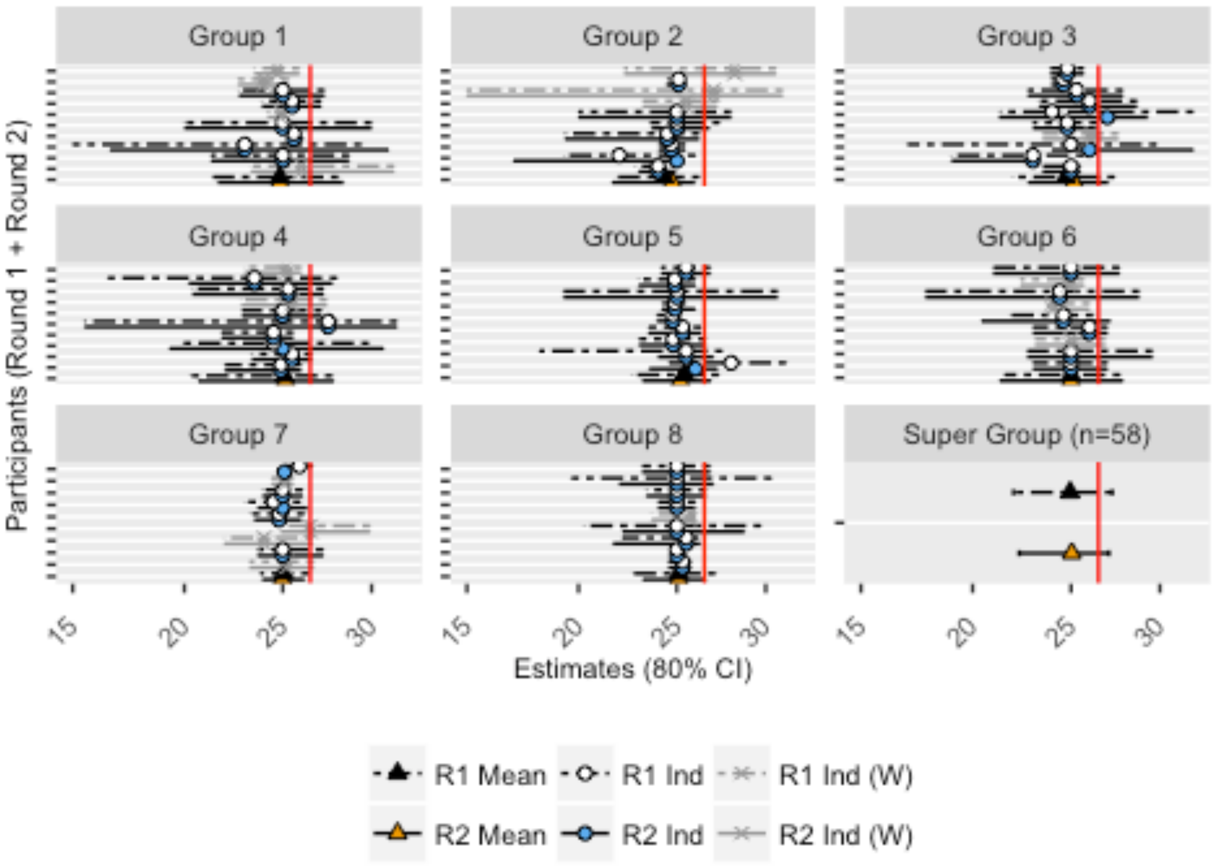

Turbidity (NTU) High West (Answer: 1.38)

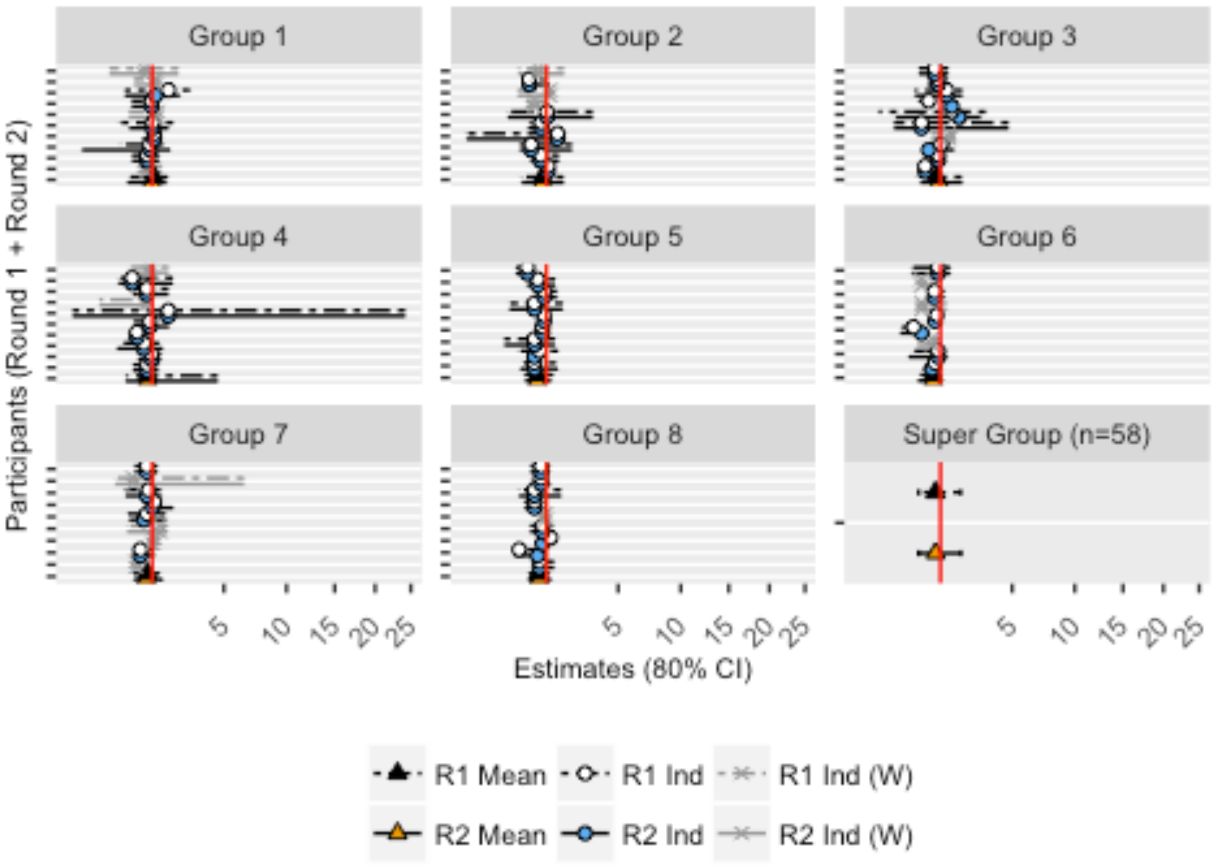

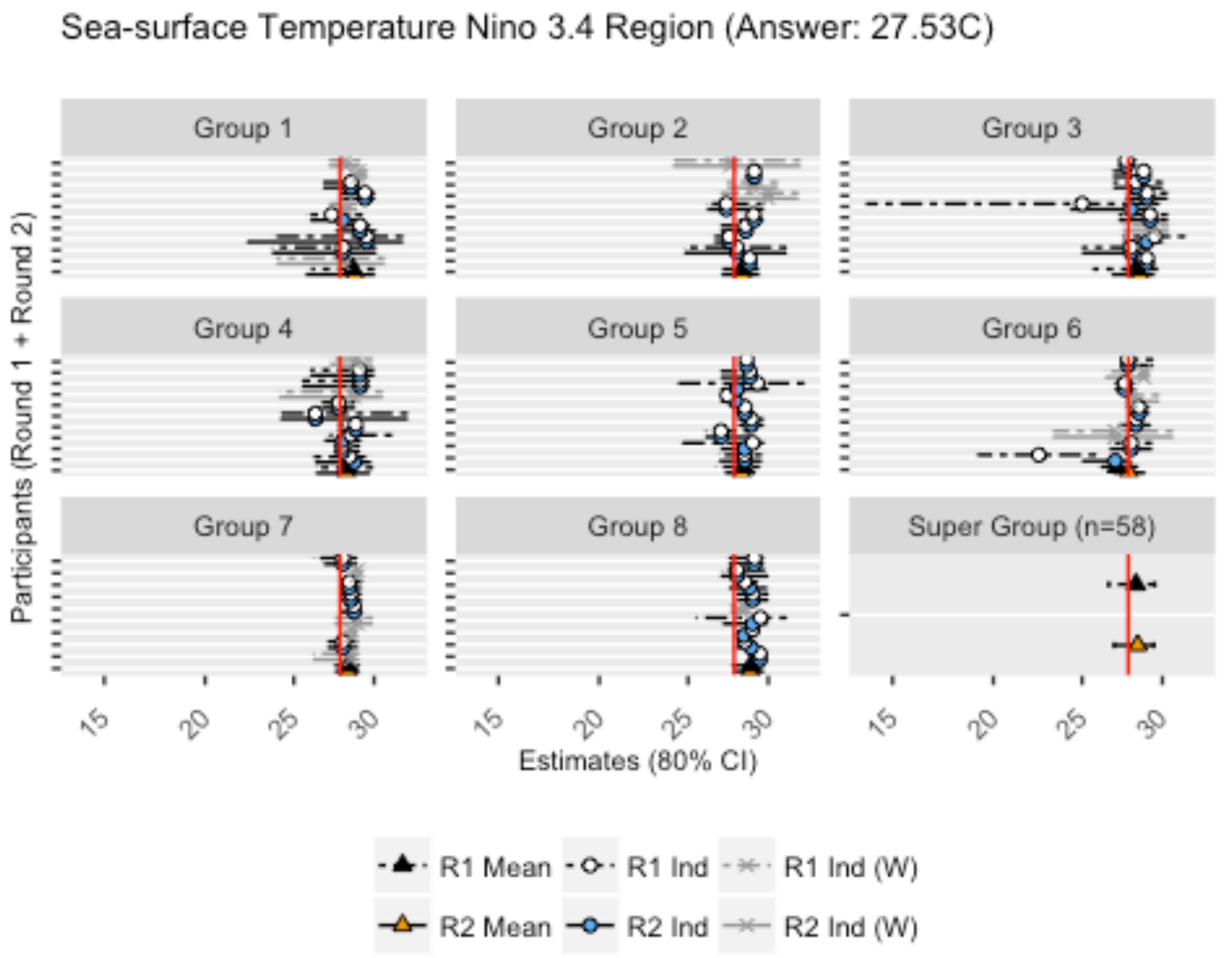

Sea-surface Temperature Nino 3.4 Region (Answer: 27.53C)
Group 1
Group 2
Group 3
Group 4
Group 5
Group 6
Group 7
Group 8
Super Group (n=58)
Participants (Round 1 + Round 2)
15
20
25
30
Estimates (80% CI)
R1 Mean
R1 Ind
R1 Ind (W)
R2 Mean
R2 Ind
R2 Ind (W)

## All 76 participants Round 1 and Round 2

## Round 1: Demographics and performance

### Round 1: Sector vs Accuracy, Calibration, Informativeness

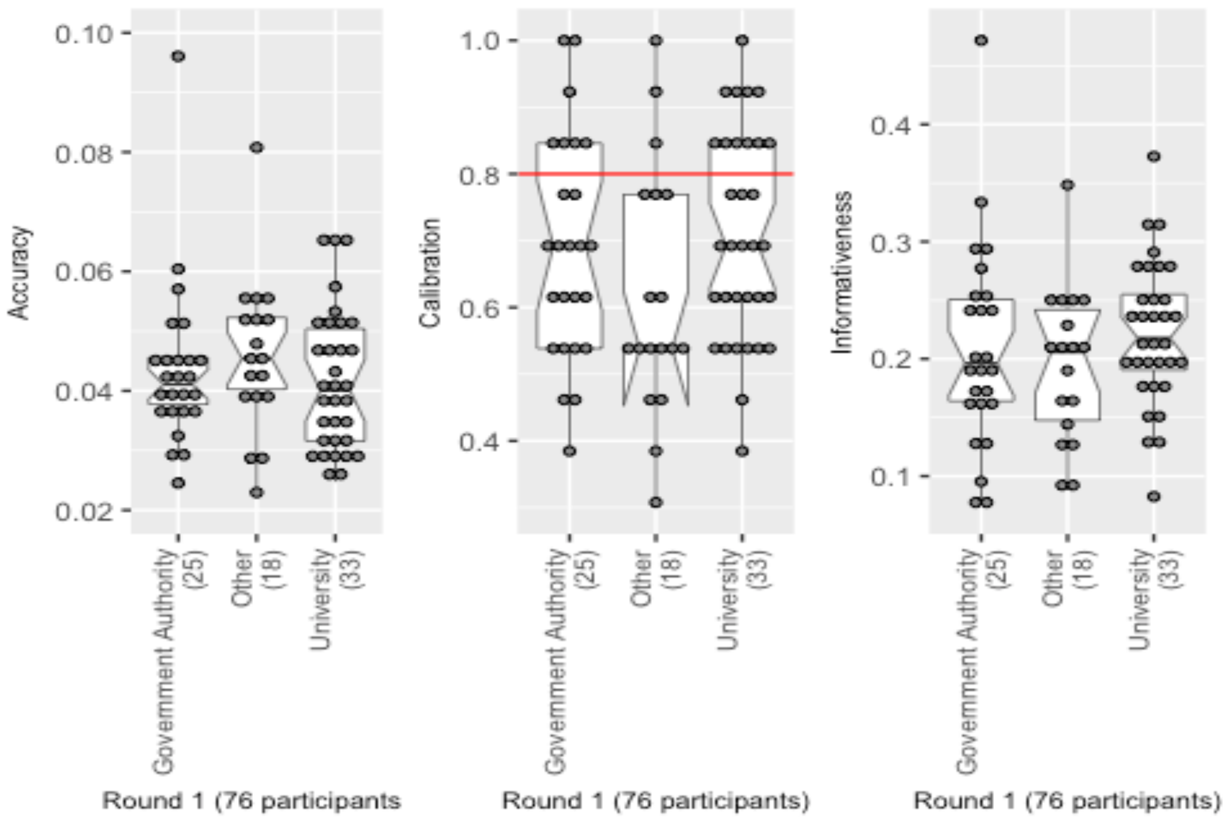


| Test | Median | n | 25th | 75th | LowCI | UpperCI | Stat | Round |
|---|---|---|---|---|---|---|---|---|
| Government Authority | 0.041 | 25 | 0.046 | 0.038 | 0.039 | 0.044 | Accuracy | Round 1 76 |
| Other | 0.045 | 18 | 0.052 | 0.04 | 0.041 | 0.05 | Accuracy | Round 1 76 |
| University | 0.04 | 33 | 0.05 | 0.032 | 0.035 | 0.045 | Accuracy | Round 1 76 |
| Government Authority | 0.692 | 25 | 0.846 | 0.538 | 0.596 | 0.789 | Calibration | Round 1 76 |
| Other | 0.538 | 18 | 0.769 | 0.538 | 0.453 | 0.624 | Calibration | Round 1 76 |
| University | 0.692 | 33 | 0.846 | 0.615 | 0.629 | 0.755 | Calibration | Round 1 76 |
| Government Authority | 0.196 | 25 | 0.251 | 0.164 | 0.169 | 0.223 | Informativeness | Round 1 76 |
| Other | 0.206 | 18 | 0.242 | 0.148 | 0.171 | 0.241 | Informativeness | Round 1 76 |
| University | 0.218 | 33 | 0.255 | 0.191 | 0.2 | 0.235 | Informativeness | Round 1 76 |

## Round 1: Age vs Accuracy, Calibration, Informativeness

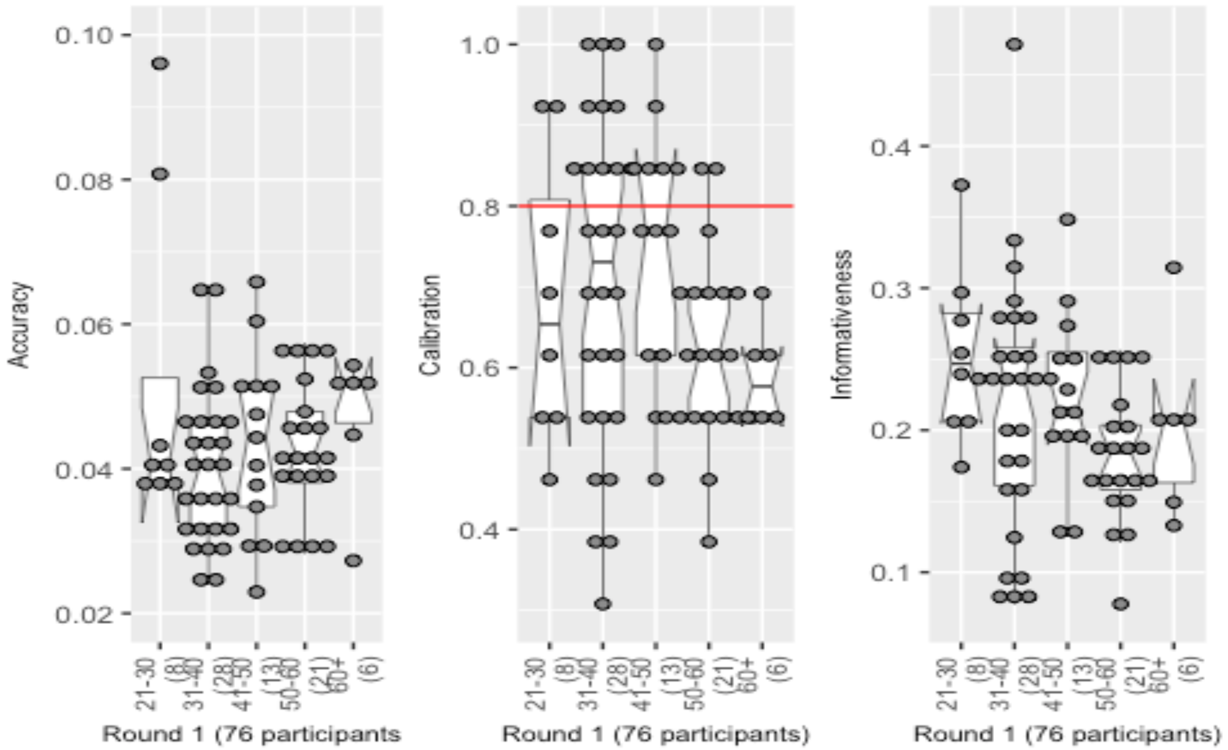


| Test | Median | n | 25th | 75th | LowCI | UpperCI | Stat | Round |
|---|---|---|---|---|---|---|---|---|
| 21-30 | 0.041 | 8 | 0.053 | 0.038 | 0.033 | 0.048 | Accuracy | Round 1 76 |
| 31-40 | 0.04 | 28 | 0.046 | 0.032 | 0.036 | 0.044 | Accuracy | Round 1 76 |
| 41-50 | 0.044 | 13 | 0.051 | 0.035 | 0.037 | 0.052 | Accuracy | Round 1 76 |
| 50-60 | 0.042 | 21 | 0.048 | 0.038 | 0.039 | 0.045 | Accuracy | Round 1 76 |
| 60+ | 0.052 | 6 | 0.052 | 0.046 | 0.048 | 0.055 | Accuracy | Round 1 76 |
| 21-30 | 0.654 | 8 | 0.808 | 0.538 | 0.504 | 0.803 | Calibration | Round 1 76 |
| 31-40 | 0.731 | 28 | 0.846 | 0.538 | 0.639 | 0.822 | Calibration | Round 1 76 |
| 41-50 | 0.769 | 13 | 0.846 | 0.615 | 0.669 | 0.87 | Calibration | Round 1 76 |
| 50-60 | 0.615 | 21 | 0.692 | 0.538 | 0.563 | 0.668 | Calibration | Round 1 76 |
| 60+ | 0.577 | 6 | 0.615 | 0.538 | 0.528 | 0.626 | Calibration | Round 1 76 |
| 21-30 | 0.247 | 8 | 0.282 | 0.207 | 0.205 | 0.289 | Informativeness | Round 1 76 |
| 31-40 | 0.236 | 28 | 0.258 | 0.161 | 0.207 | 0.265 | Informativeness | Round 1 76 |
| 41-50 | 0.215 | 13 | 0.255 | 0.196 | 0.19 | 0.241 | Informativeness | Round 1 76 |
| 50-60 | 0.184 | 21 | 0.204 | 0.158 | 0.169 | 0.2 | Informativeness | Round 1 76 |
| 60+ | 0.206 | 6 | 0.21 | 0.163 | 0.176 | 0.236 | Informativeness | Round 1 76 |

## Round 1: Professional Experience vs Accuracy, Calibration, Informativeness

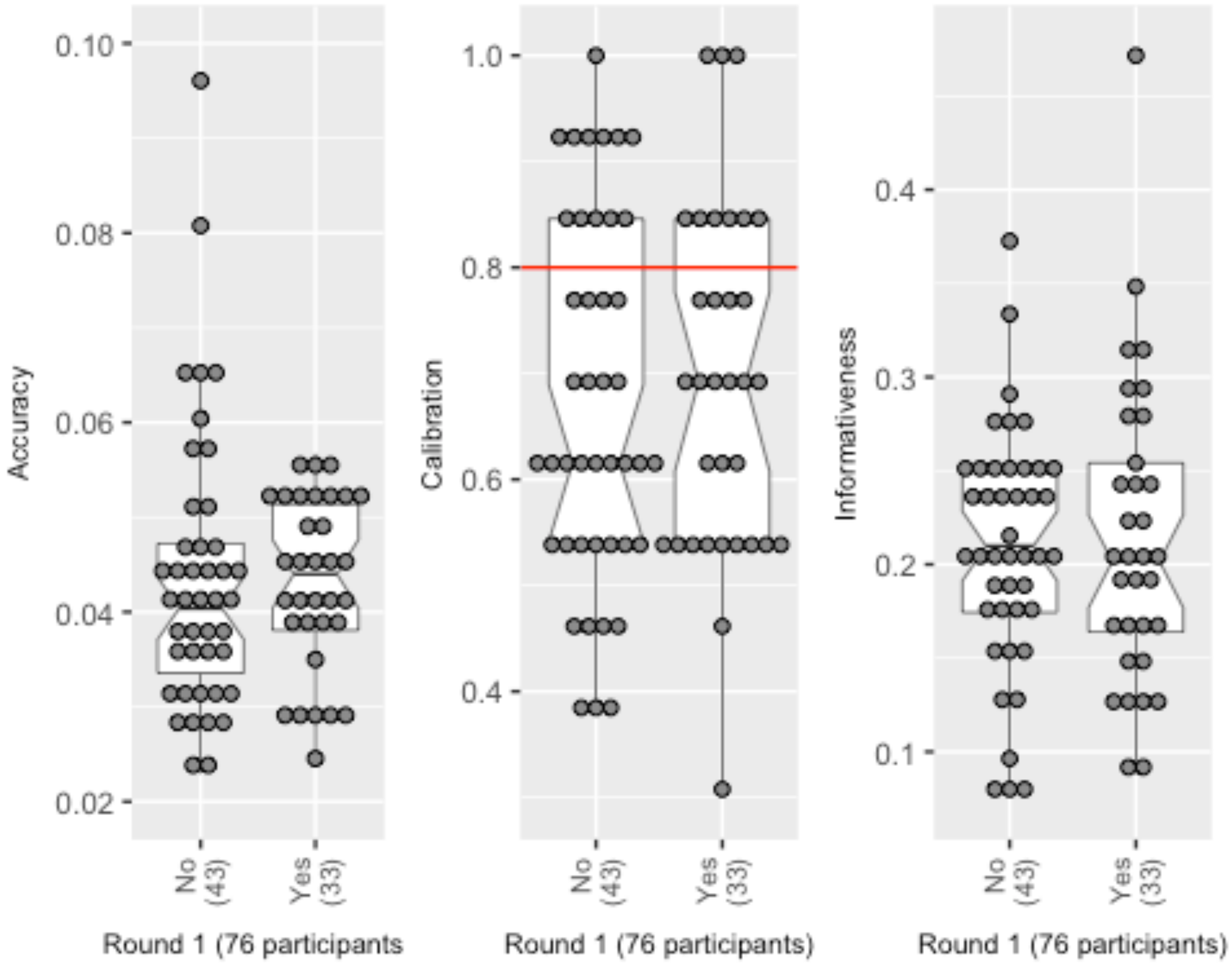


| Test | Median | n | 25th | 75th | LowCI | UpperCI | Stat | Round |
|---|---|---|---|---|---|---|---|---|
| No | 0.04 | 43 | 0.047 | 0.034 | 0.037 | 0.044 | Accuracy | Round 1 76 |
| Yes | 0.044 | 33 | 0.051 | 0.038 | 0.04 | 0.048 | Accuracy | Round 1 76 |
| No | 0.615 | 43 | 0.846 | 0.538 | 0.542 | 0.689 | Calibration | Round 1 76 |
| Yes | 0.692 | 33 | 0.846 | 0.538 | 0.608 | 0.776 | Calibration | Round 1 76 |
| No | 0.21 | 43 | 0.252 | 0.174 | 0.191 | 0.228 | Informativeness | Round 1 76 |
| Yes | 0.202 | 33 | 0.254 | 0.164 | 0.177 | 0.226 | Informativeness | Round 1 76 |

## Round 1: Committee Membership vs Accuracy, Calibration, Informativeness

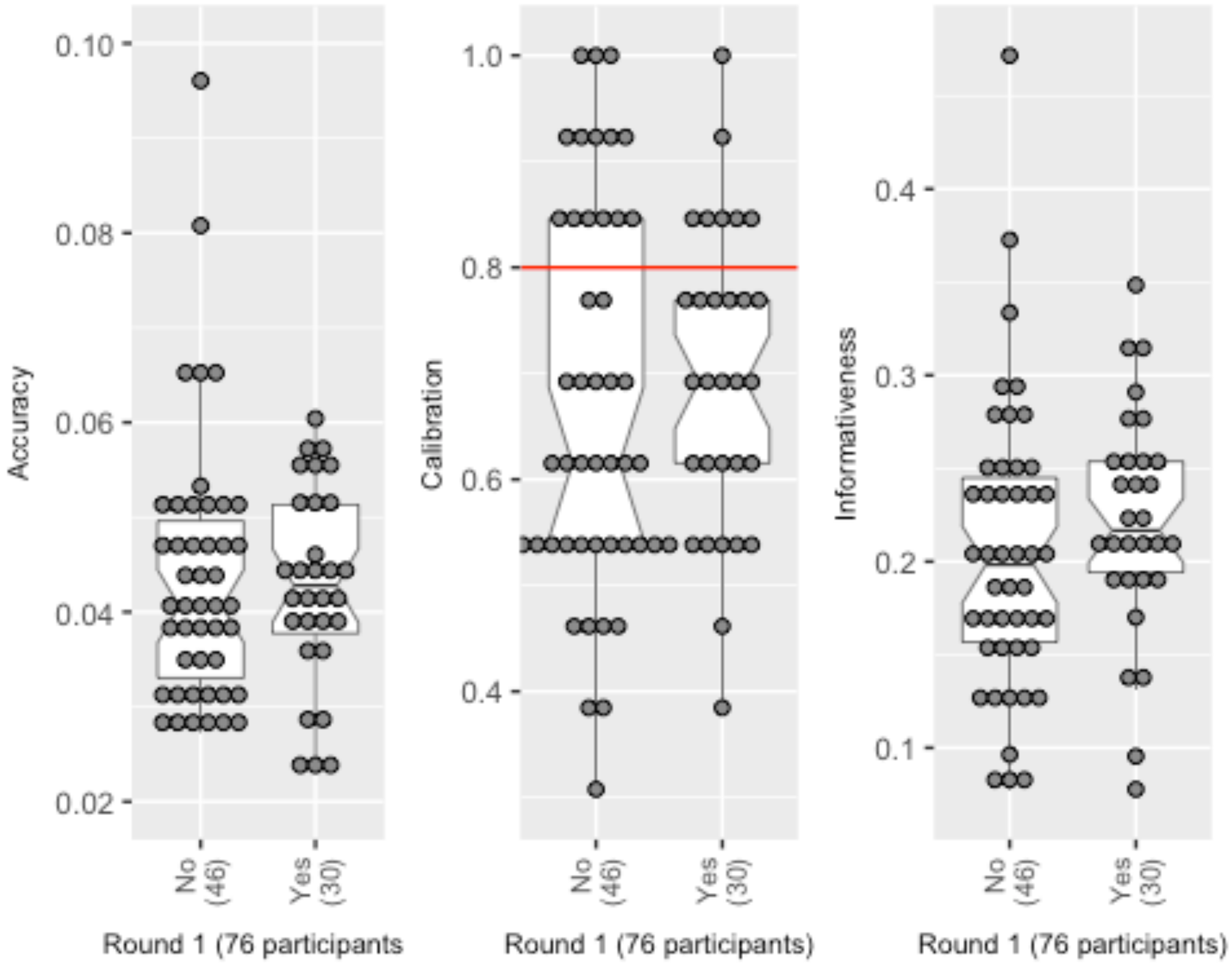


| Test | Median | n | 25th | 75th | LowCI | UpperCI | Stat | Round |
|---|---|---|---|---|---|---|---|---|
| No | 0.041 | 46 | 0.05 | 0.033 | 0.037 | 0.044 | Accuracy | Round 1 76 |
| Yes | 0.043 | 30 | 0.051 | 0.038 | 0.039 | 0.047 | Accuracy | Round 1 76 |
| No | 0.615 | 46 | 0.846 | 0.538 | 0.544 | 0.687 | Calibration | Round 1 76 |
| Yes | 0.692 | 30 | 0.769 | 0.615 | 0.648 | 0.736 | Calibration | Round 1 76 |
| No | 0.198 | 46 | 0.245 | 0.157 | 0.178 | 0.219 | Informativeness | Round 1 76 |
| Yes | 0.217 | 30 | 0.254 | 0.194 | 0.199 | 0.234 | Informativeness | Round 1 76 |

## Round 1: Profession of Expert advice vs Accuracy, Calibration, Informativeness

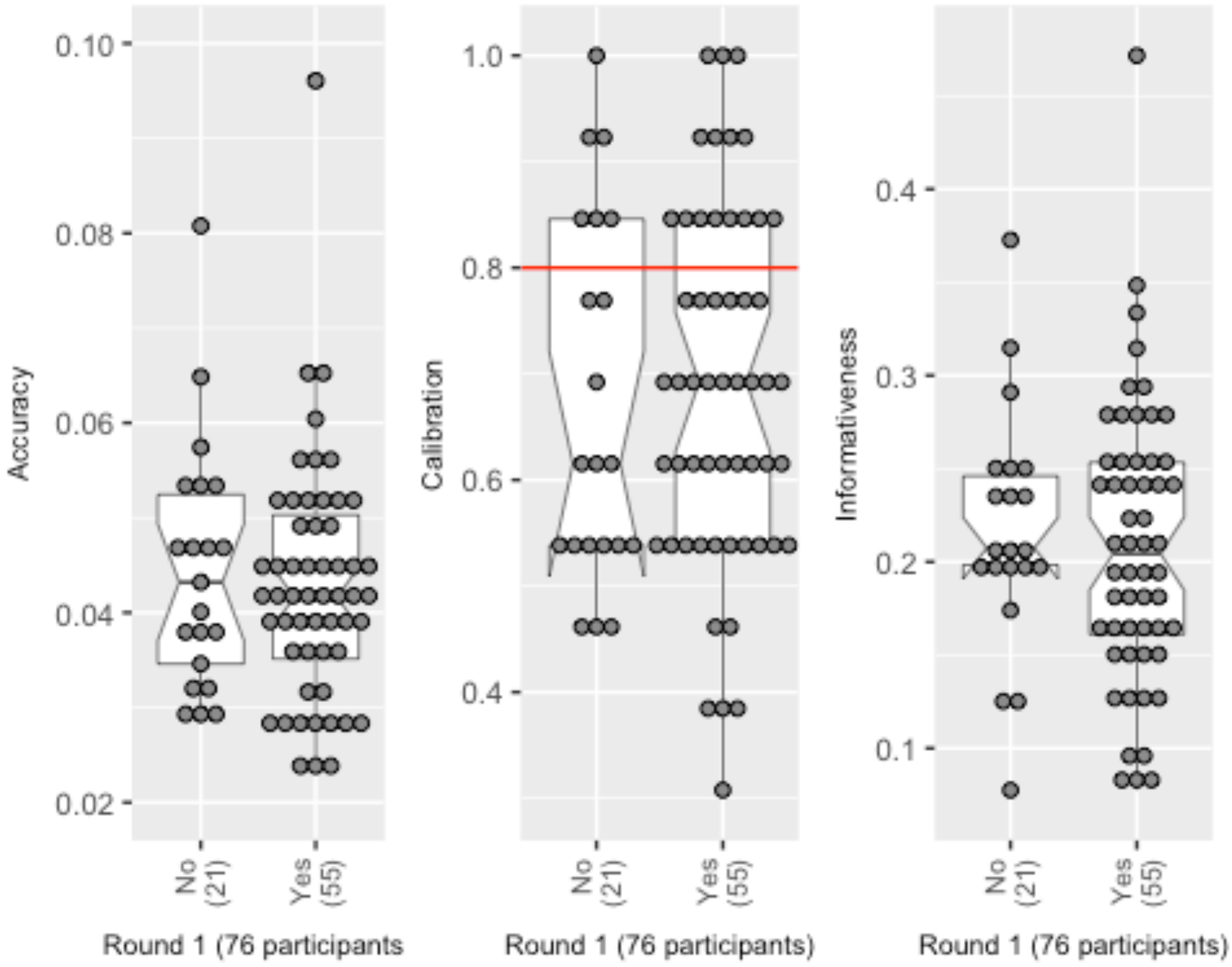


| Test | Median | n | 25th | 75th | LowCI | UpperCI | Stat | Round |
|---|---|---|---|---|---|---|---|---|
| No | 0.043 | 21 | 0.052 | 0.035 | 0.037 | 0.049 | Accuracy | Round 1 76 |
| Yes | 0.042 | 55 | 0.05 | 0.035 | 0.038 | 0.045 | Accuracy | Round 1 76 |
| No | 0.615 | 21 | 0.846 | 0.538 | 0.51 | 0.721 | Calibration | Round 1 76 |
| Yes | 0.692 | 55 | 0.846 | 0.538 | 0.627 | 0.757 | Calibration | Round 1 76 |
| No | 0.207 | 21 | 0.246 | 0.198 | 0.191 | 0.224 | Informativeness | Round 1 76 |
| Yes | 0.204 | 55 | 0.253 | 0.161 | 0.185 | 0.224 | Informativeness | Round 1 76 |

## Round 1: Salutation vs Accuracy, Calibration, Informativeness

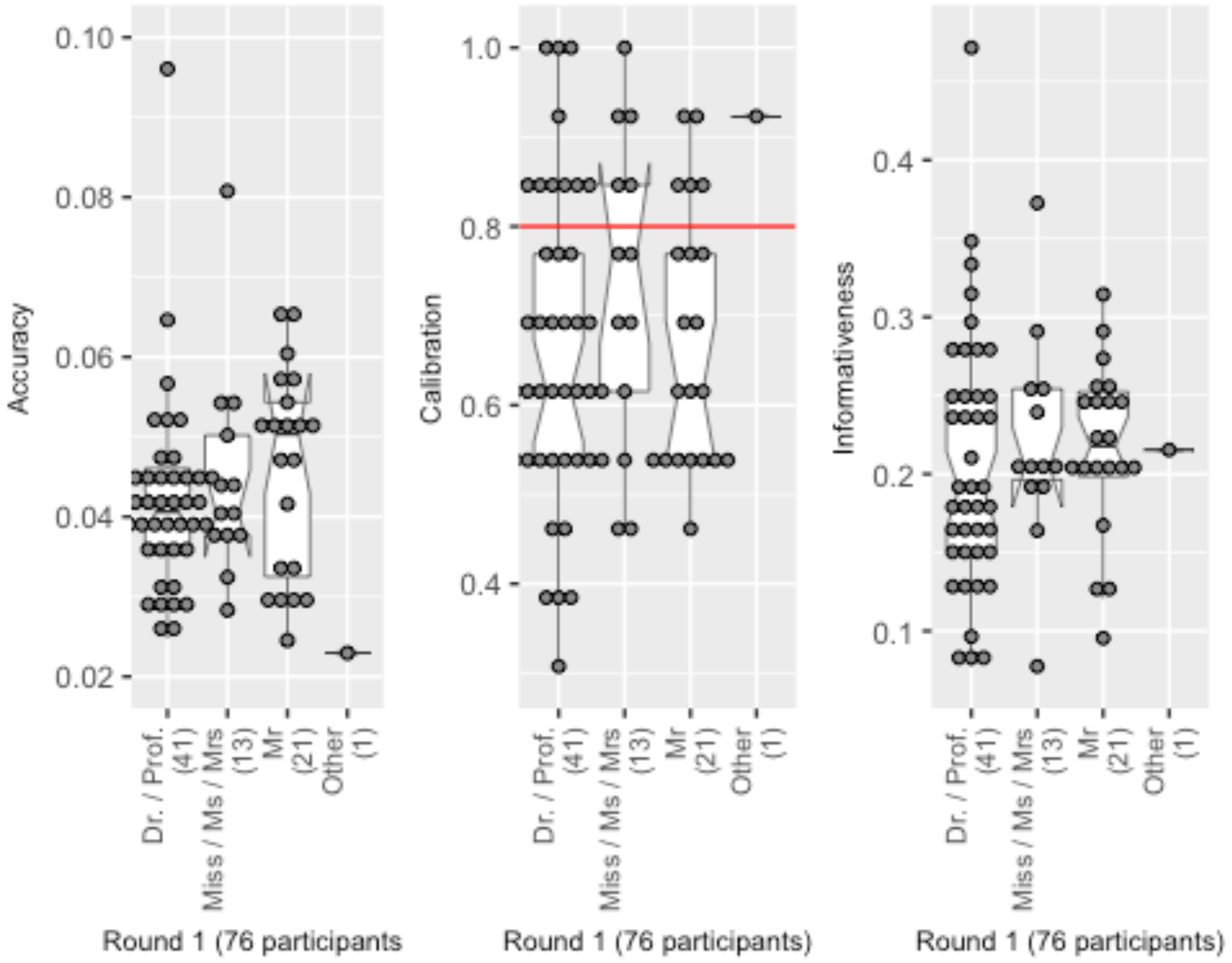


| Test | Median | n | 25th | 75th | LowCI | UpperCI | Stat | Round |
|---|---|---|---|---|---|---|---|---|
| Dr. / Prof. | 0.041 | 41 | 0.046 | 0.035 | 0.038 | 0.043 | Accuracy | Round 1 76 |
| Miss / Ms / Mrs | 0.04 | 13 | 0.05 | 0.038 | 0.035 | 0.046 | Accuracy | Round 1 76 |
| Mr | 0.05 | 21 | 0.054 | 0.033 | 0.043 | 0.058 | Accuracy | Round 1 76 |
| Other | 0.023 | 1 | 0.023 | 0.023 | 0.023 | 0.023 | Accuracy | Round 1 76 |
| Dr. / Prof. | 0.615 | 41 | 0.769 | 0.538 | 0.559 | 0.672 | Calibration | Round 1 76 |
| Miss / Ms / Mrs | 0.769 | 13 | 0.846 | 0.615 | 0.669 | 0.87 | Calibration | Round 1 76 |
| Mr | 0.615 | 21 | 0.769 | 0.538 | 0.536 | 0.694 | Calibration | Round 1 76 |
| Other | 0.923 | 1 | 0.923 | 0.923 | 0.923 | 0.923 | Calibration | Round 1 76 |
| Dr. / Prof. | 0.19 | 41 | 0.251 | 0.152 | 0.166 | 0.214 | Informativeness | Round 1 76 |
| Miss / Ms / Mrs | 0.204 | 13 | 0.254 | 0.196 | 0.179 | 0.23 | Informativeness | Round 1 76 |
| Mr | 0.218 | 21 | 0.252 | 0.198 | 0.199 | 0.236 | Informativeness | Round 1 76 |
| Other | 0.215 | 1 | 0.215 | 0.215 | 0.215 | 0.215 | Informativeness | Round 1 76 |

## Round 1: Sourced vs Accuracy, Calibration, Informativeness

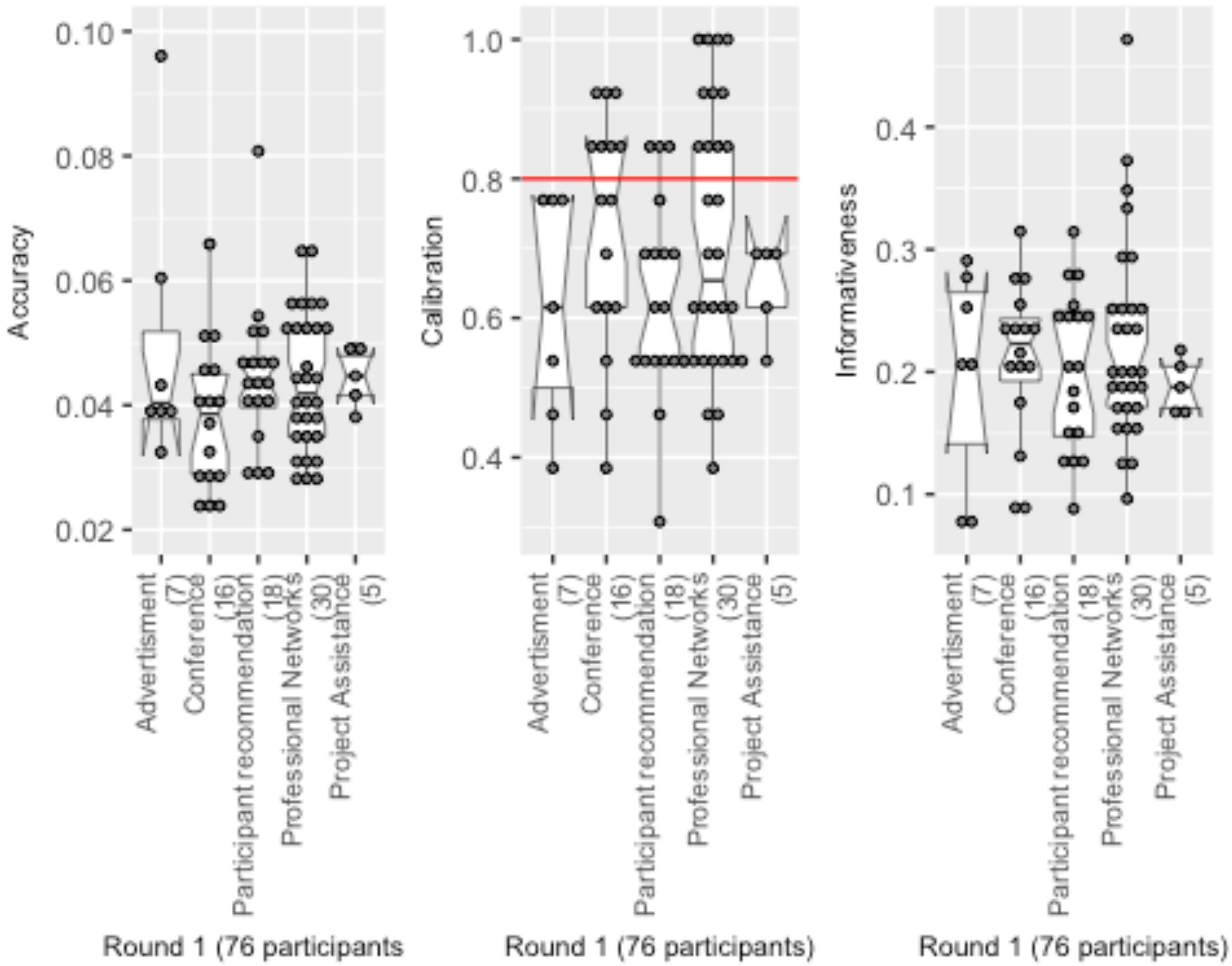


| Test | Median | n | 25th | 75th | LowCI | UpperCI | Stat | Round |
|---|---|---|---|---|---|---|---|---|
| Advertisment | 0.04 | 7 | 0.052 | 0.038 | 0.032 | 0.049 | Accuracy | Round 1 76 |
| Conference | 0.039 | 16 | 0.045 | 0.029 | 0.032 | 0.045 | Accuracy | Round 1 76 |
| Participant recommendation | 0.044 | 18 | 0.047 | 0.04 | 0.041 | 0.047 | Accuracy | Round 1 76 |
| Professional Networks | 0.042 | 30 | 0.052 | 0.035 | 0.037 | 0.047 | Accuracy | Round 1 76 |
| Project Assistance | 0.045 | 5 | 0.048 | 0.042 | 0.04 | 0.049 | Accuracy | Round 1 76 |
| Advertisment | 0.615 | 7 | 0.769 | 0.5 | 0.456 | 0.775 | Calibration | Round 1 76 |

| | | | | | | | | |
|---|---|---|---|---|---|---|---|---|
| Conference | 0.769 | 16 | 0.846 | 0.615 | 0.679 | 0.86 | Calibration | Round 1 76 |
| Participant recommendation | 0.615 | 18 | 0.692 | 0.538 | 0.558 | 0.672 | Calibration | Round 1 76 |
| Professional Networks | 0.654 | 30 | 0.846 | 0.538 | 0.566 | 0.742 | Calibration | Round 1 76 |
| Project Assistance | 0.692 | 5 | 0.692 | 0.615 | 0.638 | 0.746 | Calibration | Round 1 76 |
| Advertisment | 0.208 | 7 | 0.265 | 0.141 | 0.134 | 0.281 | Informativeness | Round 1 76 |
| Conference | 0.223 | 16 | 0.244 | 0.192 | 0.203 | 0.243 | Informativeness | Round 1 76 |
| Participant recommendation | 0.204 | 18 | 0.25 | 0.147 | 0.166 | 0.242 | Informativeness | Round 1 76 |
| Professional Networks | 0.2 | 30 | 0.254 | 0.171 | 0.176 | 0.224 | Informativeness | Round 1 76 |
| Project Assistance | 0.187 | 5 | 0.204 | 0.17 | 0.164 | 0.211 | Informativeness | Round 1 76 |

## Round 1: First language vs Accuracy, Calibration, Informativeness

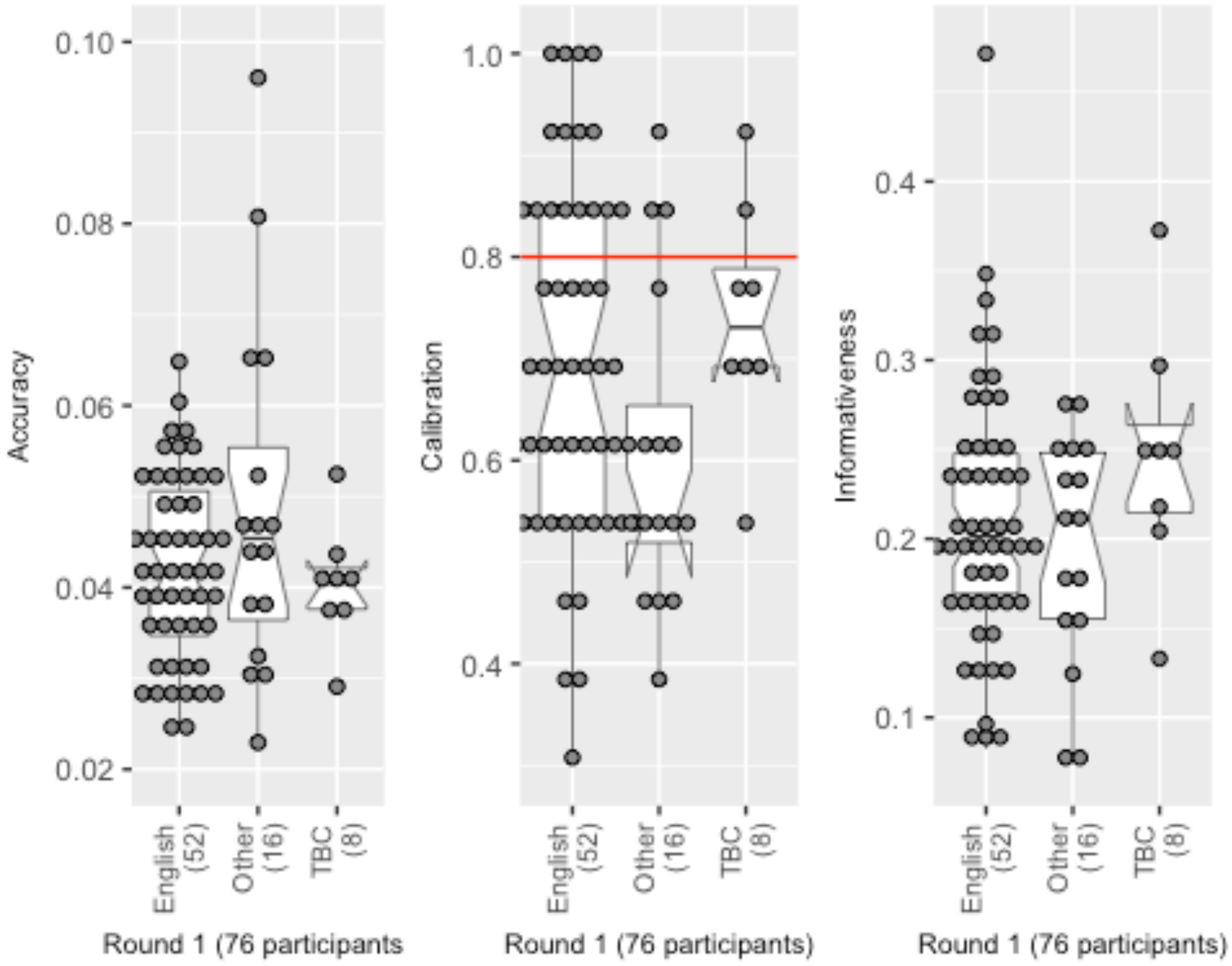


| Test | Median | n | 25th | 75th | LowCI | UpperCI | Stat | Round |
|---|---|---|---|---|---|---|---|---|
| English | 0.042 | 52 | 0.051 | 0.035 | 0.038 | 0.045 | Accuracy | Round 1 76 |
| Other | 0.045 | 16 | 0.055 | 0.036 | 0.038 | 0.053 | Accuracy | Round 1 76 |
| TBC | 0.04 | 8 | 0.042 | 0.038 | 0.038 | 0.043 | Accuracy | Round 1 76 |
| English | 0.692 | 52 | 0.846 | 0.538 | 0.625 | 0.759 | Calibration | Round 1 76 |
| Other | 0.538 | 16 | 0.654 | 0.519 | 0.486 | 0.591 | Calibration | Round 1 76 |
| TBC | 0.731 | 8 | 0.788 | 0.692 | 0.677 | 0.784 | Calibration | Round 1 76 |
| English | 0.201 | 52 | 0.248 | 0.169 | 0.184 | 0.218 | Informativeness | Round 1 76 |
| Other | 0.212 | 16 | 0.248 | 0.155 | 0.175 | 0.248 | Informativeness | Round 1 76 |
| TBC | 0.248 | 8 | 0.264 | 0.214 | 0.221 | 0.276 | Informativeness | Round 1 76 |

## Round 1: Nationality vs Accuracy, Calibration, Informativeness

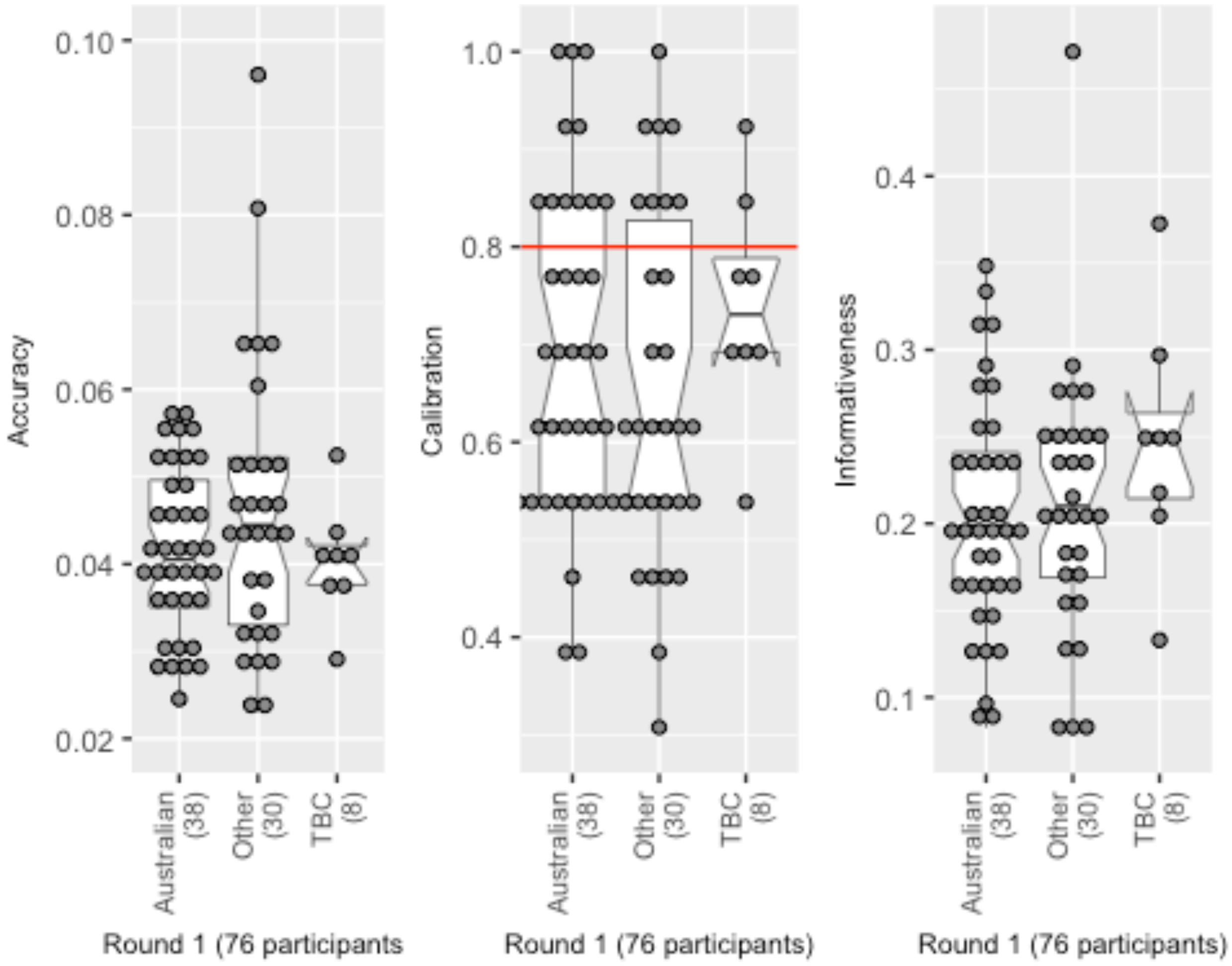


| Test | Median | n | 25th | 75th | LowCI | UpperCI | Stat | Round |
|---|---|---|---|---|---|---|---|---|
| Australian | 0.041 | 38 | 0.05 | 0.035 | 0.037 | 0.044 | Accuracy | Round 1 76 |
| Other | 0.044 | 30 | 0.052 | 0.033 | 0.039 | 0.05 | Accuracy | Round 1 76 |
| TBC | 0.04 | 8 | 0.042 | 0.038 | 0.038 | 0.043 | Accuracy | Round 1 76 |
| Australian | 0.692 | 38 | 0.846 | 0.538 | 0.614 | 0.771 | Calibration | Round 1 76 |
| Other | 0.615 | 30 | 0.827 | 0.538 | 0.533 | 0.698 | Calibration | Round 1 76 |
| TBC | 0.731 | 8 | 0.788 | 0.692 | 0.677 | 0.784 | Calibration | Round 1 76 |
| Australian | 0.199 | 38 | 0.241 | 0.165 | 0.179 | 0.218 | Informativeness | Round 1 76 |
| Other | 0.21 | 30 | 0.251 | 0.169 | 0.187 | 0.234 | Informativeness | Round 1 76 |
| TBC | 0.248 | 8 | 0.264 | 0.214 | 0.221 | 0.276 | Informativeness | Round 1 76 |

## Round 1: Peer recommendation vs Accuracy, Calibration, Informativeness

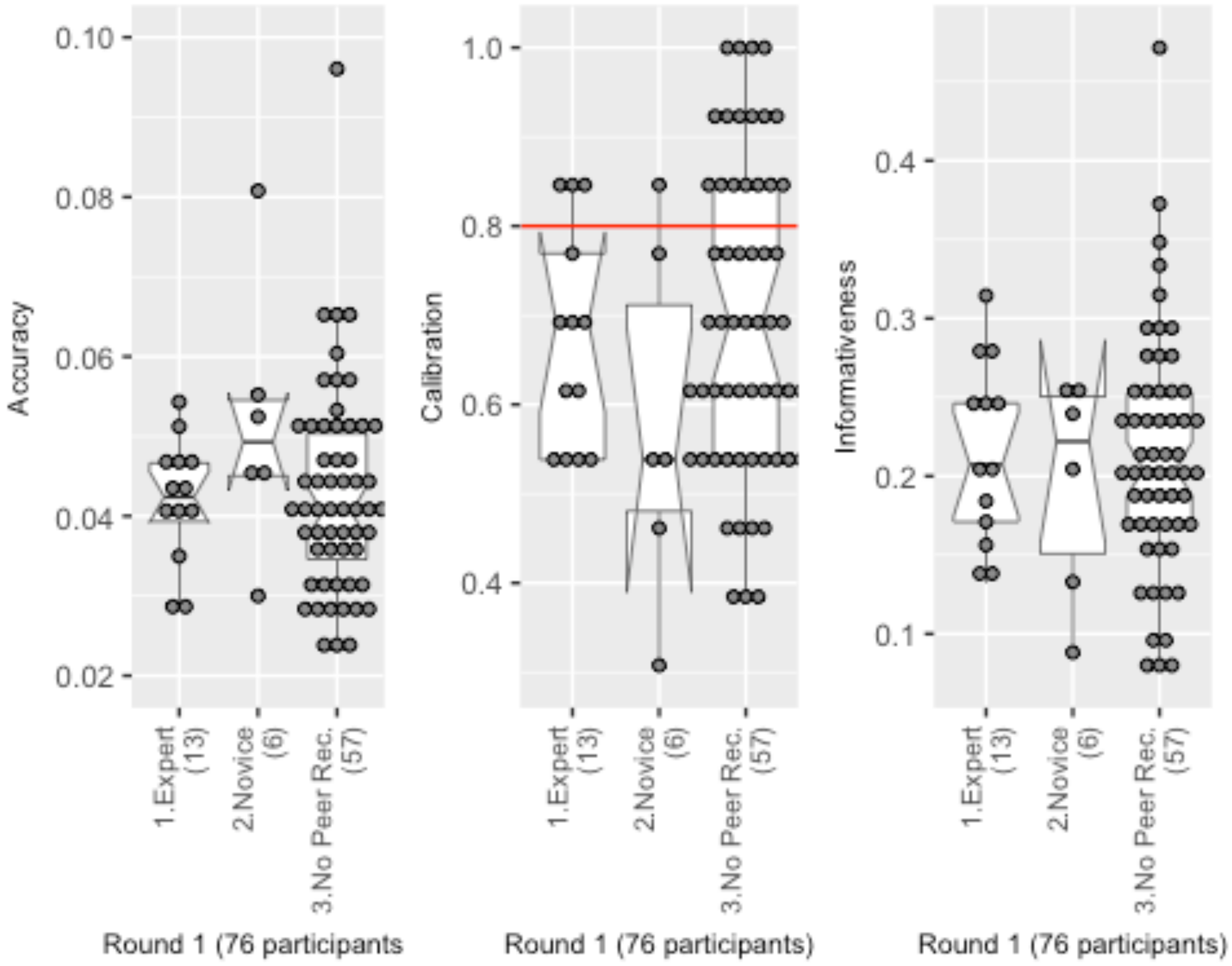


| Test | Median | n | 25th | 75th | LowCI | UpperCI | Stat | Round |
|---|---|---|---|---|---|---|---|---|
| 1.Expert | 0.042 | 13 | 0.047 | 0.039 | 0.039 | 0.046 | Accuracy | Round 1 76 |
| 2.Novice | 0.049 | 6 | 0.055 | 0.045 | 0.043 | 0.055 | Accuracy | Round 1 76 |
| 3.No Peer Rec. | 0.04 | 57 | 0.05 | 0.035 | 0.037 | 0.044 | Accuracy | Round 1 76 |
| 1.Expert | 0.692 | 13 | 0.769 | 0.538 | 0.592 | 0.793 | Calibration | Round 1 76 |
| 2.Novice | 0.538 | 6 | 0.712 | 0.481 | 0.391 | 0.686 | Calibration | Round 1 76 |
| 3.No Peer Rec. | 0.692 | 57 | 0.846 | 0.538 | 0.628 | 0.756 | Calibration | Round 1 76 |
| 1.Expert | 0.207 | 13 | 0.246 | 0.171 | 0.175 | 0.24 | Informativeness | Round 1 76 |
| 2.Novice | 0.222 | 6 | 0.25 | 0.151 | 0.158 | 0.286 | Informativeness | Round 1 76 |
| 3.No Peer Rec. | 0.204 | 57 | 0.252 | 0.17 | 0.187 | 0.221 | Informativeness | Round 1 76 |

## Round 1: Publications vs Accuracy, Calibration, Informativeness

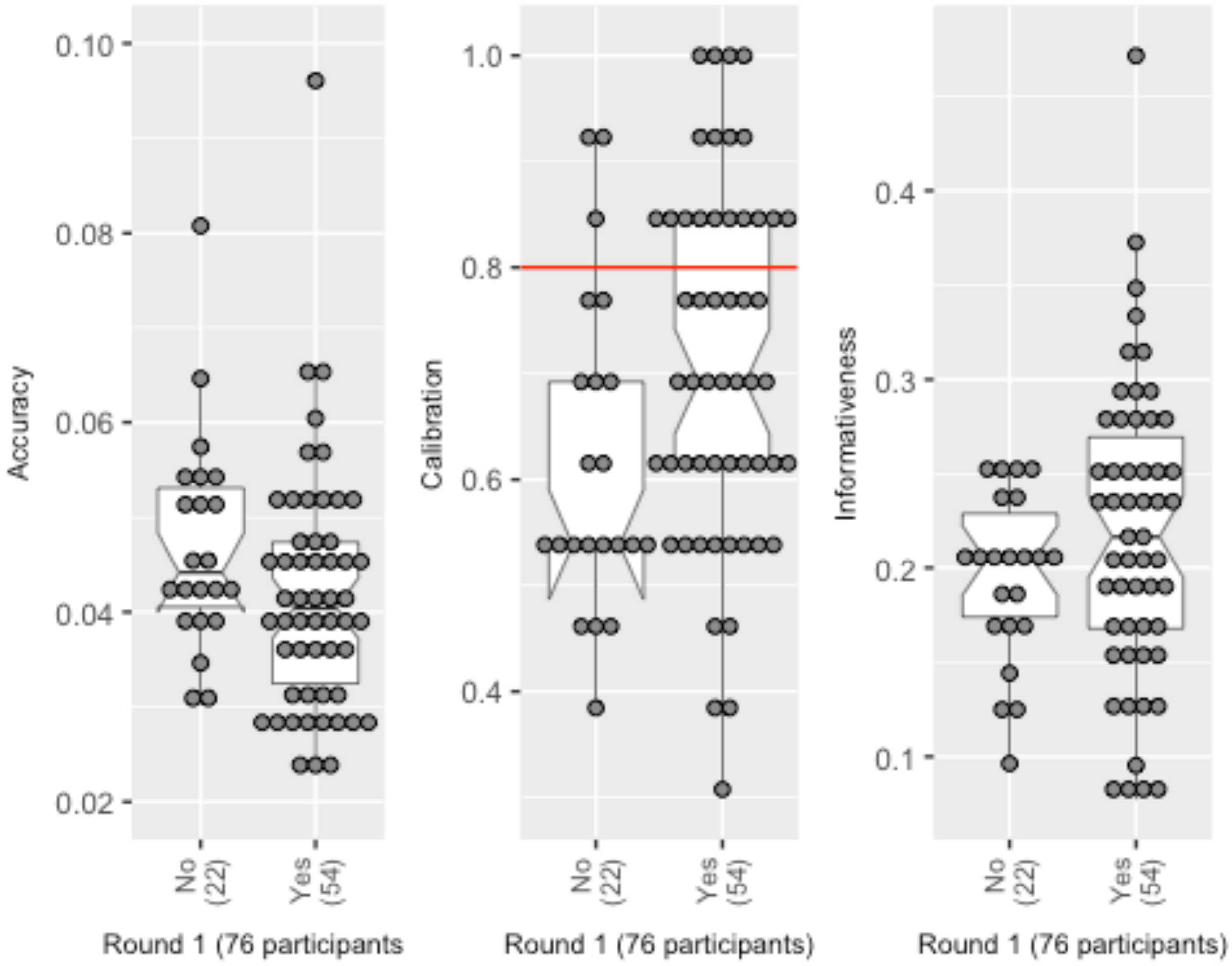


| Test | Median | n | 25th | 75th | LowCI | UpperCI | Stat | Round |
|---|---|---|---|---|---|---|---|---|
| No | 0.044 | 22 | 0.053 | 0.041 | 0.04 | 0.048 | Accuracy | Round 1 76 |
| Yes | 0.04 | 54 | 0.047 | 0.032 | 0.037 | 0.044 | Accuracy | Round 1 76 |
| No | 0.538 | 22 | 0.692 | 0.538 | 0.487 | 0.59 | Calibration | Round 1 76 |
| Yes | 0.692 | 54 | 0.846 | 0.615 | 0.643 | 0.742 | Calibration | Round 1 76 |
| No | 0.204 | 22 | 0.229 | 0.174 | 0.186 | 0.222 | Informativeness | Round 1 76 |
| Yes | 0.217 | 54 | 0.269 | 0.168 | 0.195 | 0.238 | Informativeness | Round 1 76 |

## Round 1: Gender vs Accuracy, Calibration, Informativeness

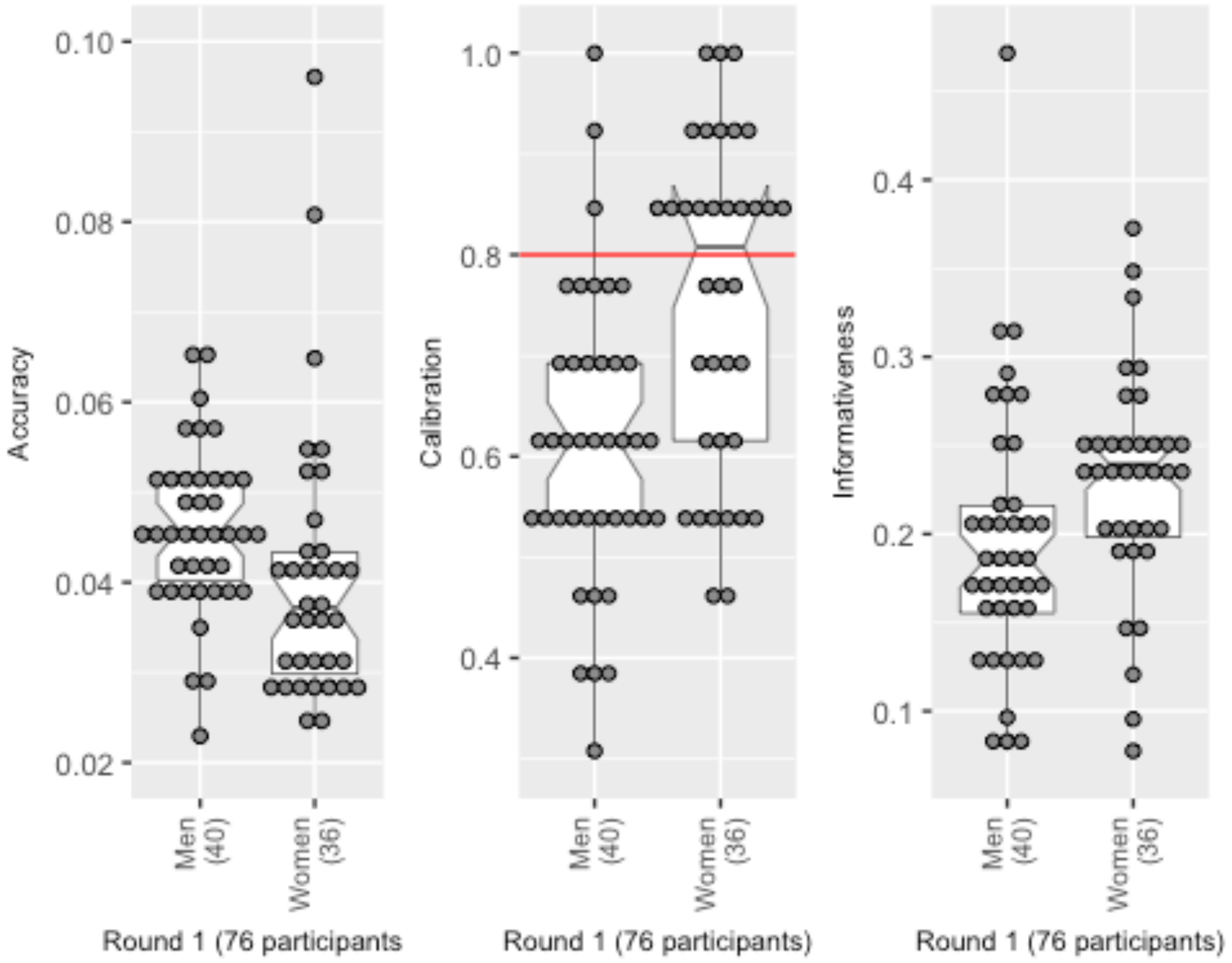


| Test | Median | n | 25th | 75th | LowCI | UpperCI | Stat | Round |
|---|---|---|---|---|---|---|---|---|
| Men | 0.046 | 40 | 0.052 | 0.04 | 0.043 | 0.049 | Accuracy | Round 1 76 |
| Women | 0.037 | 36 | 0.043 | 0.03 | 0.034 | 0.041 | Accuracy | Round 1 76 |
| Men | 0.615 | 40 | 0.692 | 0.538 | 0.577 | 0.654 | Calibration | Round 1 76 |
| Women | 0.808 | 36 | 0.846 | 0.615 | 0.747 | 0.868 | Calibration | Round 1 76 |
| Men | 0.185 | 40 | 0.216 | 0.155 | 0.17 | 0.2 | Informativeness | Round 1 76 |
| Women | 0.239 | 36 | 0.254 | 0.198 | 0.225 | 0.254 | Informativeness | Round 1 76 |

## Round 1: Updated vs Accuracy, Calibration, Informativeness

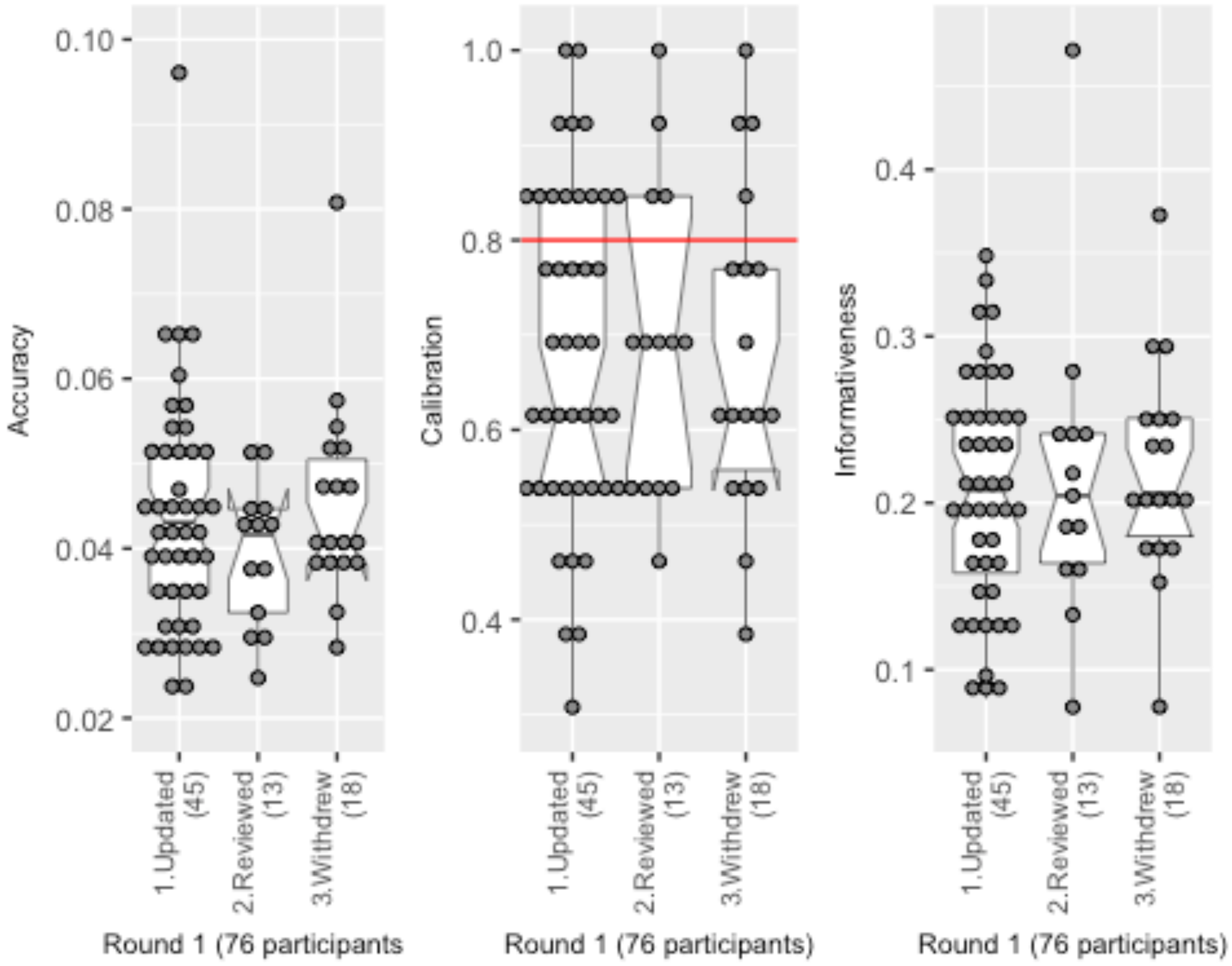


| Test | Median | n | 25th | 75th | LowCI | UpperCI | Stat | Round |
|---|---|---|---|---|---|---|---|---|
| 1.Updated | 0.043 | 45 | 0.052 | 0.035 | 0.039 | 0.047 | Accuracy | Round 1 76 |
| 2.Reviewed | 0.042 | 13 | 0.045 | 0.032 | 0.036 | 0.047 | Accuracy | Round 1 76 |
| 3.Withdrew | 0.041 | 18 | 0.05 | 0.038 | 0.036 | 0.045 | Accuracy | Round 1 76 |
| 1.Updated | 0.615 | 45 | 0.846 | 0.538 | 0.543 | 0.687 | Calibration | Round 1 76 |
| 2.Reviewed | 0.692 | 13 | 0.846 | 0.538 | 0.558 | 0.826 | Calibration | Round 1 76 |
| 3.Withdrew | 0.615 | 18 | 0.769 | 0.558 | 0.537 | 0.694 | Calibration | Round 1 76 |
| 1.Updated | 0.208 | 45 | 0.254 | 0.158 | 0.185 | 0.23 | Informativeness | Round 1 76 |
| 2.Reviewed | 0.204 | 13 | 0.242 | 0.164 | 0.171 | 0.238 | Informativeness | Round 1 76 |
| 3.Withdrew | 0.206 | 18 | 0.251 | 0.18 | 0.18 | 0.232 | Informativeness | Round 1 76 |

## Linear Models

### Number of Publications

```
## integer(0)

##
## Call:
## lm(formula = ResultsB$R1_ScGBR13 ~ ResultsB$X27_Peer.review)
##
## Residuals:
##       Min        1Q    Median        3Q       Max
## -0.020786 -0.008249 -0.001325  0.007405  0.051563
##
## Coefficients:
##                            Estimate Std. Error t value Pr(>|t|)
## (Intercept)               4.453e-02  1.662e-03  26.803   <2e-16 ***
## ResultsB$X27_Peer.review -3.994e-05  4.865e-05  -0.821    0.414
## ---
## Signif. codes:  0 '***' 0.001 '**' 0.01 '*' 0.05 '.' 0.1 ' ' 1
##
## Residual standard error: 0.01276 on 69 degrees of freedom
##   (5 observations deleted due to missingness)
## Multiple R-squared:  0.009673,   Adjusted R-squared:  -0.004679
## F-statistic: 0.674 on 1 and 69 DF,  p-value: 0.4145

## integer(0)

##
## Call:
## lm(formula = ResultsB$R1_ScGBR13 ~ ResultsB$X28_Tech)
##
## Residuals:
##       Min        1Q    Median        3Q       Max
## -0.020703 -0.008705 -0.001602  0.007274  0.052706
##
## Coefficients:
##                     Estimate Std. Error t value Pr(>|t|)
## (Intercept)        4.334e-02  1.673e-03  25.899   <2e-16 ***
## ResultsB$X28_Tech 6.266e-06  2.267e-05   0.276    0.783
## ---
## Signif. codes:  0 '***' 0.001 '**' 0.01 '*' 0.05 '.' 0.1 ' ' 1
##
## Residual standard error: 0.0132 on 66 degrees of freedom
##   (8 observations deleted due to missingness)
## Multiple R-squared:  0.001156,   Adjusted R-squared:  -0.01398
## F-statistic: 0.07642 on 1 and 66 DF,  p-value: 0.7831

## integer(0)
```

```
##
## Call:
## lm(formula = ResultsB$R2_ScGBR13 ~ ResultsB$X27_Peer.review)
##
## Residuals:
##       Min        1Q    Median        3Q       Max
## -0.015694 -0.006713 -0.001612  0.005476  0.041701
##
## Coefficients:
##                           Estimate Std. Error t value Pr(>|t|)
## (Intercept)              3.906e-02  1.356e-03  28.814   <2e-16 ***
## ResultsB$X27_Peer.review 6.905e-06  3.969e-05   0.174    0.862
## ---
## Signif. codes:  0 '***' 0.001 '**' 0.01 '*' 0.05 '.' 0.1 ' ' 1
##
## Residual standard error: 0.01041 on 69 degrees of freedom
##   (5 observations deleted due to missingness)
## Multiple R-squared:  0.0004384,  Adjusted R-squared:  -0.01405
## F-statistic: 0.03026 on 1 and 69 DF,  p-value: 0.8624
```

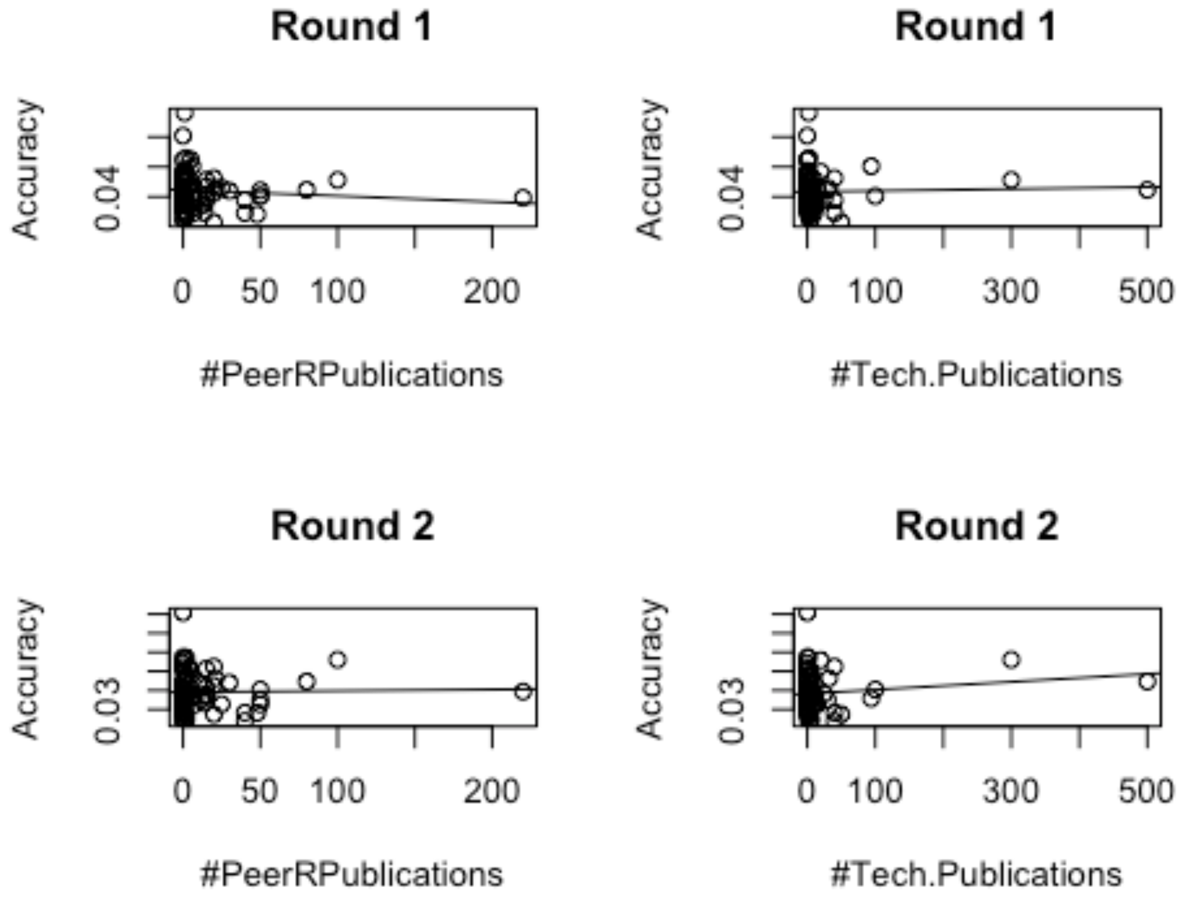

```
## integer(0)

##
## Call:
## lm(formula = ResultsB$R2_ScGBR13 ~ ResultsB$X28_Tech)
##
## Residuals:
##       Min        1Q    Median        3Q       Max
## -0.015156 -0.007066 -0.001569  0.006188  0.042486
##
## Coefficients:
##                    Estimate Std. Error t value Pr(>|t|)
## (Intercept)       3.828e-02  1.322e-03  28.946   <2e-16 ***
## ResultsB$X28_Tech 2.066e-05  1.791e-05   1.154    0.253
## ---
## Signif. codes:  0 '***' 0.001 '**' 0.01 '*' 0.05 '.' 0.1 ' ' 1
##
## Residual standard error: 0.01043 on 66 degrees of freedom
##   (8 observations deleted due to missingness)
## Multiple R-squared:  0.01976,    Adjusted R-squared:  0.00491
## F-statistic: 1.331 on 1 and 66 DF,  p-value: 0.2529

## integer(0)

##
## Call:
## lm(formula = ResultsB$R1_HiGBR13 ~ ResultsB$X27_Peer.review)
##
## Residuals:
##      Min       1Q   Median       3Q      Max
## -0.35584 -0.12344 -0.04651  0.10733  0.33756
##
## Coefficients:
##                           Estimate Std. Error t value Pr(>|t|)
## (Intercept)              0.6618989  0.0215442  30.723   <2e-16 ***
## ResultsB$X27_Peer.review 0.0005453  0.0006308   0.864     0.39
## ---
## Signif. codes:  0 '***' 0.001 '**' 0.01 '*' 0.05 '.' 0.1 ' ' 1
##
## Residual standard error: 0.1655 on 69 degrees of freedom
##   (5 observations deleted due to missingness)
## Multiple R-squared:  0.01071,    Adjusted R-squared:  -0.003623
## F-statistic: 0.7473 on 1 and 69 DF,  p-value: 0.3903

## integer(0)

##
## Call:
## lm(formula = ResultsB$R1_HiGBR13 ~ ResultsB$X28_Tech)
##
## Residuals:
```

```
##      Min       1Q   Median       3Q      Max 
## -0.29628 -0.14226 -0.06402  0.16542  0.32102 
## 
## Coefficients:
##                       Estimate Std. Error t value Pr(>|t|)    
## (Intercept)         6.809e-01  2.170e-02  31.371   <2e-16 ***
## ResultsB$X28_Tech -4.779e-05  2.940e-04  -0.163    0.871    
## ---
## Signif. codes:  0 '***' 0.001 '**' 0.01 '*' 0.05 '.' 0.1 ' ' 1
## 
## Residual standard error: 0.1712 on 66 degrees of freedom
##   (8 observations deleted due to missingness)
## Multiple R-squared:  0.0004003,  Adjusted R-squared:  -0.01475 
## F-statistic: 0.02643 on 1 and 66 DF,  p-value: 0.8713

## integer(0)

## 
## Call:
## lm(formula = ResultsB$R2_HiGBR13 ~ ResultsB$X27_Peer.review)
## 
## Residuals:
##      Min       1Q   Median       3Q      Max 
## -0.46533 -0.08123 -0.00350  0.07357  0.30434 
## 
## Coefficients:
##                           Estimate Std. Error t value Pr(>|t|)    
## (Intercept)              0.6956565  0.0198980  34.961   <2e-16 ***
## ResultsB$X27_Peer.review 0.0001473  0.0005826   0.253    0.801    
## ---
## Signif. codes:  0 '***' 0.001 '**' 0.01 '*' 0.05 '.' 0.1 ' ' 1
## 
## Residual standard error: 0.1528 on 69 degrees of freedom
##   (5 observations deleted due to missingness)
## Multiple R-squared:  0.0009255,  Adjusted R-squared:  -0.01355 
## F-statistic: 0.06392 on 1 and 69 DF,  p-value: 0.8012
```

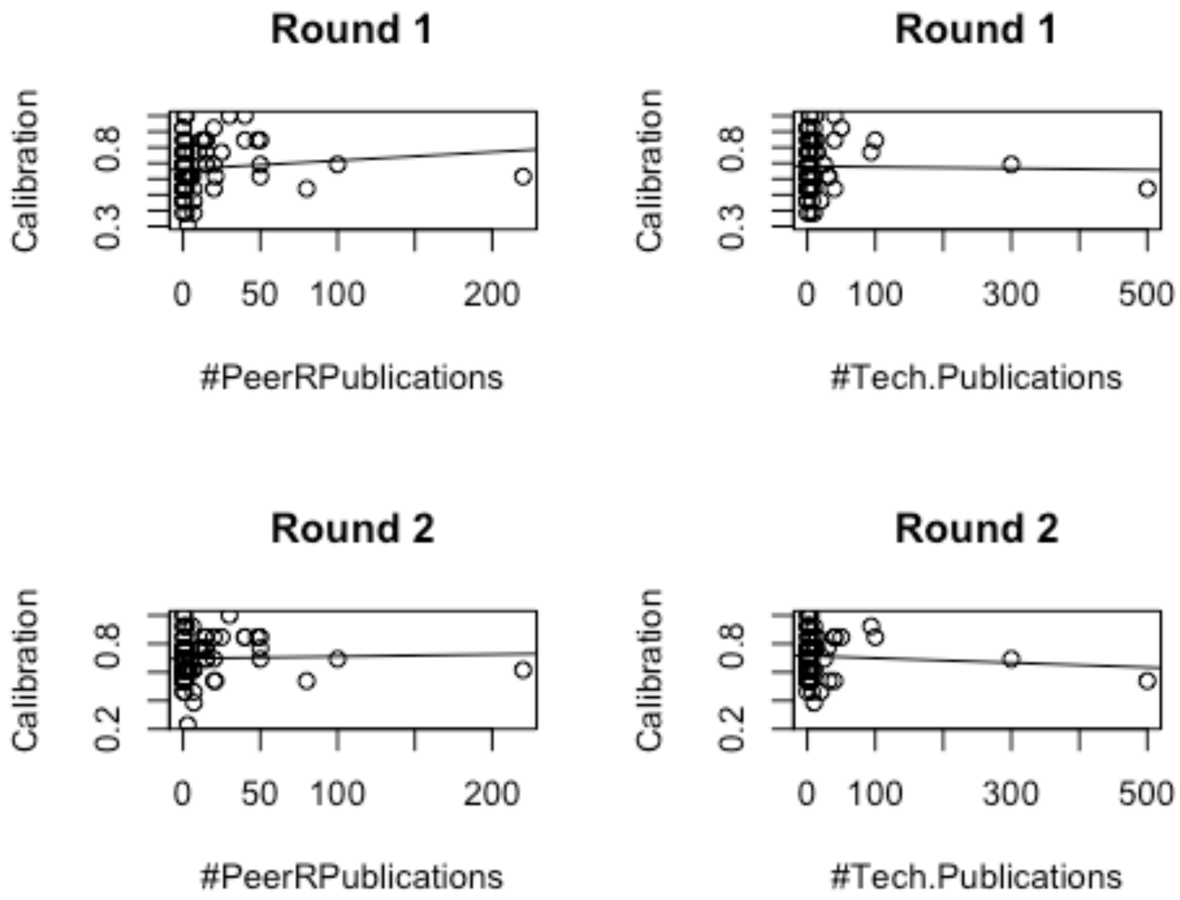


```
## integer(0)

##
## Call:
## lm(formula = ResultsB$R2_HiGBR13 ~ ResultsB$X28_Tech)
##
## Residuals:
##      Min       1Q   Median       3Q      Max
## -0.32879 -0.09965 -0.02029  0.13173  0.28578
##
## Coefficients:
##                    Estimate Std. Error t value Pr(>|t|)
## (Intercept)        0.7150382  0.0190973   37.44   <2e-16 ***
## ResultsB$X28_Tech -0.0001630  0.0002587   -0.63    0.531
## ---
## Signif. codes:  0 '***' 0.001 '**' 0.01 '*' 0.05 '.' 0.1 ' ' 1
##
## Residual standard error: 0.1507 on 66 degrees of freedom
##   (8 observations deleted due to missingness)
```

```
## Multiple R-squared:  0.005981,   Adjusted R-squared:  -0.00908
## F-statistic: 0.3971 on 1 and 66 DF,  p-value: 0.5308

## integer(0)

##
## Call:
## lm(formula = ResultsB$R1_InGBR13 ~ ResultsB$X27_Peer.review)
##
## Residuals:
##       Min        1Q    Median        3Q       Max
## -0.130564 -0.041895 -0.003052  0.045005  0.261441
##
## Coefficients:
##                           Estimate Std. Error t value Pr(>|t|)
## (Intercept)              2.074e-01  9.270e-03  22.373   <2e-16 ***
## ResultsB$X27_Peer.review 8.985e-05  2.714e-04   0.331    0.742
## ---
## Signif. codes:  0 '***' 0.001 '**' 0.01 '*' 0.05 '.' 0.1 ' ' 1
##
## Residual standard error: 0.07121 on 69 degrees of freedom
##   (5 observations deleted due to missingness)
## Multiple R-squared:  0.001586,   Adjusted R-squared:  -0.01288
## F-statistic: 0.1096 on 1 and 69 DF,  p-value: 0.7416

## integer(0)

##
## Call:
## lm(formula = ResultsB$R1_InGBR13 ~ ResultsB$X28_Tech)
##
## Residuals:
##       Min        1Q    Median        3Q       Max
## -0.134653 -0.041979 -0.007586  0.040905  0.259471
##
## Coefficients:
##                    Estimate Std. Error t value Pr(>|t|)
## (Intercept)       2.119e-01  9.182e-03  23.082   <2e-16 ***
## ResultsB$X28_Tech 2.623e-05  1.244e-04   0.211    0.834
## ---
## Signif. codes:  0 '***' 0.001 '**' 0.01 '*' 0.05 '.' 0.1 ' ' 1
##
## Residual standard error: 0.07245 on 66 degrees of freedom
##   (8 observations deleted due to missingness)
## Multiple R-squared:  0.0006733,  Adjusted R-squared:  -0.01447
## F-statistic: 0.04447 on 1 and 66 DF,  p-value: 0.8336

## integer(0)

##
## Call:
```

```
## lm(formula = ResultsB$R2_InGBR13 ~ ResultsB$X27_Peer.review)
##
## Residuals:
##       Min        1Q    Median        3Q       Max
## -0.128275 -0.043566 -0.001508  0.043299  0.263668
##
## Coefficients:
##                           Estimate Std. Error t value Pr(>|t|)
## (Intercept)              2.051e-01  8.382e-03  24.468   <2e-16 ***
## ResultsB$X27_Peer.review 9.251e-05  2.454e-04   0.377    0.707
## ---
## Signif. codes:  0 '***' 0.001 '**' 0.01 '*' 0.05 '.' 0.1 ' ' 1
##
## Residual standard error: 0.06439 on 69 degrees of freedom
##   (5 observations deleted due to missingness)
## Multiple R-squared:  0.002055,   Adjusted R-squared:  -0.01241
## F-statistic: 0.1421 on 1 and 69 DF,  p-value: 0.7074
```

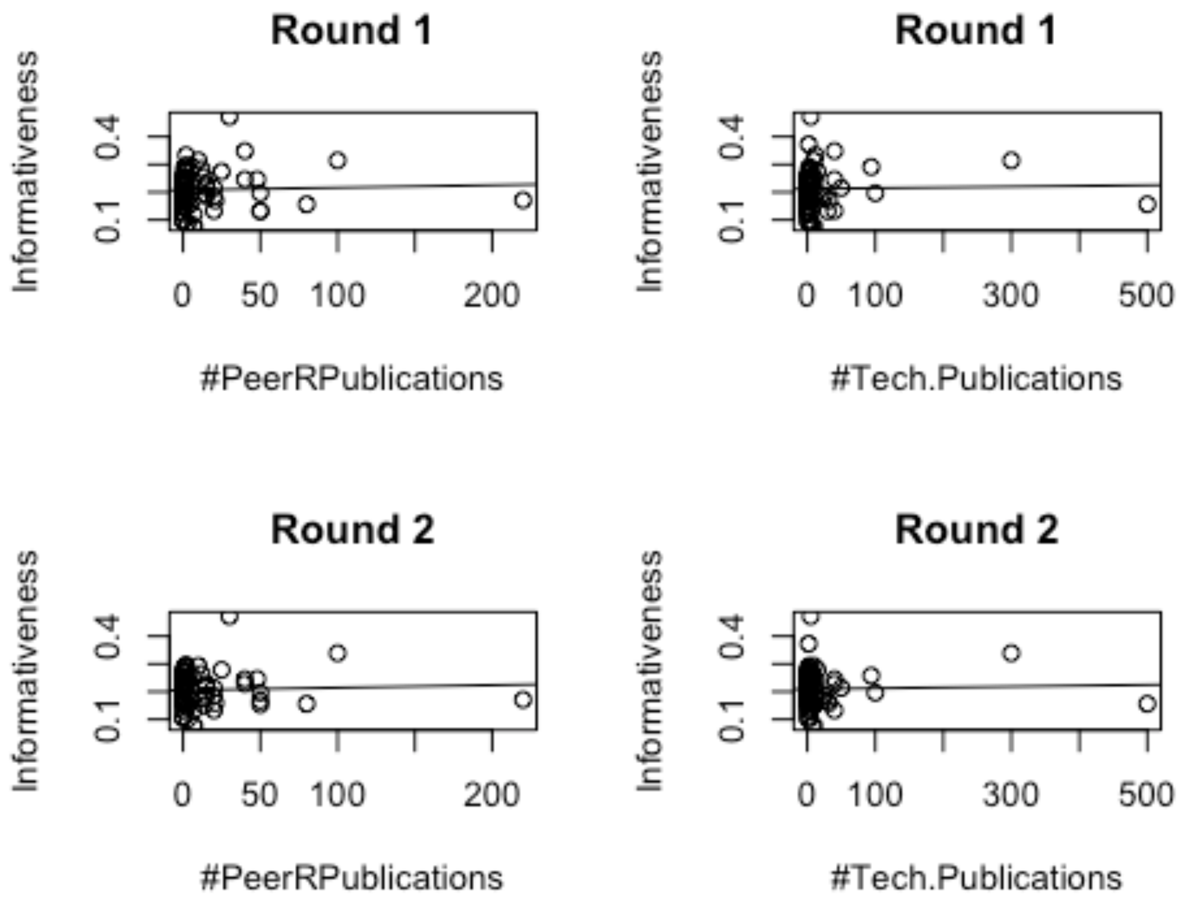


```
## integer(0)
```

```
##
## Call:
## lm(formula = ResultsB$R2_InGBR13 ~ ResultsB$X28_Tech)
##
## Residuals:
##       Min        1Q    Median        3Q       Max
## -0.131792 -0.042805 -0.005075  0.038197  0.262341
##
## Coefficients:
##                     Estimate Std. Error t value Pr(>|t|)
## (Intercept)        2.090e-01  8.391e-03  24.913   <2e-16 ***
## ResultsB$X28_Tech 3.084e-05  1.136e-04   0.271    0.787
## ---
## Signif. codes:  0 '***' 0.001 '**' 0.01 '*' 0.05 '.' 0.1 ' ' 1
##
## Residual standard error: 0.0662 on 66 degrees of freedom
##   (8 observations deleted due to missingness)
## Multiple R-squared:  0.001114,   Adjusted R-squared:  -0.01402
## F-statistic: 0.07363 on 1 and 66 DF,  p-value: 0.787
```

### Self-rating and accuracy in a domain (Round 1)

```
##
## Call:
## lm(formula = R1_Q1ALREabs1 ~ X20_COTS_Rate, data = ResultsB)
##
## Residuals:
##       Min        1Q    Median        3Q       Max
## -0.022382 -0.018030 -0.012057  0.004669  0.276985
##
## Coefficients:
##                Estimate Std. Error t value Pr(>|t|)
## (Intercept)    0.023033   0.007706   2.989   0.0038 **
## X20_COTS_Rate -0.001378   0.001568  -0.879   0.3822
## ---
## Signif. codes:  0 '***' 0.001 '**' 0.01 '*' 0.05 '.' 0.1 ' ' 1
##
## Residual standard error: 0.04098 on 74 degrees of freedom
## Multiple R-squared:  0.01034,   Adjusted R-squared:  -0.003038
## F-statistic: 0.7729 on 1 and 74 DF,  p-value: 0.3822

##
##  Spearman's rank correlation rho
##
## data:  ResultsB$R1_Q1ALREabs1 and ResultsB$X20_COTS_Rate
## S = 75253, p-value = 0.8053
## alternative hypothesis: true rho is not equal to 0
## sample estimates:
##         rho
## -0.02874416
```

```
##
## Call:
## lm(formula = R1_Q2ALREabs1 ~ X21_Coral_Rate, data = ResultsB)
##
## Residuals:
##      Min       1Q   Median       3Q      Max
## -0.07829 -0.05801 -0.02025  0.06162  0.14388
##
## Coefficients:
##                 Estimate Std. Error t value Pr(>|t|)
## (Intercept)    8.865e-02  1.480e-02   5.988 7.06e-08 ***
## X21_Coral_Rate 1.273e-05  2.764e-03   0.005    0.996
## ---
## Signif. codes:  0 '***' 0.001 '**' 0.01 '*' 0.05 '.' 0.1 ' ' 1
##
## Residual standard error: 0.0654 on 74 degrees of freedom
## Multiple R-squared:  2.867e-07,  Adjusted R-squared:  -0.01351
## F-statistic: 2.121e-05 on 1 and 74 DF,  p-value: 0.9963

##
##  Spearman's rank correlation rho
##
## data:  ResultsB$R1_Q2ALREabs1 and ResultsB$X21_Coral_Rate
## S = 76271, p-value = 0.7144
## alternative hypothesis: true rho is not equal to 0
## sample estimates:
##         rho
## -0.04266864

##
## Call:
## lm(formula = BioInvSc3 ~ X22_Bioinvasions_Rate, data = ResultsB)
##
## Residuals:
##       Min        1Q    Median        3Q       Max
## -0.024156 -0.011790 -0.005720  0.002137  0.272189
##
## Coefficients:
##                        Estimate Std. Error t value Pr(>|t|)
## (Intercept)            0.028504   0.007760   3.673  0.00045 ***
## X22_Bioinvasions_Rate -0.002333   0.001322  -1.765  0.08170 .
## ---
## Signif. codes:  0 '***' 0.001 '**' 0.01 '*' 0.05 '.' 0.1 ' ' 1
##
## Residual standard error: 0.0352 on 74 degrees of freedom
## Multiple R-squared:  0.04039,    Adjusted R-squared:  0.02743
## F-statistic: 3.115 on 1 and 74 DF,  p-value: 0.0817

##
##  Spearman's rank correlation rho
```

```
##
## data:  ResultsB$BioInvSc3 and ResultsB$X22_Bioinvasions_Rate
## S = 80392, p-value = 0.3949
## alternative hypothesis: true rho is not equal to 0
## sample estimates:
##         rho
## -0.09900031
```

```
##
## Call:
## lm(formula = WaterSc ~ X23_Water_Rate, data = ResultsB)
##
## Residuals:
##       Min        1Q    Median        3Q       Max
## -0.040332 -0.016101 -0.002901  0.014040  0.057340
##
## Coefficients:
##                  Estimate Std. Error t value Pr(>|t|)
## (Intercept)     0.0453011  0.0048532   9.334  3.9e-14 ***
## X23_Water_Rate -0.0004169  0.0008536  -0.488    0.627
## ---
## Signif. codes:  0 '***' 0.001 '**' 0.01 '*' 0.05 '.' 0.1 ' ' 1
##
## Residual standard error: 0.02169 on 74 degrees of freedom
## Multiple R-squared:  0.003213,   Adjusted R-squared:  -0.01026
## F-statistic: 0.2386 on 1 and 74 DF,  p-value: 0.6267
```

```
##
##  Spearman's rank correlation rho
##
## data:  ResultsB$WaterSc and ResultsB$X23_Water_Rate
## S = 74191, p-value = 0.9029
## alternative hypothesis: true rho is not equal to 0
## sample estimates:
##        rho
## -0.0142338
```

```
##
##  Pearson's product-moment correlation
##
## data:  ResultsB$WaterSc and ResultsB$X23_Water_Rate
## t = -0.48842, df = 74, p-value = 0.6267
## alternative hypothesis: true correlation is not equal to 0
## 95 percent confidence interval:
##  -0.2785817  0.1709539
## sample estimates:
##         cor
## -0.05668652
```

```
##
## Call:
```

```
## lm(formula = Climate ~ X25_Climate_Rate, data = ResultsB)
##
## Residuals:
##       Min        1Q    Median        3Q       Max
## -0.033204 -0.009147 -0.004807  0.001793  0.061014
##
## Coefficients:
##                    Estimate Std. Error t value Pr(>|t|)
## (Intercept)       0.0483904  0.0040893  11.833   <2e-16 ***
## X25_Climate_Rate -0.0011364  0.0008393  -1.354     0.18
## ---
## Signif. codes:  0 '***' 0.001 '**' 0.01 '*' 0.05 '.' 0.1 ' ' 1
##
## Residual standard error: 0.01943 on 74 degrees of freedom
## Multiple R-squared:  0.02418,    Adjusted R-squared:  0.01099
## F-statistic: 1.833 on 1 and 74 DF,  p-value: 0.1799
```

```
##
##  Spearman's rank correlation rho
##
## data:  ResultsB$Climate and ResultsB$X25_Climate_Rate
## S = 85658, p-value = 0.1397
## alternative hypothesis: true rho is not equal to 0
## sample estimates:
##        rho
## -0.1709957
```

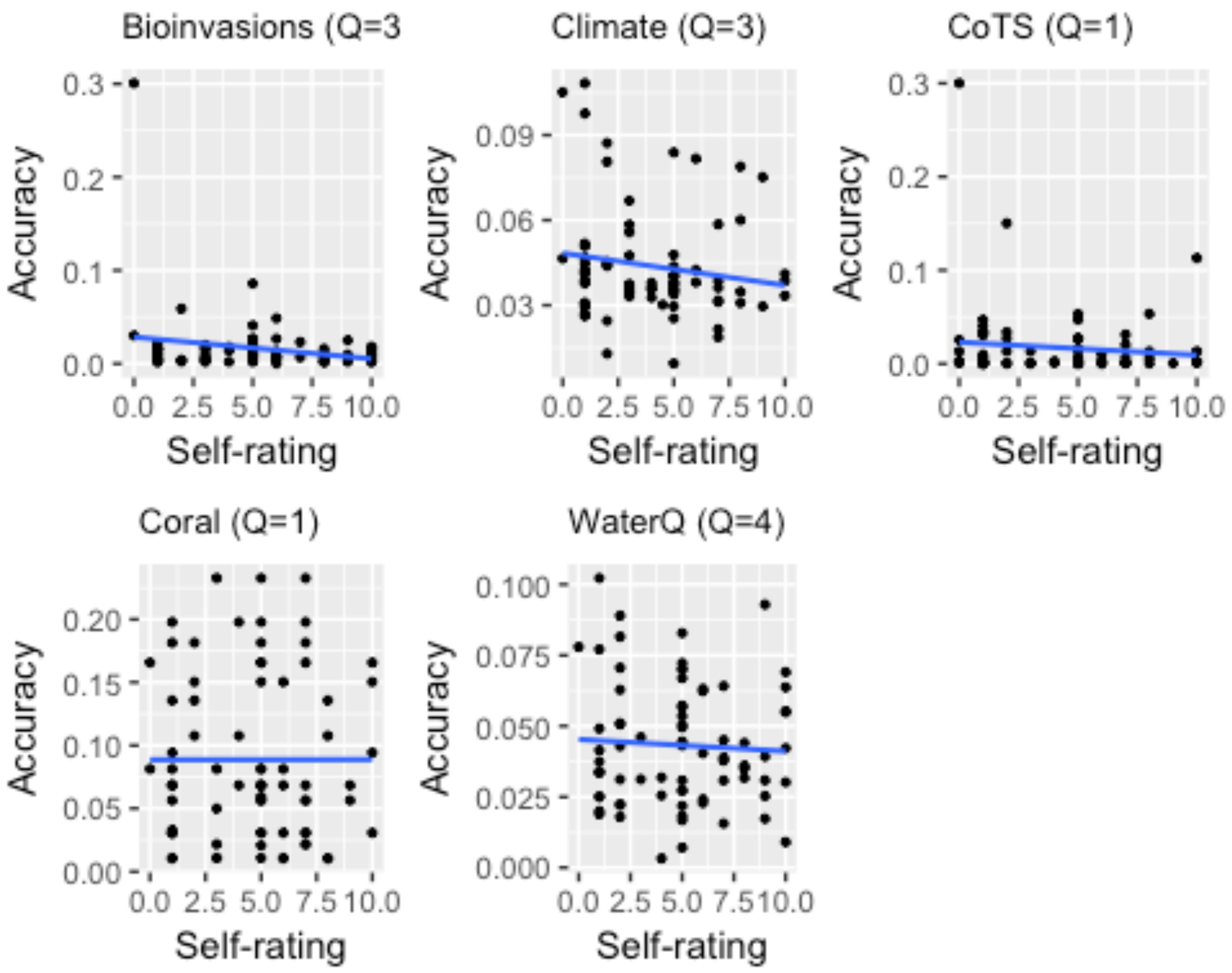


## Self-rating vs years of experience

```
##
## Formula: X20_COTS_Rate ~ SSasymp(X14_COTS_Yr, Asym, R0, lrc)
##
## Parameters:
##      Estimate Std. Error t value Pr(>|t|)
## Asym   7.4474     0.5172  14.398  < 2e-16 ***
## R0     1.9812     0.2886   6.865 1.85e-09 ***
## lrc   -0.6069     0.3667  -1.655    0.102
## ---
## Signif. codes:  0 '***' 0.001 '**' 0.01 '*' 0.05 '.' 0.1 ' ' 1
##
## Residual standard error: 1.908 on 73 degrees of freedom
##
## Number of iterations to convergence: 3
## Achieved convergence tolerance: 9.98e-07
```

```
##
## Formula: X21_Coral_Rate ~ SSasymp(X15_Coral_Yr, Asym, R0, lrc)
##
## Parameters:
##      Estimate Std. Error t value Pr(>|t|)
## Asym   7.0325     0.3488  20.161  < 2e-16 ***
## R0     2.3245     0.3023   7.690 5.37e-11 ***
## lrc   -0.6386     0.3315  -1.927   0.0579 .
## ---
## Signif. codes:  0 '***' 0.001 '**' 0.01 '*' 0.05 '.' 0.1 ' ' 1
##
## Residual standard error: 1.731 on 73 degrees of freedom
##
## Number of iterations to convergence: 6
## Achieved convergence tolerance: 4.091e-06
```

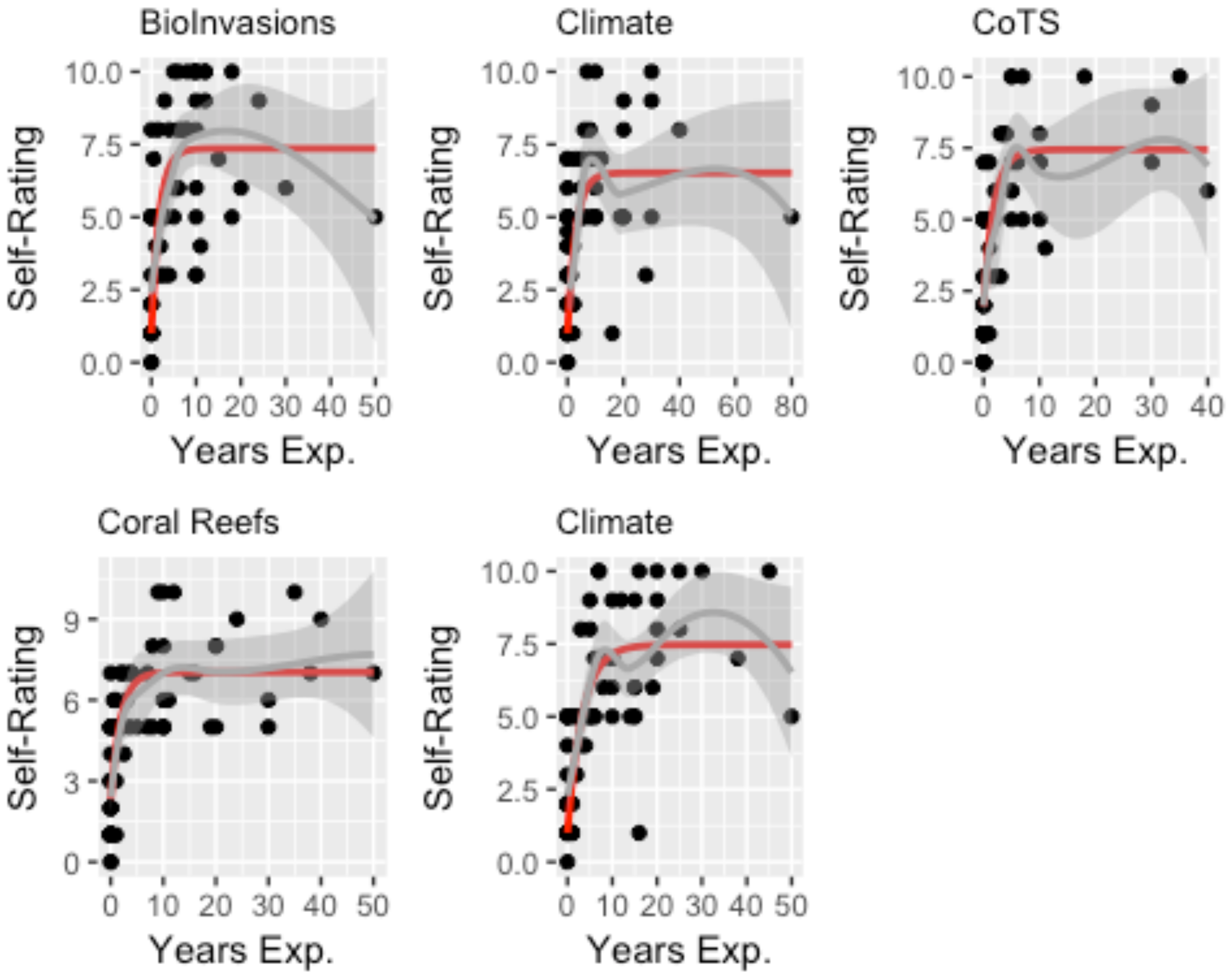

## Round 2: Demographics and performance

### Round 2: Updated vs Accuracy, Calibration, Informativeness

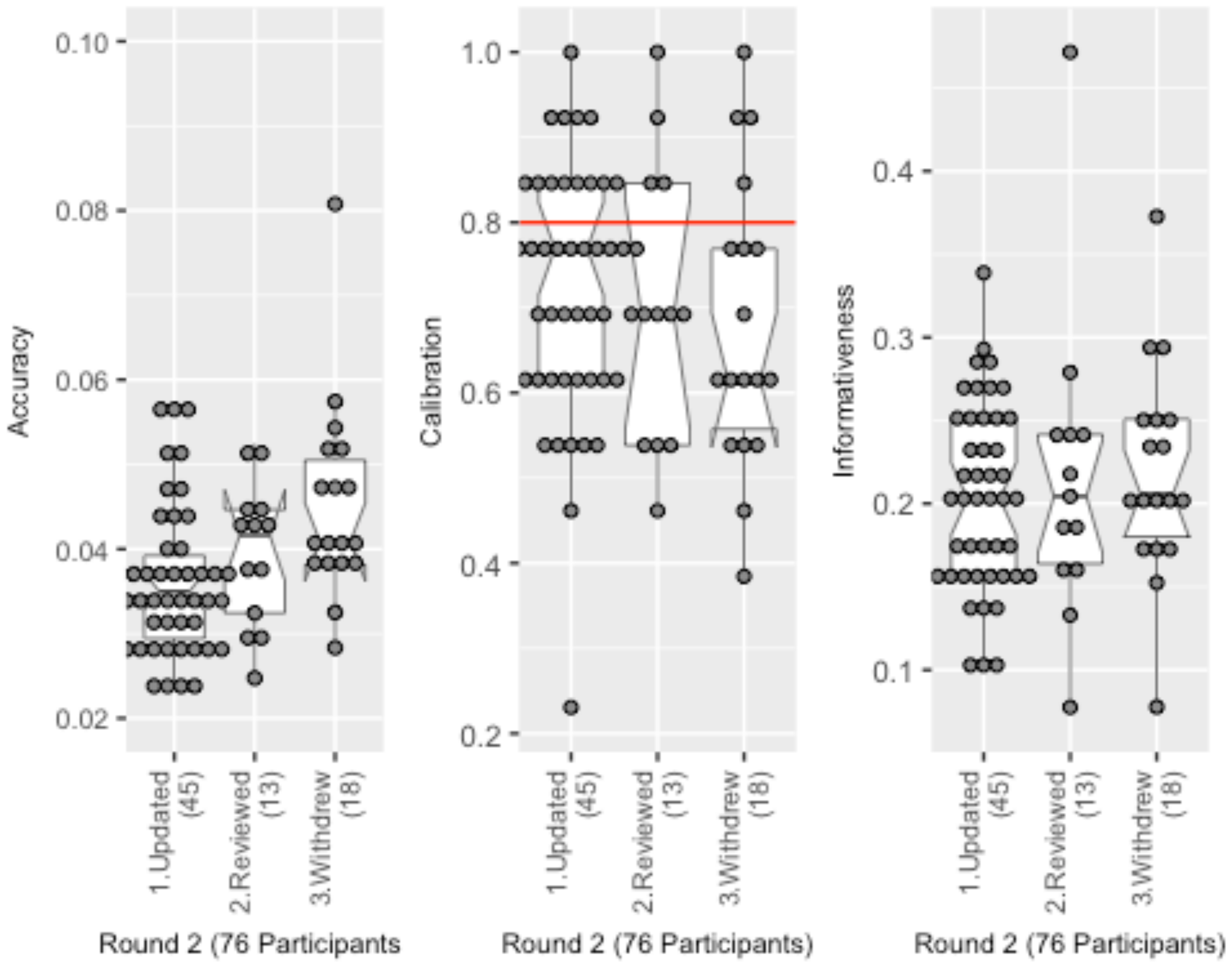


| Test | Median | n | 25th | 75th | LowCI | UpperCI | Stat | Round |
|---|---|---|---|---|---|---|---|---|
| 1.Updated | 0.035 | 45 | 0.039 | 0.029 | 0.033 | 0.037 | Accuracy | Round 2 |
| 2.Reviewed | 0.042 | 13 | 0.045 | 0.032 | 0.036 | 0.047 | Accuracy | Round 2 |
| 3.Withdrew | 0.041 | 18 | 0.05 | 0.038 | 0.036 | 0.045 | Accuracy | Round 2 |
| 1.Updated | 0.769 | 45 | 0.846 | 0.615 | 0.715 | 0.823 | Calibration | Round 2 |
| 2.Reviewed | 0.692 | 13 | 0.846 | 0.538 | 0.558 | 0.826 | Calibration | Round 2 |
| 3.Withdrew | 0.615 | 18 | 0.769 | 0.558 | 0.537 | 0.694 | Calibration | Round 2 |
| 1.Updated | 0.203 | 45 | 0.251 | 0.159 | 0.181 | 0.225 | Informativeness | Round 2 |
| 2.Reviewed | 0.204 | 13 | 0.242 | 0.164 | 0.171 | 0.238 | Informativeness | Round 2 |
| 3.Withdrew | 0.206 | 18 | 0.251 | 0.18 | 0.18 | 0.232 | Informativeness | Round 2 |

## Correlation between Round 1 and Round 2

```
## #Accuracy

##
## Call:
## lm(formula = R1_ScGBR13 ~ R2_ScGBR13, data = ResultsB)
##
## Residuals:
##       Min        1Q    Median        3Q       Max
## -0.013878 -0.006726 -0.003313  0.002644  0.059119
##
## Coefficients:
##             Estimate Std. Error t value Pr(>|t|)
## (Intercept)  0.01858    0.00663   2.802 0.006957 **
## R2_ScGBR13   0.65638    0.17526   3.745 0.000428 ***
## ---
## Signif. codes:  0 '***' 0.001 '**' 0.01 '*' 0.05 '.' 0.1 ' ' 1
##
## Residual standard error: 0.01162 on 56 degrees of freedom
## Multiple R-squared:  0.2003, Adjusted R-squared:  0.186
## F-statistic: 14.03 on 1 and 56 DF,  p-value: 0.0004276

## #Calibration

##
## Call:
## lm(formula = R1_HiGBR13 ~ R2_HiGBR13, data = ResultsB)
##
## Residuals:
##      Min       1Q   Median       3Q      Max
## -0.26609 -0.05212  0.01641  0.04999  0.27237
##
## Coefficients:
##             Estimate Std. Error t value Pr(>|t|)
## (Intercept)  0.04236    0.06900   0.614    0.542
## R2_HiGBR13   0.89085    0.09434   9.443 3.49e-13 ***
## ---
## Signif. codes:  0 '***' 0.001 '**' 0.01 '*' 0.05 '.' 0.1 ' ' 1
##
## Residual standard error: 0.1068 on 56 degrees of freedom
## Multiple R-squared:  0.6143, Adjusted R-squared:  0.6074
## F-statistic: 89.18 on 1 and 56 DF,  p-value: 3.493e-13

## #Informativeness

##
## Call:
## lm(formula = R1_InGBR13 ~ R2_InGBR13, data = ResultsB)
##
## Residuals:
```

```
##       Min        1Q    Median        3Q       Max
## -0.085447 -0.019554 -0.003806  0.007593  0.115012
##
## Coefficients:
##             Estimate Std. Error t value Pr(>|t|)
## (Intercept)  0.01245    0.01713   0.727    0.471
## R2_InGBR13   0.95362    0.07923  12.036   <2e-16 ***
## ---
## Signif. codes:  0 '***' 0.001 '**' 0.01 '*' 0.05 '.' 0.1 ' ' 1
##
## Residual standard error: 0.03929 on 56 degrees of freedom
## Multiple R-squared:  0.7212, Adjusted R-squared:  0.7162
## F-statistic: 144.9 on 1 and 56 DF,  p-value: < 2.2e-16
```

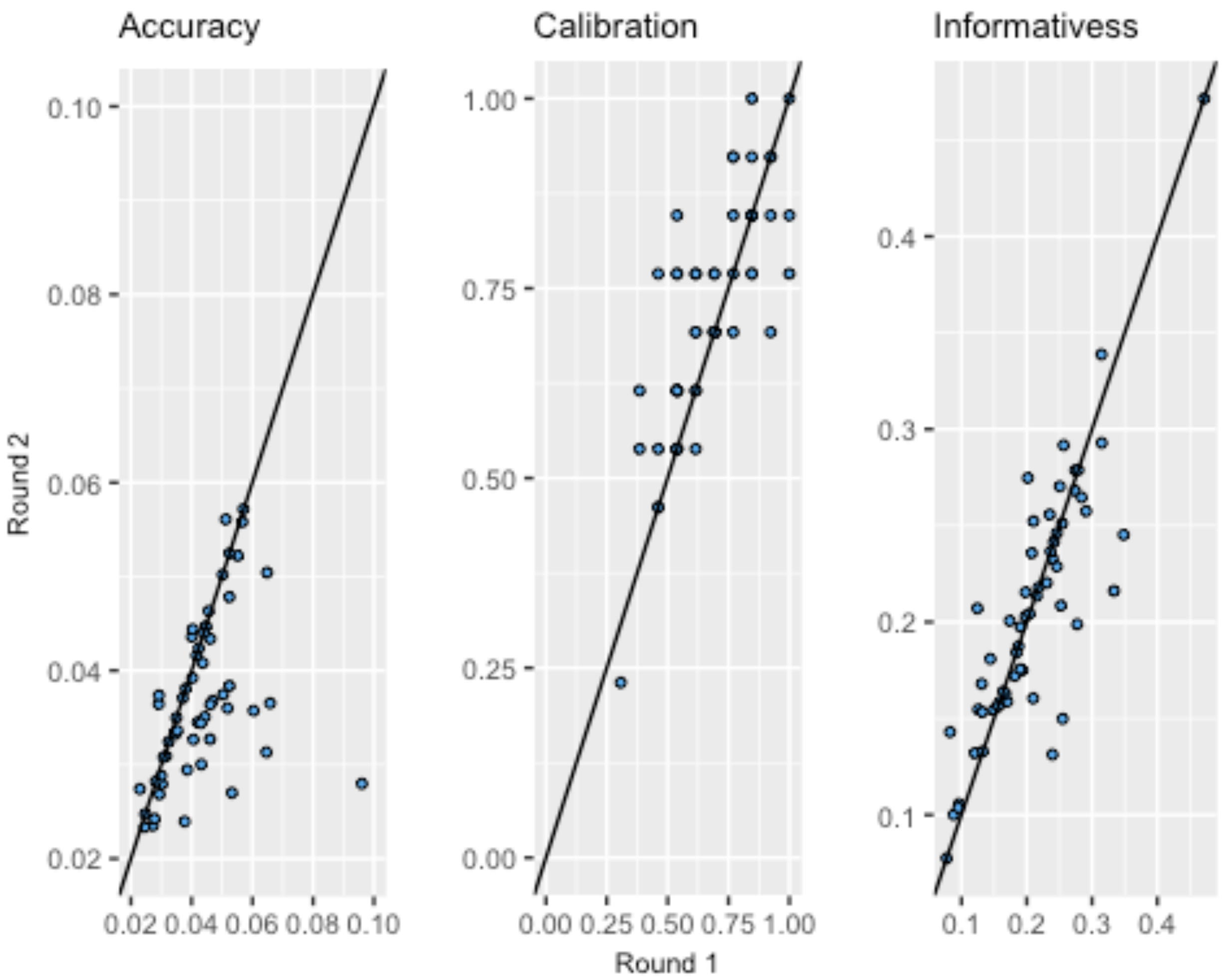

## Change in Accuracy

*Individual change in accuracy for those who updated vs direction of change*

| Change | n | Min | 25th | Median | Mean | 75th | Max | LowMedCI | UpperMedCI | Sd | Stat |
|---|---|---|---|---|---|---|---|---|---|---|---|
| Better | 36 | 0.001 | 0.002 | 0.008 | 0.01 | 0.013 | 0.068 | 0.005 | 0.011 | 0.013 | Change Accuray R1-R2 |
| Worse | 8 | -0.008 | -0.005 | -0.004 | -0.004 | -0.003 | 0 | -0.006 | -0.003 | 0.003 | Change Accuray R1-R2 |

*Individual change in accuracy for those who updated*

| n | Min | 25th | Median | Mean | 75th | Max | LowMedCI | UpperMedCI | Sd | Stat |
|---|---|---|---|---|---|---|---|---|---|---|
| 44 | 0 | 0.002 | 0.005 | 0.009 | 0.013 | 0.068 | 0.002 | 0.007 | 0.012 | Change Accuracy R1-R2 |

*Number of questions those who updated a best guess updated*

| n | Min | 25th | Median | Mean | 75th | Max | LowMedCI | UpperMedCI | Sd | Stat |
|---|---|---|---|---|---|---|---|---|---|---|
| 44 | 1 | 2 | 3 | 3.705 | 6 | 10 | 2.053 | 3.947 | 2.378 | #Qs Best Guess Updated |

*Proportion of questions updated where accuracy improved*

| n | Min | 25th | Median | Mean | 75th | Max | LowMedCI | UpperMedCI | Sd | Stat |
|---|---|---|---|---|---|---|---|---|---|---|
| 44 | 0 | 0.5 | 0.667 | 0.653 | 1 | 1 | 0.548 | 0.785 | 0.302 | #Prop Qs Best Guess Updated improved |

## Change in calibration (distance from 0.80) vs direction of change

*Distance from 0.80 vs direction of change*

| Change | n | Min | 25th | Median | Mean | 75th | Max | LowMedCI | UpperMedCI | Sd | Stat |
|---|---|---|---|---|---|---|---|---|---|---|---|
| Better | 24 | 0.015 | 0.077 | 0.077 | 0.121 | 0.158 | 0.308 | 0.051 | 0.103 | 0.078 | Change Calibration |

| | | | | | | | | | | | |
|---|---|---|---|---|---|---|---|---|---|---|---|
| No Diff | 13 | 0 | 0 | 0 | 0 | 0 | 0 | 0 | 0 | 0 | Change Calibration |
| Worse | 8 | -0.154 | -0.092 | -0.077 | -0.083 | -0.077 | -0.015 | -0.085 | -0.068 | 0.038 | Change Calibration |

*Number of realisations captured vs direction*

| Change | n | Min | 25th | Median | Mean | 75th | Max | LowMedCI | UpperMedCI | Sd | Stat |
|---|---|---|---|---|---|---|---|---|---|---|---|
| Better | 23 | 0.077 | 0.077 | 0.077 | 0.137 | 0.154 | 0.308 | 0.052 | 0.102 | 0.077 | Change Calibration |
| No Diff | 13 | 0 | 0 | 0 | 0 | 0 | 0 | 0 | 0 | 0 | Change Calibration |
| Worse | 9 | -0.231 | -0.154 | -0.077 | -0.12 | -0.077 | -0.077 | -0.117 | -0.037 | 0.068 | Change Calibration |

*Change in informativeness x change in number of realisations captured*

| Change | n | Min | 25th | Median | Mean | 75th | Max | LowMedCI | UpperMedCI | Sd | Stat |
|---|---|---|---|---|---|---|---|---|---|---|---|
| Better | 23 | -0.078 | 0.002 | 0.011 | 0.013 | 0.029 | 0.083 | 0.003 | 0.02 | 0.036 | Change Calibration |
| No Diff | 13 | -0.105 | -0.017 | -0.007 | -0.007 | 0.021 | 0.042 | -0.023 | 0.01 | 0.038 | Change Calibration |
| Worse | 9 | -0.117 | -0.103 | -0.018 | -0.041 | -0.003 | 0.012 | -0.071 | 0.034 | 0.052 | Change Calibration |

## All 76 participants: Group vs individual performance

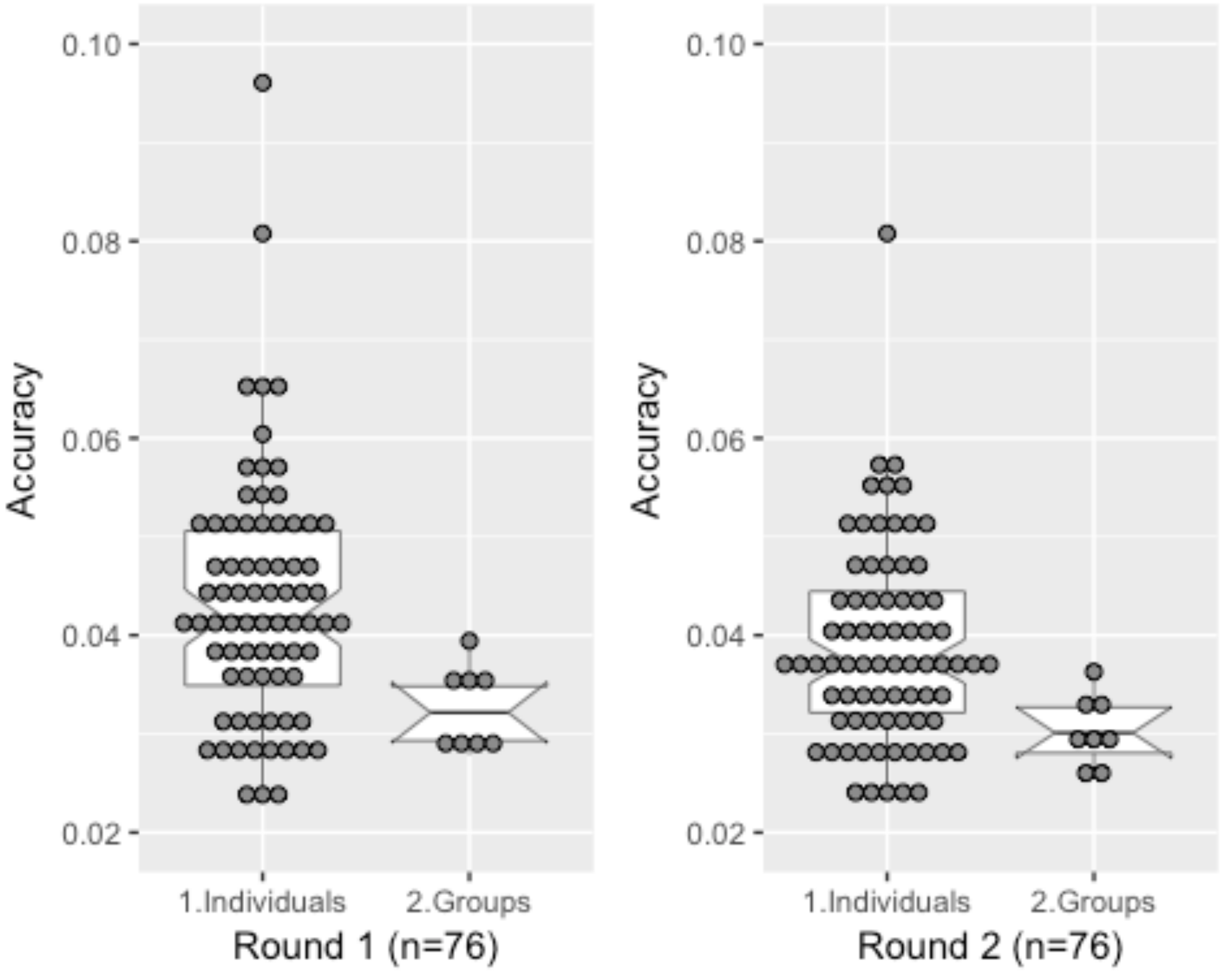

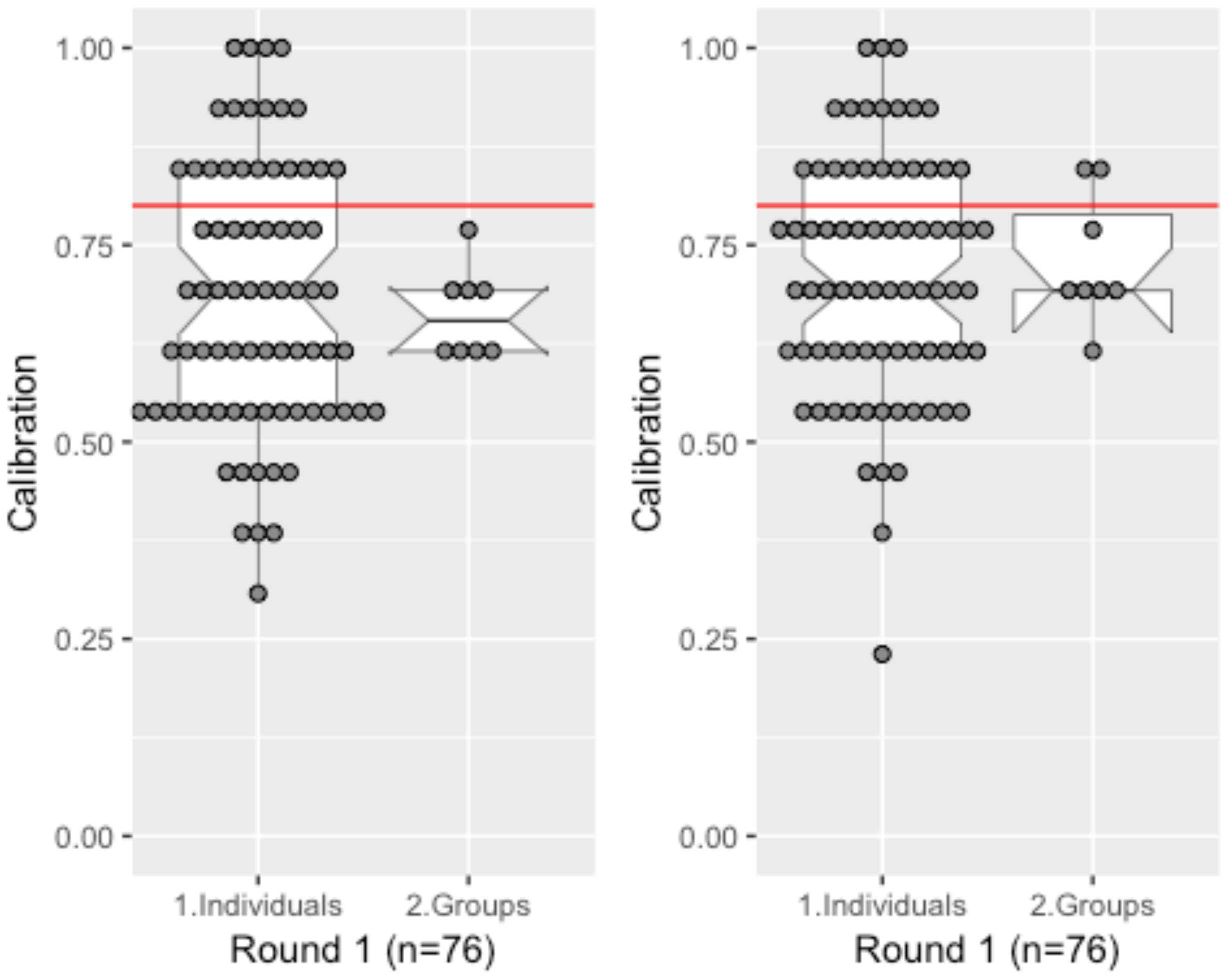

1.00
0.75
0.50
0.25
0.00
Calibration
1.Individuals
2.Groups
Round 1 (n=76)
1.00
0.75
0.50
0.25
0.00
Calibration
1.Individuals
2.Groups
Round 1 (n=76)

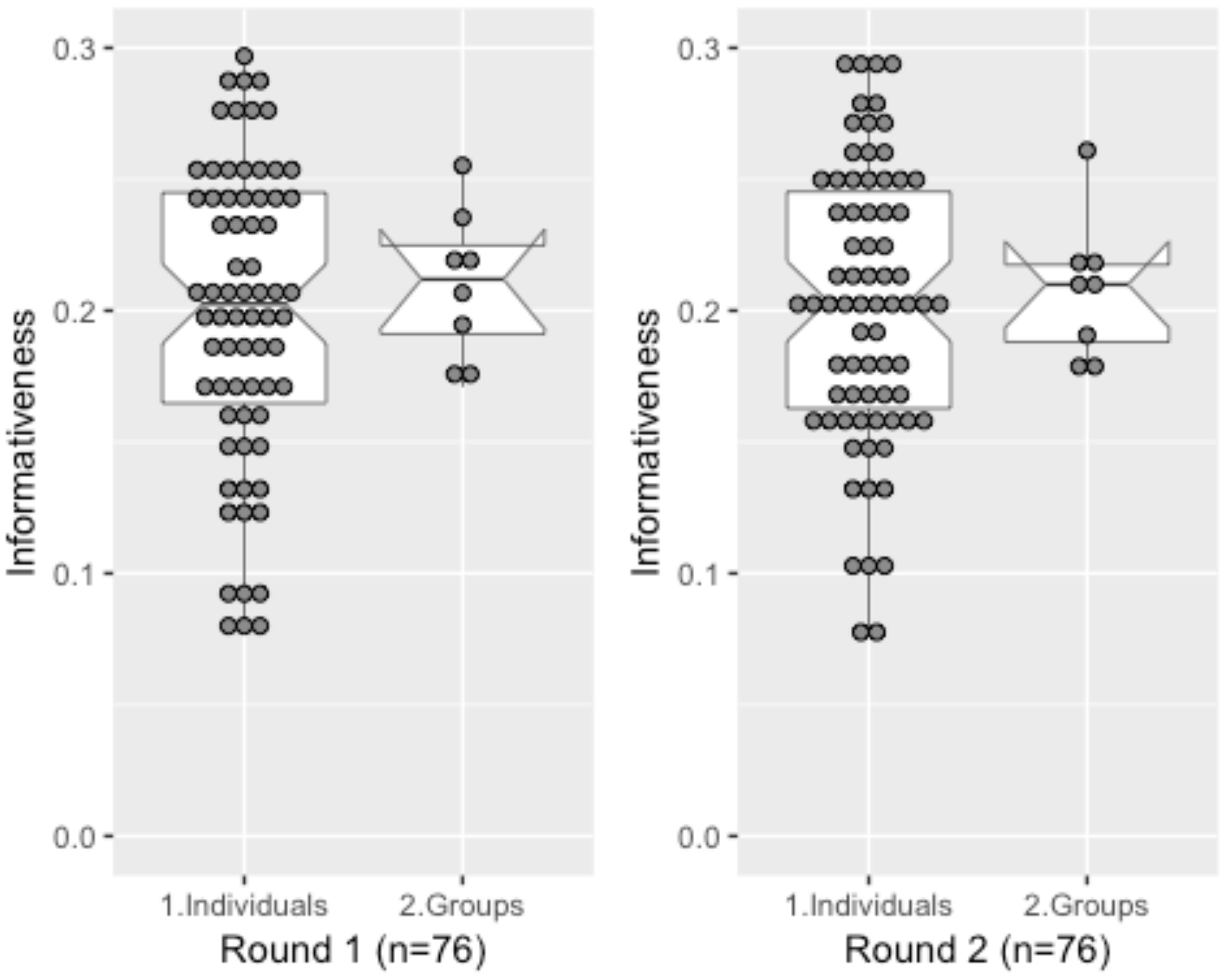


| Test | n | Min | 25th | Median | Mean | 75th | Max | LowMedCI | UpperMedCI | Sd | MAD | Stat |
|---|---|---|---|---|---|---|---|---|---|---|---|---|
| 1.Individuals | 76 | 0.023 | 0.035 | 0.042 | 0.043 | 0.051 | 0.096 | 0.039 | 0.045 | 0.013 | 0.012 | Accuracy R1 (76) |
| 2.Groups | 8 | 0.028 | 0.029 | 0.032 | 0.033 | 0.035 | 0.039 | 0.029 | 0.035 | 0.004 | 0.005 | Accuracy R1 (76) |
| 1.Individuals | 76 | 0.308 | 0.538 | 0.692 | 0.68 | 0.846 | 1 | 0.637 | 0.748 | 0.169 | 0.228 | Calibration R1 (76) |
| 2.Groups | 8 | 0.615 | 0.615 | 0.654 | 0.663 | 0.692 | 0.769 | 0.611 | 0.697 | 0.057 | 0.057 | Calibration R1 (76) |
| 1.Individuals | 76 | 0.07747 | 0.1693 | 0.2059 | 0.2114 | 0.2525 | 0.4715 | 0.1909 | 0.2208 | 0.07139 | 0.0643 | Informativeness R1 (76) |
| 2.Groups | 8 | 0.171 | 0.1911 | 0.2119 | 0.2102 | 0.2248 | 0.2552 | 0.1932 | 0.2306 | 0.02802 | 0.03028 | Informativeness R1 (76) |

| | | | | | | | | | | | | |
|---|---|---|---|---|---|---|---|---|---|---|---|---|
| 1.Individuals | 76 | 0.023 | 0.032 | 0.037 | 0.039 | 0.044 | 0.081 | 0.035 | 0.04 | 0.01 | 0.01 | Accuracy R2 (76) |
| 2.Groups | 8 | 0.026 | 0.028 | 0.03 | 0.03 | 0.033 | 0.036 | 0.028 | 0.033 | 0.004 | 0.004 | Accuracy R2 (76) |
| 1.Individuals | 76 | 0.231 | 0.615 | 0.692 | 0.707 | 0.846 | 1 | 0.651 | 0.734 | 0.154 | 0.114 | Calibration R2 (76) |
| 2.Groups | 8 | 0.615 | 0.692 | 0.692 | 0.731 | 0.788 | 0.846 | 0.639 | 0.746 | 0.082 | 0.057 | Calibration R2 (76) |
| 1.Individuals | 76 | 0.077 | 0.164 | 0.204 | 0.209 | 0.247 | 0.472 | 0.189 | 0.219 | 0.065 | 0.062 | Informativeness R2 (76) |
| 2.Groups | 8 | 0.177 | 0.188 | 0.21 | 0.208 | 0.217 | 0.261 | 0.194 | 0.226 | 0.027 | 0.021 | Informativeness R2 (76) |

## 58 Participants: Group vs individual performance (removing those who withdrew)

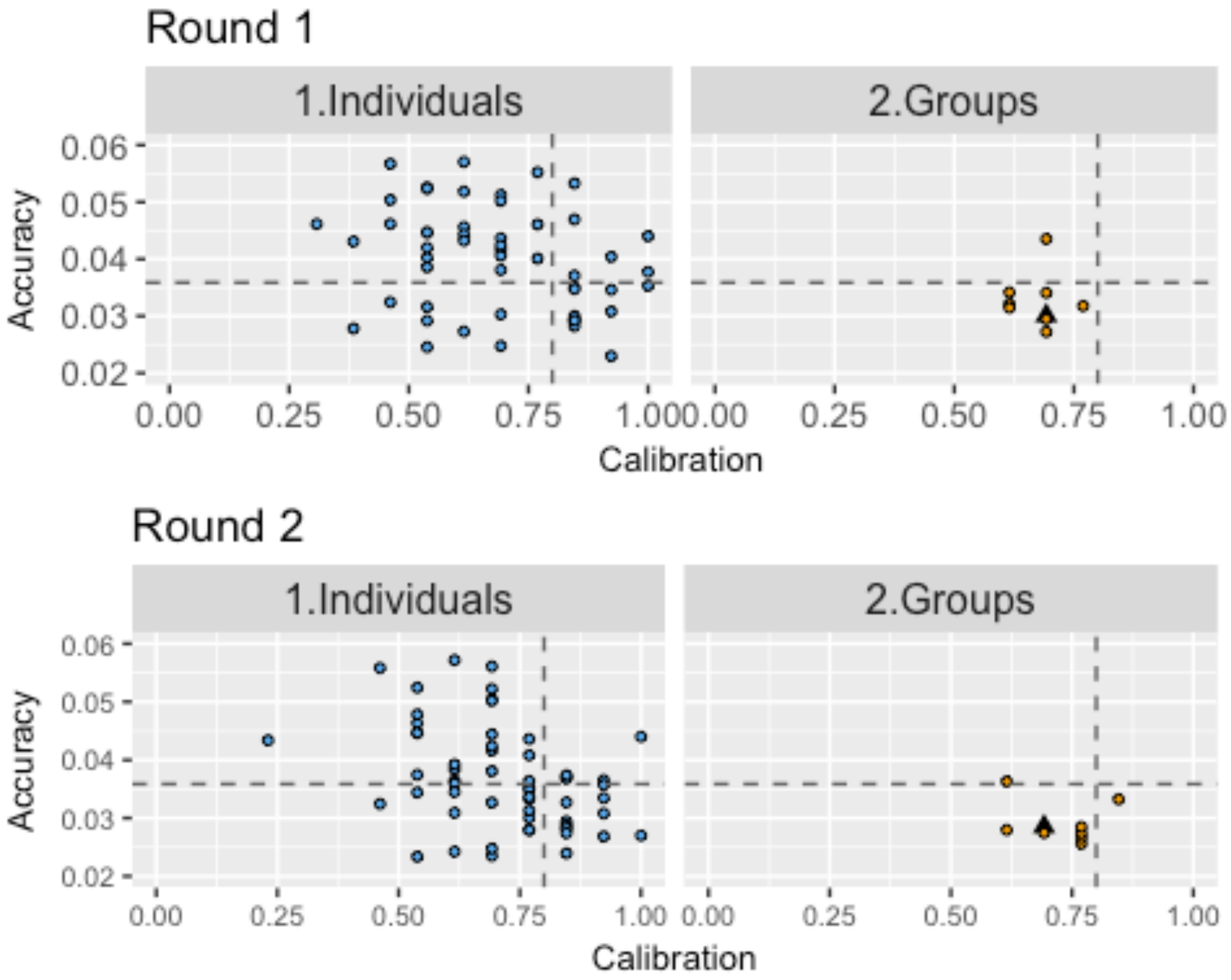


| | Accuracy | Calibration | Informativeness | Round | Code_Name |
|---|---|---|---|---|---|
| **19** | 0.03005 | 0.6923 | 0.2091 | 1 | SuperGroup(n=58) |
| **191** | 0.02853 | 0.6923 | 0.2062 | 2 | SuperGroup(n=58) |

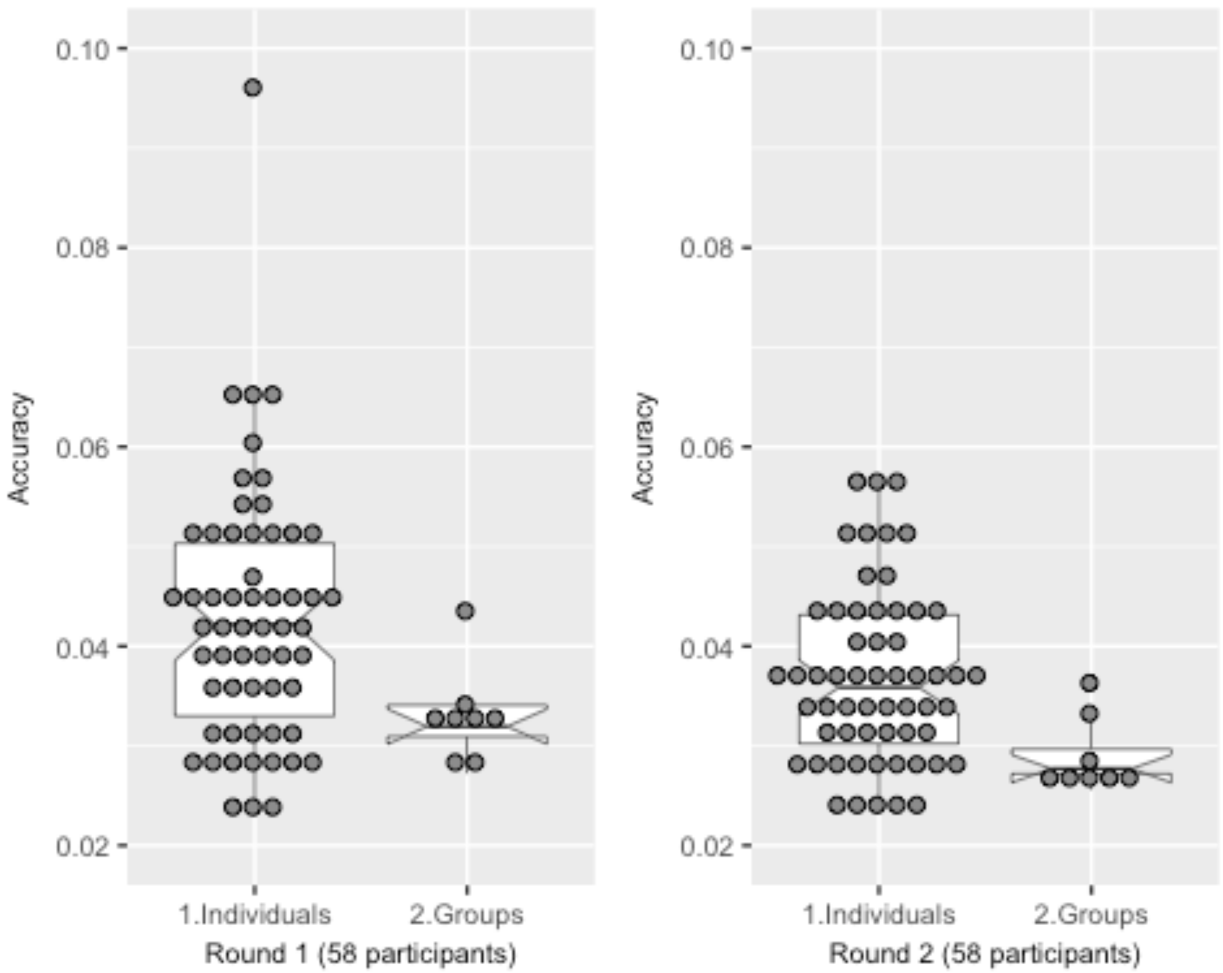

0.10
0.08
0.06
0.04
0.02
Accuracy
1.Individuals
2.Groups
Round 1 (58 participants)
0.10
0.08
0.06
0.04
0.02
Accuracy
1.Individuals
2.Groups
Round 2 (58 participants)

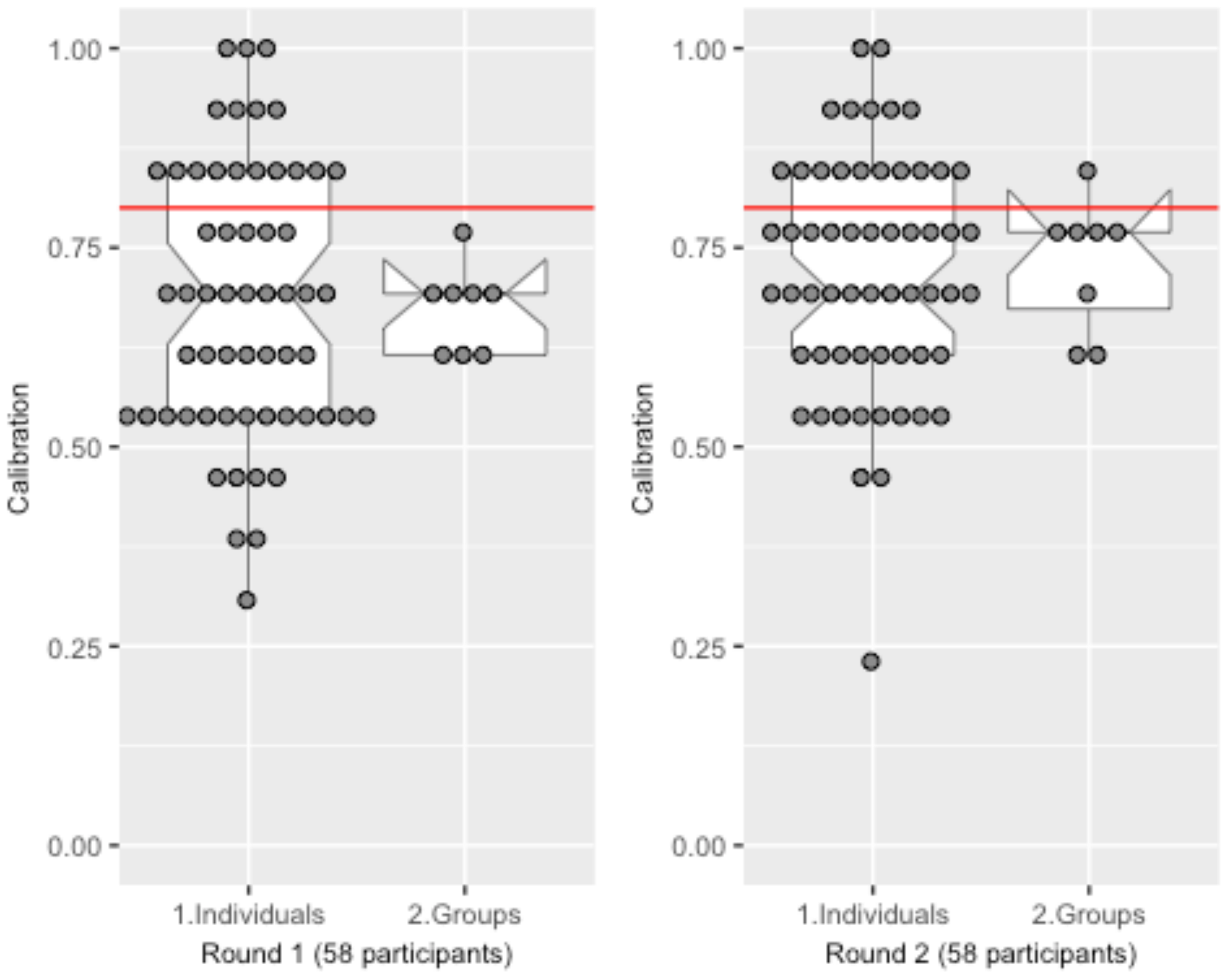


| Test | n | Min | 25th | Median | Mean | 75th | Max | LowMedCI | UpperMedCI | Sd | MAD | Stat |
|---|---|---|---|---|---|---|---|---|---|---|---|---|
| 1.Individuals | 58 | 0.308 | 0.538 | 0.692 | 0.68 | 0.846 | 1 | 0.629 | 0.756 | 0.17 | 0.228 | Calibration R1 (58) |
| 2.Groups | 8 | 0.615 | 0.615 | 0.692 | 0.673 | 0.692 | 0.769 | 0.65 | 0.735 | 0.054 | 0.057 | Calibration R1 (58) |
| 1.Individuals | 58 | 0.231 | 0.615 | 0.692 | 0.716 | 0.846 | 1 | 0.645 | 0.74 | 0.15 | 0.114 | Calibration R2 (58) |
| 2.Groups | 8 | 0.615 | 0.673 | 0.769 | 0.731 | 0.769 | 0.846 | 0.716 | 0.823 | 0.082 | 0.057 | Calibration R2 (58) |

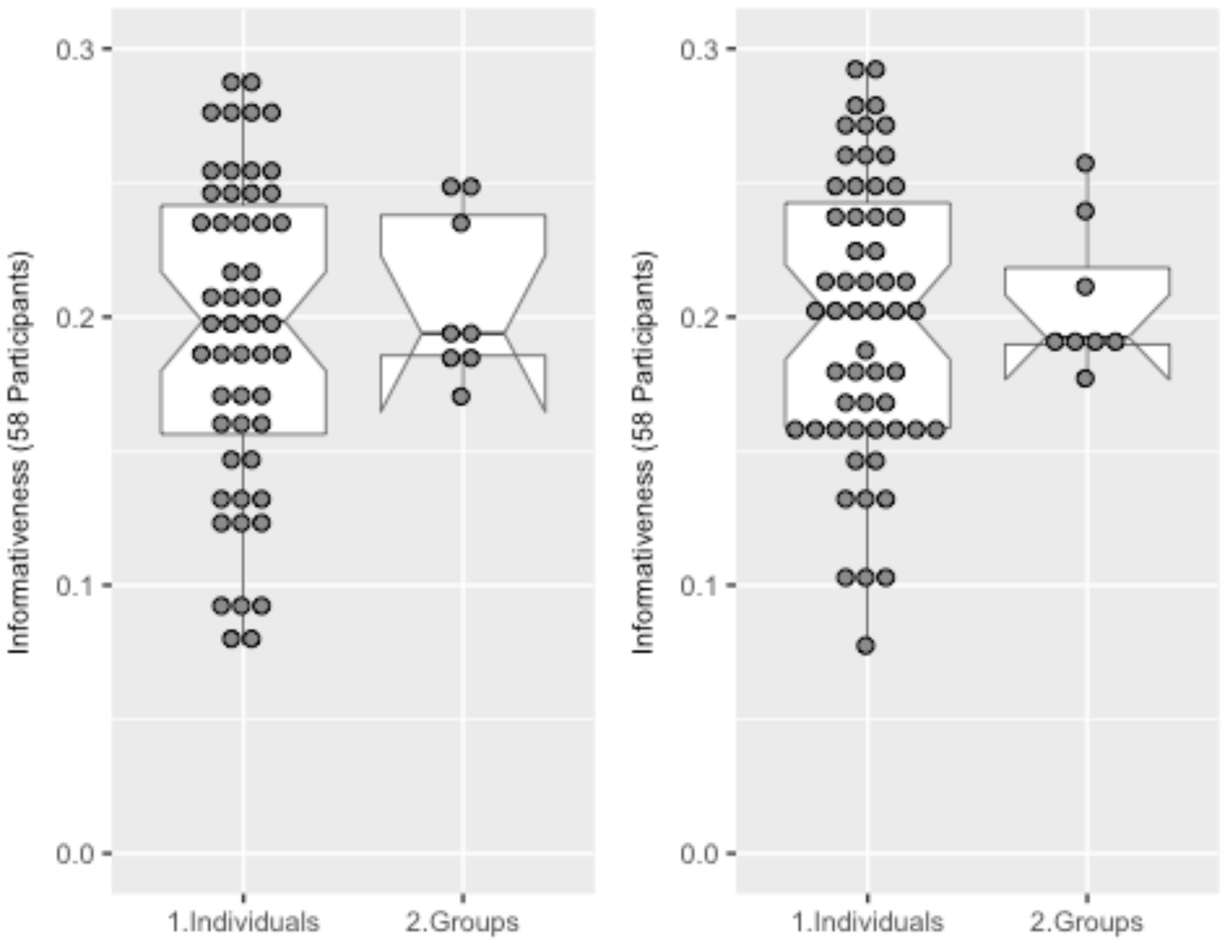


| Test | n | Min | 25th | Median | Mean | 75th | Max | LowMedCI | UpperMedCI | Sd | MAD | Stat |
|---|---|---|---|---|---|---|---|---|---|---|---|---|
| 1.Individuals | 58 | 0.023 | 0.033 | 0.042 | 0.043 | 0.05 | 0.096 | 0.039 | 0.046 | 0.013 | 0.013 | Accuracy R1 (58) |
| 2.Groups | 8 | 0.027 | 0.031 | 0.032 | 0.033 | 0.034 | 0.044 | 0.03 | 0.034 | 0.005 | 0.003 | Accuracy R1 (58) |
| 1.Individuals | 58 | 0.308 | 0.538 | 0.692 | 0.68 | 0.846 | 1 | 0.629 | 0.756 | 0.17 | 0.228 | Calibration R1 (58) |
| 2.Groups | 8 | 0.615 | 0.615 | 0.692 | 0.673 | 0.692 | 0.769 | 0.65 | 0.735 | 0.054 | 0.057 | Calibration R1 (58) |
| 1.Individuals | 58 | 0.07747 | 0.1595 | 0.206 | 0.2091 | 0.252 | 0.4715 | 0.187 | 0.2251 | 0.07375 | 0.06996 | Informativeness R1 (58) |

| | | | | | | | | | | | | |
|---|---|---|---|---|---|---|---|---|---|---|---|---|
| 2.Groups | 8 | 0.1704 | 0.1856 | 0.1938 | 0.2074 | 0.238 | 0.2501 | 0.1648 | 0.2229 | 0.03148 | 0.02571 | Informativeness R1 (58) |
| 1.Individuals | 58 | 0.023 | 0.03 | 0.036 | 0.037 | 0.043 | 0.057 | 0.033 | 0.039 | 0.009 | 0.01 | Accuracy R2 (58) |
| 2.Groups | 8 | 0.026 | 0.027 | 0.028 | 0.029 | 0.03 | 0.036 | 0.026 | 0.029 | 0.004 | 0.001 | Accuracy R2 (58) |
| 1.Individuals | 58 | 0.231 | 0.615 | 0.692 | 0.716 | 0.846 | 1 | 0.645 | 0.74 | 0.15 | 0.114 | Calibration R2 (58) |
| 2.Groups | 8 | 0.615 | 0.673 | 0.769 | 0.731 | 0.769 | 0.846 | 0.716 | 0.823 | 0.082 | 0.057 | Calibration R2 (58) |
| 1.Individuals | 58 | 0.077 | 0.159 | 0.204 | 0.206 | 0.246 | 0.472 | 0.186 | 0.222 | 0.066 | 0.065 | Informativeness R2 (58) |
| 2.Groups | 8 | 0.177 | 0.19 | 0.192 | 0.206 | 0.218 | 0.257 | 0.177 | 0.208 | 0.028 | 0.014 | Informativeness R2 (58) |

## Changes in accuracy + thresholds

*Table 1 Individual and Group changes in Accuracy per question.   Dots above the threshold represent changes in accuracy that would be considered meaningful (e.g. above standard error). Green dots represent improvements, red dots represent reduced accuracy.*

| | | |
|---|---|---|
| 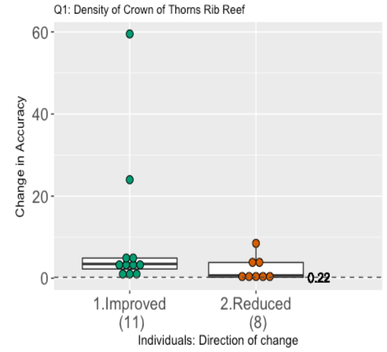 | 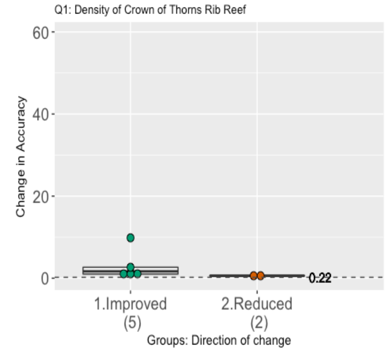 | **Grain Size Threshold:** 0.22<br>**Justification:** Densities above 0.22 are considered incipient outbreaks by AIMS, and those above 1 are considered active outbreaks.<br><br>http://data.aims.gov.au/waCOTSPage/cotspage.jsp |
| 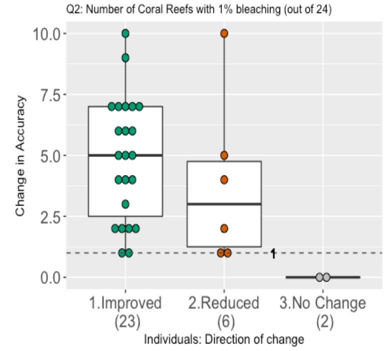 | 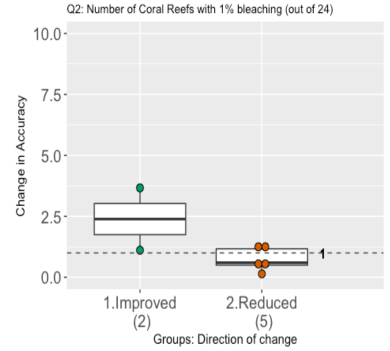 | **Grain Size Threshold:** 1<br>**Justification:** Out of 24 reefs a suitable grain size for improvement would need to be above a single reef. |
| 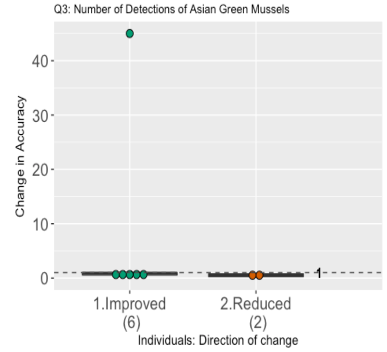 | 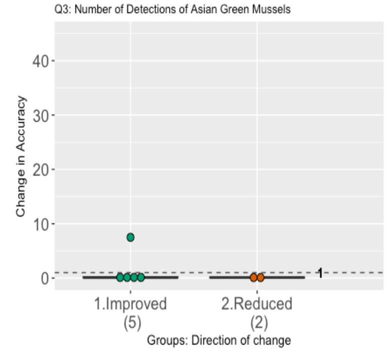 | **Grain Size Threshold:** 1<br>**Justification:** Detections of Asian Green mussels are quite rare (1 detection every 2 years pers. comms. AR., Senior Biosecurity Officer, Department Agriculture and Fisheries QLD email to VH 12/01/2016.  A single detection is therefore meaningful. |
| 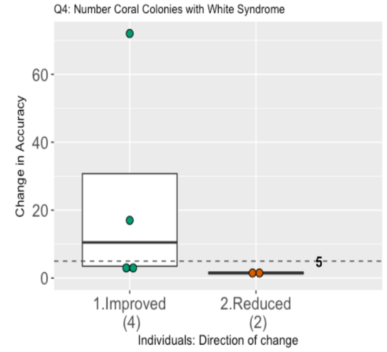 | 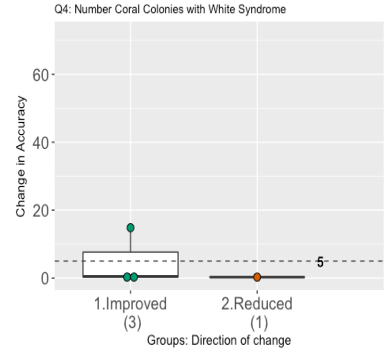 | **Grain Size Threshold:** 5<br>**Justification:** Since 2010, the number of coral colonies affected has been below 5, and only fluctuated by 1 or 2.  A change in 5 would indicate that the disease may be starting to spread again (as opposed to incidental observations).<br><br>http://data.aims.gov.au/reefpage2/rpdetail.jsp?fullReefID=21060S&sampleType=VPOINT |

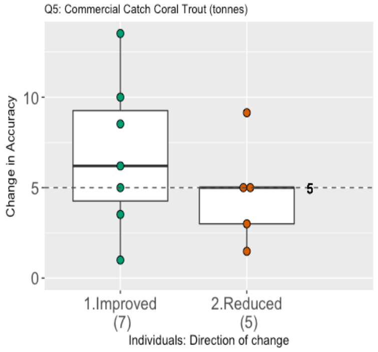


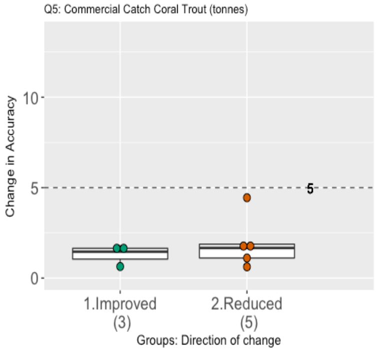


**Grain Size Threshold:** 1
**Justification:** For this question, we used the standard error of recorded catches between 2010 and 2015 (catches: 89.77, 57.15, 76.78, 75.85, 58.57, 66.3)

http://qfish.fisheries.qld.gov.au/query/93670a99-b20f-49bc-84ad-272beff7cb9a/table?customise=True

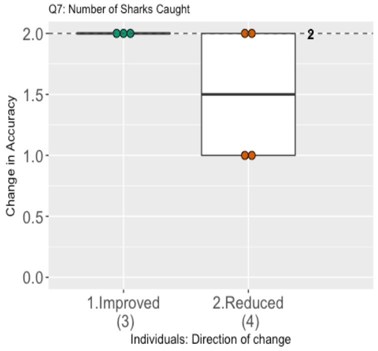


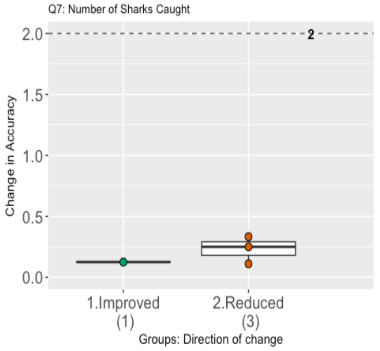


**Grain Size Threshold:** 2
**Justification:** Based on the standard error of catch data between 2010 and 2015 (data: 7, 14, 5, 1, 1, 9)

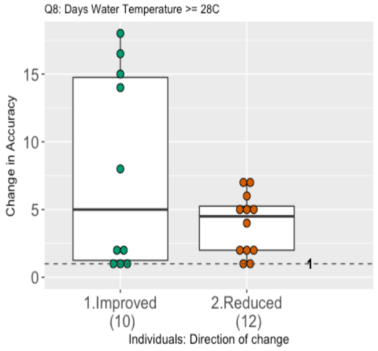


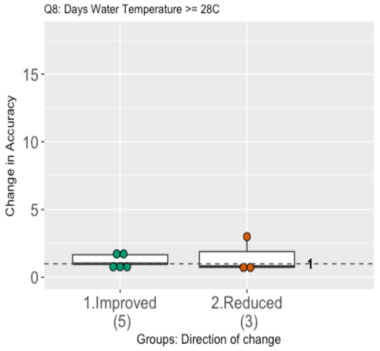


**Grain Size Threshold:** 1
**Justification:** A rounded number of the standard error, based on the data available for this question (2013-2015: 4,3, 7). Standard error is 1.20.

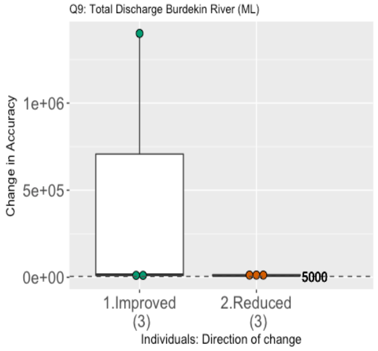


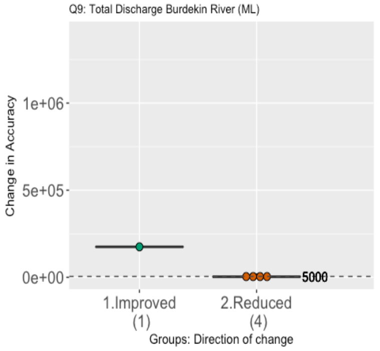


**Grain Size Threshold:** 5000ML
**Justification:** The discharge between 2010 and 2015: 588861.64, 2992521.7, 1287637.85, 130941.06, 524510.95, 70645.68).
448, 688.6183.

This equates to an average discharge for April between 2010 and 2015 was 93,2520 ML with a coefficient of variation of 1.17), thus small differences in judgments would be of little consequence.

Given some years the discharge is approximately 1/6 of this it was not deemed a suitable measure.


65

<table>
<tr><td></td><td></td><td>A threshold of 5000ML was chosen as a reasonable scale for accuracy, and improvements of 5000ML as meaningful</td></tr>
<tr><td>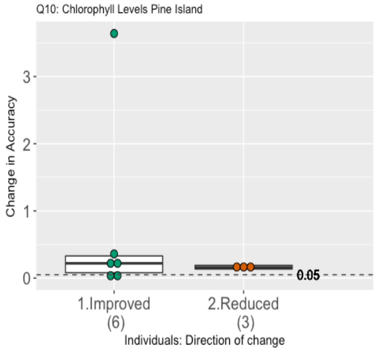
</td><td>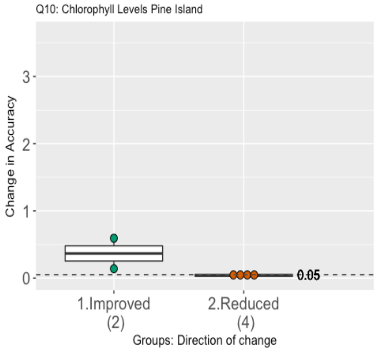
</td><td>Grain Size Threshold: 0.05<br>Justification: Based on standard error of from 2008-2015 (0.03)</td></tr>
<tr><td>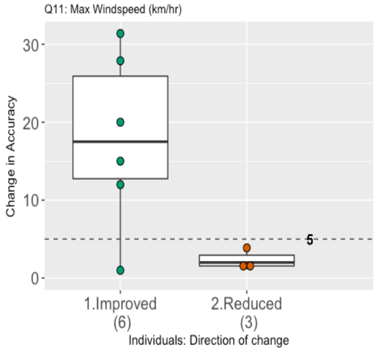
</td><td>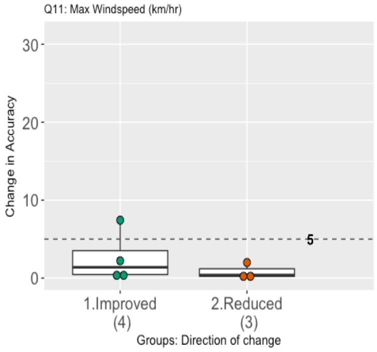
</td><td>Grain Size Threshold: 5<br>Justification: This is based on the standard error of the maximum wind speed for 2010-2015 (67.32, 67.88, 70.2, 86.4, 69.12, 68.4). Which is 2.99km/hr, rounded up to 5 to take account for measurement error.</td></tr>
<tr><td>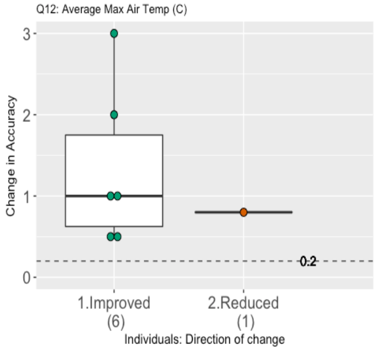
</td><td>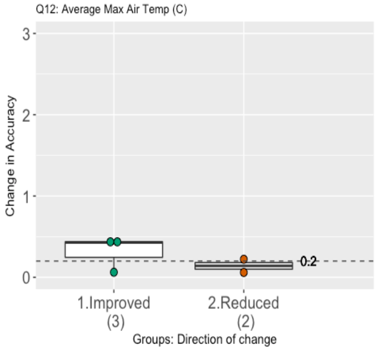
</td><td>Grain Size Threshold: 0.2<br>Justification: Background data for this could not be obtained freely, however, according to BOM small increments in the maximum temperature are meaningful (0.2C).<br><br>http://www.bom.gov.au/climate/current/annual/qld/summary.shtml<br><br>http://www.bom.gov.au/climate/current/annual/aus/#tabs=Temperature</td></tr>
</table>

| | | |
|---|---|---|
| 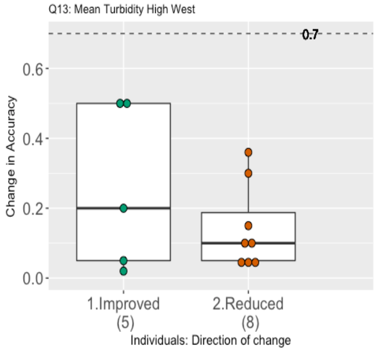  | 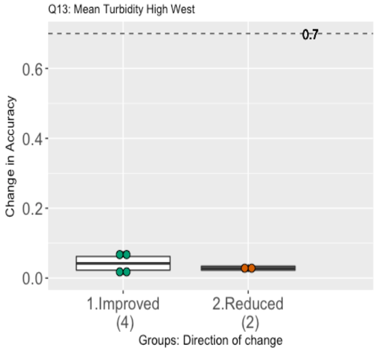  | **Grain Size Threshold:** 0.7<br>**Justification:** This is based on the standard error from 2008-2015 (0.70) |
| 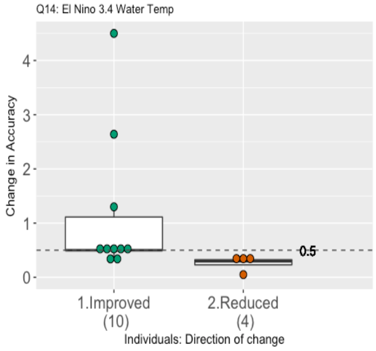  | 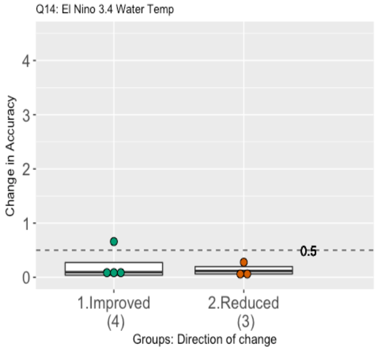  | **Grain Size Threshold:** 0.5<br>**Justification:**Warm and cold phase s are defined as a minimum of five consecutive 3-month running mean of SST anomalies (ERSST.v4, 1971-2000 base period) in the Niño 3.4 region surpassing a threshold **of +/- 0.5°C.**<br><br>https://www.ncdc.noaa.gov/teleconnections/enso/indicators/sst.php |
